# Cord blood lipid correlation network profiles are associated with subsequent attention-deficit/hyperactivity disorder and autism spectrum disorder symptoms at 2 years: a prospective birth cohort study

**DOI:** 10.1016/j.ebiom.2023.104949

**Published:** 2024-01-09

**Authors:** Kristina Vacy, Sarah Thomson, Archer Moore, Alex Eisner, Sam Tanner, Cindy Pham, Richard Saffrey, Toby Mansell, David Burgner, Fiona Collier, Peter Vuillermin, Martin O’Hely, Wah Chin Boon, Peter Meikle, Satvika Burugupalli, Anne-Louise Ponsonby, Barwon Infant Study Investigator Group, Mimi L.K. Tang, Lawrence Gray, Sarath Ranganathan, Peter Sly, Jochen Mueller, Terry Dwyerm, John Carlin

**Affiliations:** a Florey Institute of Neuroscience and Mental Health, University of Melbourne, Parkville 3010, Australia; b Melbourne School of Population and Global Health, University of Melbourne, Parkville 3010, Australia; c Melbourne School of Mathematics and Statistics, University of Melbourne, Parkville 3010, Australia; d Murdoch Children’s Research Institute, Royal Children’s Hospital, Parkville 3010, Australia; e Department of Paediatrics, University of Melbourne, Parkville 3010, Australia; f Department of Paediatrics, Monash University, Clayton 3168, Australia; g Child Health Research Unit, Barwon Health, Geelong 3220, Australia; h School of Medicine, Deakin University, Geelong 3220, Australia; i Metabolomics Laboratory, Baker Heart and Diabetes Institute, Melbourne 3004, Australia; j Baker Department of Cardiovascular Research, Translation and Implementation, La Trobe University, Bundoora, VIC 3086, Australia

**Keywords:** Lipidomics, Cord blood, Fatty acids, Acylcarnitine, Autism spectrum disorder symptoms, Attention-deficit/hyperactivity disorder symptoms

## Abstract

**Background:**

Attention-deficit/hyperactivity disorder (ADHD) and autism spectrum disorder (ASD) are neurodevelopmental conditions with early life origins. Alterations in blood lipids have been linked to ADHD and ASD; however, prospective early life data are limited. This study examined (i) associations between the cord blood lipidome and ADHD/ASD symptoms at 2 years of age, (ii) associations between prenatal and perinatal predictors of ADHD/ASD symptoms and cord blood lipidome, and (iii) mediation by the cord blood lipidome.

**Methods:**

From the Barwon Infant Study cohort (1074 mother-child pairs, 52.3% male children), child circulating lipid levels at birth were analysed using ultra-high-performance liquid chromatography-tandem mass spectrometry. These were clustered into lipid network modules via Weighted Gene Correlation Network Analysis. Associations between lipid modules and ADHD/ASD symptoms at 2 years, assessed with the Child Behavior Checklist, were explored via linear regression analyses. Mediation analysis identified indirect effects of prenatal and perinatal risk factors on ADHD/ASD symptoms through lipid modules.

**Findings:**

The acylcarnitine lipid module is associated with both ADHD and ASD symptoms at 2 years of age. Risk factors of these outcomes such as low income, Apgar score, and maternal inflammation were partly mediated by higher birth acylcarnitine levels. Other cord blood lipid profiles were also associated with ADHD and ASD symptoms.

**Interpretation:**

This study highlights that elevated cord blood birth acylcarnitine levels, either directly or as a possible marker of disrupted cell energy metabolism, are on the causal pathway of prenatal and perinatal risk factors for ADHD and ASD symptoms in early life.

**Funding:**

The foundational work and infrastructure for the BIS was sponsored by the 10.13039/100014555Murdoch Children's Research Institute, 10.13039/501100001778Deakin University, and 10.13039/501100019573Barwon Health. Subsequent funding was secured from the 10.13039/501100016056Minderoo Foundation, the 10.13039/501100007601European Union's Horizon 2020 research and innovation programme (ENDpoiNTs: No 825759), 10.13039/501100000925National Health and Medical Research Council of Australia (NHMRC) and 10.13039/501100001348Agency for Science, Technology and Research Singapore [APP1149047], The William and Vera Ellen Houston Memorial Trust Fund (via HOMER Hack), 10.13039/501100018898The Shepherd Foundation, 10.13039/100012698The Jack Brockhoff Foundation, the Scobie & Claire McKinnon Trust, the Shane O'Brien Memorial Asthma Foundation, the Our Women Our Children's Fund Raising Committee Barwon Health, the Rotary Club of Geelong, the Ilhan Food Allergy Foundation, 10.13039/501100020380Geelong Medical and Hospital Benefits Association, Vanguard Investments Australia Ltd, the Percy Baxter Charitable Trust, and Perpetual Trustees.

**Research in context**

**Evidence before this study**

Neurodevelopmental disorders like attention-deficit/hyperactivity disorder (ADHD) and autism spectrum disorder (ASD) have complex origins involving numerous biological and environmental factors. Lipid metabolism has been suggested to play a role in both ADHD and ASD aetiology, but data have largely been cross-sectional. Several studies have reported discrepancies in lipid profiles, for example, higher levels of long-chain acylcarnitine have been associated with ASD. Prospective studies have noted associations between polyunsaturated fatty acids in cord blood and emotional problems or attention symptoms in later childhood.

**Added value of this study**

Our study extends the current knowledge by implementing a systems biology approach combined with molecular mediation analysis to investigate the impact of cord blood lipid profiles on the risk of ADHD and ASD symptoms at 2 and 4 years of age. We observed that elevated levels of specific lipid networks, particularly those comprising acylcarnitines, are positively associated with subsequent increased ADHD and ASD symptoms. Furthermore, we demonstrated that elevated acylcarnitine levels partly mediate the impact of previously reported risk factors such as lower household income, inflammation, and low Apgar scores on neurodevelopment.

**Implications of all the available evidence**

Our findings underscore the potential importance of the lipid profile at birth in ADHD and ASD, and indicate that the later prenatal and perinatal period is likely to be critical to ADHD and ASD pathogenesis and prevention.

## Introduction

The incidence of neurodevelopmental disorders in early childhood is increasing. Childhood disorders of concern include attention-deficit/hyperactivity disorder (ADHD) and autism spectrum disorder (ASD). ADHD is defined by symptoms of inattention and/or hyperactivity-impulsivity,^1^ and ASD is characterised by difficulties in social interaction and communication, along with restricted and repetitive interests and behaviours.^1^

The incidences of ADHD and ASD are increasing and this is only partially explained by temporal changes in diagnostic criteria.^2^ While the aetiologies of ADHD and ASD remain poorly understood, both multigenetic predisposition and early environment appear important.^3^, ^4^, ^5^, ^6^ ADHD generally becomes apparent during the preschool years,^7^ and children with ASD may have atypical characteristics detected *in utero* by magnetic resonance imaging^8^ and at 3 months by electroencephalogram.^9^ Early environmental risk factors potentially linked to the rising prevalence of ADHD and ASD include a range of manufactured environmental contaminant chemicals and maternal inflammation.^10^ These can lead to changes in fatty acid metabolism^11^ and associated central carbon metabolism.^12^

Lipids are critical for neurodevelopment, and they constitute 50–60% of the brain’s dry weight.^13^^,^^14^ The field of lipidomics has informed our understanding of several brain disorders, particularly in adulthood, such as Alzheimer’s disease.^15^^,^^16^ Lipids comprise many different classes. Fatty acids are the basic lipid monomers and are typically found as free fatty acids or as various classes of esters or ethers, such as triglycerides, glycerophospholipids, sphingolipids, and cholesteryl esters. However, research on lipid metabolism and the lipid profile in early neurodevelopmental disorders, such as ADHD and ASD, is limited, and studies have been mainly cross-sectional.

Cross-sectional studies of ADHD and ASD in children have often, but not always, reported a deficit of circulating omega-3 (such as docosahexaenoic acid, DHA) and an excess of omega-6 (such as arachidonic acid, AA) and total polyunsaturated fatty acids (PUFAs).^17^ An ASD subtype characterised by dyslipidaemia^18^ has been reported, as well as changes in circulating lipoproteins.^19^, ^20^, ^21^, ^22^ Lipid classes of interest associated with ADHD and ASD include cholesterol, phospholipids, and acylcarnitines.^23^, ^24^, ^25^ Several studies have reported higher levels of long-chain acylcarnitine in the blood of children aged 2–11 years with ASD.^21^^,^^26^, ^27^, ^28^, ^29^, ^30^ However, interpretation of these findings is complicated by a range of factors, including potential reverse causation. As ADHD and ASD alter lifestyle, particularly diet, prospective data, where the exposure is measured prior to the outcome,^31^ are required.^32^^,^^33^

One prospective cohort study reported that in cord blood, total-PUFA and the PUFA AA (conjugated to glycerophospholipids) are positively associated with emotional problems in 10-year-old children.^34^ Within the same study, cord blood DHA (conjugated to glycerophospholipids) concentrations were inversely associated with attention symptoms.^34^ DHA and other omega-3 fatty acid supplements have been trialled for both ADHD and ASD, but findings are inconsistent.^35^ Another prospective study reported higher acylcarnitine C18 levels, measured from newborn Guthrie cards shortly after birth, were positively associated with subsequent ASD diagnosis among 3–5-year-old boys but not girls.^36^

Acylcarnitines are part of the carnitine shuttle, which is particularly important in transporting long-chain fatty acids across the mitochondrial membrane.^37^ Thus alterations in plasma acylcarnitines, which are esters of carnitines and fatty acids, are sensitive to shifts in mitochondrial activity.^28^ Consistently higher plasma or serum levels of long-chain acylcarnitines can occur due to energy metabolism disturbances including mitochondrial dysfunction, inflammation, and secondary carnitine deficiency.^28^^,^^38^ In the Barwon Infant Study (BIS) cohort, we recently reported serum maternal metabolomic profiles reflecting impaired energy metabolism at 28-week gestation to be associated with ASD symptoms at 2 and 4 years of age.^12^ A cross-sectional study found that more than one-sixth (17%) of ASD cases also presented with consistent acylcarnitine panel abnormalities.^26^ Overall, these findings indicate that lipidomic profiles reflecting impaired energy metabolism are of potential importance in ADHD and ASD.

Modern molecular analytical approaches allow these issues to be advanced. To apply a systems biology approach, we can employ Weighted Correlation Network Analysis (Weighted Gene Co-expression Network Analysis, WGCNA) that links to functional networks and has been more recently applied to lipidomic data.^39^, ^40^, ^41^, ^42^, ^43^, ^44^ WGCNA allows individual lipid measures to form unsupervised networks, reducing data dimensionality while not depending on current pathway libraries, ontologies, or annotations.^41^

To evaluate whether these lipid profiles contribute causally to neurodevelopmental conditions, we can examine if known prenatal and perinatal risk factors, such as low income, maternal smoking during pregnancy, inflammation, and mode of birth, impact ADHD and ASD through changes in the lipidome.

Molecular counterfactual mediation can be employed to investigate underlying pathways, as in our past work demonstrating that one mechanism by which prenatal factors, such as low maternal education and maternal smoking during pregnancy, negatively affect offspring cognition is through increased inflammation at 28-week gestation.^10^

Here, using lipidomic data from a large, population-derived birth cohort study, we aim to investigate (i) whether cord blood lipidomic profiles are associated with ADHD and ASD symptomology at 2 years of age, (ii) whether prenatal and perinatal risk factors of ADHD and ASD are associated with cord blood lipidomic profiles; and (iii) if and to what extent the cord blood lipidomic profiles mediate the association between prenatal/perinatal risk factors and ADHD/ASD symptoms.

## Methods

The Barwon Infant Study (BIS) consists of 1074 mother-child pairs (including 10 sets of twins) recruited from the Barwon region (Victoria, Australia) using an antenatal sampling frame between July 2010 and July 2013. Population characteristics, eligibility criteria, and data collection details have been previously described.^45^ Exclusion criteria included: delivery before 32 weeks, genetic disease or major congenital malformation diagnosis, and serious illness. The broad aim of BIS is to investigate the potential mediators of environmental risk factors, including the epigenome, microbiome, and metabolome, on clinically relevant immune, respiratory, cardiovascular, and neurodevelopmental outcomes.^45^

### Ethics

The study was approved by the Barwon Health Human Research Ethics Committee (HREC 10/24), and families provided written informed consent. This study complies with the requirements of the HREC.

### Blood collection and processing

Peripheral blood was collected from mothers at 28 weeks of gestation and from the umbilical cord blood at birth, which was then placed into serum clotting tubes (BD Vacutainer)^46^ for serum isolation by centrifugation. Venous peripheral blood was collected from infants at the 6-month, 12-month, and 4-year time points in sodium heparin.^47^ Time from blood collection to processing and storage plus overall length of time at −80 °C was recorded and accounted for in subsequent analysis. Cord blood samples were checked for maternal blood contamination using DNA methylation profiling as described elsewhere.^48^^,^^49^ Contamination status (yes/no) was included as a covariate in analyses.

### Lipidomics profiling

Lipid measures were obtained from maternal blood serum samples at 28 weeks of gestation (n = 1032), cord blood serum samples (n = 920), and child blood plasma samples at 6 months (n = 793), 12 months (n = 735) and 4 years (n = 511) and were analysed using ultra-high-performance-tandem mass spectrometry (UHPLC-MS/MS).

The details of the UHPLC-MS/MS lipidomics platform and analysis have been described elsewhere.^46^ Briefly, we quantified 776 lipid features in 36 lipid classes. The lipids were extracted using butanol:methanol (1:1) with 10 mM ammonium formate containing a mixture of internal standards. UHPLC-MS/MS was performed on an Agilent 6490 QQQ mass spectrometer with an Agilent 1290 series UHPLC system and two ZORBAX eclipse plus C18 column (21 × 100 mm, 18 mm, Agilent) with the thermostat set at 45 °C. Mass spectrometry analysis was performed in both positive and negative ion mode with dynamic scheduled multiple reaction monitoring.

Lipid species concentrations were quantified by comparison to the relevant internal standards. The concentrations of each total lipid class were calculated by summing the concentrations of the lipid species belonging to that class. For triacylglycerols (TGs) and alkyl-diacylglycerols, both neutral loss and single ion monitoring (SIM) peaks were measured, and the SIM concentrations were used for the summation. Data processing and quantification have been described previously.^46^

### Metabolomics profiling

Targeted metabolomic profiles (mmol/L) were also obtained from maternal peripheral blood (n = 1032) and cord blood (n = 920) and analysed using nuclear magnetic resonance (NMR) at Nightingale Health, Helsinki, Finland, with details described elsewhere.^12^ The analysis was restricted to metabolites related to central carbon metabolism and inflammation, which included amino acids, n = 9 measures; ketone bodies, n = 3; glycolysis-related metabolites, n = 5, as well as the inflammatory marker, Glycoprotein acetylation (GlycA).

### Attention-deficit/hyperactivity disorder and autism spectrum disorder symptom measures

While ADHD and ASD can be detected at a relatively young age, the median age of diagnosis for ADHD is 6 years,^50^ and for ASD it is 5.9 years.^51^ To assess the early life impact of prenatal and perinatal risk factors, as compared to postnatal exposures, it is important to look at outcomes in early childhood. In the preschool child, we measured ADHD and ASD-related symptoms using the Child Behavior Checklist for ages 1.5–5 (CBCL) at 2 years of age and the Strengths and Difficulties Questionnaire P4-10 (SDQ) at 4 years of age. We used the DSM-5-oriented attention-deficit/hyperactivity problems (CBCL-ADHP) and autism spectrum problems (CBCL-ASP) subscales of the CBCL and the following SDQ subscales: hyperactivity (SDQ-hyper), peer problems (SDQ-peer), and prosocial (SDQ-prosocial). The child’s parent/guardian completed the questionnaires. Subscale scores were calculated by summing behavioural statement responses (CBCL-ADHP: 6 items, range 0–12; CBCL-ASP: 12 items, range 0–24; SDQ subscales: 5 items, range 0–10). The CBCL-ASP has high accuracy in distinguishing pre-schoolers with early doctor-diagnosed ASD in this study (area under the curve, AUC, 0.92).^52^ For CBCL-ADHP, the AUC among 3-year-old children is 0.87 and 0.80 for girls (n = 238) and boys (n = 276).^53^ The test-retest reliability in children aged 3–5 years for the CBCL-ADHP is *r* = 0.74 and for CBCL-ASP, it is *r* = 0.86.^54^

### Other factors

Prenatal factors were obtained from maternal questionnaires, antenatal records, and clinical examination. Maternal perceived stress during trimesters 1 and 2 was measured by the Perceived Stress Scale (PSS).^55^ Perinatal factors were validated with hospital records, including mode of delivery reported as unassisted vs. assisted (vacuum or forceps), caesarean birth reported as planned vs. emergency, as well as duration of labour, child’s assigned sex at birth, gestational age at birth, Apgar score at 5 min, and low birth weight reported as <2.5 kg vs. ≥2.5 kg. Further detail on the full set of other factors can be found in Supplementary Table S4.2.

### Statistical methods

#### Weighted Gene Co-expression Network Analysis (WGCNA) for cord blood lipids

Lipids in the body interact within complex networks and pathways, assuming multiple roles, and blood levels tend to correlate with each other. WGCNA, a systems biology method and dimensionality reduction technique initially used for identifying gene networks via co-expression patterns,^56^ was applied to our lipidomic data. This analysis aids in discovering clusters of lipid concentrations that co-vary, suggesting involvement in shared biological pathways. Furthermore, WGCNA is data-driven, and the lipid modules are formed unsupervised (modules are not formed by predefined outcomes) and without the input of existing pathway libraries. The findings can link specific lipid sets to ADHD or ASD outcomes, providing insight into associated lipidomic profiles or pathways.

We obtained concentrations of 776 lipid features across 36 classes from UHPLC-MS/MS, as previously described. These concentrations were log2-transformed and normalized to z-scores. The WGCNA package^57^ in R (version 4.1.0) constructs a weighted adjacency matrix from the Pearson correlation coefficient values of the normalised lipid concentrations, which is the default correlation type in WGCNA.^57^ The adjacency matrix is elevated to the power of a soft threshold, emphasising the strongest connections.^56^^,^^57^ To determine the soft threshold value, we plotted the scale-free topology model fit index (R^2^) against powers from 1 to 20. The lowest power where the curve plateaued near R^2^ = 0.80 was chosen to ensure a good fit to a scale-free topology. A topological overlap matrix (TOM) was calculated to gauge network interconnectivity.^56^^,^^57^ We opted to utilise the default unsigned networks (accounting for both negative and positive correlations as connected) over the signed ones (where negative correlations are deemed unconnected). This is because in biochemical pathways, negatively correlated metabolites may still be on the same pathway as precursors and products, and prior work suggests these can appear in the same module.^41^

A dendrogram was created from a dissimilarity matrix (1-TOM) using average hierarchical clustering. The dynamic tree cutting algorithm was used to define modules. We selected a minimum module size of 10 and a merging threshold for similar modules at a correlation of 0.75 (merge cut height of 0.25), which has been used previously.^39^^,^^43^ Each module was randomly assigned a colour label for convenience. We also added the abbreviation of the lipid class of the module’s hub lipid for readability. If the module comprised multiple classes, the word ‘hub’ was appended to indicate this is the hub lipid’s class. Abbreviations are defined in the Fig. 4 footnote.

The *WGCNA* R package^57^ represents each lipid module by the first principal component (PC1) of the standardised lipid module concentrations, labelled as the ‘eigenlipid’. The parameter ‘align’ was set to “along average” so that when the orientation of the eigenlipid was undetermined, the orientation was aligned with average lipid concentration.^57^ Thus, positive values of the eigenlipid were interpreted as increased pathway activity.

In summary, the inputs were the transformed and normalised cord blood lipid concentrations obtained from UHPLC-MS/MS, as described above. The main parameters adjusted from their default values were the soft threshold and the minimum module size.

#### Non-oxidative pyruvate metabolism score (NOPMS)

Impaired oxidative energy metabolism can result in upregulation of alternative and substantially less efficient non-oxidative energy metabolism.^12^ A key non-oxidative energy pathway is the conversion of pyruvate to lactate; however, pyruvate can also be converted into acetate and alanine. Previously, we derived a summary score capturing these pathways, NOPMS, and found it to be associated with greater ASD symptoms at age 2 and 4 years.^12^ Briefly, principal component analysis was run on metabolite concentrations of pyruvate, lactate, alanine, and acetate, and the first principal component (PC1) was extracted as a composite measure from both maternal blood and cord blood.

#### Regression models

Separate minimally adjusted multivariable linear regression models estimated the strength of association between each of the 15 eigenlipids and each of the behavioural outcomes at 2 and 4 years. Model adjustments included child's age at assessment, assigned sex at birth, time to serum freezing, time in freezer, and maternal cord blood contamination.^48^^,^^49^ Robust confidence intervals were calculated using the Huber-White sandwich estimator due to detected model heteroscedasticity. In each model that was fitted, complete cases were used. We also fit a mutually adjusted model containing all 15 lipid modules and the other adjustment factors. We established that with 555 subjects and an 80% power level, we can detect a minimum standardized effect size (delta) of 0.026 for our exposure variable within a multiple linear regression model with 7 predictors, corresponding to a detectable R-squared of 0.025, with a significance level of 5%.

We examined potential confounders for 2-year CBCL outcomes using the comprehensive information on potential confounders available in the BIS cohort and models with a more extensive set of adjustment factors were also run. For this, we considered the three lipid modules’ eigenlipids which correlated strongest in magnitude with the outcomes CBCL-ADHP and CBCL-ASP. To inform our multivariable analysis, prior knowledge, including from our past work^12^^,^^49^^,^^52^^,^^58^ was used to construct a directed acyclic graph (DAG)^59^ which is a visual tool to represent the relationships between variables in order to map out how factors affect the outcome. This was done for each of the six exposure-outcome relationships. As in past work,^10^^,^^12^^,^^49^ a data-adaptive approach was used to check that inappropriate factors were not included as confounders in the models where *a priori* knowledge of the highly dimensioned lipidomic measures was low.^60^ A list of confounders that were evaluated is listed in Supplementary Table S1 and a correlation matrix of Pearson’s correlations between exposures, outcomes, and covariates can be found in (Supplementary Figure S2). Due to the similarity of adjustment factors between each of the exposure-outcome relationships, we arrived at a single confounder set for each of the three lipids and two outcomes at age 2 (Supplementary Figure S1.1). These factors were also applied to the age 4 outcomes.

As a sensitivity analysis, we dichotomised each CBCL-ADHP and CBCL-ASP outcome into the 85th percentile and greater, compared to the 15th percentile and lower, and conducted minimally adjusted logistic regression analyses to investigate if the eigenlipid-CBCL associations found in the main analysis persisted.

### Mediation analysis

Biomeasures are more likely to be causal if they mediate the effects of known risk factors. Therefore, we investigated whether the lipid modules mediated the relationship between risk factors of ADHD/ASD and our CBCL outcomes. Similar to our past work,^10^ we first used minimally adjusted linear regression models to examine the associations between early life risk factors of ADHD or ASD (as exposures) and cord blood eigenlipids (as outcomes). Eigenlipids from each lipid module that were significantly associated with both an early life exposure and a neurodevelopmental outcome were then each tested as mediators in formal counterfactual mediation analyses. These risk factors included low household income, mode of birth, and Apgar score, as well as metabolic risk factors like non-oxidative pyruvate metabolism and inflammation. The total effect of the early life exposure on ADHD or ASD symptoms at 2 years of age was parsed into two components: the natural direct effect (the portion of the effect not acting through lipid levels) and the natural indirect effect (the portion of the effect acting through lipid levels; Supplementary Figure S1.2).

### Additional analysis

To test whether the time of day of cord blood collection influenced results, sinusoidal and cosinusoidal functions were included as adjustment factors in minimally adjusted regression models.

To exclude the impact of perinatal events on the lipid-module-outcome relationship for Cyan-AC, we also restricted the dataset to uneventful births (unassisted vaginal births, no resuscitation required at birth, Apgar Score >8 at 5 min and no prolonged labour with labour duration ≤ 18 h, n = 260), and re-fit regression models with Cyan-AC, the module containing acylcarnitines, as the exposure, and CBCL-ADHP or CBCL-ASP as the outcome.

To determine if any of the lipid modules were associated with the 2-year outcomes independently of other lipid modules, we fit minimally adjusted regression models with two lipid modules (modules listed in Fig. 4). A full model with all lipid modules and the minimal set of adjustment factors was then examined.

To determine if the same lipid networks at other timepoints were also associated with the outcomes, we took the four top (most associated with CBCL-ADHP and CBCL-ASP by magnitude and *p*-value) lipid modules discovered by WGCNA at birth and calculated eigenlipids for these modules at four other timepoints. We did this by running PCA on maternal (28-week gestation) blood lipids as well as the child’s blood lipids at 6 months, 12 months, and 4 years of age for each lipid module and extracting PC1. We did not undertake extensive confounder adjustment for these additional timepoints.

As the metabolite measures for birth NOPMS and birth GlycA were cross-sectional with birth lipids and were utilised in mediation analyses, we performed an additional sensitivity analysis. In this analysis, we reversed the positions of the exposure and the mediator. Consequently, the lipid module eigenlipid became the exposure, and the birth NOPMS or birth GlycA served as the mediator (Supplementary Figure S1.2).

We used inverse probability weighting to assess the potential influence of sampling bias introduced by differences between participants with complete relevant data and all other individuals in the eligible cohort (i.e., participants with missing data and non-participants). We used covariates that we also collected from non-responders, which were: maternal age during trimester 1 or 2, maternal, paternal or sibling asthma status, maternal, paternal or sibling eczema status, SEIFA disadvantage tertile (based on ABS 2011 data), remoteness area (based on ABS 2011 data) and household size. Stabilised weights were used.

Statistical analyses were performed using Stata 17.0 and R 4.1.0. WGCNA was implemented using the *WGCNA* R package with modules generated using the blockwiseModules() function.^57^ Mediation analysis with bootstrap standard errors was implemented using the *medflex* R package.^61^ Nominal statistical significance was set at *p* < 0.05. The Benjamini–Hochberg (BH) procedure with a 5% false discovery rate was applied to account for multiple hypothesis testing for the 2-year and 4-year outcomes.

### Role of funders

The funders of the study had no role in study design, data collection, data analysis, data interpretation, or writing of the report. The corresponding author had full access to all the data in the study and had final responsibility for the decision to submit for publication.

## Results

### Cohort characteristics

**Fig. 1:**
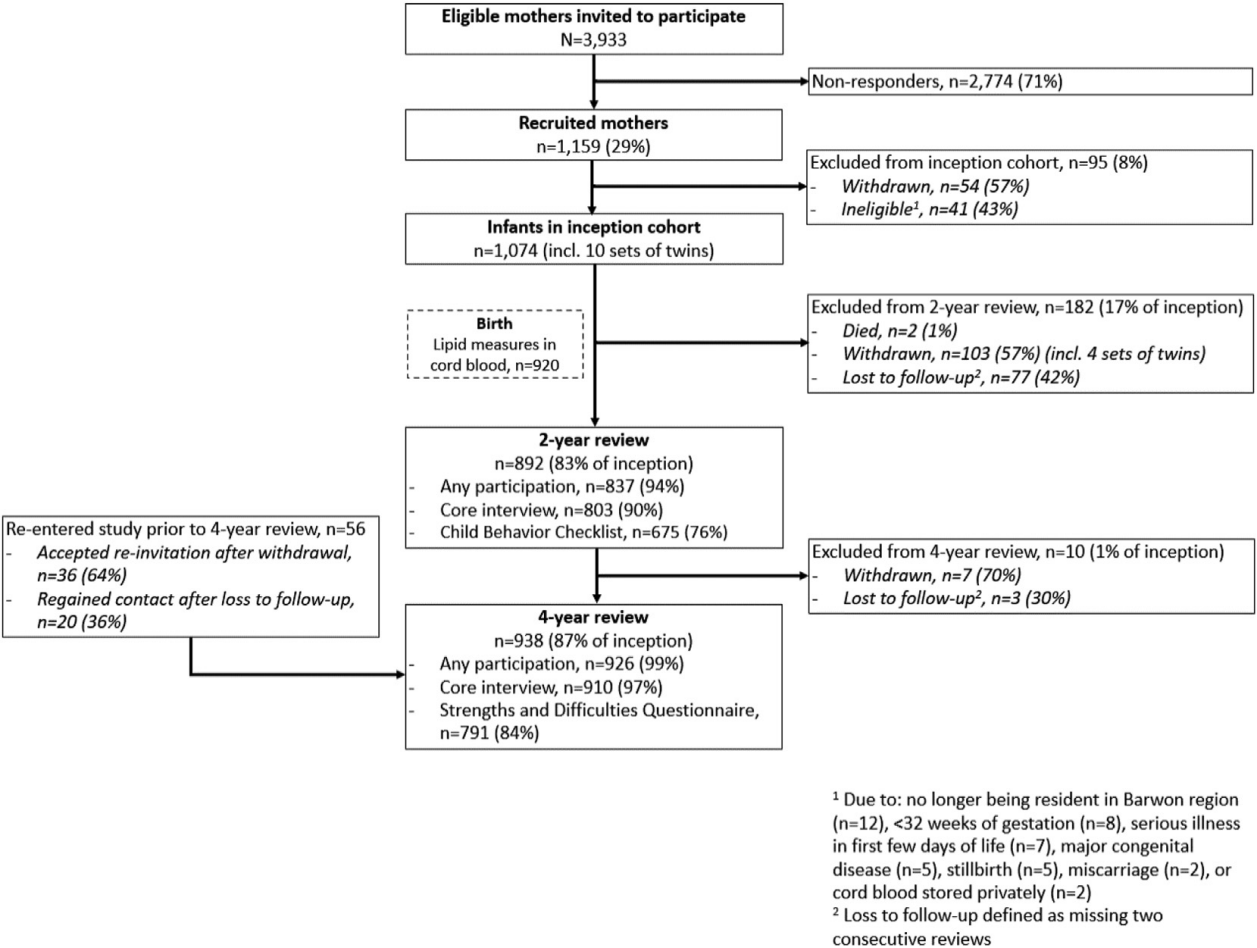
**Barwon Infant Study participant flowchart.** Participants included in each analysis were those with complete data on the variables of interest.

Cohort characteristics are shown in Table 1, and the participant flow chart for this study is included in Fig. 1. Of the children with available lipidomic data (n = 920), 52% were male and 29% were born via caesarean section. Median scores of the outcomes were: 3 for CBCL-ADHP, 1 for CBCL-ASP, 3 for SDQ-Hyper, 1 for SDQ-Peer, and 8 for SDQ-Prosocial.

**Table 1:** Cohort characteristics.

Characteristics	Cord blood lipidome sample N = 920	
	N	Mean (SD) or median [IQR] or % (n)
Parent and household factors		
Maternal age at conception (years)	920	31.39 (4.76)
Paternal age at conception (years)	879	33.61 (5.93)
All 4 grandparents European (yes vs. no)	916	94.9% (869)
Maternal education (university vs. other)	917	52.3% (480)
SEIFA disadvantage (low tertile vs. other)	913	33.6% (307)
Household income ($10,000 AUD)^a^	905	9.42 (3.37)
Prenatal factors		
Mother's weight at 28-week interview (kg)	739	80.93 (15.26)
Mothers weight grain during pregnancy >20 kg (yes vs. no)	466	11.2% (52)
Mother smoked during pregnancy (any vs. none)	914	14.9% (136)
Mother consumed alcohol during pregnancy (any vs. none)	855	52.5% (449)
Mother’s PSS score over trimester 1 and 2	690	18.72 (6.81)
Lone parent during pregnancy (yes vs. no)	920	3.8% (35)
Mother is multiparous (yes vs. no)	919	56.1% (516)
Glycoprotein acetyls, maternal 28 weeks serum (mmol/L)	892	1.63 (0.20)
Maternal NOPMS during pregnancy (PC1)^b^	892	0.01 (1.21)
Gestational age at blood collection (weeks)	899	28.26 (1.21)
Birth factors		
Labor time (hrs)	919	6.04 (5.69)
Gestational age at birth (weeks)	920	39.49 (1.43)
Prematurity (<37 vs. 37 or more weeks)	920	3.8% (35)
Child birthweight <2.5 kg	920	2.1% (19)
Child assigned sex at birth (male vs. female)^c^	920	52.3% (481)
Mode of birth	920	
Unassisted vaginal birth		50.0% (460)
Assisted vaginal delivery		20.8% (191)
Emergency caesarean		12.2% (112)
Elective caesarean		17.1% (157)
Resuscitation at birth (intubation or cardiac compressions vs. other/nil)	920	0.8% (7)
Apgar score at 5 min	908	9.02 (0.82)
Glycoprotein acetyls, cord blood serum (mmol/L)	908	0.75 (0.19)
NOPMS at birth (PC1)^b^	880	−0.01 (1.08)
Maternal contamination of cord blood serum (yes vs. no)	871	4.4% (38)
Cord blood serum time from collection to storage (minutes)	892	2050.70 (8462.70)
Cord blood serum storage time from collection to analysis (weeks)	893	329.01 (42.86)
Child ASD and ADHD symptoms		
CBCL/1.5–5 autism spectrum problems subscale (raw score)	587	1.00 [0.00, 2.00]
CBCL/1.5–5 attention-deficit/hyperactivity problems subscale (raw score)	587	3.00 [1.00, 5.00]
Child’s age at CBCL/1.5–5 assessment (years)	587	2.46 (0.15)
SDQ P2-4 hyperactivity/inattention subscale	686	3.00 [2.00, 5.00]
SDQ P2-4 peer relationship problems subscale	686	1.00 [0.00, 2.00]
SDQ P2-4 prosocial behaviour subscale	687	8.00 [6.00, 9.00]
Child’s age at SDQ P2-4 assessment (years)	689	4.16 (0.26)

SD, standard deviation; IQR, interquartile range; SEIFA, Socio-Economic Indexes for Areas; IRSD, Index of Relative Socio-economic Disadvantage; CBCL, Child Behavior Checklist; SDQ, Strengths and Difficulties Questionnaire; ADHD, attention-deficit/hyperactivity disorder; ASD, autism spectrum disorder; min, minutes; hrs, hours; PSS, Perceived Stress Scale.

a Mean household income during pregnancy and 1st year of child's life.

b NOPMS, non-oxidative pyruvate metabolism score, PC1 of pyruvate, lactate, acetate, and alanine.

c There were no known intersex children in the cohort.

### Construction of co-expression modules

**Fig. 2:**
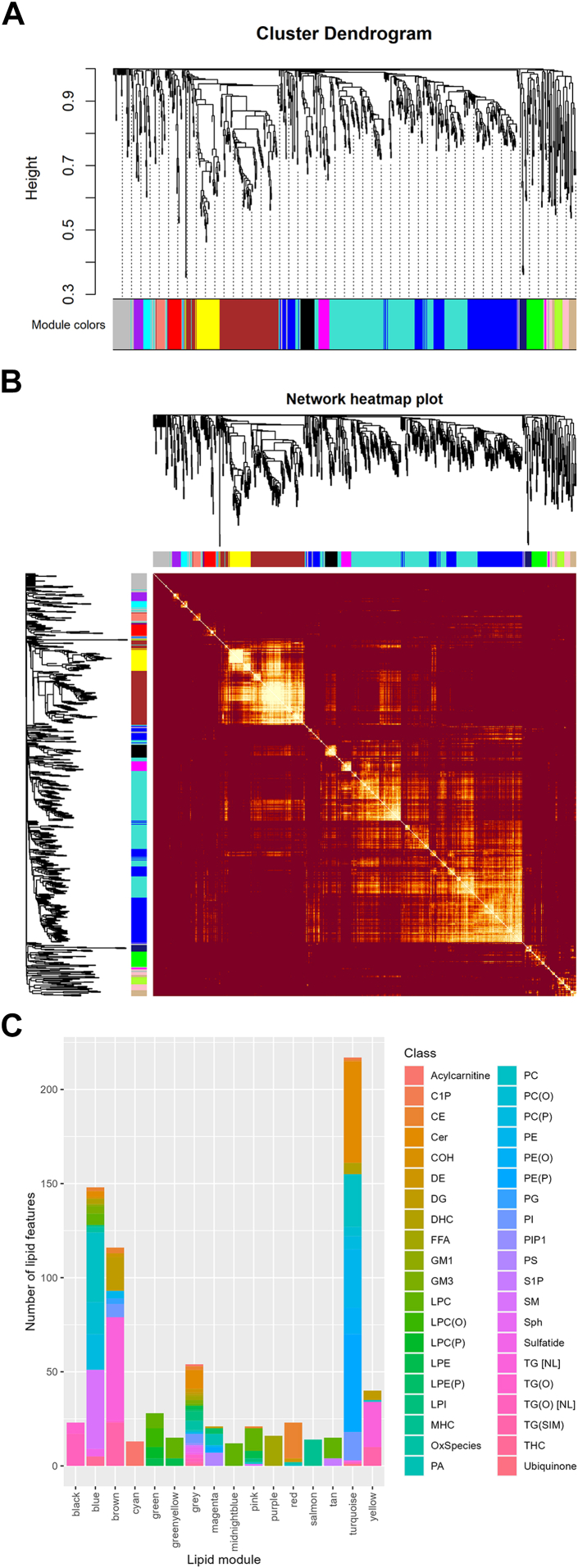
**Construction of modules using WGCNA. (A)** The cluster dendrogram built by hierarchical clustering of the dissimilarity (1-TOM) matrix, where dissimilarity is represented by height. Each colour represents one module and grey are unclustered lipids. **(B)** Heatmap representation of the lipid network's topological association. Within this heatmap, each row and column represents a specific lipid feature. Pale shades indicate minimal topological association, while deeper shades of red indicate increased overlap. Darker patches on the diagonal represent modules. Lipid module assignments are displayed along the left and on top. **(C)** Histogram visualizes the distribution of the number and class types of lipid features in each module. Full list of lipid feature assignment can be found in Supplementary Table S2.

**Fig. 3:**
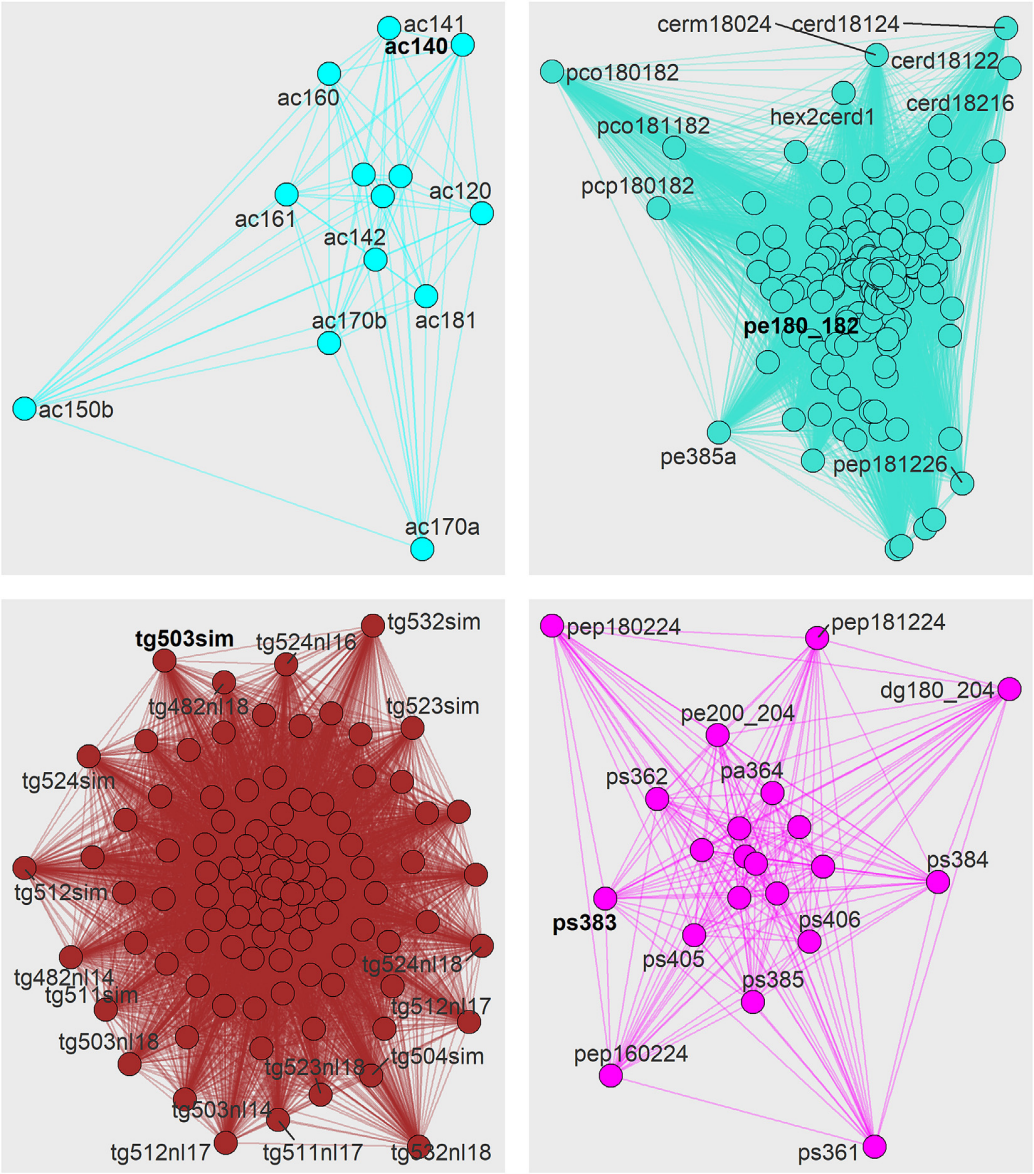
**Visualization of the Cyan, Turquoise, Brown and Magenta networks constructed from the Topological Overlay Matrix.** The nodes represent individual lipid features, node and edge colour denotes module membership. The hub lipid is denoted by bold text.

To identify networks of lipids, WGCNA was performed on the cord blood lipid concentration values (the inputs) using a soft threshold of 9, resulting in a scale-free topology of R^2^ = 0.81. A total output of 16 colour-coded modules was generated. The Grey module comprised unclustered lipids (7% of all lipid features), and thus was excluded from further analysis. The dendogram and the number of lipid features belonging to each module per lipid class is shown in Fig. 2 and a network diagram Fig. 3. The lipid features that comprise the lipid modules can be found in Supplementary Table S2.

#### The prospective association between the cord blood lipid profile and subsequent ADHD and ASD symptoms at 2 years of age

**Fig. 4:**
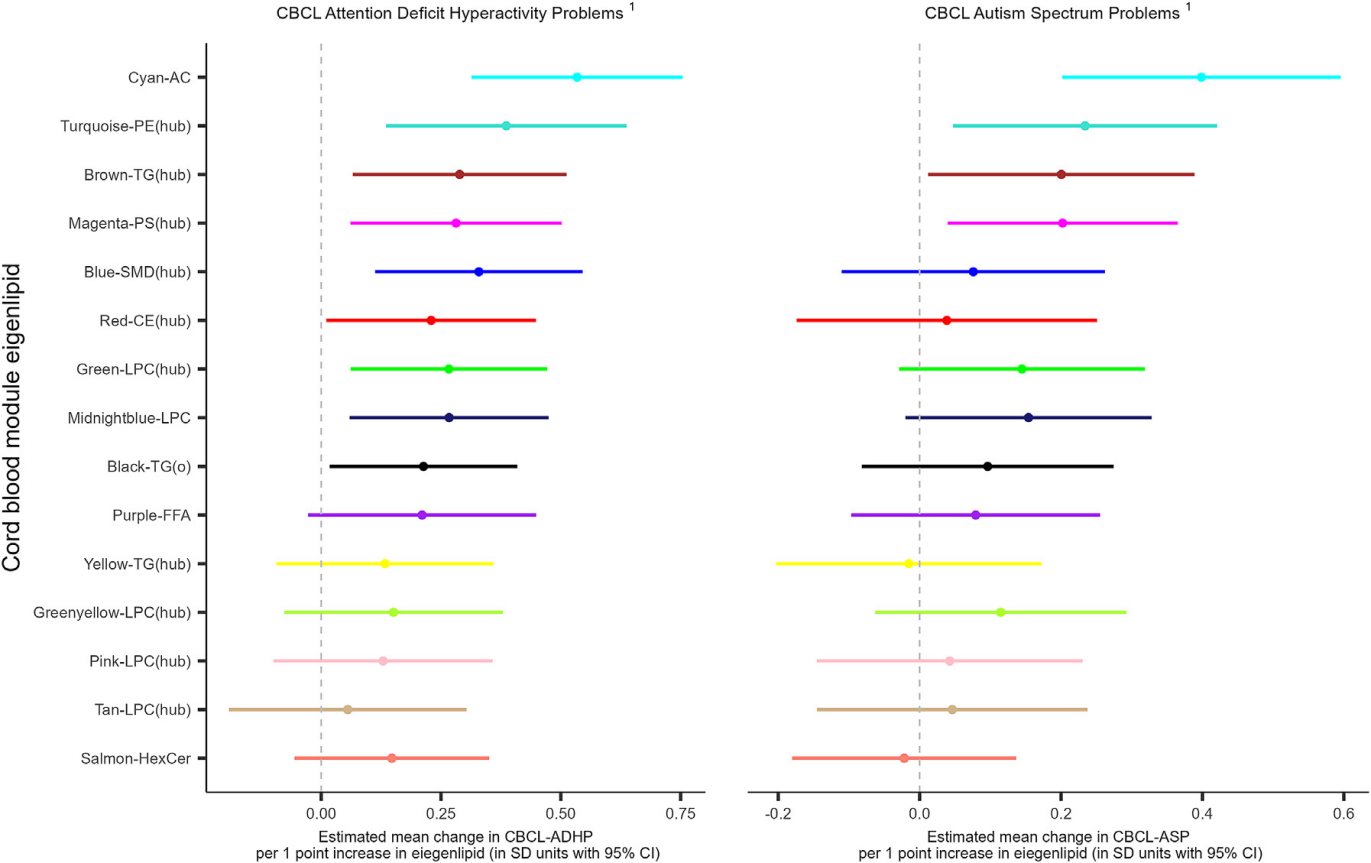
**The association between the cord blood lipid module eigenlipids and subsequent CBCL-ADHP and CBCL-ASP outcomes at 2 years.** Note: Point estimates represent adjusted mean difference in module eigenlipid (SD units with 95% CI) per unit increase in CBCL raw score with 95% CI; AC, acylcarnitine; PE, phosphatidylethanolamine; TG, triglyceride; PS, phosphatidylserine; SM, sphingomyelin; CE, cholesteryl esters; LPC, lysophosphatidylcholine; TG(o), alkyldiacylglycerol; FFA, free fatty acid; HexCer, hexosylceramide. Ranked by strength of the association across both outcomes. ^1^Model adjusted for child sex, gestational age (weeks) at birth, minutes from cord blood sample collection to storage, days cord blood sample stored, maternal blood contamination and age at CBCL. *p*-values, Q-values, R^2^, adjusted alpha (Benjamini–Hochberg critical value) and Akaike information criteria (AIC) can be found in Supplementary Table S3.

Prospective associations of the lipid modules and CBCL ADHP & ASP symptoms were assessed by fitting multivariable (minimally adjusted) linear regression models. All significant associations of lipid module eigenlipids with either ADHD or ASD symptoms at 2 years were positive (Fig. 4, Supplementary Table S3). Nine modules were significantly associated with increased ADHD symptoms at 2 years (Fig. 4, Supplementary Table S3). Four of these modules were also significantly associated with increased ASD symptoms at 2 years. After correction for multiple comparisons, eight of the module-ADHD-symptom associations and one of the module-ASD-symptom associations persisted.

We denoted the three modules ranked highest in magnitude and lowest in *p*-value with ADHD symptoms as Cyan-AC, Turquoise-PE(hub), and Blue-SMD(hub) (Fig. 4). For a 1 SD increase in Cyan-AC, Turquoise-PE(hub), and Blue-SMD(hub), the estimated mean differences in ADHD symptoms at 2 years were 0.54 points (95% CI 0.33, 0.75), 0.41 points (95% CI 0.16, 0.66), and 0.34 points (95% CI 0.13, 0.56), respectively. Six of these nine significant associations (Fig. 4, Supplementary Table S3) persisted after further confounder adjustment (Supplementary Tables S4.1 and S4.2). An additional module, Salmon-HexCer, became significantly associated after adjustment by confounder Set A (Supplementary Table S4.1).

The three modules ranked highest in significance and magnitude with ASD symptoms were denoted as Cyan-AC, Turquoise-PE(hub), and Brown-TG(hub). For a 1 SD increase in Cyan-AC, Turquoise-PE(hub), and Brown-TG(hub), the estimated mean differences in ASD symptoms at 2 years were 0.38 points (95% CI 0.19, 0.57), 0.24 points (95% CI 0.05, 0.43), and 0.21 points (95% CI 0.02, 0.39). Following adjustments for confounders using the two confounder sets (Supplementary Tables S4.1 and S4.2), one association remained significant with Set A, while only one persisted with Set B. Further details on the measurement of the confounders are described in Supplementary Table S4.3. Cyan-AC had the most robust association with both ADHD and ASD symptoms in terms of strength of association and persisting after extensive confounder adjustment. This association remained significant even after multiple comparisons testing with an FDR-adjusted alpha level of 0.05.

We conducted a post hoc statistical power analysis on the top associations for each outcome. The statistical power analysis for the Cyan-AC and ADHD symptoms model suggests that with a sample size of 555 and an R-squared value of 0.07, the study has a power of 1 to detect an effect size of 0.54, with a significance level of 5%. For the Cyan-AC and ASD symptoms model suggests that with a sample size of 555 and an R-squared value of 0.04, the study has a power of 0.99 to detect an effect size of 0.38, with a significance level of 5%.

As a sensitivity analysis, we compared the top 15% of CBCL symptom scores to the bottom 15%. A 1 SD increase in Cyan-AC was associated with being in the top 15% of symptom scores with an AOR of 1.52 (95% CI 1.20, 1.93), *p* < 0.0001 (Supplementary Table S5). This dichotomised approach supports the findings of analyses in which the continuous measure was used.

We further explored the associations between the concentrations of individual lipid features and the outcomes. Out of the 776 lipid features examined, 375 were significantly associated with ADHD symptoms, and 38 with ASD symptoms after applying an FDR correction (Supplementary Table S6.1). Additionally, from a total of 39 lipid classes, 23 showed associations with ADHD symptoms and 3 with ASD symptoms post FDR correction (Supplementary Table S6.2).

#### The prospective association between the cord blood lipid profile and subsequent ADHD and ASD symptoms at 4 years of age

We then examined three Strength and Difficulties Questionnaire outcomes at age 4 years—hyperactivity, peer problems, and prosocial behaviour. Only Cyan-AC significantly associated with all three outcomes (Supplementary Table S7). For a 1 SD increase in Cyan-AC, there was an estimated mean difference of 0.33 points (95% CI 0.14, 0.52) in hyperactivity, 0.12 points (95% CI 0.01, 0.25) in peer problems, and −0.17 points (95% CI −0.25, −0.01) in prosocial behaviour.

### Mediation analysis

As in past work,^10^^,^^12^^,^^49^ we conducted a mediation analysis to explore whether environmental factors preceded the molecular eigenlipid-cognition associations. We individually assessed each combination of environmental/metabolic factor, lipid module, and neurodevelopmental outcome (CBCL-ADHP and CBCL-ASP) in separate models.

#### Acylcarnitines mediate the relationship between low household income and ADHD symptoms

In a mediation analysis, the indirect effect estimate indicated that higher cord blood Cyan-AC partially mediated the association between lower household income during pregnancy and higher ADHD symptoms at age 2 years. The estimated proportion of the total effect mediated was 16% (Table 2).

**Table 2:** Mediation by the lipid module eigenlipids of the relationships between early life factors and CBCL-ADHP.

Early life factor (exposure vs. reference)	Lipid module (a)^a^		CBCL-ADHP (c)^b^		Mediation analysis				
			Total effect		Direct effect (c’)		Indirect effect (ab)		Proportion mediated
	*β* (95% CI)	*p*-value	*β* (95% CI)	*p*-value	*β* (95% CI)	*p*-value	*β* (95% CI)	*p*-value	
Cyan-AC									
Household income (decreasing)^c^	**0.11 (0.03, 0.20)**	**0.01**	**0.38 (0.15, 0.61)**	**0.001**	**0.32 (0.1, 0.54)**	**0.004**	**0.06 (0.01, 0.1)**	**0.02**	0.16
Prenatal maternal smoking (any vs. none)^d^	0.09 (−0.16, 0.34)	0.46	**0.81 (0.13, 1.48)**	**0.02**	**0.76 (0.08, 1.44)**	**0.029**	0.05 (−0.08, 0.18)	0.47	0.06
Vaginal birth type (assisted vs. unassisted)^e^	**0.29 (0.07, 0.50)**	**0.01**	0.14 (−0.38, 0.66)	0.6	−0.01 (−0.53, 0.52)	0.98	**0.14 (0.01, 0.28)**	**0.04**	–
Caesarean type (emergency vs. planned)	**0.73 (0.44, 1.01)**	**<0.0001**	**0.76 (0.02, 1.5)**	**0.04**	0.43 (−0.44, 1.3)	0.33	0.33 (−0.03, 0.69)	0.08	0.43
Infant Apgar score at 5 min (per unit increase)	**0.15 (0.04, 0.25)**	**0.01**	**0.43 (0.16, 0.7)**	**0.0016**	**0.36 (0.1, 0.62)**	**0.007**	**0.07 (0.01, 0.13)**	**0.02**	0.16
Turquoise-PE(hub)									
Household income (decreasing)^c^	**0.13 (0.06, 0.20)**	**0.0006**	**0.38 (0.15, 0.61)**	**0.001**	**0.34 (0.11, 0.56)**	**0.003**	0.04 (0, 0.09)	0.06	0.11
Vaginal birth type (assisted vs. unassisted)^e^	0.15 (−0.04, 0.34)	0.12	0.14 (−0.38, 0.66)	0.6	0.07 (−0.46, 0.59)	0.81	0.07 (−0.04, 0.18)	0.2	0.5
Caesarean type (emergency vs. planned)	**0.61 (0.31, 0.90)**	**<0.0001**	**0.76 (0.02, 1.5)**	**0.045**	0.68 (−0.1, 1.47)	0.089	0.08 (−0.2, 0.35)	0.58	0.11
Brown-TG(hub)									
Household income (decreasing)^c^	**0.09 (0.01, 0.17)**	**0.03**	**0.38 (0.15, 0.61)**	**0.001**	**0.35 (0.13, 0.58)**	**0.002**	0.03 (−0.01, 0.06)	0.11	0.08
Prenatal maternal smoking (any vs. none)^d^	0.09 (−0.17, 0.35)	0.51	**0.81 (0.13, 1.48)**	**0.019**	**0.78 (0.1, 1.46)**	**0.02**	0.03 (−0.06, 0.11)	0.54	0.04
Vaginal birth type (assisted vs. unassisted)^e^	**0.42 (0.24, 0.60)**	**<0.0001**	**0.43 (0.16, 0.7)**	**0.0016**	**0.4 (0.12, 0.68)**	**0.01**	0.04 (−0.01, 0.08)	0.12	0.09
Caesarean type (emergency vs. planned)	**0.93 (0.61, 1.25)**	**<0.0001**	**0.76 (0.02, 1.5)**	**0.045**	0.74 (−0.03, 1.52)	0.06	0.02 (−0.39, 0.43)	0.94	0.03
Magenta-PS(hub)									
Household income (decreasing)^c^	**0.14 (0.06, 0.23)**	**0.0009**	**0.38 (0.15, 0.61)**	**0.001**	**0.35 (0.12, 0.57)**	**0.003**	0.03 (−0.01, 0.07)	0.09	0.08
Prenatal maternal smoking (any vs. none)^d^	0.24 (0.00, 0.48)	0.05	**0.81 (0.13, 1.48)**	**0.019**	**0.74 (0.07, 1.41)**	**0.031**	0.07 (−0.02, 0.16)	0.13	0.09
Blue-SMD(hub)									
Household income (decreasing)^c^	**0.1 (0.01, 0.18)**	**0.02**	**0.38 (0.15, 0.61)**	**0.001**	**0.35 (0.13, 0.57)**	**0.002**	0.03 (0, 0.06)	0.10	0.08

The indirect effect (ab) is the amount provided by the module eigenlipid. Bold indicates statistical significance.

Proportion mediated was calculated when the direct effect and indirect effect where in the same direction.

a Model adjusted for child sex, gestational age (weeks) at birth, minutes from cord blood sample collection to storage, days cord blood sample stored and maternal blood contamination.

b Model additionally adjusted for child's age at CBCL.

c Mean household income during pregnancy and 1st year of child's life Standardized to have a mean of 0 and a standard deviation of 1.

d Any maternal smoking during preconception or pregnancy.

e Assisted vaginal birth includes forceps and vacuum assisted.

#### Additional assessment of the metabolomic markers NOPMS and GlycA at either 28 weeks or birth as antecedents of the cord blood lipid profile

Within BIS, we previously reported that a measure of prenatal non-oxidative pyruvate metabolism positively associates with ASD symptoms at age 2 years^12^ and that birth GlycA positively associates with emotional and behavioural problems at 2 years.^49^ In addition, lipid profiles are greatly influenced by metabolism and by inflammation.^47^

Prior to mediation analysis, we investigated how non-oxidative pyruvate metabolism score (NOPMS) and GlycA at the maternal and birth timepoints each associated with ADHD and ASD symptoms at age 2 years. Maternal NOPMS, birth NOPMS, and birth GlycA were associated with ADHD symptoms, but maternal GlycA was not (Table 3). Maternal NOPMS and birth GlycA were associated with ASD symptoms, but maternal GlycA and birth NOPMS were not (Table 5). Associations of these metabolite measures with the lipid modules can also be found in Tables 3 and 5.

**Table 3:** Mediation by the lipid module eigenlipids of the relationships between metabolic early life factors and CBCL-ADHP.

Metabolic early life factor	Lipid module (a)^a^		CBCL-ADHP (c)^b^		Mediation analysis				
			Total effect		Direct effect (c’)		Indirect effect (ab)		Proportion mediated
	*β* (95% CI)	*p*-value	*β* (95% CI)	*p*-value	*β* (95% CI)	*p*-value	*β* (95% CI)	*p*-value	
Cyan-AC									
Maternal NOPMS (PC1)^c^	0.08 (0.03, 0.14)	**0.004**	−0.01 (−0.16, 0.13)	0.84	−0.06 (−0.2, 0.09)	0.42	**0.04 (0.01, 0.08)**	**0.01**	–
NOPMS at birth (PC1)	0.39 (0.34, 0.44)	**<0.0001**	**0.19 (0.05, 0.32)**	**0.007**	−0.04 (−0.2, 0.12)	0.62	**0.23 (0.12, 0.34)**	**<0.0001**	–
GlycA at birth (mmol/L)	3.08 (2.43, 3.73)	**<0.0001**	**2.49 (1.02, 3.97)**	**0.0009**	1.08 (−0.63, 2.79)	0.22	**1.41 (0.6, 2.22)**	**0.0006**	0.57
Turquoise-PE(hub)									
NOPMS at birth (PC1)	3.73 (3.14, 4.31)	**<0.0001**	**0.19 (0.05, 0.32)**	**0.007**	0.12 (−0.02, 0.26)	0.10	**0.07 (0.02, 0.12)**	**0.008**	0.37
GlycA at birth (mmol/L)	0.17 (0.12, 0.23)	**<0.0001**	**2.49 (1.02, 3.97)**	**0.0009**	1.67 (−0.03, 3.38)	0.06	0.82 (−0.29, 1.93)	0.15	0.33
Brown-TG(hub)									
Maternal NOPMS (PC1)^c^	0.68 (0.24, 1.11)	**0.003**	0.63 (−0.47, 1.73)	0.26	0.44 (−0.65, 1.54)	**0.43**	0.19 (−0.01, 0.39)	0.07	0.3
GlycA at birth (mmol/L)	3.7 (3.18, 4.23)	**<0.0001**	**2.49 (1.02, 3.97)**	**0.001**	**2.26 (0.44, 4.08)**	**0.02**	0.2 (−0.21, 0.61)	0.33	0.09
Magenta-PS(hub)									
Maternal NOPMS (PC1)^c^	0.04 (−0.02, 0.11)	0.20	−0.01 (−0.16, 0.13)	0.84	−0.03 (−0.17, 0.12)	0.72	0.01 (−0.01, 0.03)	0.29	–
NOPMS at birth (PC1)	0.27 (0.22, 0.32)	**<0.0001**	**0.19 (0.05, 0.32)**	**0.007**	0.12 (−0.03, 0.26)	0.12	**0.07 (0.01, 0.14)**	**0.03**	0.37
GlycA at birth (mmol/L)	2.91 (2.42, 3.39)	**<0.0001**	**2.49 (1.02, 3.97)**	**0.001**	**2.1 (0.46, 3.74)**	**0.01**	0.39 (−0.27, 1.06)	0.25	0.16
Blue-SMD(hub)									
NOPMS at birth (PC1)	0.11 (0.06, 0.16)	**<0.0001**	**0.19 (0.05, 0.32)**	**0.007**	**0.15 (0.01, 0.29)**	**0.04**	**0.04 (0.01, 0.07)**	**0.02**	0.21
GlycA at birth (mmol/L)	2.41 (1.81, 3.01)	**<0.0001**	**2.49 (1.02, 3.97)**	**0.001**	**1.99 (0.41, 3.58)**	**0.01**	0.5 (−0.09, 1.1)	0.10	0.2
Green-LPC(hub)									
NOPMS at birth (PC1)	0.1 (0.05, 0.16)	**0.0001**	**0.19 (0.05, 0.32)**	**0.007**	**0.17 (0.03, 0.3)**	**0.02**	0.02 (0, 0.05)	0.11	0.11
GlycA at birth (mmol/L)	1.23 (0.46, 1.99)	**0.002**	**2.49 (1.02, 3.97)**	**0.001**	**2.26 (0.75, 3.77)**	**0.003**	0.24 (−0.06, 0.53)	0.11	0.1
Midnightblue-LPC(hub)									
NOPMS at birth (PC1)	0.11 (0.05, 0.16)	**0.0001**	**0.19 (0.05, 0.32)**	**0.007**	**0.17 (0.03, 0.31)**	**0.02**	0.02 (−0.01, 0.05)	0.16	0.11
GlycA at birth (mmol/L)	1.15 (0.45, 1.85)	**0.001**	**2.49 (1.02, 3.97)**	**0.0009**	**2.29 (0.76, 3.81)**	**0.003**	0.21 (−0.07, 0.48)	0.14	0.08
Black (TG(o))									
NOPMS at birth (PC1)	0.11 (0.04, 0.17)	**0.0008**	**0.19 (0.05, 0.32)**	**0.007**	**0.17 (0.03, 0.3)**	**0.02**	0.02 (−0.01, 0.05)	0.13	0.11
GlycA at birth (mmol/L)	2.66 (1.89, 3.44)	**<0.0001**	**2.49 (1.02, 3.97)**	**0.001**	**2.22 (0.7, 3.75)**	**0.004**	0.27 (−0.26, 0.79)	0.31	0.11

The indirect effect (ab) is the amount provided by the module eigenlipid. Bold indicates statistical significance.

Proportion mediated was calculated when the direct effect and indirect effect where in the same direction.

Note: NOPMS, non-oxidative pyruvate metabolism score, PC1 of pyruvate, lactate, acetate and alanine^12^ (all loadings were in the positive direction and therefore higher PC1 values were interpreted as increased pathway activity).

a Model adjusted for child sex, gestational age (weeks) at birth, minutes from cord blood sample collection to storage, days cord blood sample stored and maternal blood contamination.

b Model adjusted for child sex, gestational age (weeks) at birth, minutes from cord blood sample collection to storage, days cord blood sample stored, maternal blood contamination and child's age at CBCL.

c Model additionally adjusted by gestational age (weeks) at maternal blood collection.

**Table 4:** Mediation by the lipid module eigenlipids of the relationships between early life factors and CBCL-ASP.

Early life factor (exposure vs. reference)	Lipid module (a)^a^		CBCL-ASP (c)^b^		Mediation analysis				
			Total effect		Direct effect (c’)		Indirect effect (ab)		Proportion mediated
	*β* (95% CI)	*p*-value	*β* (95% CI)	*p*-value	*β* (95% CI)	*p*-value	*β* (95% CI)	*p*-value	
Cyan-AC									
Household income (decreasing)^c^	**0.11 (0.03, 0.20)**	**0.01**	**0.32 (0.14, 0.51)**	**0.001**	**0.28 (0.1, 0.46)**	**0.002**	**0.04 (0, 0.07)**	**0.03**	0.12
Prenatal maternal smoking (any vs. none)^d^	0.09 (−0.16, 0.34)	0.46	**0.55 (0.03, 1.06)**	**0.04**	**0.51 (0.01, 1.01)**	**0.046**	0.04 (−0.06, 0.13)	0.48	0.07
Vaginal birth type (assisted vs. unassisted)^e^	**0.29 (0.07, 0.50)**	**0.01**	**0.46 (0, 0.91)**	**0.048**	0.36 (−0.09, 0.81)	0.12	0.1 (0, 0.19)	0.05	0.22
Caesarean type (emergency vs. planned)	**0.73 (0.44, 1.01)**	**<0.0001**	**0.86 (0.31, 1.41)**	**0.002**	0.67 (0.03, 1.3)	**0.04**	0.19 (−0.14, 0.53)	0.25	0.22
Infant Apgar score at 5 min (per unit increase)	**0.15 (0.04, 0.25)**	**0.01**	0.01 (−0.16, 0.18)	0.910	−0.05 (−0.22, 0.12)	0.58	**0.06 (0.01, 0.11)**	**0.03**	–
Turquoise-PE(hub)									
Household income (decreasing)^c^	**0.13 (0.06, 0.20)**	**0.0006**	**0.32 (0.14, 0.51)**	**0.001**	**0.3 (0.11, 0.48)**	**0.002**	0.03 (0, 0.06)	0.08	0.09
Vaginal birth type (assisted vs. unassisted)^e^	0.15 (−0.04, 0.34)	0.12	**0.46 (0, 0.91)**	**0.05**	0.42 (−0.04, 0.87)	0.07	0.04 (−0.03, 0.11)	0.27	0.09
Caesarean type (emergency vs. planned)	**0.61 (0.31, 0.90)**	**<0.0001**	**0.86 (0.31, 1.41)**	**0.002**	**0.86 (0.26, 1.46)**	**0.01**	0 (−0.2, 0.2)	1	0
Brown-TG(hub)									
Household income (decreasing)^c^	**0.09 (0.01, 0.17)**	**0.03**	**0.32 (0.14, 0.51)**	**0.001**	**0.3 (0.12, 0.49)**	**0.001**	0.02 (−0.01, 0.04)	0.15	0.06
Prenatal maternal smoking (any vs. none)^d^	0.09 (−0.17, 0.35)	0.51	**0.55 (0.03, 1.06)**	**0.04**	**0.53 (0.02, 1.04)**	**0.04**	0.02 (−0.04, 0.08)	0.59	0.04
Vaginal birth type (assisted vs. unassisted)^e^	**0.42 (0.24, 0.60)**	**<0.0001**	**0.46 (0, 0.91)**	**0.05**	0.33 (−0.13, 0.8)	0.16	0.12 (0, 0.25)	0.06	0.27
Caesarean type (emergency vs. planned)	**0.93 (0.61, 1.25)**	**<0.0001**	**0.86 (0.31, 1.41)**	**0.00**	**1.05 (0.41, 1.7)**	**0.001**	−0.19 (−0.5, 0.11)	0.22	0.0014
Infant Apgar score at 5 min (per unit increase)	**0.13 (0.04, 0.22)**	**0.004**	0.01 (−0.16, 0.18)	0.91	−0.02 (−0.19, 0.16)	0.84	0.03 (−0.01, 0.06)	0.12	–
Magenta-PS(hub)									
Household income (decreasing)^c^	**0.14 (0.06, 0.23)**	**0.0009**	**0.32 (0.14, 0.51)**	**0.001**	**0.3 (0.11, 0.49)**	**0.00**	0.02 (0, 0.05)	0.10	0.06
Prenatal maternal smoking (any vs. none)^d^	**0.24 (0.00, 0.48)**	**0.05**	**0.55 (0.03, 1.06)**	**0.04**	0.5 (−0.01, 1.01)	0.05	0.05 (−0.02, 0.11)	0.18	0.09

The indirect effect (ab) is the amount provided by the module eigenlipid. Bold indicates statistical significance.

Proportion mediated was calculated when the direct effect and indirect effect where in the same direction.

a Model adjusted for child sex, gestational age (weeks) at birth, minutes from cord blood sample collection to storage, days cord blood sample stored and maternal blood contamination.

b Model additionally adjusted for child's age at CBCL.

c Mean household income during pregnancy and 1st year of child's life.

d Any maternal smoking during preconception or pregnancy.

e Assisted vaginal birth includes forceps and vacuum assisted.

**Table 5:** Mediation by the lipid module eigenlipids of the relationships between metabolic early life factors and CBCL-ASP.

Metabolic early life factor	Lipid module (a)^a^		CBCL-ASD (c)^b^		Mediation analysis				
					Direct effect (c’)		Indirect effect (ab)		Proportion mediated
	*β* (95% CI)	*p*-value	*β* (95% CI)	*p*-value	*β* (95% CI)	*p*-value	*β* (95% CI)	*p*-value	
Cyan (AC)									
Maternal NOPMS (PC1)^c^	0.08 (0.03, 0.14)	0.004	**0.17 (0.02, 0.32)**	**0.03**	0.14 (−0.01, 0.29)	0.07	**0.03 (0, 0.06)**	**0.02**	0.18
NOPMS at birth (PC1)%	0.39 (0.34, 0.44)	<0.0001	0.11 (0, 0.22)	0.06	−0.04 (−0.19, 0.1)	0.58	**0.15 (0.05, 0.25)**	**0.002**	1.36
GlycA at birth	3.08 (2.43, 3.73)	<0.0001	**1.26 (0.36, 2.16)**	**0.006**	0.08 (−0.98, 1.15)	0.88	**1.18 (0.44, 1.91)**	**0.002**	0.94
Turquoise (PE)									
NOPMS at birth (PC1)	3.73 (3.14, 4.31)	<0.0001	**1.26 (0.36, 2.16)**	**0.006**	0.63 (−0.69, 1.96)	0.35	0.62 (−0.36, 1.61)	0.21	0.5
GlycA at birth	0.17 (0.12, 0.23)	<0.0001	0.11 (0, 0.22)	0.06	0.08 (−0.04, 0.19)	0.21	0.03 (0, 0.07)	0.07	0.27
Brown (TG)									
Maternal NOPMS (PC1)^c^	0.68 (0.24, 1.11)	0.003	**0.82 (−0.12, 1.77)**	**0.09**	0.71 (−0.21, 1.63)	0.13	0.12 (−0.03, 0.26)	0.11	0.14
NOPMS at birth (PC1)	3.7 (3.18, 4.23)	<0.0001	**1.26 (0.36, 2.16)**	**0.006**	0.8 (−0.45, 2.06)	0.21	0.46 (−0.47, 1.38)	0.33	0.37
GlycA at birth	0.21 (0.15, 0.27)	<0.0001	0.11 (0, 0.22)	0.06	0.08 (−0.05, 0.2)	0.25	0.03 (−0.01, 0.08)	0.14	0.27
Magenta (PS)									
NOPMS at birth (PC1)	0.27 (0.22, 0.32)	>0.0001	0.11 (0, 0.22)	0.06	0.07 (−0.05, 0.19)	0.24	0.04 (−0.01, 0.09)	0.14	0.36
GlycA at birth	2.91 (2.42, 3.39)	>0.0001	**1.26 (0.36, 2.16)**	**0.01**	0.81 (−0.21, 1.82)	0.12	0.45 (−0.09, 0.99)	0.10	0.36

The indirect effect (ab) is the amount provided by the module eigenlipid. Bold indicates statistical significance.

Proportion mediated was calculated when the direct effect and indirect effect where in the same direction.

Note: NOPMS, non-oxidative pyruvate metabolism score, PC1 of pyruvate, lactate, acetate and alanine^12^ (all loadings were in the positive direction and therefore higher PC1 values were interpreted as increased pathway activity).

a Model adjusted for child sex, gestational age (weeks) at birth, minutes from cord blood sample collection to storage, days cord blood sample stored and maternal blood contamination.

b Model adjusted for child sex, gestational age (weeks) at birth, minutes from cord blood sample collection to storage, days cord blood sample stored, maternal blood contamination and child's age at CBCL.

c Model additionally adjusted by gestational age (weeks) at maternal blood collection.

#### Cord blood lipid profile mediates the relationship between the birth factors of low Apgar score, birth GlycA, birth non-oxidative pyruvate metabolism, and ADHD symptoms

The indirect effect estimate indicated that higher cord blood Cyan-AC partially mediated the association between lower Apgar score of the child 5 min after birth and higher ADHD symptoms at age 2. The estimated proportion of the total effect mediated was 16% (Table 2).

Overall, there was strong evidence of mediation by Cyan-AC of the association between birth NOPMS and ADHD symptoms, as well as partial mediation (estimated proportion 57%) by Cyan-AC of birth GlycA and ADHD symptoms (Table 3). In addition, there was partial mediation by Turquoise-PE(hub), Magenta-PS(hub), and Blue-SMD(hub) of the birth NOPMS and ADHD-symptom association, with the estimated proportion of the mediated effects ranging from 21% to 37% (Table 3).

#### Acylcarnitines mediate the relationship between household income, maternal non-oxidative pyruvate metabolism, birth GlycA, and ASD symptoms

The indirect effect estimate indicated that higher cord blood Cyan-AC partially mediated the association between lower household income in pregnancy and higher ASD symptoms at age 2. The estimated proportion of the total effect mediated was 12% (Table 4). In addition, there was partial mediation of the association between maternal NOPMS and ASD symptoms by Cyan-AC, with the proportion mediated estimated to be 18% (Table 5). There was also evidence suggesting almost full mediation (estimated proportion 94%) of the effect of birth GlycA on ASD symptoms by Cyan-AC (Table 5).

### Additional analyses

Given that Cyan-AC, GlycA, and NOPMS were associated with both ADHD and ASD symptoms, we conducted further analyses to assess their relative importance.

As birth NOPMS/GlycA and the cord blood lipid modules are cross-sectional, and thus there is no clear temporal relationship for mediation analysis, we performed additional analyses whereby the position of the exposure and mediator were reversed in our mediation analyses (Supplementary Tables S8.1 and S8.2). Neither NOPMS nor birth GlycA mediated the relationship between Cyan-AC and ADHD symptoms, but we did observe that birth GlycA mediated associations between many of the other module eigenlipids and ADHD symptoms at 2 years, which are listed in Supplementary Table S8.1. We did not observe any mediation by maternal or birth NOPMS or maternal or birth GlycA of any of the lipid modules to ASD symptoms at 2 years (Supplementary Table S8.2).

To determine the most critical period of the lipid module’s effects, we then used the cord blood lipid modules and calculated eigenlipids for the top four modules for the maternal (28 weeks gestation), 6-month, 1-year, and 4-year blood sample timepoints. There were no correlations between the top four lipid modules (Cyan-AC, Brown-TG(hub), Turquoise-PE(hub), and Magenta-PS(hub)) and ADHD or ASD symptoms (age 2) at these time points, except for a positive correlation between Cyan-AC and ASD symptoms (age 2) at 1 year of age lipidome (data not shown).

Further, Cyan-AC appears to have exhibited a dominant role compared to the other lipid modules. In a mutually adjusted model with all lipid modules, Cyan-AC remained significant in both the ADHD-symptom model (0.53 CBCL-ADHP points per 1 SD change in eigenlipid (95% CI 0.25, 0.82), *p* < 0.00001) and ASD-symptom model (0.40 CBCL-ADHP points per 1 SD change in eigenlipid (95% CI 0.14, 0.65), *p* < 0.0001) (Supplementary Table S9).

To account for the influence of perinatal factors on the minimally adjusted associations between the lipid modules and the outcomes at 2 years, we restricted the sample to healthy natural uncomplicated vaginal births (unassisted vaginal births, no resuscitation, Apgar >8 at 5 min, labour time shorter than 18 h). This increased the magnitude of association between both Cyan-AC and ADHD symptoms (0.60 points per 1 SD change in eigenlipid (95% CI 0.27, 0.95)) and Cyan-AC and ASD symptoms (0.50 points per 1 SD change in eigenlipid (95% CI 0.24, 0.76)). We conclude that the positive cord blood Cyan-AC associations with the symptoms of both disorders were unlikely to be driven by birth process events.

Since metabolites vary by time of day, and in relation to sleeping and feeding patterns,^62^ we further adjusted lipid-module-ADHD and lipid-module-ASD models by a sin and cosine weighted time of day of blood sample collection. This did not materially change the findings, and therefore this factor was not routinely adjusted for.

We used inverse probability weighting to adjust for the potential influence of sampling bias between non-responders at initial recruitment and attrition by age 2 years. When accounting for selection bias from non-participation, the relationship of Cyan-AC to ADHD symptoms (IPW: β = 0.62, 95% CI 0.38, 0.85), *p* < 0.00001; original: β = 0.54, 95% CI 0.33,0.75, *p* < 0.0001) and ASD symptoms (IPW: β = 0.43, 95% CI 0.19, 0.68), *p* = 0.0005; original: β = 0.38, 95% CI 0.19, 0.57, *p* < 0.00001) appeared to be originally underestimated by 15% and 14%, respectively. Thus, when accounting for potential selection bias of responders using IPW, the positive associations between Cyan-AC and both neurodevelopmental outcomes persisted.

## Discussion

In this population-derived prospective birth cohort, we found that a lipid module consisting of only acylcarnitines in cord blood was strongly positively associated with subsequent ADHD and ASD at age 2 years. Furthermore, lipid modules containing mainly phosphoglycerolipids, mainly triglycerides, and mainly phosphatidylserine and other phospholipids were also associated with ADHD and ASD; although these associations attenuated following adjustment for the cord blood acylcarnitine lipid module. In our analysis of lipid concentrations, we noted that both the total class and individual lipid features typically showed positive associations with ADHD and ASD, corroborating the WGCNA associations. In particular, as levels of both total phosphatidylethanolamine and total phosphatidylinositol increased, we observed greater symptoms of ADHD and ASD. Overall, the contribution of variation of cord blood elevated long-chain acylcarnitine module, unweighted for function, on ADHD and ASD symptoms (represented by R^2^ values) ranged between 7% and 11%. This is higher than most unweighted polygenic risk scores for ASD and ADHD traits. Studies report that variance accounted for by unweighted polygenic risk scores for either ASD or ADHD traits is often below 3%.^63^, ^64^, ^65^, ^66^ Therefore, if the perinatal acylcarnitine lipidomic profile or its determinants are causal in nature, variation in this could partly explain the onset of ASD and ADHD traits in early childhood.

To assess evidence of causality, we undertook mediation analysis using a causal framework. A metabolite is more likely to be causally associated with a neurodevelopmental outcome if an established risk factor is shown to be mediated to some extent by the putative metabolite biomarker.^10^ Here, elevated acylcarnitine lipid profile in cord blood mediated 16% and 12% of the effect of lower household income (a well-established risk factor for adverse neurodevelopment^10^^,^^12^^,^^49^^,^^67^, ^68^, ^69^, ^70^) on increased ADHD and ASD symptoms. We also observed that several lipid modules, but especially acylcarnitines, mediated the association between low household income and neurodevelopmental outcomes. Social adversity might influence lifestyle factors, such as diet during pregnancy, or increased exposure to environmental pollutants or infections. These factors can potentially impact the lipidome by inducing a pro-inflammatory state. Further, the effect of a lower Apgar score at 5 min, which has previously been established as a risk factor for ADHD and ASD,^71^^,^^72^ on both ADHD and ASD symptoms was mediated through elevated acylcarnitine levels.

The Cyan-AC module comprised acylcarnitines ranging from AC12:0 to AC18:3, with the exception of AC13:0. Moreover, the total concentration of cord blood acylcarnitines (from AC12:0 to AC18:3) was linked with both outcomes. This supports the functional relevance of the Cyan-AC module, leading us to conclude that it is the long-chain acylcarnitines that associate with the behavioural outcomes. Excess long-chain acylcarnitines can be generated when the influx of fatty acids surpasses the mitochondria's processing capacity, which then results in a misalignment between β-oxidation and the tricarboxylic acid cycle (TCA cycle).^73^^,^^74^ Several cross-sectional studies have also found that ASD cases aged 2–11 years have higher circulating levels of various long-chain acylcarnitines. There is mounting evidence that mitochondrial dysfunction may be a key factor in autism pathogenesis,^75^^,^^76^ including our previous work showing increases in maternal plasma lactate and pyruvate are associated with ASD symptoms.^12^ A single prospective study,^36^ reported elevated long-chain acylcarnitines, measured by the acylcarnitine C16/C2 ratio, in the neonatal heel prick screening test, was associated with subsequent ASD diagnosis at 3–5 years old, but only among preterm babies (<37 weeks), and C18 was elevated in boys but not in girls. All but one of the previous studies were cross-sectional and most considered each species of acylcarnitines independently rather than as a ratio or network, unlike this prospective correlation network analysis.

Our findings are consistent with previous work indicating prenatal and perinatal risk factors for adverse neurodevelopment are acting through metabolic disruption, including lipid metabolism.^10^ We have previously reported that an increase in the maternal non-oxidative pyruvate metabolism score (NOPMS) in pregnancy is associated with subsequent ASD symptoms at age 2 years.^12^ While maternal acylcarnitine levels were not associated with either the child’s ADHD or ASD symptoms, they were correlated with maternal NOPMS. As the newborn’s acylcarnitine levels are higher compared to maternal levels during pregnancy (28-week gestation),^46^ it is possible the effect size is too small to be detected in our cohort. Here, we show that an estimated 18% of the effect of this increase in maternal non-oxidative energy production on ASD symptoms is mediated through elevated acylcarnitines in the cord blood, or factors linked closely to this lipid profile. A similar pattern is observed for NOPMS in the cord blood (rather than the prenatal period) and ADHD. However, in addition to Cyan-AC, Turquoise-PE(hub), Magenta-PS(hub) and Blue-SMD(hub) also mediate the association between NOPMS in the cord blood and ADHD symptoms. Another key finding was that the acylcarnitine increase linked to later ADHD and ASD was strongly associated with a pro-inflammatory state, as measured by the marker for cumulative inflammation, GlycA and we provide further information on temporality. Here, the findings indicate the majority of the adverse effect of inflammation on ADHD and ASD occurs through the acylcarnitine-linked pathway and that the acylcarnitine increase is a likely consequence, not a cause of the inflammation because there was no mediation of the association of cord blood acylcarnitines and the neurodevelopmental outcomes by elevated cord blood GlycA. This indicates that the elevated acylcarnitines, or related pathways, could be a consequence of pro-inflammatory processes that adversely affect neuronal function and/or structure in early life.

One possibility is that the cord blood acylcarnitines directly affect brain function or structure. Past work investigating acylcarnitines and ASD presents a second possibility also, that is, elevated acylcarnitines are a consequence of an intracellular pathological process such as mitochondrial and fatty acid metabolism dysfunction. This is because high blood acylcarnitines are present as a consequence of mitochondrial dysfunction.^77^ Both mitochondrial dysfunction and impaired fatty acid metabolism could also lead to free carnitine deficiency, which has been linked to ASD.^78^ Further, inflammatory states can cause mitochondrial dysfunction^79^ and vice versa.^80^ Some acylcarnitine species are also a by-product of incomplete fatty acid oxidation of long-chain fatty acids,^81^ which occurs during inflammatory states.^38^ Thus, elevated blood acylcarnitines likely indicate mitochondrial and/or metabolic dysfunction.

The major strength of this study is the prospective longitudinal study design with comprehensive early life data, which includes serial lipidomic and metabolic measures and serial ADHD and ASD symptoms before school age. We identified several lines of evidence for causation. There were consistent findings across both the 2-year CBCL and the 4-year SDQ questionnaire subscales that acylcarnitines were associated with ADHD and ASD symptoms. The prospective temporality of cord blood lipids and an outcome in children are consistent with a causal pathway. There is also biological plausibility that intracellular pathological processes resulting in elevated serum acylcarnitines as its biomarker may lead to adverse neurodevelopment. Lastly, our findings are consistent with past work. This allowed an in-depth assessment of whether the cord blood lipidomics and neurodevelopmental outcomes were potentially causally associated. We also employed a counterfactual mediation framework and found that previously identified prenatal and perinatal risk factors for neurodevelopmental diagnoses were operating partly through elevated acylcarnitines or factors closely related to them. We also considered non-causal explanations, including confounding and selection bias. The comprehensive data assembled in BIS allowed for extensive evaluation of confounding, and the main findings persisted after multiple sets of confounder adjustments. While unmeasured confounding factors cannot be completely removed from an observational study, it is much less likely in our cohort due to our extensive data collection. This is important within the counterfactual mediation framework, which assumes no unmeasured confounders. Selection bias is less likely to affect our cohort, as demonstrated by the current and our other studies^10^^,^^12^ in which results did not materially change after the application of inverse probability weighting. To examine how certain prenatal factors affect acylcarnitines independently of factors leading to perinatal hypoxia,^49^ we restricted our analysis sample to cases with uneventful births. We found that the effect persisted, indicating that the elevated acylcarnitines were not solely driven by perinatal hypoxia.

There are several limitations. The clinical relevance of our measures of ADHD and autism symptoms, as compared to validated clinical diagnoses, is uncertain. While we assessed if the lipid modules mediated some known risk factors of ADHD and ASD, our cohort was not large enough for sufficient power to assess relatively uncommon risk factors for ADHD and ASD, which may also influence the lipid profile (for example, preeclampsia^82^, ^83^, ^84^ and gestational diabetes,^85^ conditions also associated with elevated acylcarnitines). Blood samples were collected at different times of the day across participants, and some metabolite levels are regulated by the circadian clock (especially long-chain acylcarnitines).^62^^,^^86^ However, we found after additionally adjusting for time of day of sample collection, the lipid module-outcome estimates were only nominally changed.

The use of WGCNA has inherent strengths and limitations. Lipid networks produced using WGCNA are contingent upon the dataset. This implies that co-expression influencing elements, even those unrelated to the outcome, can determine the resultant networks. An advantage, however, is that, unlike pathway analysis methods, WGCNA doesn't hinge on pre-existing knowledge of pathways. We supplemented our WGCNA analysis with regression analyses of individual lipid features and the outcomes. The results confirmed that the lipid classes related to the outcomes and the direction of the associations were consistent with those observed for the WGCNA eigenlipids.

While our WGCNA-based clustering provided valuable insights into broader lipid networks, the networks formulated in cord blood were more influenced by lipid class metabolism than by fatty acid metabolism. Hence, we did not analyse associations of particular circulating fatty acid ratios, such as the omega-3 to omega-6 ratio, previously associated with ADHD or ASD. Furthermore, lipids may participate in multiple networks biologically, but WGCNA with its hierarchical clustering algorithm only clusters each lipid to a single network. Finally, because the construction of lipid networks via WGCNA is inherently data-driven, the resultant networks may be influenced by the initial dataset and the choice of parameters. This potential for bias underscores the need for additional research into the methodological application of WGCNA within lipidomics to ensure robust and unbiased network formation that is biologically meaningful.^87^

Though we assessed an extensive array of lipid features across numerous lipid classes, we mainly measured long-chain acylcarnitines using UHPLC-MS/MS. Future research investigating the role of acylcarnitines might also incorporate measurements of carnitine and short, medium, and very long-chain acylcarnitines. Our population cohort was in a single region in Australia which was representative of the Australian population, except for a lower proportion of families from non-English speaking backgrounds. While IPW was used to increase generalisability, findings should be replicated in other population birth cohorts. Moreover, future studies using replicated birth cohorts could facilitate the investigation of case diagnosis, which often occurs after children start school and would therefore require a longer follow-up period. Ideally, appropriate birth cohorts would have sequential measurements of metabolomics and lipidomics, as well as data on prenatal environment and lifestyle. Such studies could also incorporate measures of mitochondrial function, carnitine shuttle transport, and related metabolites to further elucidate the mechanism of ASD and ADHD in children.

In conclusion, the present study identified eight cord blood lipid modules that positively associated with ADHD symptoms at age 2 years, with one of those modules also positively associating with ASD symptoms at age 2 years, after correcting for multiple comparisons. The lipid module Cyan-AC, consisting entirely of acylcarnitines, demonstrated the strongest positive association with both ADHD and ASD symptoms at age 2. The identified lipid modules also partially mediated the impact of established risk factors, including lower household income, low Apgar scores, increased non-oxidative pyruvate metabolism in the mother during pregnancy and the infant at birth, and higher GlycA levels at birth, on the symptoms of ADHD and ASD. The major implications of our study lie in identifying new potential targets for intervention, and importantly, pinpointing a critical window for potential intervention - the later prenatal period.

## Contributors

Conceptualisation: KV, ALP, PM, SB, AM, STa, STh, WCB, MO, RS, TM, DB, FC, PV.

Methodology: KV, ALP, AM, STa, STh, SB, MO, RS, TM, DB, FC, PV.

Data collection: PM, ALP, RS, TM, DB, FC, CP, SB, PV.

Data Analysis: KV, ALP, AM, STa, STh.

Visualisation: KV, AM, AE, STh.

Funding acquisition: ALP, RS, FC, DB, PM, PV.

Writing—original draft: KV, ALP, STh, AE, MO, SB, WCB.

Writing—review & editing: All.

Verified underlying data: STh, AE, AM.

All authors read and approved the final version of the manuscript.

## Data and material availability

### Data availability statement

Barwon Infant Study (BIS) data requests are considered on scientific and ethical grounds by the BIS Steering Committee. If approved, data re provided under collaborative research agreements.

### Code availability statement

For academic and government purposes, please contact the corresponding author. All experiments and implementation details are described in sufficient detail to support replication with non-proprietary libraries.

## Declaration of generative AI and AI-assisted technologies in the writing process

During the preparation of this work the first author used ChatGPT for copyediting. After using this tool/service, the first author personally reviewed and edited the ChatGPT sourced suggestions.

## Declaration of interests

The authors declare that they have no known competing financial interests or personal relationships that could have appeared to influence the work reported in this paper.
