# Supplementary material for: Cord blood lipid correlation network profiles are associated with subsequent attention-deficit/hyperactivity disorder and autism spectrum disorder symptoms at 2 years: a prospective birth cohort study
Source: eBioMedicine. 2024 Jan 9;100:104949. doi: 10.1016/j.ebiom.2023.104949 (PMC10825361; doi:10.1016/j.ebiom.2023.104949)
Supplement: Supplementary Materials [file mmc1.docx]

**Supplementary Materials**

**Cord blood lipid correlation network profiles are associated with subsequent attention-deficit/hyperactivity disorder and autism spectrum disorder symptoms at 2 years: a prospective birth cohort study.**

MBMSc Kristina Vacy^a,b^, MBioStat Sarah Thomson^a^, Bsc Maths Archer Moore^c^, MAppStat Alex Eisner^a^, MSc (Bioinf) Sam Tanner^a^, PhD Cindy Pham^d,e^, PhD Richard Saffrey^d,e^, PhD Toby Mansell^d,e^, PhD David Burgner^d,e,f^, PhD Fiona Collier^g,h^, PhD Peter Vuillermin^g^, PhD Martin O’Hely^d,h^, PhD Wah Chin Boon^a^, PhD Satvika Burugupalli^i^, PhD Anne-Louise Ponsonby^a,d,e^# and the Barwon Infant Study Investigator Group

^a^ Florey Institute of Neuroscience and Mental Health, University of Melbourne, Parkville 3010, Australia.

^b^ Melbourne School of Population and Global Health, University of Melbourne, Parkville 3010, Australia.

^c^ Melbourne School of Mathematics and Statistics, University of Melbourne, Parkville 3010, Australia.

^d^ Murdoch Children’s Research Institute, Royal Children’s Hospital, Parkville 3010, Australia.

^e^ Department of Paediatrics, University of Melbourne, Parkville 3010, Australia.

^f^ Department of Paediatrics, Monash University, Clayton 3168, Australia.

^g^ Child Health Research Unit, Barwon Health, Geelong 3220, Australia.

^h^ School of Medicine, Deakin University, Geelong 3220, Australia.

^i^ Metabolomics Laboratory, Baker Heart and Diabetes Institute, Melbourne 3004, Australia.

Corresponding Author:

#Address correspondence to Professor Anne-Louise Ponsonby [annelouise.ponsonby@florey.edu.au](mailto:annelouise.ponsonby@florey.edu.au)

Present address: Florey Institute of Neuroscience and Mental Health, 30 Royal Parade, Parkville, VIC, Australia, 3052.

**This PDF file includes:**

Description of supplemental tables and figures

Supplementary Tables S1 to S9

Supplementary Figure S1-S2

**Description of supplemental tables and figures**

**Supplementary Table S1:** Additional adjustment factors considered in change-in-estimate analyses.

**Supplementary Fig. S1.1:** Directed Acyclic Graph (DAG) for adjustment Set A and adjustment Set B.

**Supplementary Figure S1.2:** Mediation concept diagram.

**Supplementary Table S2:** The composition of the lipid modules, ranked by kWithin.

**Supplementary Table S3**: The association between the cord blood lipid module eigenlipids and subsequent CBCL-ADHP and CBCL-ASP outcomes at 2 years, additional statistics.

**Supplementary Table S4.1:** The association between the cord blood lipid module eigenlipids and subsequent CBCL-ADHP and CBCL-ASP outcomes at 2 years (Set A confounders).

**Supplementary Table S4.2:** The association between the cord blood lipid module eigenlipids and subsequent CBCL-ADHP and CBCL-ASP outcomes at 2 years (Set B confounders).

**Supplementary Table S4.3:** Details for descriptive and potential confounder variables.

**Supplementary Table S5:** The association between the cord blood lipid module eigenlipids and subsequent CBCL-ADHD and CBCL-ASD binary outcomes at 2 years.

**Supplementary Table S6.1:** The association between the individual cord blood lipid concentrations and subsequent CBCL-ADHP and CBCL-ASP outcomes at 2 years.

**Supplementary Table S6.2:** The association between total cord blood lipid concentrations and subsequent CBCL-ADHP and CBCL-ASP outcomes at 2 years.

**Supplementary Table S7.1:** The association between the cord blood lipid module eigenlipids and subsequent SDQ outcomes at 4 years.

**Supplementary Table S7.2:** The association between the cord blood lipid module eigenlipids and subsequent SDQ outcomes at 4 years (Set A confounders).

**Supplementary Table S7.3:** The association between the cord blood lipid module eigenlipids and subsequent SDQ outcomes at 4 years (Set B confounders).

**Supplementary Table S8.1:** Mediation by the either NOMPS at birth or GlycA at birth of the relationships between the lipid module eigenlipids and CBCL-ADHP.

**Supplementary Table S8.2:** Mediation by the either NOMPS at birth or GlycA at birth of the relationships between the lipid module eigenlipids and CBCL-ASP.

**Supplementary Table S9:** Mutually adjusted model of all eigenlipids and subsequent CBCL-ADHP and CBCL-ASP outcomes at 2 years.

**Supplementary Fig. S2:** Correlation matrix of the lipid modules, neurodevelopmental outcomes and covariates used in regression models.

| ***Supplementary Table S1:* Additional adjustment factors considered in change-in-estimate analyses.** |  |
| --- | --- |
| **Additional adjustments in the investigation of the association between the top three lipid modules (Cyan, Turquoise, and Brown) and the ADHD and ASD outcomes for Set A confounders** |  |
| These factors did not change the magnitude of association between the lipid modules and the outcomes by more than 10%: Maternal age (years), (<25yr vs >25yr), Number of additional household members, PC1 of maternal diet during pregnancy (modern healthy), PC2 of maternal diet during pregnancy (western unhealthy), any smoking during pregnancy. |  |
|  |  |
|  |  |
|  |  |

**a**


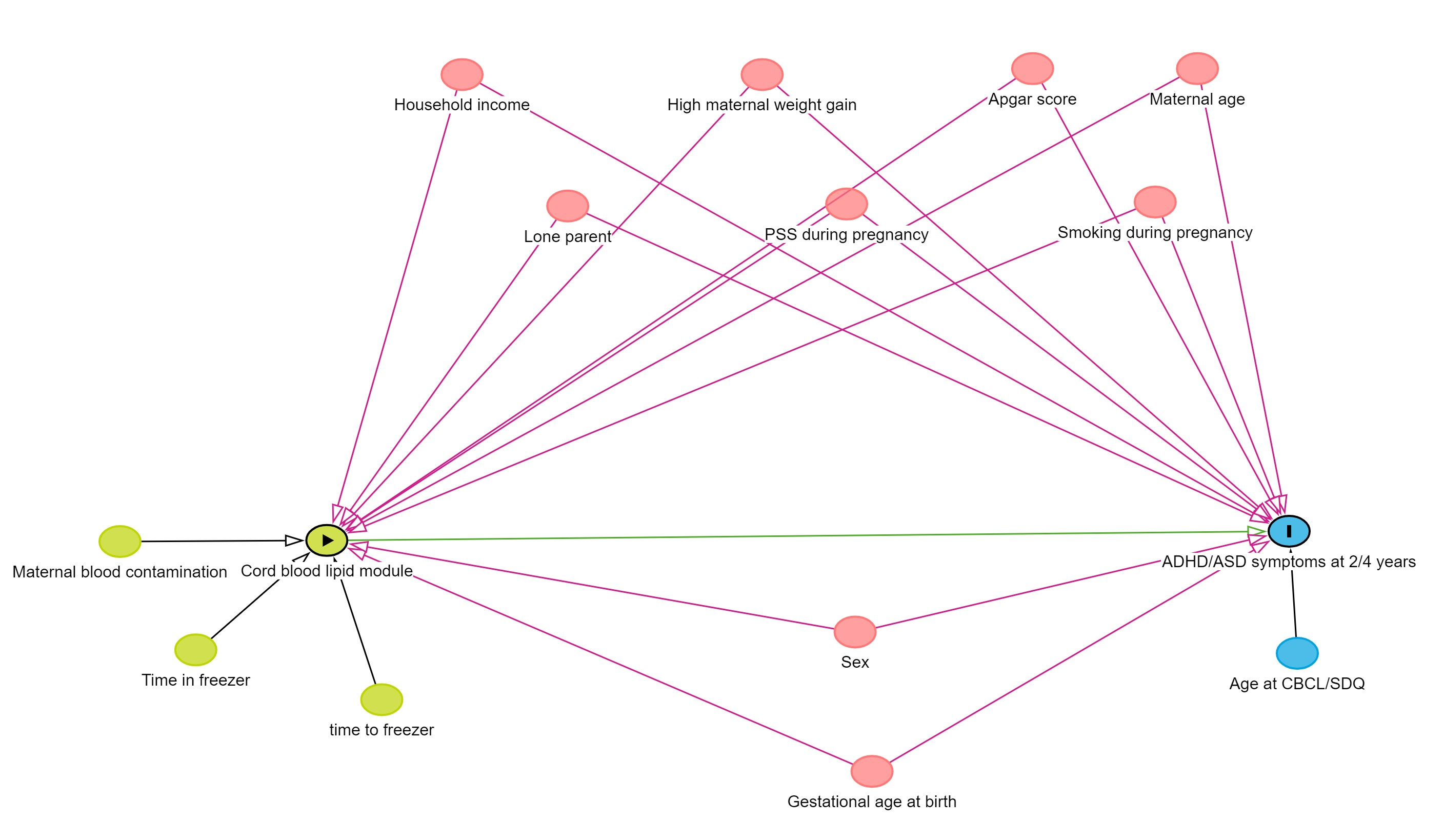


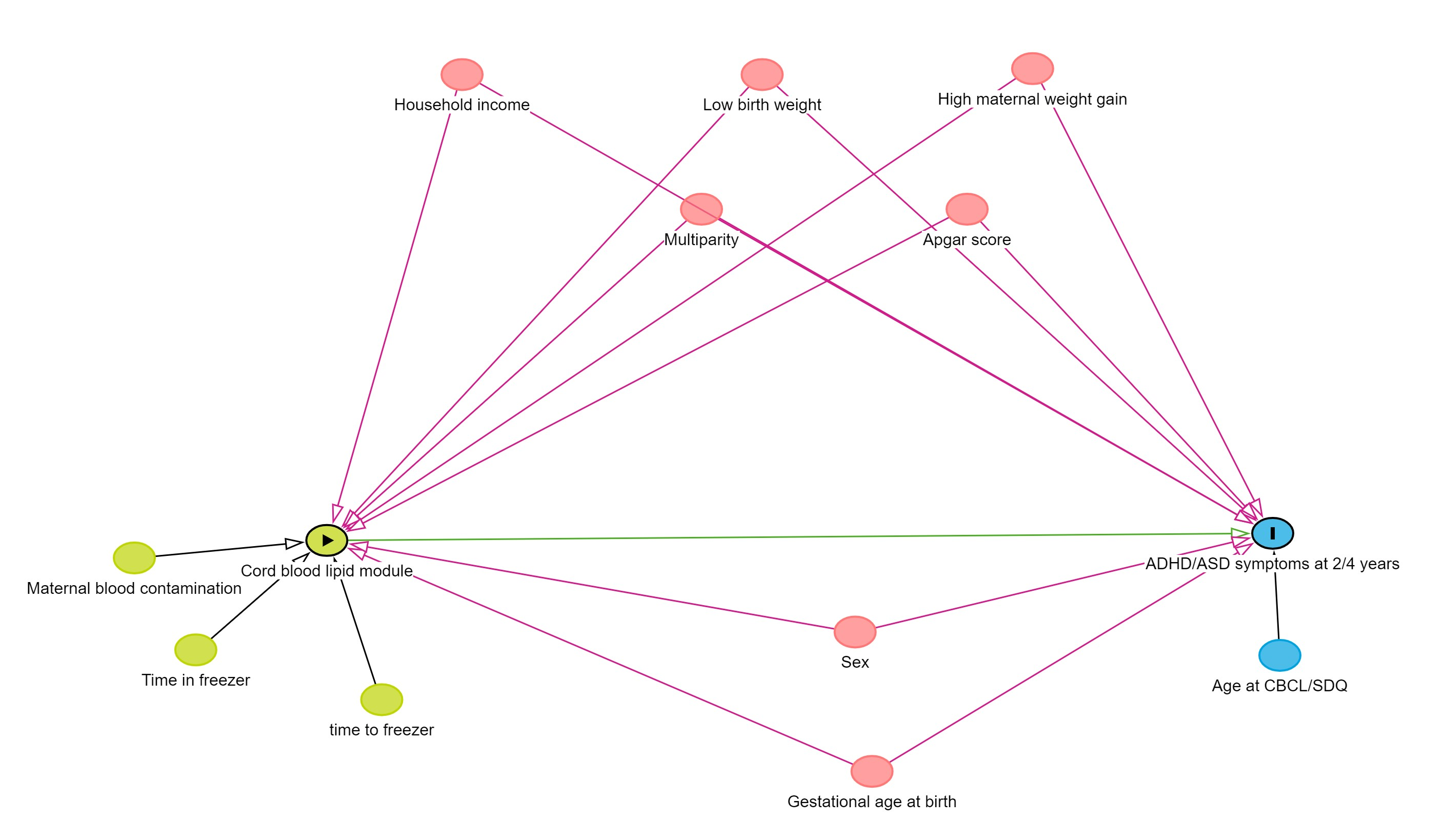


*****

*****

*****

*****

*****

**b**

***Supplementary Fig. S1.1:* Directed Acyclic Graph (DAG) for (a) adjustment Set A and (b) adjustment Set B.** Interrelationships between multiple exposure levels and covariates are not shown. For these interrelationships, see the correlation matrix in Figure S2.

**Note:** CBCL, Child Behavior Checklist; SDQ, Strengths and Difficulties Questionnaire; PSS, Perceived Stress Score.

* Partial antecedent or mediator ^1,2^


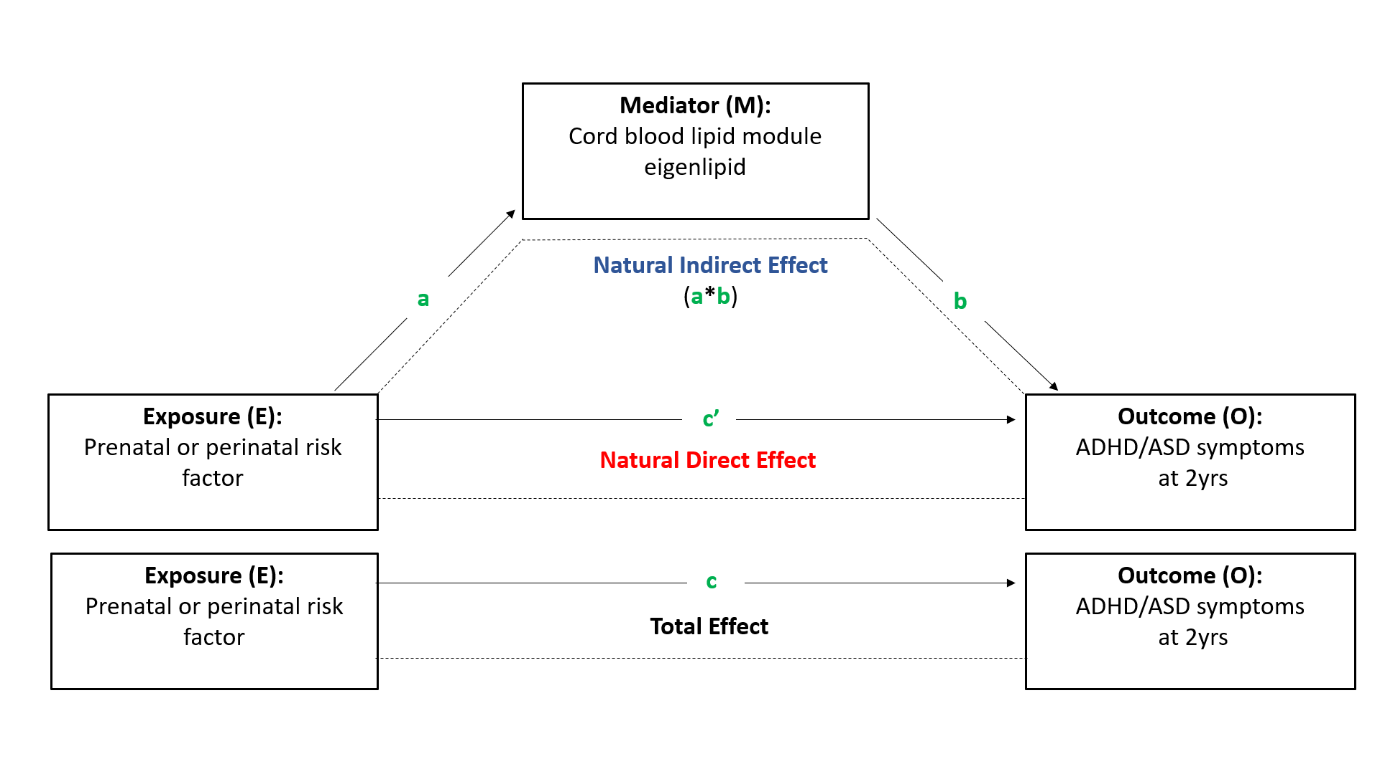


***
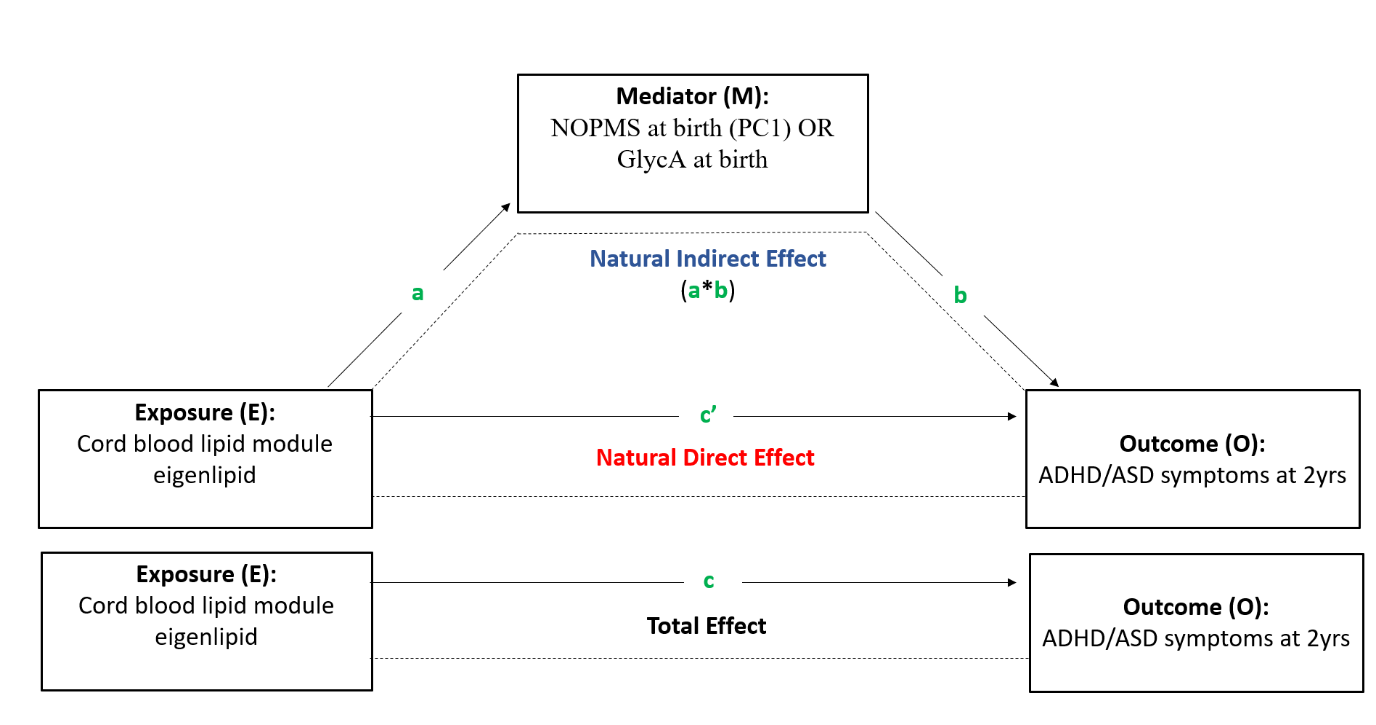
***

***Supplementary Fig. S1.2:* Mediation concept diagram.** Top panel shows the mediation concept diagram for mediation analysis. Bottom panel states the exposure and mediator in the reversed position for the applicable analysis.

**Note:** NOPMS, Non-oxidative pyruvate metabolism; GlycA, Glycoprotein Acetylation; PC1 Principal Component 1.

***Supplementary Table S2:* The composition of the lipid modules, ranked by kWithin.**

| **Module** | **Lipid Feature** | **kTotal** | **kWithin** | **kOut** | **kDiff** |
| --- | --- | --- | --- | --- | --- |
| **black (hub)** | **TG(O-54:2) [NL-18:1]** | **6.00** | **3.83** | **2.17** | **1.66** |
| black | **TG(O-52:1) [NL-18:1]** | 5.36 | 3.81 | 1.55 | 2.26 |
| black | **TG(O-50:1) [NL-18:1]** | 5.04 | 3.78 | 1.26 | 2.51 |
| black | **TG(O-52:2) [NL-18:1]** | 5.59 | 3.28 | 2.31 | 0.98 |
| black | **TG(O-50:1) [NL-16:0]** | 4.49 | 3.19 | 1.30 | 1.88 |
| black | **TG(O-52:1) [NL-16:0]** | 4.64 | 3.04 | 1.60 | 1.44 |
| black | **TG(O-52:2) [SIM]** | 3.06 | 2.68 | 0.38 | 2.30 |
| black | **TG(O-52:2) [NL-17:1]** | 5.09 | 2.60 | 2.48 | 0.12 |
| black | **TG(O-52:1) [SIM]** | 2.56 | 2.43 | 0.13 | 2.30 |
| black | **TG(O-54:2) [SIM]** | 2.36 | 2.25 | 0.11 | 2.14 |
| black | **TG(O-50:1) [SIM]** | 2.10 | 2.06 | 0.05 | 2.01 |
| black | **TG(O-54:3) [NL-18:1]** | 4.13 | 1.93 | 2.20 | -0.26 |
| black | **TG(O-52:2) [NL-16:0]** | 5.06 | 1.63 | 3.44 | -1.81 |
| black | **TG(O-50:1) [NL-17:1]** | 1.56 | 1.43 | 0.12 | 1.31 |
| black | **TG(O-52:0) [NL-16:0]** | 1.15 | 0.80 | 0.35 | 0.45 |
| black | **TG(O-50:1) [NL-15:0]** | 1.04 | 0.70 | 0.34 | 0.37 |
| black | **TG(O-50:2) [NL-18:2]** | 1.39 | 0.63 | 0.76 | -0.13 |
| black | **TG(O-50:2) [NL-18:1]** | 0.64 | 0.61 | 0.03 | 0.58 |
| black | TG(O-50:2) [SIM] | 0.44 | 0.44 | 0.01 | 0.43 |
| black | TG(O-54:2) [NL-17:1] | 0.42 | 0.32 | 0.09 | 0.23 |
| black | TG(O-50:2) [NL-16:1] | 0.26 | 0.24 | 0.02 | 0.23 |
| black | TG(O-50:3) [SIM] | 0.12 | 0.11 | 0.01 | 0.10 |
| black | TG(O-54:4) [NL-18:2] | 0.22 | 0.06 | 0.16 | -0.09 |
| **blue (hub)** | SM(d18:1/16:0) | **16.69** | **13.25** | **3.44** | **9.81** |
| blue | SM(d17:1/16:0) | 18.90 | 12.61 | 6.29 | 6.31 |
| blue | SM(d18:1/14:0/SM(d16:1/16:0) | 17.48 | 12.12 | 5.36 | 6.76 |
| blue | SM(37:1) | 15.39 | 11.98 | 3.41 | 8.58 |
| blue | SM(d18:1/20:0)/SM(d16:1/22:0) | 14.43 | 11.82 | 2.60 | 9.22 |
| blue | SM(d18:2/17:0) | 14.02 | 11.43 | 2.59 | 8.84 |
| blue | SM(40:3) (b) | 13.85 | 11.40 | 2.45 | 8.96 |
| blue | PC(P-38:5) (a) | 13.60 | 11.29 | 2.31 | 8.98 |
| blue | SM(35:2) (b) | 14.68 | 11.06 | 3.61 | 7.45 |
| blue | SM(34:3) | 12.80 | 10.41 | 2.38 | 8.03 |
| blue | PC(P-16:0/20:4) | 11.44 | 10.41 | 1.03 | 9.38 |
| blue | PC(P-16:0/18:1) | 11.35 | 10.19 | 1.16 | 9.02 |
| blue | PC(O-16:0/20:4) | 11.15 | 10.05 | 1.10 | 8.94 |
| blue | SM(d18:2/16:0) | 11.45 | 9.96 | 1.49 | 8.47 |
| blue | SM(d16:1/23:0)/SM(d17:1/22:0) | 16.75 | 9.93 | 6.82 | 3.11 |
| blue | SM(37:2) | 11.64 | 9.84 | 1.80 | 8.04 |
| blue | SM(d18:1/18:0/SM(d16:1/20:0) | 11.15 | 9.70 | 1.46 | 8.24 |
| blue | SM(d16:1/24:1) | 13.02 | 9.62 | 3.40 | 6.23 |
| blue | SM(d17:1/24:1) | 12.20 | 9.45 | 2.75 | 6.70 |
| blue | PC(P-18:0/20:4) | 10.42 | 9.37 | 1.05 | 8.33 |
| blue | SM(d18:1/24:1) | 12.17 | 9.24 | 2.93 | 6.31 |
| blue | SM(d18:2/14:0) | 10.36 | 8.89 | 1.47 | 7.42 |
| blue | PC(O-32:2) | 13.12 | 8.60 | 4.53 | 4.07 |
| blue | PC(O-38:5) | 9.99 | 8.56 | 1.43 | 7.13 |
| blue | SM(d17:1/14:0) | 12.32 | 8.46 | 3.86 | 4.61 |
| blue | SM(d18:1/22:0)/SM(d16:1/24:0) | 11.67 | 8.45 | 3.22 | 5.24 |
| blue | SM(d18:2/18:1) | 11.20 | 8.39 | 2.81 | 5.59 |
| blue | PC(P-16:0/14:0) | 9.45 | 7.94 | 1.52 | 6.42 |
| blue | PC(P-18:1/22:6) | 11.31 | 7.85 | 3.46 | 4.39 |
| blue | PC(O-18:1/18:1) | 11.86 | 7.84 | 4.03 | 3.81 |
| blue | PC(17:0_18:1) | 12.86 | 7.80 | 5.06 | 2.74 |
| blue | PC(O-18:0/18:1) | 11.46 | 7.23 | 4.23 | 3.00 |
| blue | SM(d18:2/22:0) | 7.84 | 7.15 | 0.70 | 6.45 |
| blue | PC(16:0_18:0) | 8.75 | 7.09 | 1.65 | 5.44 |
| blue | PC(33:1) | 13.26 | 7.07 | 6.19 | 0.88 |
| blue | PC(P-17:0/20:4) (b) | 8.31 | 7.04 | 1.26 | 5.78 |
| blue | SM(d18:2/23:0) | 10.68 | 7.00 | 3.68 | 3.32 |
| blue | SM(43:2) (b) | 8.87 | 6.94 | 1.93 | 5.02 |
| blue | PC(P-16:0/18:0) | 8.95 | 6.84 | 2.12 | 4.72 |
| blue | PC(33:0) (b) | 9.21 | 6.76 | 2.44 | 4.32 |
| blue | PC(O-16:0/16:0) | 7.68 | 6.74 | 0.93 | 5.81 |
| blue | PC(O-40:5) | 9.38 | 6.68 | 2.70 | 3.98 |
| blue | SM(d18:1/23:0)/SM(d17:1/24:0) | 13.70 | 6.56 | 7.14 | -0.57 |
| blue | PC(P-18:0/22:5) | 7.50 | 6.55 | 0.96 | 5.59 |
| blue | PC(O-40:7) (a) | 7.88 | 6.50 | 1.38 | 5.12 |
| blue | PC(P-18:0/22:6) | 7.75 | 6.32 | 1.43 | 4.89 |
| blue | PC(O-18:0/20:4) | 7.13 | 6.31 | 0.82 | 5.50 |
| blue | PC(36:4) [+OH] | 7.46 | 6.14 | 1.32 | 4.82 |
| blue | PC(16:0_18:1) | 8.57 | 6.07 | 2.50 | 3.57 |
| blue | PC(P-16:0/22:6) | 6.75 | 6.01 | 0.74 | 5.27 |
| blue | PC(18:0_18:1) | 7.87 | 5.96 | 1.90 | 4.06 |
| blue | PC(P-38:5) (b) | 6.04 | 5.52 | 0.52 | 5.00 |
| blue | SM(d18:2/24:0) | 5.82 | 5.44 | 0.38 | 5.05 |
| blue | PC(15-MHDA_18:1) | 8.44 | 5.42 | 3.02 | 2.40 |
| blue | PC(P-16:0/16:1) | 6.13 | 5.35 | 0.77 | 4.58 |
| blue | PC(15:0_20:4) | 8.08 | 5.34 | 2.73 | 2.61 |
| blue | PC(P-36:3) | 6.00 | 5.20 | 0.80 | 4.41 |
| blue | SM(d16:1/19:0) | 7.22 | 5.16 | 2.06 | 3.11 |
| blue | PC(P-16:0/16:0) | 5.57 | 5.12 | 0.45 | 4.67 |
| blue | PC(14:0_16:0) | 7.26 | 5.06 | 2.20 | 2.85 |
| blue | SM(d18:1/17:0)/SM(d17:1/18:0) | 5.72 | 5.01 | 0.71 | 4.30 |
| blue | SM(40:3) (a) | 5.73 | 5.01 | 0.73 | 4.28 |
| blue | PC(O-35:4) | 6.55 | 4.96 | 1.59 | 3.37 |
| blue | PC(P-17:0/20:4) (a) | 5.65 | 4.92 | 0.73 | 4.19 |
| blue | PC(39:5)(b) | 5.85 | 4.88 | 0.97 | 3.91 |
| blue | PC(38:5) (a) | 5.72 | 4.86 | 0.87 | 3.99 |
| blue | SM(d18:1/24:0) | 5.77 | 4.73 | 1.03 | 3.70 |
| blue | COH | 5.94 | 4.58 | 1.36 | 3.22 |
| blue | PC(O-18:0/22:6) | 5.15 | 4.47 | 0.68 | 3.80 |
| blue | PC(O-34:1) | 5.61 | 4.41 | 1.20 | 3.21 |
| blue | PC(O-16:0/22:6) | 4.62 | 4.15 | 0.47 | 3.67 |
| blue | PC(38:6) [+OH] | 4.92 | 4.03 | 0.89 | 3.14 |
| blue | PC(15-MHDA_20:4) | 4.38 | 3.64 | 0.74 | 2.91 |
| blue | PC(40:8) | 5.36 | 3.64 | 1.72 | 1.91 |
| blue | Hex3Cer(d18:1/16:0) | 4.37 | 3.51 | 0.86 | 2.65 |
| blue | PC(31:0) (b) | 4.41 | 3.45 | 0.96 | 2.49 |
| blue | CE(18:1) | 4.87 | 3.38 | 1.50 | 1.88 |
| blue | PC(P-15:0/20:4) (b) | 4.60 | 3.34 | 1.27 | 2.07 |
| blue | PC(38:7)(c) | 3.86 | 3.19 | 0.67 | 2.52 |
| blue | PC(P-20:0/20:4) | 3.38 | 3.16 | 0.22 | 2.95 |
| blue | PC(15:0_22:6) | 4.75 | 3.13 | 1.62 | 1.51 |
| blue | PC(14:0_22:6) | 6.07 | 2.97 | 3.09 | -0.12 |
| blue | PC(16:0_16:0) | 3.74 | 2.96 | 0.78 | 2.19 |
| blue | SM(43:2) (c) | 3.23 | 2.81 | 0.42 | 2.39 |
| blue | PC(16:1_22:6) | 3.81 | 2.79 | 1.02 | 1.78 |
| blue | PC(17:0_20:4) | 2.90 | 2.64 | 0.26 | 2.38 |
| blue | PC(33:0) (a) | 3.18 | 2.53 | 0.65 | 1.87 |
| blue | PC(O-16:0/20:3) | 3.44 | 2.48 | 0.96 | 1.51 |
| blue | PC(17:0_22:6) | 3.22 | 2.32 | 0.90 | 1.42 |
| blue | PC(P-18:1/18:1) | 2.77 | 2.24 | 0.53 | 1.71 |
| blue | SM(38:3) (b) | 2.47 | 2.16 | 0.31 | 1.86 |
| blue | SM(d18:2/18:0) | 2.26 | 2.11 | 0.15 | 1.95 |
| blue | PC(O-32:1) | 2.43 | 2.03 | 0.40 | 1.62 |
| blue | CE(16:0) | 2.33 | 2.01 | 0.32 | 1.70 |
| blue | SM(d19:1/24:1) | 2.32 | 1.99 | 0.33 | 1.66 |
| blue | Hex3Cer(d18:1/24:1) | 2.20 | 1.96 | 0.23 | 1.73 |
| blue | SM(43:1) | 2.20 | 1.94 | 0.26 | 1.67 |
| blue | PC(31:0) (a) | 3.45 | 1.87 | 1.58 | 0.29 |
| blue | PC(15-MHDA_22:6) | 2.53 | 1.86 | 0.66 | 1.20 |
| blue | Hex3Cer(d18:1/22:0) | 2.58 | 1.85 | 0.73 | 1.12 |
| blue | PC(39:5)(a) | 2.51 | 1.79 | 0.72 | 1.07 |
| blue | PC(18:1_22:6) (b) | 2.10 | 1.73 | 0.37 | 1.36 |
| blue | PC(40:7) (a) | 2.71 | 1.67 | 1.04 | 0.64 |
| blue | PC(38:5) (b) | 2.04 | 1.59 | 0.45 | 1.14 |
| blue | PC(O-34:4) | 2.52 | 1.49 | 1.03 | 0.47 |
| blue | SM(d18:2/20:0) | 1.62 | 1.47 | 0.15 | 1.32 |
| blue | PC(38:4) (b) | 1.91 | 1.39 | 0.52 | 0.86 |
| blue | LPC(24:0) [sn1] | 1.39 | 1.24 | 0.15 | 1.10 |
| blue | SM(38:3) (a) | 1.39 | 1.24 | 0.15 | 1.10 |
| blue | PC(16:0_22:6) | 1.81 | 1.22 | 0.58 | 0.64 |
| blue | LPC(24:0) [sn2] | 1.29 | 1.17 | 0.12 | 1.05 |
| blue | PC(18:0_22:5) (n3) | 2.12 | 1.13 | 0.99 | 0.14 |
| blue | SM(41:1) (a) | 1.20 | 1.01 | 0.19 | 0.82 |
| blue | LPC(22:0) [sn1] | 1.38 | 1.00 | 0.38 | 0.61 |
| blue | SM(44:2) | 1.04 | 0.92 | 0.12 | 0.80 |
| blue | PC(20:0_20:4) | 0.93 | 0.82 | 0.11 | 0.70 |
| blue | Cer(d19:1/24:1) | 1.71 | 0.81 | 0.90 | -0.09 |
| blue | Sulfatide (d18:1:/16:0) | 1.64 | 0.76 | 0.88 | -0.11 |
| blue | GM3(d18:1/24:1) | 0.83 | 0.75 | 0.08 | 0.67 |
| blue | SM(44:3) | 0.79 | 0.68 | 0.11 | 0.56 |
| blue | LPC(26:0) [sn1] | 0.72 | 0.66 | 0.06 | 0.60 |
| blue | GM3(d18:1/22:0) | 0.69 | 0.65 | 0.04 | 0.61 |
| blue | LPC(22:0) [sn2] | 0.85 | 0.64 | 0.21 | 0.43 |
| blue | PC(18:1_22:6) (a) | 0.80 | 0.61 | 0.18 | 0.43 |
| blue | GM3(d18:1/16:0) | 0.67 | 0.57 | 0.09 | 0.48 |
| blue | Hex3Cer(d18:1/24:0) | 0.61 | 0.53 | 0.08 | 0.45 |
| blue | SM(d18:0/14:0) | 0.71 | 0.53 | 0.19 | 0.34 |
| blue | GM1(d18:1/16:0) | 0.71 | 0.53 | 0.18 | 0.35 |
| blue | Cer(d19:1/24:0) | 1.07 | 0.50 | 0.57 | -0.06 |
| blue | PC(18:0_20:4) | 0.47 | 0.41 | 0.06 | 0.35 |
| blue | Hex2Cer(d18:2/16:0) | 0.71 | 0.39 | 0.32 | 0.07 |
| blue | GM3(d18:1/24:0) | 0.41 | 0.37 | 0.04 | 0.33 |
| blue | LPC(26:0) [sn2] | 0.37 | 0.32 | 0.04 | 0.28 |
| blue | Hex3Cer(d18:1/20:0) | 0.34 | 0.31 | 0.03 | 0.28 |
| blue | Sulfatide (d18:1:/24:1) | 0.33 | 0.29 | 0.04 | 0.24 |
| blue | PC(44:12) | 0.58 | 0.28 | 0.30 | -0.01 |
| blue | Hex2Cer(d18:2/24:1) | 0.32 | 0.25 | 0.07 | 0.18 |
| blue | PC(16:0_20:4) | 0.23 | 0.22 | 0.01 | 0.21 |
| blue | PC(18:0_22:4) | 0.40 | 0.22 | 0.19 | 0.03 |
| blue | SM(44:1) | 0.28 | 0.20 | 0.07 | 0.13 |
| blue | SM(41:0) | 0.18 | 0.16 | 0.02 | 0.14 |
| blue | Cer(d20:1/24:1) | 0.34 | 0.14 | 0.20 | -0.05 |
| blue | PC(O-40:7) (b) | 0.16 | 0.13 | 0.03 | 0.10 |
| blue | CE(22:6) [+OH] | 0.15 | 0.12 | 0.03 | 0.09 |
| blue | Sulfatide (d18:1:/24:0) | 0.12 | 0.11 | 0.01 | 0.10 |
| blue | Hex2Cer(d16:1/24:1) | 0.19 | 0.10 | 0.10 | 0.00 |
| blue | Sulfatide (d18:1:/24:1(OH)) | 0.11 | 0.06 | 0.05 | 0.01 |
| blue | LPI(18:1) [sn1] | 0.14 | 0.04 | 0.10 | -0.06 |
| **brown (hub)** | **TG(50:3) [SIM]** | **22.99** | **21.32** | **1.67** | **19.64** |
| brown | TG(48:2) [NL-14:0] | 21.47 | 20.26 | 1.21 | 19.06 |
| brown | TG(50:3) [NL-18:2] | 21.92 | 19.72 | 2.20 | 17.53 |
| brown | TG(52:4) [SIM] | 21.87 | 19.01 | 2.86 | 16.15 |
| brown | TG(50:4) [SIM] | 21.95 | 18.99 | 2.95 | 16.04 |
| brown | TG(52:4) [NL-18:3] | 20.48 | 18.23 | 2.26 | 15.97 |
| brown | TG(50:3) [NL-14:0] | 19.05 | 17.61 | 1.44 | 16.16 |
| brown | TG(48:2) [SIM] | 17.94 | 17.20 | 0.75 | 16.45 |
| brown | TG(52:3) [SIM] | 18.74 | 17.19 | 1.55 | 15.65 |
| brown | TG(48:2) [NL-18:2] | 18.48 | 16.86 | 1.62 | 15.24 |
| brown | TG(50:3) [NL-16:1] | 17.15 | 16.18 | 0.97 | 15.21 |
| brown | TG(51:2) [SIM] | 17.56 | 15.97 | 1.59 | 14.38 |
| brown | TG(52:4) [NL-16:1] | 16.82 | 15.50 | 1.33 | 14.17 |
| brown | TG(48:3) [NL-14:0] | 17.02 | 15.23 | 1.79 | 13.44 |
| brown | TG(50:2) [SIM] | 15.87 | 15.11 | 0.76 | 14.36 |
| brown | TG(50:4) [NL-18:3] | 17.85 | 15.10 | 2.75 | 12.36 |
| brown | TG(52:3) [NL-18:2] | 16.47 | 14.87 | 1.60 | 13.26 |
| brown | TG(50:3) [NL-18:3] | 17.31 | 14.84 | 2.47 | 12.36 |
| brown | TG(48:3) [NL-18:3] | 16.77 | 14.69 | 2.08 | 12.61 |
| brown | TG(51:2) [NL-17:1] | 15.86 | 14.56 | 1.30 | 13.25 |
| brown | TG(51:2) [NL-17:0] | 15.86 | 14.55 | 1.31 | 13.24 |
| brown | TG(52:5) [SIM] | 20.04 | 14.41 | 5.63 | 8.78 |
| brown | TG(48:3) [SIM] | 15.36 | 14.39 | 0.97 | 13.42 |
| brown | TG(48:1) [NL-18:1] | 14.81 | 14.04 | 0.77 | 13.27 |
| brown | TG(54:4) [SIM] | 15.90 | 13.74 | 2.16 | 11.58 |
| brown | TG(50:4) [NL-14:0] | 16.27 | 13.57 | 2.70 | 10.87 |
| brown | TG(53:2) [NL-18:1] | 14.79 | 13.52 | 1.27 | 12.25 |
| brown | TG(52:4) [NL-18:2] | 15.11 | 13.37 | 1.74 | 11.63 |
| brown | TG(52:5) [NL-18:3] | 15.90 | 13.24 | 2.66 | 10.58 |
| brown | TG(53:2) [SIM] | 13.98 | 12.94 | 1.03 | 11.91 |
| brown | TG(50:1) [SIM] | 13.43 | 12.82 | 0.62 | 12.20 |
| brown | TG(52:2) [SIM] | 13.31 | 12.75 | 0.56 | 12.20 |
| brown | TG(48:2) [NL-14:1] | 13.27 | 12.48 | 0.78 | 11.70 |
| brown | TG(50:1) [NL-16:0] | 12.66 | 12.04 | 0.62 | 11.42 |
| brown | TG(48:1) [SIM] | 12.37 | 11.90 | 0.47 | 11.44 |
| brown | TG(50:1) [NL-18:1] | 12.24 | 11.71 | 0.53 | 11.18 |
| brown | TG(51:1) [SIM] | 12.50 | 11.59 | 0.92 | 10.67 |
| brown | TG(51:1) [NL-17:0] | 12.65 | 11.44 | 1.21 | 10.23 |
| brown | TG(54:5) [NL-18:3] | 12.38 | 11.24 | 1.14 | 10.10 |
| brown | TG(52:1) [SIM] | 11.56 | 11.18 | 0.38 | 10.80 |
| brown | TG(52:1) [NL-18:0] | 11.58 | 11.16 | 0.43 | 10.73 |
| brown | TG(50:3) [NL-14:1] | 11.37 | 10.70 | 0.67 | 10.02 |
| brown | TG(51:2) [NL-15:0] | 11.90 | 10.35 | 1.55 | 8.80 |
| brown | TG(50:2) [NL-18:1] | 10.35 | 10.06 | 0.29 | 9.76 |
| brown | TG(52:1) [NL-18:1] | 10.35 | 9.98 | 0.37 | 9.61 |
| brown | TG(50:2) [NL-18:2] | 11.94 | 9.92 | 2.02 | 7.90 |
| brown | TG(50:2) [NL-16:1] | 10.26 | 9.89 | 0.37 | 9.52 |
| brown | TG(54:2) [SIM] | 9.98 | 9.50 | 0.49 | 9.01 |
| brown | TG(54:6) [NL-18:3] | 11.00 | 9.42 | 1.58 | 7.84 |
| brown | DG(16:0_18:2) | 10.47 | 9.40 | 1.07 | 8.33 |
| brown | TG(48:3) [NL-16:1] | 9.97 | 9.38 | 0.59 | 8.79 |
| brown | TG(54:2) [NL-20:1] | 10.99 | 8.47 | 2.52 | 5.95 |
| brown | TG(48:2) [NL-16:1] | 8.66 | 8.39 | 0.27 | 8.11 |
| brown | TG(52:2) [NL-16:0] | 8.71 | 8.31 | 0.40 | 7.91 |
| brown | TG(54:2) [NL-18:0] | 8.61 | 8.25 | 0.36 | 7.88 |
| brown | DG(16:0_18:1) | 9.07 | 8.02 | 1.05 | 6.98 |
| brown | TG(50:0) [SIM] | 7.98 | 7.74 | 0.24 | 7.50 |
| brown | DG(18:1_18:2) | 8.91 | 7.61 | 1.30 | 6.32 |
| brown | DG(16:1_18:1) | 8.13 | 7.47 | 0.66 | 6.81 |
| brown | TG(50:2) [NL-14:0] | 7.61 | 7.33 | 0.28 | 7.05 |
| brown | TG(50:0) [NL-18:0] | 7.59 | 7.30 | 0.29 | 7.02 |
| brown | TG(54:4) [NL-20:3] | 9.59 | 6.78 | 2.81 | 3.97 |
| brown | TG(54:3) [NL-18:2] | 7.26 | 6.71 | 0.55 | 6.16 |
| brown | DG(14:0_18:2) | 6.98 | 6.52 | 0.46 | 6.05 |
| brown | TG(54:3) [SIM] | 6.22 | 6.08 | 0.14 | 5.94 |
| brown | TG(48:0) [SIM] | 5.97 | 5.74 | 0.23 | 5.51 |
| brown | TG(49:1) [NL-17:1] | 6.11 | 5.66 | 0.45 | 5.21 |
| brown | TG(54:4) [NL-18:2] | 5.87 | 5.51 | 0.36 | 5.16 |
| brown | DG(18:1_18:3) | 6.01 | 5.33 | 0.69 | 4.64 |
| brown | TG(54:1) [SIM] | 5.30 | 5.22 | 0.08 | 5.14 |
| brown | TG(51:0) [NL-16:0] | 5.46 | 4.98 | 0.48 | 4.50 |
| brown | TG(53:2) [NL-17:1] | 5.03 | 4.85 | 0.19 | 4.66 |
| brown | DG(18:1_18:1) | 5.83 | 4.72 | 1.11 | 3.62 |
| brown | TG(48:1) [NL-16:1] | 4.83 | 4.63 | 0.21 | 4.42 |
| brown | TG(52:2) [NL-18:2] | 5.68 | 4.57 | 1.11 | 3.46 |
| brown | TG(52:3) [NL-16:1] | 4.37 | 4.28 | 0.09 | 4.19 |
| brown | DG(18:0_18:2) | 4.55 | 4.17 | 0.38 | 3.79 |
| brown | TG(48:0) [NL-16:0] | 4.32 | 4.11 | 0.21 | 3.90 |
| brown | TG(O-54:4) [SIM] | 4.23 | 4.08 | 0.15 | 3.94 |
| brown | DG(18:0_18:1) | 4.36 | 4.02 | 0.34 | 3.67 |
| brown | PE(18:0_22:6) | 6.03 | 3.97 | 2.06 | 1.91 |
| brown | TG(54:1) [NL-18:1] | 3.97 | 3.91 | 0.06 | 3.85 |
| brown | DG(16:0_16:0) | 4.78 | 3.85 | 0.93 | 2.92 |
| brown | TG(49:1) [SIM] | 4.17 | 3.57 | 0.60 | 2.96 |
| brown | DG(16:0_16:1) | 3.79 | 3.56 | 0.24 | 3.32 |
| brown | DG(18:1_20:4) | 4.89 | 3.29 | 1.60 | 1.70 |
| brown | DG(16:0_22:5) | 5.48 | 3.16 | 2.32 | 0.83 |
| brown | TG(54:3) [NL-18:1] | 3.00 | 2.93 | 0.07 | 2.85 |
| brown | PE(17:0_22:6) | 5.05 | 2.83 | 2.21 | 0.62 |
| brown | DG(18:1_22:5) | 3.46 | 2.63 | 0.83 | 1.80 |
| brown | DG(16:0_20:4) | 3.43 | 2.56 | 0.87 | 1.68 |
| brown | TG(50:1) [NL-14:0] | 2.39 | 2.31 | 0.08 | 2.24 |
| brown | DE(20:4) | 2.60 | 2.31 | 0.29 | 2.02 |
| brown | DG(14:0_16:0) | 2.63 | 2.25 | 0.38 | 1.87 |
| brown | DG(18:2_18:2) | 2.23 | 2.09 | 0.15 | 1.94 |
| brown | PE(16:0_22:6) | 3.37 | 1.92 | 1.45 | 0.47 |
| brown | PG(34:1) | 3.68 | 1.57 | 2.11 | -0.54 |
| brown | DG(18:1_20:3) | 1.82 | 1.47 | 0.35 | 1.12 |
| brown | TG(48:0) [NL-18:0] | 1.52 | 1.46 | 0.06 | 1.41 |
| brown | PG(36:1) | 2.17 | 1.44 | 0.72 | 0.72 |
| brown | PE(15-MHDA_22:6) | 2.59 | 1.19 | 1.40 | -0.22 |
| brown | CE(20:4) | 1.43 | 1.15 | 0.28 | 0.86 |
| brown | PI(18:0_22:5) (n3) | 2.31 | 1.10 | 1.20 | -0.10 |
| brown | TG(54:0) [NL-18:0] | 0.99 | 0.99 | 0.00 | 0.99 |
| brown | PI(15-MHDA_20:4)\PI(17:0_20:4) | 1.52 | 0.68 | 0.83 | -0.15 |
| brown | TG(54:0) [SIM] | 0.64 | 0.63 | 0.01 | 0.62 |
| brown | PI(39:6) | 0.98 | 0.54 | 0.44 | 0.10 |
| brown | PG(36:2) | 0.56 | 0.46 | 0.10 | 0.36 |
| brown | CE(16:2) | 0.67 | 0.36 | 0.31 | 0.05 |
| brown | PI(38:6) | 0.63 | 0.35 | 0.28 | 0.08 |
| brown | TG(49:1) [NL-16:1] | 0.29 | 0.27 | 0.02 | 0.25 |
| brown | PI(18:0_20:4) | 0.69 | 0.24 | 0.46 | -0.22 |
| brown | CE(20:5) | 0.28 | 0.17 | 0.11 | 0.06 |
| brown | PI(16:0_20:4) | 0.13 | 0.09 | 0.04 | 0.04 |
| brown | PI(20:0_20:4) | 0.28 | 0.08 | 0.19 | -0.11 |
| brown | DE(16:0) | 0.06 | 0.03 | 0.03 | 0.01 |
| **cyan (hub)** | **AC(14:0)** | **1.35** | **1.34** | **0.01** | **1.33** |
| cyan | AC(16:1) | 1.33 | 1.30 | 0.03 | 1.27 |
| cyan | AC(18:1) | 1.30 | 1.28 | 0.03 | 1.25 |
| cyan | AC(14:1) | 1.16 | 1.13 | 0.03 | 1.10 |
| cyan | AC(12:0) | 1.10 | 1.06 | 0.04 | 1.02 |
| cyan | AC(16:0) | 1.06 | 1.04 | 0.02 | 1.03 |
| cyan | AC(17:0) (b) | 0.81 | 0.71 | 0.09 | 0.62 |
| cyan | AC(15:0) (b) | 0.74 | 0.70 | 0.03 | 0.67 |
| cyan | AC(14:2) | 0.62 | 0.62 | 0.01 | 0.61 |
| cyan | AC(17:0) (a) | 0.66 | 0.57 | 0.09 | 0.48 |
| cyan | AC(18:0) | 0.72 | 0.50 | 0.23 | 0.27 |
| cyan | AC(18:2) | 0.46 | 0.46 | 0.00 | 0.45 |
| cyan | AC(15:0) (a) | 0.08 | 0.04 | 0.04 | 0.00 |
| **green (hub)** | **LPC(18:0) [sn1]** | **1.79** | **1.39** | **0.40** | **0.99** |
| green | LPC(18:0) [sn2] | 1.73 | 1.36 | 0.37 | 0.99 |
| green | LPC(O-18:0) | 1.25 | 1.14 | 0.10 | 1.04 |
| green | LPC(O-20:0) | 1.19 | 1.12 | 0.06 | 1.06 |
| green | LPC(O-22:1) | 1.13 | 1.10 | 0.02 | 1.08 |
| green | LPC(O-18:1) | 1.23 | 1.08 | 0.16 | 0.92 |
| green | LPC(16:0) [sn1] | 1.55 | 1.07 | 0.48 | 0.60 |
| green | LPC(16:0) [sn2] | 1.37 | 0.96 | 0.41 | 0.56 |
| green | LPC(20:1) [sn1] | 1.32 | 0.95 | 0.37 | 0.58 |
| green | LPC(O-22:0) | 1.09 | 0.93 | 0.16 | 0.76 |
| green | LPC(O-20:1) | 0.95 | 0.89 | 0.06 | 0.84 |
| green | LPC(P-16:0) | 1.04 | 0.88 | 0.16 | 0.73 |
| green | LPC(P-18:1) | 1.02 | 0.78 | 0.23 | 0.55 |
| green | LPC(O-24:1) | 1.03 | 0.78 | 0.26 | 0.52 |
| green | LPC(20:1) [sn2] | 0.93 | 0.70 | 0.23 | 0.47 |
| green | LPC(O-16:0) | 0.77 | 0.69 | 0.08 | 0.61 |
| green | LPE(18:0) [sn2] | 0.80 | 0.67 | 0.13 | 0.54 |
| green | LPC(P-18:0) | 1.10 | 0.67 | 0.43 | 0.24 |
| green | LPC(22:1) [sn1] | 1.18 | 0.65 | 0.53 | 0.13 |
| green | LPE(16:0) [sn2] | 0.68 | 0.65 | 0.04 | 0.61 |
| green | LPE(16:0) [sn1] | 0.66 | 0.63 | 0.02 | 0.61 |
| green | LPE(18:0) [sn1] | 0.89 | 0.63 | 0.27 | 0.36 |
| green | LPC(P-17:0) (b) | 0.67 | 0.49 | 0.18 | 0.31 |
| green | LPC(P-17:0) (a) | 0.63 | 0.41 | 0.22 | 0.19 |
| green | LPC(O-24:0) | 1.44 | 0.38 | 1.06 | -0.68 |
| green | LPC(22:1) [sn2] | 0.48 | 0.30 | 0.18 | 0.12 |
| green | LPC(O-24:2) | 0.18 | 0.18 | 0.01 | 0.17 |
| green | LPC(P-20:0) | 0.19 | 0.16 | 0.03 | 0.13 |
| **greenyellow (hub)** | **LPC(18:2) [sn1]** | **1.98** | **1.58** | **0.41** | **1.17** |
| greenyellow | LPE(18:2) [sn1] | 1.70 | 1.43 | 0.28 | 1.15 |
| greenyellow | LPC(18:1) [sn1] | 2.00 | 1.39 | 0.61 | 0.77 |
| greenyellow | LPC(18:2) [sn2] | 1.41 | 1.29 | 0.13 | 1.16 |
| greenyellow | LPC(18:1) [sn2] | 1.86 | 1.22 | 0.65 | 0.57 |
| greenyellow | LPC(18:3) [sn1] (a)/LPC(18:3) [sn2] (b) | 1.89 | 1.19 | 0.70 | 0.49 |
| greenyellow | LPE(18:2) [sn2] | 1.33 | 1.13 | 0.20 | 0.93 |
| greenyellow | LPC(20:2) [sn1] | 1.69 | 0.94 | 0.75 | 0.18 |
| greenyellow | LPC(18:3) (a) [sn1] [104_sn1] | 1.02 | 0.90 | 0.11 | 0.79 |
| greenyellow | LPC(20:2) [sn2] | 1.21 | 0.81 | 0.40 | 0.42 |
| greenyellow | LPC(20:5) [sn1] | 0.88 | 0.79 | 0.09 | 0.70 |
| greenyellow | LPE(18:1) [sn1] | 0.89 | 0.76 | 0.12 | 0.64 |
| greenyellow | LPE(18:1) [sn2] | 0.85 | 0.76 | 0.10 | 0.66 |
| greenyellow | LPC(20:5) [sn2] | 0.82 | 0.75 | 0.08 | 0.67 |
| greenyellow | LPC(18:3) [sn2] (a) | 0.51 | 0.47 | 0.03 | 0.44 |
| grey | CE(22:6) | 0.62 | 0.48 | 0.13 | 0.35 |
| grey | CE(22:5) (n3) | 0.53 | 0.47 | 0.06 | 0.41 |
| grey | Cer(d18:0/22:0) | 0.42 | 0.38 | 0.04 | 0.33 |
| grey | LPC(22:6) [+OH] | 0.38 | 0.35 | 0.03 | 0.31 |
| grey | PI(16:0_20:3) (b) | 0.59 | 0.30 | 0.30 | 0.00 |
| grey | LPC(20:4) [+OH] | 0.32 | 0.30 | 0.02 | 0.27 |
| grey | PI(18:0_20:3) (b) | 0.66 | 0.29 | 0.37 | -0.08 |
| grey | Cer(d18:0/24:1) | 0.29 | 0.22 | 0.07 | 0.14 |
| grey | Cer(d18:0/24:0) | 0.22 | 0.21 | 0.01 | 0.20 |
| grey | PC(34:2) [+OH] | 0.20 | 0.19 | 0.01 | 0.19 |
| grey | CE(18:2) [+OH] | 0.20 | 0.19 | 0.00 | 0.19 |
| grey | LPC(18:2) [+OH] | 0.18 | 0.17 | 0.02 | 0.15 |
| grey | LPE(17:0) [sn1] | 0.13 | 0.13 | 0.00 | 0.13 |
| grey | LPE(17:0) [sn2] | 0.12 | 0.12 | 0.00 | 0.12 |
| grey | DE(22:6) | 0.13 | 0.11 | 0.02 | 0.09 |
| grey | Cer(d18:0/20:0) | 0.12 | 0.08 | 0.05 | 0.03 |
| grey | Sph(d18:1) | 0.05 | 0.05 | 0.00 | 0.04 |
| grey | Sph(d16:1) | 0.05 | 0.05 | 0.00 | 0.05 |
| grey | Hex3Cer(d18:1/18:0) | 0.14 | 0.03 | 0.12 | -0.09 |
| grey | GM3(d18:1/18:0) | 0.14 | 0.03 | 0.11 | -0.08 |
| grey | Cer(d18:1/17:0) | 0.02 | 0.02 | 0.00 | 0.02 |
| grey | LPI(18:2) [sn2] | 0.04 | 0.02 | 0.02 | 0.00 |
| grey | LPI(18:2) [sn1] | 0.04 | 0.02 | 0.02 | 0.00 |
| grey | Cer(d18:1/26:0) | 0.09 | 0.02 | 0.07 | -0.05 |
| grey | PI(18:0_22:5) (n6) | 0.02 | 0.02 | 0.00 | 0.01 |
| grey | Cer(d19:1/26:0) | 0.01 | 0.01 | 0.00 | 0.01 |
| grey | Cer(d20:1/24:0) | 0.03 | 0.01 | 0.02 | -0.02 |
| grey | SM(d18:0/16:0) | 0.14 | 0.00 | 0.14 | -0.13 |
| grey | LPI(18:0) [sn1] | 0.04 | 0.00 | 0.04 | -0.04 |
| grey | LPE(P-20:0) | 0.16 | 0.00 | 0.16 | -0.16 |
| grey | PI(38:5) (a) | 0.02 | 0.00 | 0.02 | -0.02 |
| grey | LPI(18:0) [sn2] | 0.00 | 0.00 | 0.00 | 0.00 |
| grey | DE(20:5) | 0.04 | 0.00 | 0.04 | -0.04 |
| grey | Cer(d20:1/26:0) | 0.00 | 0.00 | 0.00 | 0.00 |
| grey | TG(51:0) [SIM] | 0.00 | 0.00 | 0.00 | 0.00 |
| grey | PC(40:10) | 0.01 | 0.00 | 0.01 | -0.01 |
| grey | TG(O-54:3) [SIM] | 0.01 | 0.00 | 0.01 | -0.01 |
| grey | LPI(18:1) [sn2] | 0.01 | 0.00 | 0.01 | -0.01 |
| grey | FA(16:2) | 0.00 | 0.00 | 0.00 | 0.00 |
| grey | FA(18:0) | 0.01 | 0.00 | 0.01 | -0.01 |
| grey | GM3(d18:1/20:0) | 0.00 | 0.00 | 0.00 | 0.00 |
| grey | TG(O-52:0) [SIM] | 0.01 | 0.00 | 0.01 | -0.01 |
| grey | LPC(19:1) (c) | 0.03 | 0.00 | 0.03 | -0.03 |
| grey | PI(18:0_20:3) (a) | 0.00 | 0.00 | 0.00 | 0.00 |
| grey | Hex2Cer(d18:1/18:0) | 0.01 | 0.00 | 0.01 | -0.01 |
| grey | DG(18:0_22:6) | 0.00 | 0.00 | 0.00 | 0.00 |
| grey | Cer(d18:2/17:0) | 0.00 | 0.00 | 0.00 | 0.00 |
| grey | PE(36:0) | 0.01 | 0.00 | 0.01 | -0.01 |
| grey | TG(O-50:3) [NL-18:2] | 0.00 | 0.00 | 0.00 | 0.00 |
| grey | PIP1(38:4) | 0.00 | 0.00 | 0.00 | 0.00 |
| grey | AC(13:0) | 0.03 | 0.00 | 0.03 | -0.03 |
| grey | Sulfatide (d18:1:/24:0(OH)) | 0.00 | 0.00 | 0.00 | 0.00 |
| grey | TG(O-54:3) [NL-17:1] | 0.00 | 0.00 | 0.00 | 0.00 |
| grey | Sph(d18:2) | 0.00 | 0.00 | 0.00 | 0.00 |
| **magenta (hub)** | **PS(38:3)** | **4.15** | **3.17** | **0.97** | **2.20** |
| magenta | PA(36:4) | 4.25 | 3.12 | 1.13 | 1.99 |
| magenta | PA(34:1) | 4.66 | 3.10 | 1.56 | 1.54 |
| magenta | PS(40:6) | 3.45 | 3.07 | 0.39 | 2.68 |
| magenta | PS(40:5) | 3.33 | 3.02 | 0.32 | 2.70 |
| magenta | PA(36:3) | 4.07 | 2.98 | 1.09 | 1.89 |
| magenta | PS(38:5) | 3.42 | 2.95 | 0.47 | 2.47 |
| magenta | PS(36:2) | 3.92 | 2.78 | 1.15 | 1.63 |
| magenta | PA(36:1) | 3.77 | 2.36 | 1.41 | 0.95 |
| magenta | PS(38:4) | 2.31 | 2.21 | 0.11 | 2.10 |
| magenta | PE(P-18:0/22:4) | 2.58 | 2.04 | 0.53 | 1.51 |
| magenta | PA(36:2) | 4.55 | 1.95 | 2.60 | -0.65 |
| magenta | PS(36:1) | 1.97 | 1.61 | 0.36 | 1.25 |
| magenta | PE(P-16:0/22:4) | 0.89 | 0.76 | 0.13 | 0.62 |
| magenta | PE(P-18:1/22:4) | 1.22 | 0.70 | 0.52 | 0.18 |
| magenta | PA(40:6) | 0.94 | 0.62 | 0.32 | 0.30 |
| magenta | DG(18:0_20:4) | 1.28 | 0.62 | 0.66 | -0.04 |
| magenta | LPE(P-18:0) | 0.85 | 0.60 | 0.25 | 0.34 |
| magenta | LPE(P-18:1) | 0.63 | 0.54 | 0.09 | 0.45 |
| magenta | LPE(P-16:0) | 0.60 | 0.48 | 0.11 | 0.37 |
| magenta | PE(20:0_20:4) | 0.68 | 0.41 | 0.27 | 0.13 |
| **midnightblue (hub)** | **LPC(19:0) [sn1] (a) / LPC(19:0) [sn2] (b)** | **2.43** | **2.33** | **0.11** | **2.22** |
| midnightblue | LPC(15-MHDA) [sn1] / LPC(17:0) [sn2] | 2.67 | 2.18 | 0.49 | 1.69 |
| midnightblue | LPC(19:0) (a) [sn1] [104_sn1] | 2.02 | 1.95 | 0.07 | 1.88 |
| midnightblue | LPC(15-MHDA) [sn1] [104_sn1] | 2.24 | 1.92 | 0.32 | 1.60 |
| midnightblue | LPC(15-MHDA) [sn2] | 2.19 | 1.88 | 0.31 | 1.56 |
| midnightblue | LPC(19:0) [sn2] (a) | 1.88 | 1.84 | 0.04 | 1.79 |
| midnightblue | LPC(19:0) [sn1] (b) | 1.83 | 1.65 | 0.18 | 1.47 |
| midnightblue | LPC(17:0) [sn1] | 1.95 | 1.34 | 0.61 | 0.73 |
| midnightblue | LPC(20:0) [sn2] | 0.91 | 0.61 | 0.30 | 0.32 |
| midnightblue | LPC(19:1) (b) | 0.87 | 0.47 | 0.40 | 0.07 |
| midnightblue | LPC(20:0) [sn1] | 1.52 | 0.46 | 1.06 | -0.60 |
| midnightblue | LPC(19:1) (a) | 0.44 | 0.27 | 0.17 | 0.10 |
| **pink (hub)** | **LPC(20:4) [sn2]** | **2.00** | **1.67** | **0.34** | **1.33** |
| pink | LPC(20:4) [sn1] | 2.20 | 1.64 | 0.56 | 1.07 |
| pink | LPC(20:3) [sn1] | 2.33 | 1.51 | 0.82 | 0.69 |
| pink | LPE(20:4) [sn1] | 1.85 | 1.35 | 0.50 | 0.84 |
| pink | LPC(22:4) [sn1] | 1.37 | 1.19 | 0.19 | 1.00 |
| pink | LPE(22:6) [sn1] | 1.32 | 1.17 | 0.15 | 1.01 |
| pink | LPE(20:4) [sn2] | 1.57 | 1.16 | 0.41 | 0.75 |
| pink | LPC(20:3) [sn2] | 1.62 | 1.14 | 0.48 | 0.65 |
| pink | LPC(22:6) [sn1] | 1.12 | 1.00 | 0.12 | 0.88 |
| pink | LPC(22:5) (n3) [sn1] [104_sn1] | 0.98 | 0.91 | 0.07 | 0.84 |
| pink | LPC(22:6) [sn2] | 0.99 | 0.89 | 0.10 | 0.79 |
| pink | LPC(22:4) [sn2] | 0.98 | 0.87 | 0.10 | 0.77 |
| pink | LPE(22:6) [sn2] | 0.90 | 0.85 | 0.05 | 0.80 |
| pink | LPC(22:5) [sn2] (n3) | 0.95 | 0.83 | 0.11 | 0.72 |
| pink | LPC(22:5) [sn1] (n3)/LPC(22:5) [sn2] (n6) | 0.84 | 0.69 | 0.14 | 0.55 |
| pink | LPC(22:5) [sn1] (n6) | 0.73 | 0.66 | 0.08 | 0.58 |
| pink | CE(22:5) (n6) | 0.30 | 0.23 | 0.07 | 0.16 |
| pink | LPI(20:4) [sn1] | 0.20 | 0.19 | 0.02 | 0.17 |
| pink | PC(18:0_22:5) (n6) | 0.38 | 0.19 | 0.19 | -0.01 |
| pink | LPI(20:4) [sn2] | 0.17 | 0.16 | 0.01 | 0.15 |
| pink | SM(d18:0/22:0) | 0.10 | 0.04 | 0.06 | -0.03 |
| **purple (hub)** | **FA(18:1)** | **1.60** | **1.47** | **0.12** | **1.35** |
| purple | FA(18:2) | 1.36 | 1.33 | 0.03 | 1.30 |
| purple | FA(16:0) | 1.31 | 1.22 | 0.09 | 1.13 |
| purple | FA(18:3) | 1.11 | 1.10 | 0.01 | 1.09 |
| purple | FA(16:1) | 1.08 | 1.03 | 0.06 | 0.97 |
| purple | FA(22:4) | 0.76 | 0.73 | 0.04 | 0.69 |
| purple | FA(22:5) | 0.67 | 0.66 | 0.01 | 0.64 |
| purple | FA(20:3) | 0.71 | 0.60 | 0.11 | 0.48 |
| purple | FA(14:0) | 0.39 | 0.39 | 0.00 | 0.38 |
| purple | FA(22:6) | 0.34 | 0.31 | 0.02 | 0.29 |
| purple | FA(20:4) | 0.29 | 0.27 | 0.02 | 0.25 |
| purple | FA(17:1) | 0.10 | 0.10 | 0.00 | 0.09 |
| purple | FA(20:2) | 0.09 | 0.08 | 0.01 | 0.07 |
| purple | FA(17:0) | 0.05 | 0.04 | 0.00 | 0.04 |
| purple | FA(20:5) | 0.05 | 0.04 | 0.02 | 0.02 |
| purple | FA(15:0) | 0.02 | 0.01 | 0.00 | 0.01 |
| **red (hub)** | **CE(24:1)** | **2.13** | **1.91** | **0.23** | **1.68** |
| red | CE(22:1) | 1.82 | 1.69 | 0.13 | 1.55 |
| red | CE(20:1) | 1.85 | 1.52 | 0.33 | 1.20 |
| red | CE(24:0) | 1.45 | 1.43 | 0.02 | 1.41 |
| red | CE(22:0) | 1.21 | 1.18 | 0.02 | 1.16 |
| red | CE(20:0) | 1.12 | 1.09 | 0.02 | 1.07 |
| red | CE(20:2) | 1.59 | 0.90 | 0.69 | 0.21 |
| red | CE(18:0) | 1.03 | 0.84 | 0.19 | 0.65 |
| red | CE(17:1) | 0.85 | 0.55 | 0.30 | 0.25 |
| red | CE(24:4) | 0.54 | 0.48 | 0.06 | 0.42 |
| red | CE(22:4) | 0.91 | 0.43 | 0.49 | -0.06 |
| red | CE(17:0) | 0.77 | 0.38 | 0.38 | 0.00 |
| red | CE(16:1) | 0.57 | 0.36 | 0.21 | 0.15 |
| red | CE(24:5) | 0.28 | 0.24 | 0.03 | 0.21 |
| red | CE(24:6) | 0.28 | 0.21 | 0.07 | 0.14 |
| red | CE(20:3) | 0.42 | 0.20 | 0.21 | -0.01 |
| red | DE(18:1) | 0.25 | 0.20 | 0.05 | 0.15 |
| red | CE(15:0) | 0.26 | 0.17 | 0.09 | 0.08 |
| red | CE(18:3) | 0.54 | 0.14 | 0.40 | -0.25 |
| red | CE(14:0) | 0.16 | 0.12 | 0.04 | 0.09 |
| red | PC(18:0_20:3) | 0.30 | 0.08 | 0.22 | -0.14 |
| red | CE(20:4) [+OH] | 0.15 | 0.05 | 0.10 | -0.05 |
| red | DE(18:2) | 0.07 | 0.04 | 0.03 | 0.01 |
| **salmon (hub)** | **HexCer(d18:1/20:0)** | **1.03** | **1.01** | **0.02** | **0.99** |
| salmon | HexCer(d18:2/22:0) | 0.88 | 0.86 | 0.02 | 0.85 |
| salmon | HexCer(d18:2/24:0) | 0.84 | 0.83 | 0.01 | 0.82 |
| salmon | HexCer(d18:1/18:0) | 0.82 | 0.79 | 0.03 | 0.75 |
| salmon | HexCer(d18:1/24:1) | 0.68 | 0.66 | 0.02 | 0.63 |
| salmon | HexCer(d18:1/24:0) | 0.65 | 0.64 | 0.01 | 0.63 |
| salmon | HexCer(d18:1/22:0) | 0.99 | 0.57 | 0.42 | 0.15 |
| salmon | HexCer(d16:1/20:0) | 0.47 | 0.43 | 0.04 | 0.39 |
| salmon | HexCer(d16:1/24:0) | 0.44 | 0.38 | 0.06 | 0.32 |
| salmon | HexCer(d16:1/22:0) | 0.43 | 0.26 | 0.16 | 0.10 |
| salmon | HexCer(d16:1/18:0) | 0.22 | 0.22 | 0.00 | 0.22 |
| salmon | HexCer(d18:1/16:0) | 0.20 | 0.20 | 0.00 | 0.20 |
| salmon | HexCer(d18:2/18:0) | 0.23 | 0.20 | 0.04 | 0.16 |
| salmon | HexCer(d18:2/20:0) | 0.16 | 0.14 | 0.02 | 0.11 |
| tan | LPC(17:1) [sn1] (a) / LPC(17:1) [sn2] (b) | 2.84 | 2.46 | 0.38 | 2.08 |
| tan | LPC(17:1) [sn2] (a) | 2.54 | 2.16 | 0.38 | 1.78 |
| tan | LPC(16:1) [sn1] | 2.99 | 2.02 | 0.97 | 1.05 |
| tan | LPC(17:1) (a) [sn1] [104_sn1] | 2.32 | 2.02 | 0.31 | 1.71 |
| tan | LPC(15:0) [sn2] | 2.19 | 1.79 | 0.40 | 1.39 |
| tan | LPC(14:0) [sn2] | 2.08 | 1.70 | 0.38 | 1.32 |
| tan | LPC(16:1) [sn2] | 2.40 | 1.69 | 0.70 | 0.99 |
| tan | LPC(14:0) [sn1] | 1.97 | 1.68 | 0.29 | 1.39 |
| tan | LPC(15:0) [sn1] | 1.90 | 1.36 | 0.54 | 0.82 |
| tan | S1P(d18:1) | 1.21 | 0.91 | 0.29 | 0.62 |
| tan | S1P(d18:2) | 1.11 | 0.79 | 0.33 | 0.46 |
| tan | LPC(18:3) [sn1] (b) | 1.96 | 0.68 | 1.28 | -0.60 |
| tan | S1P(d18:0) | 0.73 | 0.63 | 0.09 | 0.54 |
| tan | LPC(17:1) [sn1] (b) | 0.26 | 0.13 | 0.12 | 0.01 |
| tan | S1P(d16:1) | 0.09 | 0.08 | 0.01 | 0.08 |
| **turquoise (hub)** | **PE(18:0_18:2)** | **13.89** | **12.27** | **1.62** | **10.64** |
| turquoise | PE(P-18:0/18:2) | 12.99 | 11.99 | 1.00 | 10.99 |
| turquoise | PC(16:0_18:3) (a) | 14.30 | 11.49 | 2.81 | 8.68 |
| turquoise | PE(18:1_18:2) | 11.95 | 11.25 | 0.71 | 10.54 |
| turquoise | PE(16:0_18:2) | 12.30 | 11.19 | 1.11 | 10.07 |
| turquoise | PC(P-18:0/18:2) | 17.13 | 10.87 | 6.26 | 4.61 |
| turquoise | PC(18:2_18:2) | 12.77 | 10.37 | 2.39 | 7.98 |
| turquoise | PI(18:0_18:1) | 11.82 | 10.17 | 1.64 | 8.53 |
| turquoise | PE(38:5) (a) | 11.03 | 10.12 | 0.92 | 9.20 |
| turquoise | PC(33:2) | 14.02 | 10.10 | 3.93 | 6.17 |
| turquoise | PE(P-18:1/18:2) (a) | 10.80 | 9.91 | 0.89 | 9.02 |
| turquoise | PE(18:1_18:1) | 10.78 | 9.53 | 1.24 | 8.29 |
| turquoise | PE(P-16:0/18:2) | 10.64 | 9.45 | 1.19 | 8.26 |
| turquoise | PE(16:0_20:3) | 10.14 | 9.34 | 0.80 | 8.55 |
| turquoise | PC(P-35:2)(a) | 12.77 | 9.19 | 3.58 | 5.61 |
| turquoise | PE(18:0_18:1) | 10.75 | 9.11 | 1.63 | 7.48 |
| turquoise | PE(P-18:1/20:4) (b) | 10.02 | 9.10 | 0.91 | 8.19 |
| turquoise | PE(17:0_18:2) | 10.76 | 8.99 | 1.77 | 7.21 |
| turquoise | PE(16:0_18:1) | 10.28 | 8.87 | 1.41 | 7.46 |
| turquoise | PC(O-18:0/18:2) | 13.94 | 8.76 | 5.18 | 3.58 |
| turquoise | PE(P-18:1/18:1) (b) | 9.58 | 8.71 | 0.87 | 7.84 |
| turquoise | PC(O-18:1/18:2) | 16.39 | 8.70 | 7.70 | 1.00 |
| turquoise | PE(P-18:1/18:1) | 9.46 | 8.56 | 0.90 | 7.65 |
| turquoise | PE(P-16:0/22:5) (n3) | 9.58 | 8.52 | 1.06 | 7.46 |
| turquoise | PC(P-16:0/18:3) | 11.44 | 8.51 | 2.93 | 5.58 |
| turquoise | PE(P-18:0/22:5) (n3) | 9.35 | 8.41 | 0.94 | 7.47 |
| turquoise | PC(32:2) | 12.33 | 8.30 | 4.03 | 4.28 |
| turquoise | PE(17:0_18:1) | 9.35 | 8.13 | 1.23 | 6.90 |
| turquoise | PE(P-18:0/18:1) | 9.86 | 8.08 | 1.78 | 6.29 |
| turquoise | PE(P-20:0/18:2) | 8.86 | 8.06 | 0.80 | 7.26 |
| turquoise | PI(34:1) | 9.70 | 7.91 | 1.80 | 6.11 |
| turquoise | PC(16:1_18:2) | 10.73 | 7.79 | 2.94 | 4.85 |
| turquoise | PE(P-18:1/22:5) (a) | 8.42 | 7.59 | 0.83 | 6.76 |
| turquoise | PE(18:1_22:6) (a) | 8.57 | 7.54 | 1.03 | 6.51 |
| turquoise | PC(17:1_18:2) | 10.70 | 7.53 | 3.16 | 4.37 |
| turquoise | PC(O-34:2) | 11.93 | 7.48 | 4.45 | 3.02 |
| turquoise | PE(P-18:1/18:1) (a) | 8.18 | 7.37 | 0.81 | 6.55 |
| turquoise | PE(P-17:0/20:4) (b) | 8.36 | 7.33 | 1.03 | 6.31 |
| turquoise | PC(38:6) (a) | 12.27 | 6.99 | 5.27 | 1.72 |
| turquoise | PC(P-16:0/18:2) | 14.06 | 6.99 | 7.07 | -0.08 |
| turquoise | PE(O-34:1) | 7.80 | 6.96 | 0.84 | 6.11 |
| turquoise | PI(36:2) | 8.74 | 6.92 | 1.82 | 5.10 |
| turquoise | PE(P-17:0/20:4) (a) | 8.52 | 6.91 | 1.60 | 5.31 |
| turquoise | PE(18:1_22:6) (b) | 9.01 | 6.91 | 2.10 | 4.80 |
| turquoise | PE(P-18:0/22:6) | 8.50 | 6.89 | 1.61 | 5.28 |
| turquoise | PE(P-18:0/20:4) | 7.69 | 6.86 | 0.84 | 6.02 |
| turquoise | PE(P-18:1/20:4) (a) | 7.78 | 6.76 | 1.03 | 5.73 |
| turquoise | PC(18:0_18:2) | 10.93 | 6.73 | 4.20 | 2.53 |
| turquoise | Cer(d18:1/23:0) | 8.36 | 6.69 | 1.67 | 5.01 |
| turquoise | PE(P-18:1/18:2) (b) | 7.13 | 6.56 | 0.57 | 5.99 |
| turquoise | PE(P-18:0/20:3) (a) | 7.06 | 6.53 | 0.53 | 6.00 |
| turquoise | PE(P-18:1/22:6) (b) | 7.72 | 6.34 | 1.38 | 4.95 |
| turquoise | Cer(d18:1/22:0) | 7.21 | 6.27 | 0.94 | 5.32 |
| turquoise | PC(18:1_18:2) | 9.15 | 6.20 | 2.94 | 3.26 |
| turquoise | PE(O-16:0/18:2) | 6.82 | 6.12 | 0.69 | 5.43 |
| turquoise | PE(P-18:0/20:3) (b) | 6.61 | 6.06 | 0.55 | 5.51 |
| turquoise | PE(18:0_20:3) (a) | 7.50 | 6.03 | 1.46 | 4.57 |
| turquoise | PE(P-18:0/20:5) | 6.46 | 6.03 | 0.44 | 5.59 |
| turquoise | PC(16:0_18:2) | 9.90 | 6.00 | 3.90 | 2.10 |
| turquoise | PC(14:0_20:4) | 11.12 | 5.95 | 5.16 | 0.79 |
| turquoise | PE(16:0_18:3) (a) | 6.82 | 5.86 | 0.95 | 4.91 |
| turquoise | PE(O-38:5) (a) | 6.89 | 5.70 | 1.19 | 4.51 |
| turquoise | PE(16:0_20:4) | 6.13 | 5.61 | 0.52 | 5.09 |
| turquoise | PE(15-MHDA_18:2) | 6.21 | 5.59 | 0.61 | 4.98 |
| turquoise | PE(P-17:0/22:6) (b) | 7.97 | 5.57 | 2.40 | 3.16 |
| turquoise | Cer(d18:1/16:0) | 6.26 | 5.38 | 0.88 | 4.50 |
| turquoise | PC(15-MHDA_18:2) | 7.92 | 5.25 | 2.67 | 2.58 |
| turquoise | Cer(d18:1/20:0) | 5.76 | 5.16 | 0.61 | 4.55 |
| turquoise | Cer(d16:1/22:0) | 6.08 | 5.09 | 1.00 | 4.09 |
| turquoise | PC(15:0_20:3) | 7.87 | 5.04 | 2.83 | 2.22 |
| turquoise | PC(17:0_18:2) | 7.89 | 4.97 | 2.92 | 2.05 |
| turquoise | PC(18:2_20:5) | 6.67 | 4.90 | 1.77 | 3.13 |
| turquoise | Cer(m18:0/24:1) | 5.74 | 4.89 | 0.85 | 4.04 |
| turquoise | Cer(m18:0/23:0) | 5.89 | 4.89 | 1.00 | 3.89 |
| turquoise | PE(P-20:0/18:1) | 5.41 | 4.87 | 0.54 | 4.33 |
| turquoise | PE(17:0_20:4) | 6.61 | 4.83 | 1.78 | 3.05 |
| turquoise | PC(34:5) | 6.27 | 4.80 | 1.48 | 3.32 |
| turquoise | PE(P-16:0/20:4) | 5.41 | 4.78 | 0.63 | 4.15 |
| turquoise | CE(18:2) | 8.92 | 4.74 | 4.18 | 0.56 |
| turquoise | Cer(m18:0/22:0) | 5.69 | 4.68 | 1.01 | 3.68 |
| turquoise | PE(18:0_22:5) (n3) | 7.28 | 4.68 | 2.60 | 2.09 |
| turquoise | PE(P-18:1/20:3) (b) | 5.05 | 4.61 | 0.44 | 4.16 |
| turquoise | PC(18:1_18:1) | 8.96 | 4.57 | 4.39 | 0.18 |
| turquoise | PE(P-16:0/20:5) | 4.82 | 4.55 | 0.27 | 4.28 |
| turquoise | PC(P-35:2)(b) | 6.06 | 4.50 | 1.56 | 2.93 |
| turquoise | PE(P-16:0/18:1) | 5.29 | 4.46 | 0.83 | 3.63 |
| turquoise | PE(18:0_20:4) | 5.81 | 4.35 | 1.46 | 2.89 |
| turquoise | PE(16:1_18:2) | 4.49 | 4.27 | 0.22 | 4.05 |
| turquoise | PI(34:0) | 5.75 | 4.22 | 1.53 | 2.69 |
| turquoise | Cer(d18:1/24:1) | 5.11 | 4.18 | 0.93 | 3.26 |
| turquoise | PE(P-18:1/20:3) (a) | 4.65 | 4.16 | 0.50 | 3.66 |
| turquoise | Cer(d17:1/22:0) | 4.91 | 4.14 | 0.77 | 3.37 |
| turquoise | Cer(d16:1/23:0) | 5.09 | 4.11 | 0.98 | 3.13 |
| turquoise | Cer(d17:1/24:1) | 5.09 | 4.08 | 1.00 | 3.08 |
| turquoise | PE(P-18:1/20:5) (a) | 4.32 | 4.03 | 0.28 | 3.75 |
| turquoise | PE(O-18:1/18:2) | 4.49 | 4.01 | 0.48 | 3.53 |
| turquoise | PE(P-16:0/20:3) (a) | 4.41 | 3.92 | 0.50 | 3.42 |
| turquoise | Cer(d17:1/24:0) | 5.17 | 3.92 | 1.25 | 2.67 |
| turquoise | PI(17:0_18:2) | 5.71 | 3.83 | 1.88 | 1.96 |
| turquoise | PC(P-15:0/20:4) (a) | 5.76 | 3.83 | 1.93 | 1.90 |
| turquoise | Cer(d18:1/18:0) | 4.14 | 3.82 | 0.32 | 3.50 |
| turquoise | PE(16:0_16:1) | 4.04 | 3.78 | 0.26 | 3.52 |
| turquoise | PE(P-18:1/22:6) (a) | 6.17 | 3.76 | 2.41 | 1.34 |
| turquoise | PE(P-16:0/20:3) (b) | 4.12 | 3.75 | 0.37 | 3.38 |
| turquoise | PE(15-MHDA_18:1) | 4.19 | 3.64 | 0.55 | 3.09 |
| turquoise | PE(16:0_18:3) (b) | 3.82 | 3.61 | 0.21 | 3.40 |
| turquoise | Cer(d17:1/23:0) | 4.37 | 3.59 | 0.78 | 2.81 |
| turquoise | Cer(d18:1/24:0) | 4.91 | 3.51 | 1.40 | 2.11 |
| turquoise | PE(P-16:0/22:6) | 6.08 | 3.45 | 2.63 | 0.83 |
| turquoise | Cer(d16:1/24:0) | 4.28 | 3.39 | 0.89 | 2.50 |
| turquoise | PC(35:5) | 4.25 | 3.27 | 0.98 | 2.30 |
| turquoise | Cer(m18:0/24:0) | 4.07 | 3.22 | 0.85 | 2.37 |
| turquoise | Cer(d18:2/23:0) | 4.39 | 3.20 | 1.19 | 2.02 |
| turquoise | PC(28:0) | 6.66 | 3.19 | 3.46 | -0.27 |
| turquoise | PE(P-20:1/20:4) | 3.45 | 3.16 | 0.29 | 2.88 |
| turquoise | PE(P-17:0/22:6) (a) | 5.49 | 2.99 | 2.50 | 0.49 |
| turquoise | PE(16:0_16:0) | 3.71 | 2.96 | 0.75 | 2.21 |
| turquoise | PE(P-20:1/22:6) | 4.20 | 2.89 | 1.32 | 1.57 |
| turquoise | Cer(d16:1/24:1) | 3.44 | 2.87 | 0.57 | 2.30 |
| turquoise | PC(O-36:5) | 4.83 | 2.87 | 1.96 | 0.90 |
| turquoise | PE(38:5) (b) | 3.89 | 2.85 | 1.04 | 1.81 |
| turquoise | PE(16:0_20:5) | 3.00 | 2.84 | 0.16 | 2.69 |
| turquoise | PC(36:6) (a) | 3.51 | 2.84 | 0.67 | 2.16 |
| turquoise | PC(P-16:0/20:5) | 4.26 | 2.75 | 1.51 | 1.24 |
| turquoise | PE(15-MHDA_20:4) | 3.56 | 2.69 | 0.87 | 1.81 |
| turquoise | Cer(d18:2/16:0) | 3.93 | 2.66 | 1.27 | 1.39 |
| turquoise | Cer(d18:1/21:0) | 3.04 | 2.60 | 0.45 | 2.15 |
| turquoise | PE(O-38:5) (b) | 3.30 | 2.59 | 0.71 | 1.88 |
| turquoise | PI(16:0/16:0) | 4.21 | 2.55 | 1.66 | 0.88 |
| turquoise | Cer(d16:1/20:0) | 2.83 | 2.44 | 0.38 | 2.06 |
| turquoise | PC(16:0_20:5) | 2.98 | 2.44 | 0.55 | 1.89 |
| turquoise | PC(16:0_18:3) (b) | 4.73 | 2.43 | 2.30 | 0.13 |
| turquoise | Cer(m18:1/24:1) | 2.86 | 2.42 | 0.44 | 1.99 |
| turquoise | PE(P-20:0/22:6) | 3.42 | 2.40 | 1.02 | 1.38 |
| turquoise | Cer(d18:2/22:0) | 3.27 | 2.35 | 0.92 | 1.43 |
| turquoise | PE(P-16:0/18:3) | 2.44 | 2.29 | 0.15 | 2.14 |
| turquoise | PC(18:1_20:3) | 5.00 | 2.20 | 2.80 | -0.59 |
| turquoise | PE(18:0_22:4) | 3.27 | 2.20 | 1.07 | 1.13 |
| turquoise | PE(O-18:0/22:5) | 2.84 | 2.16 | 0.68 | 1.47 |
| turquoise | Cer(m18:0/20:0) | 2.58 | 2.14 | 0.44 | 1.71 |
| turquoise | PE(O-18:0/20:4) | 2.50 | 2.12 | 0.38 | 1.74 |
| turquoise | Cer(d16:1/18:0) | 2.36 | 2.11 | 0.26 | 1.85 |
| turquoise | PE(O-18:0/22:6) | 2.61 | 2.06 | 0.55 | 1.51 |
| turquoise | Cer(d16:1/16:0) | 2.34 | 2.06 | 0.28 | 1.79 |
| turquoise | Cer(d19:1/23:0) | 2.98 | 1.98 | 1.00 | 0.98 |
| turquoise | Cer(d18:2/24:1) | 3.12 | 1.96 | 1.15 | 0.81 |
| turquoise | Cer(m18:1/22:0) | 2.16 | 1.92 | 0.25 | 1.67 |
| turquoise | Hex2Cer(d18:1/16:0) | 3.16 | 1.88 | 1.27 | 0.61 |
| turquoise | PC(16:1_20:4) | 5.67 | 1.88 | 3.80 | -1.92 |
| turquoise | PE(16:1_20:4) | 1.92 | 1.85 | 0.07 | 1.78 |
| turquoise | PE(O-16:0/20:4) | 2.16 | 1.83 | 0.33 | 1.50 |
| turquoise | PI(18:1_18:2) | 1.96 | 1.75 | 0.21 | 1.54 |
| turquoise | Cer(d18:2/20:0) | 2.11 | 1.73 | 0.38 | 1.35 |
| turquoise | Ubiquinone | 2.18 | 1.73 | 0.45 | 1.28 |
| turquoise | Cer(m18:1/23:0) | 2.14 | 1.71 | 0.43 | 1.28 |
| turquoise | PC(16:0_20:3) (a) | 4.15 | 1.70 | 2.45 | -0.75 |
| turquoise | PI(17:0_18:1) | 2.58 | 1.69 | 0.89 | 0.80 |
| turquoise | Hex2Cer(d16:1/16:0) | 2.21 | 1.65 | 0.56 | 1.09 |
| turquoise | Cer(d18:2/24:0) | 2.56 | 1.64 | 0.93 | 0.71 |
| turquoise | Cer(m18:1/24:0) | 1.84 | 1.61 | 0.22 | 1.39 |
| turquoise | Cer(d18:2/18:0) | 1.80 | 1.58 | 0.22 | 1.36 |
| turquoise | PE(O-18:1/22:6) | 2.01 | 1.57 | 0.44 | 1.13 |
| turquoise | PE(O-16:0/22:6) | 1.92 | 1.48 | 0.44 | 1.03 |
| turquoise | PE(O-36:5) | 1.71 | 1.46 | 0.24 | 1.22 |
| turquoise | PE(P-18:0/18:3) | 1.55 | 1.45 | 0.10 | 1.35 |
| turquoise | Cer(m18:1/20:0) | 1.49 | 1.37 | 0.12 | 1.25 |
| turquoise | Cer(d17:1/20:0) | 1.51 | 1.33 | 0.18 | 1.16 |
| turquoise | PC(O-36:0) | 2.06 | 1.33 | 0.73 | 0.60 |
| turquoise | PE(O-16:0/20:3) | 1.47 | 1.26 | 0.21 | 1.04 |
| turquoise | PE(18:0_22:5) (n6) | 1.96 | 1.19 | 0.77 | 0.42 |
| turquoise | PE(P-18:0/22:5) (n6) | 1.38 | 1.17 | 0.21 | 0.96 |
| turquoise | PC(32:1) | 2.90 | 1.14 | 1.76 | -0.62 |
| turquoise | PE(P-20:0/20:4) | 1.27 | 1.07 | 0.20 | 0.87 |
| turquoise | Hex2Cer(d18:1/24:1) | 1.27 | 1.07 | 0.21 | 0.86 |
| turquoise | Cer(m18:1/18:0) | 1.16 | 1.03 | 0.13 | 0.90 |
| turquoise | PI(16:0_20:3) (a) | 1.14 | 1.01 | 0.14 | 0.87 |
| turquoise | PE(P-19:0/20:4) (a) | 1.26 | 0.95 | 0.31 | 0.64 |
| turquoise | Hex2Cer(d18:1/22:0) | 1.13 | 0.94 | 0.19 | 0.75 |
| turquoise | PI(16:0_16:1) | 1.31 | 0.86 | 0.46 | 0.40 |
| turquoise | Cer(d19:1/22:0) | 1.58 | 0.81 | 0.77 | 0.04 |
| turquoise | PE(P-18:1/22:5) (b) | 1.04 | 0.79 | 0.25 | 0.54 |
| turquoise | PE(P-18:1/20:5) (b) | 0.80 | 0.75 | 0.05 | 0.70 |
| turquoise | PE(P-19:0/20:4) (b) | 0.91 | 0.74 | 0.17 | 0.56 |
| turquoise | PE(O-16:0/22:4) | 0.87 | 0.71 | 0.16 | 0.55 |
| turquoise | PC(38:2) | 3.00 | 0.70 | 2.30 | -1.60 |
| turquoise | PI(18:0_20:2) | 0.80 | 0.70 | 0.10 | 0.59 |
| turquoise | Cer1P(d18:1/16:0) | 1.13 | 0.67 | 0.46 | 0.22 |
| turquoise | Cer(d17:1/16:0) | 0.75 | 0.66 | 0.08 | 0.58 |
| turquoise | Cer(d17:1/18:0) | 0.76 | 0.66 | 0.11 | 0.55 |
| turquoise | PE(P-16:0/22:5) (n6) | 0.77 | 0.63 | 0.14 | 0.49 |
| turquoise | PI(18:0_22:6) | 1.45 | 0.61 | 0.84 | -0.22 |
| turquoise | Cer(d18:0/16:0) | 0.79 | 0.61 | 0.19 | 0.42 |
| turquoise | Cer(d18:1/14:0) | 0.57 | 0.51 | 0.06 | 0.46 |
| turquoise | Cer(d18:1/19:0) | 0.52 | 0.43 | 0.09 | 0.34 |
| turquoise | Cer(d18:2/21:0) | 0.55 | 0.42 | 0.14 | 0.28 |
| turquoise | Cer(d19:1/18:0) | 0.51 | 0.41 | 0.10 | 0.31 |
| turquoise | PI (38:5) (b) | 0.51 | 0.38 | 0.14 | 0.24 |
| turquoise | Hex2Cer(d18:1/24:0) | 0.45 | 0.36 | 0.09 | 0.28 |
| turquoise | PE(P-15:0/20:4) (a) | 0.40 | 0.36 | 0.03 | 0.33 |
| turquoise | PE(P-15:0/20:4) (b) | 0.37 | 0.33 | 0.04 | 0.29 |
| turquoise | PE(P-15:0/22:6) (a) | 0.37 | 0.32 | 0.05 | 0.27 |
| turquoise | Cer(d18:2/14:0) | 0.37 | 0.30 | 0.06 | 0.24 |
| turquoise | PI(18:0_22:4) | 0.36 | 0.29 | 0.08 | 0.21 |
| turquoise | PI(37:6) | 0.43 | 0.27 | 0.16 | 0.11 |
| turquoise | PE(18:0_20:3) (b) | 0.37 | 0.23 | 0.14 | 0.09 |
| turquoise | Sulfatide (d18:1:/16:0(OH)) | 0.29 | 0.22 | 0.07 | 0.14 |
| turquoise | Cer(d19:1/16:0) | 0.21 | 0.19 | 0.02 | 0.17 |
| turquoise | PE(P-18:1/18:3) | 0.17 | 0.16 | 0.01 | 0.15 |
| turquoise | PC(16:0_20:3) (b) | 0.69 | 0.14 | 0.56 | -0.42 |
| turquoise | Cer(d19:1/20:0) | 0.16 | 0.13 | 0.03 | 0.10 |
| turquoise | Cer(d18:0/18:0) | 0.16 | 0.10 | 0.06 | 0.04 |
| turquoise | Hex2Cer(d18:1/20:0) | 0.09 | 0.08 | 0.01 | 0.07 |
| turquoise | PE(P-15:0/22:6) (b) | 0.09 | 0.07 | 0.02 | 0.06 |
| turquoise | Cer(d20:1/23:0) | 0.10 | 0.07 | 0.03 | 0.04 |
| turquoise | Cer(d20:1/22:0) | 0.12 | 0.06 | 0.06 | 0.00 |
| turquoise | Cer(d18:2/26:0) | 0.09 | 0.06 | 0.03 | 0.03 |
| turquoise | TG(O-54:4) [NL-17:1] | 0.04 | 0.02 | 0.02 | 0.00 |
| **yellow (hub)** | **TG(56:8) [SIM]** | **12.85** | **11.81** | **1.03** | **10.78** |
| yellow | TG(56:7) [SIM] | 13.37 | 11.69 | 1.67 | 10.02 |
| yellow | TG(56:8) [NL-22:6] | 10.43 | 9.99 | 0.44 | 9.55 |
| yellow | TG(54:7) [SIM] | 11.72 | 9.90 | 1.82 | 8.08 |
| yellow | TG(58:9) [SIM] | 10.11 | 9.50 | 0.61 | 8.89 |
| yellow | TG(58:8) [SIM] | 9.77 | 9.22 | 0.55 | 8.67 |
| yellow | TG(58:10) [NL-22:6] | 9.40 | 9.11 | 0.29 | 8.81 |
| yellow | TG(56:9) [SIM] | 9.73 | 9.10 | 0.63 | 8.47 |
| yellow | TG(54:6) [SIM] | 14.01 | 8.97 | 5.03 | 3.94 |
| yellow | TG(56:9) [NL-22:6] | 9.12 | 8.69 | 0.43 | 8.26 |
| yellow | TG(56:6) [SIM] | 12.03 | 8.66 | 3.37 | 5.28 |
| yellow | TG(58:9) [NL-22:6] | 9.16 | 8.65 | 0.51 | 8.14 |
| yellow | TG(54:7) [NL-20:5] | 10.29 | 8.21 | 2.08 | 6.13 |
| yellow | TG(54:7) [NL-22:6] | 8.26 | 7.80 | 0.47 | 7.33 |
| yellow | TG(56:7) [NL-22:6] | 8.01 | 7.72 | 0.29 | 7.43 |
| yellow | TG(54:6) [NL-20:5] | 10.04 | 7.58 | 2.46 | 5.13 |
| yellow | TG(58:10) [SIM] | 7.70 | 7.51 | 0.19 | 7.32 |
| yellow | TG(54:6) [NL-20:4] | 10.00 | 7.09 | 2.91 | 4.18 |
| yellow | TG(56:7) [NL-22:5](b) | 9.48 | 6.90 | 2.57 | 4.33 |
| yellow | TG(56:7) [NL-22:5](a) | 9.44 | 6.88 | 2.56 | 4.31 |
| yellow | TG(56:8) [NL-20:5] | 7.98 | 6.67 | 1.32 | 5.35 |
| yellow | TG(52:5) [NL-20:5] | 7.58 | 6.10 | 1.48 | 4.62 |
| yellow | TG(54:5) [NL-20:4] | 8.49 | 6.00 | 2.49 | 3.51 |
| yellow | TG(52:5) [NL-20:4] | 8.43 | 5.48 | 2.95 | 2.53 |
| yellow | TG(54:5) [SIM] | 14.93 | 5.16 | 9.77 | -4.61 |
| yellow | TG(56:8) [NL-20:4] | 5.99 | 5.07 | 0.92 | 4.14 |
| yellow | TG(54:6) [NL-22:6] | 5.10 | 4.98 | 0.12 | 4.87 |
| yellow | TG(56:7) [NL-20:4] | 6.98 | 4.91 | 2.07 | 2.83 |
| yellow | TG(56:6) [NL-22:5](b) | 6.31 | 4.72 | 1.59 | 3.13 |
| yellow | TG(56:7) [NL-20:5] | 6.46 | 4.70 | 1.76 | 2.93 |
| yellow | TG(58:8) [NL-22:6] | 4.82 | 4.66 | 0.16 | 4.50 |
| yellow | TG(56:6) [NL-22:5](a) | 6.10 | 4.57 | 1.52 | 3.05 |
| yellow | TG(50:4) [NL-20:4] | 8.35 | 4.54 | 3.81 | 0.73 |
| yellow | DG(18:2_22:6) | 4.40 | 4.03 | 0.37 | 3.66 |
| yellow | DG(18:1_22:6) | 3.64 | 3.41 | 0.23 | 3.18 |
| yellow | DG(16:0_22:6) | 3.66 | 3.16 | 0.50 | 2.66 |
| yellow | DG(18:1_20:5) | 3.87 | 2.81 | 1.06 | 1.76 |
| yellow | TG(56:6) [NL-20:4] | 2.87 | 2.28 | 0.59 | 1.69 |
| yellow | DG(18:2_20:4) | 4.43 | 1.74 | 2.69 | -0.95 |
| yellow | PC(18:0_22:6) | 0.65 | 0.33 | 0.32 | 0.01 |

**Note:** k, connectivity measure. They are calculated with the topological overlay matrix, which is calculated as part of the WGCNA analysis and represents the combination of weighted connection strengths between lipids.

kTotal, connectivity value of each lipid to other lipid concentrations in the whole network; kWithin, connectivity value of each lipid concentration within a single module to all other lipid concentrations within the same module; kOut, kTotal-kWithin; kDiff, kIn-kOut=2*kIn-kTotal^3^

***Supplementary Table S3:* The association between the cord blood lipid module eigenlipids and subsequent CBCL-ADHP and CBCL-ASP outcomes at 2 years.**

| **Cord blood lipid profile** | **CBCL-ADHP^1^** | | | | | |  | **CBCL-ASP^1^** | | | | | |
| --- | --- | --- | --- | --- | --- | --- | --- | --- | --- | --- | --- | --- | --- |
| **Single eigenlipid model (hub lipid's class)** | **AMD (95% CI)** | ***P-*value** | ***Q-*value** | **Adjusted Alpha** | **R^2^** | **AIC** |  | **ADM (95% CI)** | ***P*-value** | ***Q*-value** | **Adjusted Alpha** | **R^2^** | **AIC** |
| Cyan-AC | **0·54 (0·33, 0·75)** | **<0·0001** | **<0·0001** | **0.003** | 0·07 | 2550.30 |  | **0·38 (0·19, 0·57)** | **<0·0001** | **0·001** | **0.003** | 0·04 | 2306.734 |
| Turquoise-PE(hub) | **0·41 (0·16, 0·66)** | **<0·0001** | **0·009** | **0.007** | 0·05 | 2562.94 |  | **0·24 (0·05, 0·42)** | **0·01** | 0·08 | 0.007 | 0·01 | 2319.957 |
| Brown-TG(hub) | **0·32 (0·10, 0·53)** | **<0·0001** | **0·02** | **0.010** | 0·04 | 2568.00 |  | **0·21 (0·02, 0·39)** | **0·03** | 0·10 | 0.010 | 0·01 | 2321.328 |
| Magenta-PS(hub) | **0·29 (0·07, 0·51)** | **0·009** | **0·02** | **0.013** | 0·04 | 2568.70 |  | **0·20 (0·04, 0·36)** | **0·02** | 0·08 | 0.013 | 0·01 | 2321.385 |
| Blue-SMD(hub) | **0·34 (0·13, 0·56)** | **<0·0001** | **0·009** | **0.017** | 0·04 | 2565.69 |  | 0·08 (-0·11, 0·26) | 0·42 | 0·63 | 0.017 | 0·003 | 2325.961 |
| Red-CE(hub) | **0·24 (0·02, 0·45)** | **0·04** | **0·06** | **0.020** | 0·03 | 2571.34 |  | 0·04 (-0·17, 0·25) | 0·71 | 0·81 | 0.020 | 0·002 | 2326.533 |
| Green-LPC(hub) | **0·27 (0·07, 0·48)** | **0·01** | **0·02** | **0.023** | 0·04 | 2569.62 |  | 0·14 (-0·03, 0·31) | 0·10 | 0·26 | 0.023 | 0·01 | 2324.055 |
| Midnightblue-LPC | **0·26 (0·06, 0·47)** | **0·01** | **0·02** | **0.027** | 0·04 | 2570.46 |  | 0·16 (-0·01, 0·33) | 0·07 | 0·21 | 0.027 | 0·01 | 2323.681 |
| Black-TG(o) | **0·26 (0·06, 0·46)** | **0·01** | **0·02** | **0.030** | 0·04 | 2570.09 |  | 0·11 (-0·07, 0·29) | 0·24 | 0·46 | 0.030 | 0·004 | 2325.178 |
| Purple-FFA | 0·23 (-0·01, 0·46) | 0·06 | 0·09 | 0.033 | 0·03 | 2572.08 |  | 0·08 (-0·10, 0·25) | 0·38 | 0·63 | 0.033 | 0·003 | 2326.037 |
| Yellow-TG(hub) | 0·14 (-0·07, 0·36) | 0·19 | 0·21 | 0.037 | 0·03 | 2574.37 |  | -0·003 (-0·19, 0·18) | 0·97 | 0·97 | 0.037 | 0·001 | 2326.749 |
| Greenyellow-LPC(hub) | 0·18 (-0·05, 0·40) | 0·12 | 0·15 | 0.040 | 0·03 | 2573.36 |  | 0·11 (-0·07, 0·29) | 0·22 | 0·46 | 0.040 | 0·004 | 2325.162 |
| Pink-LPC(hub) | 0·17 (-0·06, 0·39) | 0·14 | 0·16 | 0.043 | 0·03 | 2573.62 |  | 0·05 (-0·14, 0·23) | 0·63 | 0·81 | 0.043 | 0·002 | 2326.477 |
| Tan-LPC(hub) | 0·06 (-0·19, 0·31) | 0·62 | 0·62 | 0.047 | 0·03 | 2575.70 |  | 0·04 (-0·14, 0·23) | 0·65 | 0·81 | 0.047 | 0·002 | 2326.513 |
| Salmon-HexCer | 0·16 (-0·20, 0·12) | 0·12 | 0·15 | 0.050 | 0·03 | 2573.46 |  | -0·02 (-0·14, 0·11) | 0·80 | 0·85 | 0.050 | 0·002 | 2326.686 |

Note: AMD, adjusted mean difference in module eigenlipid (SD units with 95% CI) per unit increase in CBCL raw score; Adjusted Alpha, Significance levels (α) reported in this table have been adjusted using the Benjamini-Hochberg Procedure to control the false discovery rate in multiple comparisons (Benjamini Hochberg critical value); R^2^, variation in the outcome explained by the predictors; AIC, Akaike information criterion; AC, Acylcarnitine; PE, Phosphatidylethanolamine; TG, Triglyceride; PS, Phosphatidylserine; SM, Sphingomyelin; CE, Cholesteryl esters; LPC, Lysophosphatidylcholine; TG(o), Alkyldiacylglycerol; FFA, Free Fatty Acid; HexCer, Hexosylceramide; CBCL-ADHP, Child Behavior Checklist attention-deficit/hyperactivity problems subscale; CBCL-ASP, Child Behavior Checklist autism spectrum problems subscale.

^1^ Model adjusted for child sex, gestational age (weeks) at birth, minutes from cord blood sample collection to storage, days cord blood sample stored, maternal blood contamination and age at CBCL testing.

***Supplementary Table S4.1:*** **The association between the cord blood lipid module eigenlipids and subsequent CBCL-ADHD and CBCL-ASD outcomes at 2 years (Set A confounders).**

| **Cord blood lipid profile** | **CBCL-ADHD** **^1^** | | | |  | **CBCL-ASD ^1^** | | | |
| --- | --- | --- | --- | --- | --- | --- | --- | --- | --- |
| **Single eigenlipid model**  **(hub lipid's class)** | **AMD  (95% CI)** | ***P*-value** | **R^2^** | **AIC** |  | **AMD  (95% CI)** | ***P*-value** | **R^2^** | **AIC** |
| Cyan-AC | **0.38 (0.06, 0.71)** | **0.02** | 0.11 | 1297.61 |  | **0.39 (0.08, 0.70)** | **0.01** | 0.11 | 1180.54 |
| Turquoise-PE(hub) | **0.4 (0.09, 0.71)** | **0.01** | 0.11 | 1296.56 |  | **0.33 (0.09, 0.57)** | **0.01** | 0.10 | 1182.54 |
| Brown-TG(hub) | 0.13 (-0.14, 0.41) | 0.33 | 0.09 | 1302.78 |  | 0.11 (-0.13, 0.35) | 0.37 | 0.08 | 1189.12 |
| Magenta-PS(hub) | **0.37 (0.07, 0.66)** | **0.01** | 0.11 | 1297.83 |  | 0.2 (-0.03, 0.43) | 0.08 | 0.09 | 1187.27 |
| Blue-SMD(hub) | **0.42 (0.11, 0.73)** | **0.01** | 0.11 | 1295.87 |  | 0.16 (-0.14, 0.47) | 0.29 | 0.08 | 1188.16 |
| Red-CE(hub) | **0.42 (0.08, 0.75)** | **0.02** | 0.11 | 1296.44 |  | 0.2 (-0.19, 0.60) | 0.31 | 0.09 | 1187.37 |
| Green-LPC(hub) | **0.32 (0.02, 0.62)** | **0.04** | 0.10 | 1299.40 |  | 0.07 (-0.17, 0.32) | 0.56 | 0.08 | 1189.57 |
| Midnightblue-LPC | 0.12 (-0.17, 0.41) | 0.42 | 0.09 | 1303.07 |  | 0.01 (-0.25, 0.26) | 0.95 | 0.08 | 1189.90 |
| Black-TG(o) | 0.22 (-0.03, 0.47) | 0.09 | 0.09 | 1301.56 |  | 0.22 (-0.02, 0.47) | 0.08 | 0.09 | 1186.66 |
| Purple-FFA | 0.2 (-0.10, 0.49) | 0.19 | 0.09 | 1302.01 |  | 0.14 (-0.10, 0.37) | 0.25 | 0.08 | 1188.77 |
| Yellow-TG(hub) | 0.1 (-0.24, 0.44) | 0.56 | 0.09 | 1303.17 |  | -0.14 (-0.42, 0.15) | 0.34 | 0.08 | 1188.74 |
| Greenyellow-LPC(hub) | 0.23 (-0.10, 0.56) | 0.18 | 0.10 | 1301.35 |  | 0.24 (-0.03, 0.51) | 0.08 | 0.09 | 1186.03 |
| Pink-LPC(hub) | 0.2 (-0.12, 0.52) | 0.22 | 0.09 | 1301.84 |  | 0.06 (-0.22, 0.34) | 0.67 | 0.08 | 1189.65 |
| Tan-LPC(hub) | 0.06 (-0.23, 0.35) | 0.68 | 0.09 | 1303.43 |  | 0 (-0.22, 0.23) | 0.98 | 0.08 | 1189.90 |
| Salmon-HexCer | **0.32 (0.06, 0.58)** | **0.02** | **0.11** | 1298.00 |  | 0.09 (-0.13, 0.31) | 0.44 | 0.08 | 1189.30 |

**Note:** AMD, adjusted mean difference in module eigenlipid (SD units with 95% CI) per unit increase in CBCL raw score; R^2^, variation in the outcome explained by the predictors; AIC, Akaike information criterion; AC, Acylcarnitine; PE, Phosphatidylethanolamine; TG, Triglyceride; PS, Phosphatidylserine; SM, Sphingomyelin; CE, Cholesteryl esters; LPC, Lysophosphatidylcholine; TG(o), Alkyldiacylglycerol; FFA, Free Fatty Acid; HexCer, Hexosylceramide; CBCL-ADHP, Child Behavior Checklist attention-deficit/hyperactivity problems subscale; CBCL-ASP, Child Behavior Checklist autism spectrum problems subscale.

^1^ Model adjusted for child sex, gestational age (weeks) at birth, minutes from cord blood sample collection to storage, days cord blood sample stored, maternal blood contamination, age at CBCL testing, **household income (decreasing), high weight gain during pregnancy (>20kg vs <20kg), Apgar score at 5 minutes, maternal age, lone parent during pregnancy(yes vs no), perceived stress score during trimester 1/2 and smoking during pregnancy (any vs none).**

***Supplementary Table S4.2:*** **The association between the cord blood lipid module eigenlipids and subsequent CBCL-ADHD and CBCL-ASD outcomes at 2 years (Set B confounders).**

| **Cord blood lipid profile** | **CBCL-ADHD ^1^** | | | |  | **CBCL-ASD ^1^** | | | |
| --- | --- | --- | --- | --- | --- | --- | --- | --- | --- |
| **Single eigenlipid model**  **(hub lipid's class)** | **AMD  (95% CI)** | ***P-*value** | **R^2^** | **AIC** |  | **AMD  (95% CI)** | ***P*-value** | **R^2^** | **AIC** |
| Cyan-AC | **0.45 (0.13, 0.78)** | **0.01** | 0.10 | 1600.6 |  | **0.37 (0.10, 0.65)** | **0.01** | 0.12 | 1443.13 |
| Turquoise-PE(hub) | **0.39 (0.06, 0.72)** | **0.02** | 0.10 | 1602.8 |  | 0.24 (-0.01, 0.48) | 0.06 | 0.10 | 1448.75 |
| Brown-TG(hub) | 0.19 (-0.11, 0.50) | 0.22 | 0.08 | 1608.1 |  | 0.10 (-0.18, 0.38) | 0.48 | 0.10 | 1452.10 |
| Magenta-PS(hub) | **0.29 (0.00, 0.59)** | **0.048** | 0.09 | 1605.7 |  | 0.16 (-0.09, 0.41) | 0.22 | 0.10 | 1451.01 |
| Blue-SMD(hub) | **0.40 (0.12, 0.68)** | **0.01** | 0.10 | 1601.8 |  | 0.07 (-0.17, 0.31) | 0.57 | 0.09 | 1452.42 |
| Red-CE(hub) | **0.32 (0.03, 0.61)** | **0.03** | 0.09 | 1604.9 |  | 0.10 (-0.23, 0.43) | 0.56 | 0.10 | 1452.11 |
| Green-LPC(hub) | **0.37 (0.08, 0.66)** | **0.01** | 0.10 | 1603.4 |  | 0.12 (-0.11, 0.35) | 0.29 | 0.10 | 1451.69 |
| Midnightblue-LPC | 0.21 (-0.06, 0.48) | 0.13 | 0.09 | 1607.7 |  | 0.06 (-0.17, 0.28) | 0.63 | 0.09 | 1452.59 |
| Black-TG(o) | 0.20 (-0.06, 0.47) | 0.14 | 0.08 | 1607.9 |  | 0.15 (-0.10, 0.40) | 0.24 | 0.10 | 1451.23 |
| Purple-FFA | 0.13 (-0.16, 0.42) | 0.39 | 0.08 | 1609 |  | 0.07 (-0.18, 0.31) | 0.60 | 0.09 | 1452.48 |
| Yellow-TG(hub) | 0.17 (-0.15, 0.49) | 0.29 | 0.08 | 1608.4 |  | -0.12 (-0.39, 0.15) | 0.38 | 0.10 | 1451.79 |
| Greenyellow-LPC(hub) | 0.23 (-0.08, 0.53) | 0.14 | 0.09 | 1607.1 |  | 0.18 (-0.07, 0.42) | 0.16 | 0.10 | 1450.33 |
| Pink-LPC(hub) | 0.15 (-0.13, 0.44) | 0.29 | 0.08 | 1608.5 |  | 0.05 (-0.21, 0.31) | 0.70 | 0.09 | 1452.61 |
| Tan-LPC(hub) | 0.14 (-0.14, 0.42) | 0.32 | 0.08 | 1608.8 |  | 0.07 (-0.21, 0.35) | 0.64 | 0.09 | 1452.44 |
| Salmon-HexCer | **0.27 (0.02, 0.51)** | **0.03** | **0.09** | 1605.7 |  | 0.06 (-0.14, 0.26) | 0.54 | 0.09 | 1452.46 |

Note: AMD, adjusted mean difference in module eigenlipid (SD units with 95% CI) per unit increase in CBCL raw score; R^2^, variation in the outcome explained by the predictors; AIC, Akaike information criterion; AC, Acylcarnitine; PE, Phosphatidylethanolamine; TG, Triglyceride; PS, Phosphatidylserine; SM, Sphingomyelin; CE, Cholesteryl esters; LPC, Lysophosphatidylcholine; TG(o), Alkyldiacylglycerol; FFA, Free Fatty Acid; HexCer, Hexosylceramide; CBCL-ADHP, Child Behavior Checklist attention-deficit/hyperactivity problems subscale; CBCL-ASP, Child Behavior Checklist autism spectrum problems subscale.

^1^ Model adjusted for child sex, gestational age (weeks) at birth, minutes from cord blood sample collection to storage, days cord blood sample stored, maternal blood contamination, age at CBCL testing, **House hold income (decreasing), low birth weight (>2.5kg vs <2.5kg), high weight gain during pregnancy (>20kg vs <20kg), multiparity and Apgar score at 5 minutes.**

| ***Supplementary Table S4.3:* Details for descriptive and potential confounder variables.** | |
| --- | --- |
|  | **Details** |
| **Parent and household factors** | |
| Mother’s age at conception (years) | Derived from questionnaire administered at 28-week antenatal review. |
| Mother is university-educated | Derived from questionnaire administered at 28-week antenatal review (diploma or below = 0, university bachelor’s degree or above = 1). |
| Socio-economic disadvantage | The Australian Bureau of Statistics Socio-Economic Indexes for Areas (SEIFA) used to classify regions in Australia based on their level of socio-economic advantage or disadvantage, using data from the 2016 Census of Population and Housing.^1^ The Index of Relative Socio-economic Disadvantage (IRSD) used to measure the extent of economic and social disadvantage, with lower scores indicating greater disadvantage. To determine each participant's SEIFA IRSD, their residential address, obtained through a questionnaire, was used. A binary version of the variable was also created based on the 33^rd^ percentile to indicate socio-economic disadvantage (top two tertiles=0, bottom tertile=1). |
| Household income during pregnancy | Derived from questionnaire administered at 28 week-week antenatal review and follow-up repeated questionnaires. Household income from lowest to highest (per AUD $10,000) was recorded as the mean of the values during pregnancy and one year after birth, standardized to have a mean of 0 and a standard deviation of 1. |
| **Prenatal factors** | |
| Lone parent | Derived from questionnaire administered at 28-week antenatal review (parent has a partner = 0, parent does not have a partner = 1). |
| Mother’s weight at 28-week interview (kg) | Measured by Barwon Infant Study staff with scales at 28-week antenatal review. |
| Mother smoked at any point during pregnancy | Derived from questionnaire administered at 28-week antenatal review for Trimester 1 & 2, and questionnaire administered at 1-month postnatal review for Trimester 3 (did not smoke = 0, smoked = 1). |
| Mother’s Perceived Stress Scale in pregnancy | The Perceived Stress Scale (PSS) was administered during the 28-week antenatal review. The PSS is a 10-item questionnaire where participants are asked about the frequency of feelings and thoughts during the previous month. Responses were scored on a 5-point Likert scale (0 = never to 4 = very often), with scores calculated by summing the responses. |
| Maternal healthy dietary pattern | Administered at the 28-week antenatal review, the Dietary Questionnaire for Epidemiological Studies Version 2 (DQES2) was used to evaluate the mother's diet. This 80-item food frequency questionnaire asked participants to report the foods they had consumed in the previous four weeks.^4^ The items from this questionnaire were subjected to a principal component analysis, with the first principal component (PC1) showing high loadings on nuts, green vegetables, eggs, fish, and wholegrains, and low loadings on white bread, hamburgers, full-cream milk, and sugar. PC1 was utilized as a continuous measure of diet, where higher scores indicated a healthier dietary pattern, and lower scores indicated an unhealthy diet. Further details on this variable have been published previously. ^5^ |
| Maternal unhealthy dietary pattern | Administered at the 28-week antenatal review, the Dietary Questionnaire for Epidemiological Studies Version 2 (DQES2) was used to evaluate the mother's diet. This 80-item food frequency questionnaire asked participants to report the foods they had consumed in the previous four weeks. ^4^ The items from this questionnaire were subjected to a principal component analysis, with the second principal component (PC2) showing high loadings on pasta, chips, meat and take-away foods, sweet biscuits and confectionery. PC2 was utilized as a continuous measure of diet with high measurements representing an unhealthy dietary pattern and low measurements representing a healthy diet. Further details on this variable have been published previously. ^5^ |
| Gestational age at blood collection (weeks) | Obtained from research clinical examination forms |
| **Birth factors** | |
| Child’s assigned sex at birth | Obtained from birth records at obstetric hospital (0 = female, 1 = male) |
| Gestational age at birth (weeks) | Obtained from birth records at obstetric hospital |
| Child birthweight (kg) | Obtained from birth records at obstetric hospital |
| Mode of birth | Obtained from birth records at obstetric hospital (0 = unassisted vaginal birth, 1 = instrumental vaginal delivery, 2 = scheduled caesarean section, 3 = unscheduled caesarean section) |
| Labour time (hrs) | Obtained from birth records at obstetric hospital |
| Resuscitation at birth | Obtained from birth records at obstetric hospital (0 = other/nil, 1 = intubation or cardiac compressions) |
| Apgar score at 5 minutes per score (range: 0-10) | Obtained from birth records at obstetric hospital |
| Mother is multiparous | Derived from questionnaire administered at 28-week antenatal review (nulliparous = 0, multiparous = 1) |
| Maternal contamination of cord serum | To identify cord blood samples that have been contaminated by maternal blood a DNA methylation profiling method described by Morin et al. was used. After quality control, seven of the ten CpG sites known to significantly distinguish between maternal and infant biospecimens were available. Cord serum samples were classified as "contaminated" if four or more of these CpG sites exceeded the methylation threshold, and "uncontaminated" if not (uncontaminated = 0, contaminated = 1). |
|  |  |
|  |  |
| **Process factors** | |
| Time interval (mins) from blood collection to ‑80°C freezer storage. | Derived from research clinical examination forms |
| Days blood sample stored in ‑80°C freezer | Derived from research clinical examination forms |

| ***Supplementary Table S5:* The association between the cord blood lipid module eigenlipids and subsequent CBCL-ADHD and CBCL-ASD binary outcomes at 2 years.** | | | | | |  |
| --- | --- | --- | --- | --- | --- | --- |
|  | **CBCL-ADHD ^a^** | |  | **CBCL-ASD ^a^** | |  |
| **Cord blood lipid profile** |  | |  |  | |  |
| **Single eigenlipid model**  **(hub lipid's class)** | **AOR  (95% CI)** | ***P-value*** |  | **AOR (95% CI)** | ***P-value*** |  |
| Cyan-AC | **2.07 (1.47, 2.92)** | **<0.0001** |  | **1.52 (1.20, 1.93)** | **<0.0001** |  |
| Turquoise-PE(hub) | **1.75 (1.18, 2.58)** | **0.005** |  | **1.34 (1.05, 1.72)** | **0.02** |  |
| Brown-TG(hub) | **1.57 (1.13, 2.19)** | **0.01** |  | 1.21 (0.94, 1.55) | 0.14 |  |
| Magenta-PS(hub) | **1.38 (1.05, 1.83)** | **0.02** |  | **1.30 (1.03, 1.65)** | **0.03** |  |
| Blue-SMD(hub) | **1.65 (1.23, 2.20)** | **0.001** |  | 1.13 (0.90, 1.43) | 0.29 |  |
| Red-CE(hub) | **1.39 (1.05, 1.83)** | **0.02** |  | 1.11 (0.86, 1.42) | 0.43 |  |
| Green-LPC(hub) | **1.64 (1.22, 2.21)** | **0.001** |  | 1.20 (0.95, 1.51) | 0.14 |  |
| Midnightblue-LPC | **1.47 (1.10, 1.97)** | **0.01** |  | 1.22 (0.95, 1.57) | 0.11 |  |
| Black-TG(o) | **1.62 (1.17, 2.25)** | **0.01** |  | 1.07 (0.86, 1.33) | 0.53 |  |
| Purple-FFA | **1.49 (1.08, 2.06)** | **0.02** |  | 1.13 (0.88, 1.44) | 0.34 |  |
| Yellow-TG(hub) | **1.35 (1.00, 1.82)** | **0.05** |  | 0.92 (0.71, 1.18) | 0.5 |  |
| Greenyellow-LPC(hub) | 1.29 (0.98, 1.70) | 0.07 |  | 1.26 (0.98, 1.64) | 0.08 |  |
| Pink-LPC(hub) | 1.27 (0.94, 1.70) | 0.12 |  | 1.08 (0.83, 1.41) | 0.55 |  |
| Tan-LPC(hub) | 1.03 (0.72, 1.48) | 0.87 |  | 1.06 (0.81, 1.39) | 0.68 |  |
| Salmon-HexCer | 1.31 (0.99, 1.73) | 0.06 |  | 0.94 (0.76, 1.16) | 0.56 |  |
|  |  |  |  |  |  |  |
| **Note:** AOR, adjusted odds ratio in module eigenlipid (SD units with 95% CI) between the top 15% compared to the bottom 15% of CBCL scores; AC, Acylcarnitine; PE Phosphatidylethanolamine; TG, Triglyceride; PS, Phosphatidylserine; SM, Sphingomyelin; CE, Cholesteryl esters; LPC, Lysophosphatidylcholine; TG(o), Alkyldiacylglycerol; FFA, Free Fatty Acid; HexCer, Hexosylceramide; CBCL-ADHP, Child Behavior Checklist attention-deficit/hyperactivity problems subscale; CBCL-ASP, Child Behavior Checklist autism spectrum problems subscale.  ^a^ Model adjusted for child sex, gestational age (weeks) at birth, minutes from cord blood sample collection to storage, days cord blood sample stored, maternal blood contamination and age at CBCL testing | | | | | | |

***Supplementary Table S6.1:*** **The associations between the cord blood lipid feature concentrations and subsequent CBCL-ADHP and CBCL-ASP outcomes at 2 years.**

|  |  | **CBCL-ADHP** | | |  | **CBCL-ASP** | | |  |
| --- | --- | --- | --- | --- | --- | --- | --- | --- | --- |
| **Cord blood lipid species** | **Module Membership** | **AMD  (95% CI)** | ***P*-value** | ***Q-*value** |  | **AMD  (95% CI)** | ***P*-value** | ***Q*-value** |  |
| **Species (annotation)** |  |  |  |  |  |  |  |  |  |
| Sph(d16:1) | grey | 0.11 (-0.09, 0.31) | 0.29 | 0.34 |  | 0.10 (-0.40, 0.60) | 0.70 | 0.83 |  |
| Sph(d18:1) | grey | 0.23 (-0.02, 0.48) | 0.07 | 0.11 |  | 0.14 (-0.04, 0.33) | 0.13 | 0.34 |  |
| Sph(d18:2) | grey | -0.02 (-0.20, 0.16) | 0.84 | 0.85 |  | -0.35 (-1.25, 0.55) | 0.45 | 0.65 |  |
| S1P(d16:1) | tan | -0.05 (-0.35, 0.25) | 0.74 | 0.76 |  | -0.28 (-0.73, 0.18) | 0.23 | 0.46 |  |
| S1P(d18:0) | tan | -0.13 (-0.37, 0.12) | 0.30 | 0.35 |  | -0.17 (-0.48, 0.13) | 0.27 | 0.49 |  |
| S1P(d18:1) | tan | -0.13 (-0.36, 0.10) | 0.25 | 0.30 |  | -0.19 (-0.44, 0.07) | 0.15 | 0.35 |  |
| S1P(d18:2) | tan | 0.02 (-0.28, 0.32) | 0.91 | 0.91 |  | -0.09 (-0.37, 0.19) | 0.53 | 0.72 |  |
| Cer(d18:0/16:0) | turquoise | 0.22 (-0.04, 0.48) | 0.10 | 0.15 |  | **0.41 (0.07, 0.76)** | **0.02** | 0.15 |  |
| Cer(d18:0/18:0) | turquoise | **0.28 (0.02, 0.53)** | **0.03** | 0.06 |  | 0.23 (-0.06, 0.52) | 0.12 | 0.33 |  |
| Cer(d18:0/20:0) | grey | 0.17 (-0.09, 0.43) | 0.19 | 0.24 |  | 0.29 (-0.14, 0.72) | 0.19 | 0.41 |  |
| Cer(d18:0/22:0) | grey | 0.13 (-0.07, 0.32) | 0.21 | 0.25 |  | 0.30 (-0.05, 0.66) | 0.09 | 0.29 |  |
| Cer(d18:0/24:0) | grey | 0.06 (-0.14, 0.26) | 0.57 | 0.60 |  | 0.22 (-0.10, 0.53) | 0.17 | 0.39 |  |
| Cer(d18:0/24:1) | grey | 0.06 (-0.16, 0.28) | 0.58 | 0.61 |  | 0.16 (-0.13, 0.44) | 0.28 | 0.51 |  |
| Cer(d16:1/16:0) | turquoise | 0.35 (-0.05, 0.74) | 0.08 | 0.12 |  | 0.14 (-0.12, 0.40) | 0.28 | 0.51 |  |
| Cer(d16:1/18:0) | turquoise | **0.37 (0.08, 0.66)** | **0.01** | **0.03** |  | 0.17 (-0.05, 0.39) | 0.14 | 0.35 |  |
| Cer(d16:1/20:0) | turquoise | **0.47 (0.13, 0.80)** | **0.01** | **0.03** |  | 0.11 (-0.15, 0.38) | 0.40 | 0.62 |  |
| Cer(d16:1/22:0) | turquoise | **0.52 (0.05, 0.99)** | **0.03** | 0.06 |  | 0.13 (-0.14, 0.40) | 0.36 | 0.58 |  |
| Cer(d16:1/23:0) | turquoise | **0.62 (0.07, 1.17)** | **0.03** | 0.05 |  | 0.15 (-0.12, 0.42) | 0.27 | 0.49 |  |
| Cer(d16:1/24:0) | turquoise | **0.67 (0.17, 1.18)** | **0.01** | **0.03** |  | 0.22 (-0.07, 0.52) | 0.14 | 0.35 |  |
| Cer(d16:1/24:1) | turquoise | **0.44 (0.03, 0.86)** | **0.04** | 0.07 |  | 0.12 (-0.16, 0.41) | 0.39 | 0.62 |  |
| Cer(d17:1/16:0) | turquoise | 0.06 (-0.21, 0.34) | 0.66 | 0.69 |  | 0.06 (-0.21, 0.34) | 0.66 | 0.80 |  |
| Cer(d17:1/18:0) | turquoise | **0.30 (0.02, 0.59)** | **0.04** | 0.07 |  | 0.18 (-0.16, 0.52) | 0.29 | 0.52 |  |
| Cer(d17:1/20:0) | turquoise | **0.40 (0.06, 0.73)** | **0.02** | **0.04** |  | **0.31 (0.01, 0.61)** | **0.04** | 0.22 |  |
| Cer(d17:1/22:0) | turquoise | **0.49 (0.07, 0.90)** | **0.02** | **0.04** |  | 0.23 (-0.07, 0.54) | 0.13 | 0.34 |  |
| Cer(d17:1/23:0) | turquoise | **0.53 (0.04, 1.03)** | **0.03** | 0.06 |  | **0.34 (0.06, 0.62)** | **0.02** | 0.15 |  |
| Cer(d17:1/24:0) | turquoise | **0.60 (0.11, 1.08)** | **0.02** | **0.04** |  | 0.21 (-0.12, 0.55) | 0.21 | 0.43 |  |
| Cer(d17:1/24:1) | turquoise | **0.53 (0.12, 0.95)** | **0.01** | **0.03** |  | 0.22 (-0.09, 0.53) | 0.16 | 0.37 |  |
| Cer(d18:1/14:0) | turquoise | 0.18 (-0.05, 0.41) | 0.13 | 0.17 |  | 0.28 (-0.00, 0.57) | 0.05 | 0.23 |  |
| Cer(d18:1/16:0) | turquoise | **0.40 (0.08, 0.72)** | **0.01** | **0.04** |  | 0.29 (-0.01, 0.59) | 0.06 | 0.23 |  |
| Cer(d18:1/17:0) | grey | 0.08 (-0.14, 0.30) | 0.45 | 0.49 |  | -0.13 (-0.42, 0.17) | 0.40 | 0.62 |  |
| Cer(d18:1/18:0) | turquoise | **0.50 (0.21, 0.80)** | **0.00** | **0.02** |  | **0.31 (0.03, 0.59)** | **0.03** | 0.18 |  |
| Cer(d18:1/19:0) | turquoise | 0.22 (-0.06, 0.50) | 0.13 | 0.17 |  | 0.05 (-0.22, 0.33) | 0.71 | 0.84 |  |
| Cer(d18:1/20:0) | turquoise | **0.52 (0.18, 0.86)** | **0.00** | **0.02** |  | **0.30 (0.01, 0.59)** | **0.04** | 0.21 |  |
| Cer(d18:1/21:0) | turquoise | 0.37 (-0.02, 0.76) | 0.06 | 0.10 |  | 0.24 (-0.01, 0.49) | 0.06 | 0.24 |  |
| Cer(d18:1/22:0) | turquoise | **0.69 (0.31, 1.08)** | **0.00** | **0.01** |  | **0.37 (0.09, 0.65)** | **0.01** | 0.11 |  |
| Cer(d18:1/23:0) | turquoise | **0.76 (0.28, 1.24)** | **0.00** | **0.02** |  | **0.31 (0.08, 0.55)** | **0.01** | 0.11 |  |
| Cer(d18:1/24:0) | turquoise | **0.74 (0.38, 1.10)** | **0.00** | **0.00** |  | **0.41 (0.09, 0.72)** | **0.01** | 0.11 |  |
| Cer(d18:1/24:1) | turquoise | **0.63 (0.26, 0.99)** | **0.00** | **0.02** |  | 0.30 (-0.00, 0.60) | 0.05 | 0.23 |  |
| Cer(d18:1/26:0) | grey | 0.24 (-0.07, 0.54) | 0.13 | 0.17 |  | 0.34 (-0.05, 0.72) | 0.09 | 0.29 |  |
| Cer(d18:2/14:0) | turquoise | 0.27 (-0.06, 0.61) | 0.11 | 0.15 |  | **0.46 (0.04, 0.88)** | **0.03** | 0.18 |  |
| Cer(d18:2/16:0) | turquoise | **0.34 (0.08, 0.60)** | **0.01** | **0.03** |  | 0.22 (-0.06, 0.50) | 0.13 | 0.34 |  |
| Cer(d18:2/17:0) | grey | 0.16 (-0.05, 0.38) | 0.13 | 0.18 |  | 0.08 (-0.09, 0.25) | 0.33 | 0.56 |  |
| Cer(d18:2/18:0) | turquoise | **0.45 (0.19, 0.72)** | **0.00** | **0.02** |  | 0.27 (-0.00, 0.55) | 0.05 | 0.23 |  |
| Cer(d18:2/20:0) | turquoise | **0.48 (0.23, 0.73)** | **0.00** | **0.01** |  | 0.24 (-0.05, 0.52) | 0.10 | 0.31 |  |
| Cer(d18:2/21:0) | turquoise | **0.45 (0.10, 0.80)** | **0.01** | **0.03** |  | 0.38 (-0.07, 0.82) | 0.10 | 0.30 |  |
| Cer(d18:2/22:0) | turquoise | **0.54 (0.27, 0.82)** | **0.00** | **0.01** |  | 0.21 (-0.07, 0.50) | 0.14 | 0.35 |  |
| Cer(d18:2/23:0) | turquoise | **0.72 (0.31, 1.14)** | **0.00** | **0.01** |  | 0.25 (-0.00, 0.50) | 0.05 | 0.23 |  |
| Cer(d18:2/24:0) | turquoise | **0.63 (0.34, 0.92)** | **0.00** | **0.00** |  | 0.24 (-0.04, 0.53) | 0.09 | 0.29 |  |
| Cer(d18:2/24:1) | turquoise | **0.45 (0.15, 0.74)** | **0.00** | **0.02** |  | 0.22 (-0.07, 0.51) | 0.13 | 0.34 |  |
| Cer(d18:2/26:0) | turquoise | **0.37 (0.09, 0.65)** | **0.01** | **0.03** |  | 0.15 (-0.13, 0.43) | 0.29 | 0.51 |  |
| Cer(d19:1/16:0) | turquoise | 0.20 (-0.07, 0.47) | 0.14 | 0.18 |  | 0.12 (-0.10, 0.35) | 0.29 | 0.51 |  |
| Cer(d19:1/18:0) | turquoise | 0.23 (-0.11, 0.58) | 0.19 | 0.23 |  | 0.21 (-0.03, 0.45) | 0.09 | 0.29 |  |
| Cer(d19:1/20:0) | turquoise | 0.25 (-0.05, 0.56) | 0.10 | 0.14 |  | 0.15 (-0.18, 0.49) | 0.37 | 0.59 |  |
| Cer(d19:1/22:0) | turquoise | **0.54 (0.13, 0.94)** | **0.01** | **0.03** |  | 0.26 (-0.10, 0.62) | 0.15 | 0.36 |  |
| Cer(d19:1/23:0) | turquoise | **0.68 (0.22, 1.15)** | **0.00** | **0.02** |  | **0.33 (0.07, 0.58)** | **0.01** | 0.12 |  |
| Cer(d19:1/24:0) | blue | **0.54 (0.15, 0.94)** | **0.01** | **0.03** |  | 0.21 (-0.11, 0.53) | 0.19 | 0.41 |  |
| Cer(d19:1/24:1) | blue | **0.51 (0.12, 0.90)** | **0.01** | **0.03** |  | 0.22 (-0.11, 0.55) | 0.19 | 0.41 |  |
| Cer(d19:1/26:0) | grey | -0.04 (-0.30, 0.22) | 0.76 | 0.78 |  | 0.28 (-0.07, 0.62) | 0.11 | 0.33 |  |
| Cer(d20:1/22:0) | turquoise | 0.24 (-0.06, 0.53) | 0.12 | 0.16 |  | 0.03 (-0.35, 0.42) | 0.87 | 0.93 |  |
| Cer(d20:1/23:0) | turquoise | **0.38 (0.02, 0.74)** | **0.04** | 0.07 |  | 0.13 (-0.17, 0.42) | 0.40 | 0.62 |  |
| Cer(d20:1/24:0) | grey | 0.19 (-0.10, 0.47) | 0.19 | 0.24 |  | 0.10 (-0.28, 0.48) | 0.60 | 0.76 |  |
| Cer(d20:1/24:1) | blue | **0.35 (0.07, 0.63)** | **0.01** | **0.04** |  | 0.15 (-0.20, 0.50) | 0.41 | 0.63 |  |
| Cer(d20:1/26:0) | grey | 0.05 (-0.15, 0.26) | 0.60 | 0.64 |  | 0.12 (-0.21, 0.45) | 0.49 | 0.68 |  |
| Cer(m18:0/20:0) | turquoise | **0.46 (0.11, 0.81)** | **0.01** | **0.03** |  | 0.25 (-0.05, 0.56) | 0.11 | 0.31 |  |
| Cer(m18:0/22:0) | turquoise | **0.55 (0.18, 0.93)** | **0.00** | **0.02** |  | 0.22 (-0.07, 0.50) | 0.14 | 0.35 |  |
| Cer(m18:0/23:0) | turquoise | **0.61 (0.17, 1.06)** | **0.01** | **0.03** |  | **0.32 (0.08, 0.57)** | **0.01** | 0.11 |  |
| Cer(m18:0/24:0) | turquoise | **0.50 (0.15, 0.84)** | **0.01** | **0.02** |  | 0.27 (-0.00, 0.55) | 0.05 | 0.23 |  |
| Cer(m18:0/24:1) | turquoise | **0.45 (0.06, 0.84)** | **0.02** | **0.05** |  | **0.29 (0.03, 0.55)** | **0.03** | 0.18 |  |
| Cer(m18:1/18:0) | turquoise | **0.61 (0.28, 0.94)** | **0.00** | **0.01** |  | 0.25 (-0.03, 0.54) | 0.08 | 0.28 |  |
| Cer(m18:1/20:0) | turquoise | **0.47 (0.12, 0.82)** | **0.01** | **0.03** |  | 0.24 (-0.09, 0.56) | 0.15 | 0.36 |  |
| Cer(m18:1/22:0) | turquoise | **0.37 (0.07, 0.67)** | **0.02** | **0.04** |  | 0.16 (-0.12, 0.43) | 0.26 | 0.48 |  |
| Cer(m18:1/23:0) | turquoise | **0.54 (0.22, 0.87)** | **0.00** | **0.02** |  | 0.22 (-0.01, 0.45) | 0.06 | 0.24 |  |
| Cer(m18:1/24:0) | turquoise | **0.40 (0.12, 0.67)** | **0.00** | **0.02** |  | 0.19 (-0.12, 0.49) | 0.23 | 0.46 |  |
| Cer(m18:1/24:1) | turquoise | **0.48 (0.14, 0.82)** | **0.01** | **0.02** |  | **0.27 (0.00, 0.55)** | **0.05** | 0.23 |  |
| Cer1P(d18:1/16:0) | turquoise | **0.50 (0.11, 0.89)** | **0.01** | **0.03** |  | **0.32 (0.05, 0.60)** | **0.02** | 0.15 |  |
| HexCer(d16:1/18:0) | salmon | 0.08 (-0.11, 0.28) | 0.40 | 0.45 |  | -0.04 (-0.24, 0.17) | 0.74 | 0.85 |  |
| HexCer(d16:1/20:0) | salmon | 0.20 (-0.05, 0.45) | 0.11 | 0.15 |  | -0.05 (-0.25, 0.15) | 0.62 | 0.77 |  |
| HexCer(d16:1/22:0) | salmon | 0.13 (-0.21, 0.46) | 0.45 | 0.49 |  | -0.04 (-0.29, 0.21) | 0.76 | 0.87 |  |
| HexCer(d16:1/24:0) | salmon | 0.15 (-0.15, 0.44) | 0.34 | 0.38 |  | -0.18 (-0.46, 0.10) | 0.21 | 0.44 |  |
| HexCer(d18:1/16:0) | salmon | 0.12 (-0.08, 0.33) | 0.24 | 0.29 |  | -0.01 (-0.27, 0.25) | 0.96 | 0.98 |  |
| HexCer(d18:1/18:0) | salmon | 0.19 (-0.03, 0.41) | 0.10 | 0.14 |  | 0.01 (-0.24, 0.26) | 0.95 | 0.98 |  |
| **HexCer(d18:1/20:0)** | **salmon (hub)** | 0.15 (-0.09, 0.40) | 0.22 | 0.27 |  | 0.01 (-0.26, 0.27) | 0.97 | 0.98 |  |
| HexCer(d18:1/22:0) | salmon | **0.36 (0.04, 0.68)** | **0.03** | 0.06 |  | 0.05 (-0.24, 0.33) | 0.74 | 0.85 |  |
| HexCer(d18:1/24:0) | salmon | 0.08 (-0.11, 0.28) | 0.38 | 0.43 |  | -0.09 (-0.38, 0.20) | 0.55 | 0.73 |  |
| HexCer(d18:1/24:1) | salmon | 0.17 (-0.08, 0.42) | 0.17 | 0.22 |  | 0.02 (-0.29, 0.34) | 0.88 | 0.93 |  |
| HexCer(d18:2/18:0) | salmon | 0.16 (-0.05, 0.37) | 0.14 | 0.18 |  | -0.10 (-0.36, 0.15) | 0.43 | 0.64 |  |
| HexCer(d18:2/20:0) | salmon | 0.13 (-0.07, 0.34) | 0.19 | 0.24 |  | 0.10 (-0.11, 0.31) | 0.33 | 0.56 |  |
| HexCer(d18:2/22:0) | salmon | 0.19 (-0.04, 0.41) | 0.10 | 0.14 |  | -0.02 (-0.31, 0.28) | 0.92 | 0.96 |  |
| HexCer(d18:2/24:0) | salmon | 0.15 (-0.05, 0.34) | 0.14 | 0.18 |  | -0.12 (-0.42, 0.18) | 0.43 | 0.64 |  |
| Hex2Cer(d16:1/16:0) | turquoise | 0.27 (-0.13, 0.67) | 0.19 | 0.24 |  | 0.02 (-0.26, 0.30) | 0.89 | 0.93 |  |
| Hex2Cer(d16:1/24:1) | blue | 0.09 (-0.21, 0.39) | 0.54 | 0.58 |  | 0.02 (-0.25, 0.30) | 0.86 | 0.92 |  |
| Hex2Cer(d18:1/16:0) | turquoise | **0.37 (0.03, 0.72)** | **0.03** | 0.06 |  | 0.13 (-0.17, 0.44) | 0.39 | 0.62 |  |
| Hex2Cer(d18:1/18:0) | grey | -0.07 (-0.35, 0.22) | 0.65 | 0.67 |  | -0.12 (-0.43, 0.20) | 0.46 | 0.67 |  |
| Hex2Cer(d18:1/20:0) | turquoise | 0.07 (-0.17, 0.31) | 0.55 | 0.59 |  | -0.13 (-0.46, 0.20) | 0.44 | 0.65 |  |
| Hex2Cer(d18:1/22:0) | turquoise | 0.18 (-0.04, 0.40) | 0.11 | 0.16 |  | 0.08 (-0.27, 0.43) | 0.64 | 0.79 |  |
| Hex2Cer(d18:1/24:0) | turquoise | 0.05 (-0.13, 0.24) | 0.55 | 0.59 |  | 0.02 (-0.26, 0.30) | 0.87 | 0.93 |  |
| Hex2Cer(d18:1/24:1) | turquoise | 0.21 (-0.06, 0.47) | 0.12 | 0.17 |  | 0.12 (-0.19, 0.43) | 0.44 | 0.65 |  |
| Hex2Cer(d18:2/16:0) | blue | 0.17 (-0.15, 0.50) | 0.29 | 0.34 |  | 0.06 (-0.26, 0.37) | 0.73 | 0.85 |  |
| Hex2Cer(d18:2/24:1) | blue | 0.11 (-0.11, 0.34) | 0.32 | 0.37 |  | -0.13 (-0.49, 0.23) | 0.48 | 0.68 |  |
| Hex3Cer(d18:1/16:0) | blue | **0.33 (0.08, 0.58)** | **0.01** | **0.03** |  | 0.29 (-0.15, 0.72) | 0.20 | 0.42 |  |
| Hex3Cer(d18:1/18:0) | grey | **0.30 (0.03, 0.57)** | **0.03** | 0.06 |  | -0.15 (-0.58, 0.27) | 0.48 | 0.68 |  |
| Hex3Cer(d18:1/20:0) | blue | **0.36 (0.12, 0.59)** | **0.00** | **0.02** |  | 0.06 (-0.23, 0.36) | 0.67 | 0.81 |  |
| Hex3Cer(d18:1/22:0) | blue | **0.29 (0.03, 0.55)** | **0.03** | 0.05 |  | 0.16 (-0.18, 0.51) | 0.35 | 0.58 |  |
| Hex3Cer(d18:1/24:0) | blue | **0.26 (0.04, 0.48)** | **0.02** | **0.04** |  | 0.29 (-0.06, 0.63) | 0.10 | 0.31 |  |
| Hex3Cer(d18:1/24:1) | blue | **0.31 (0.08, 0.54)** | **0.01** | **0.03** |  | 0.23 (-0.12, 0.59) | 0.20 | 0.42 |  |
| GM3(d18:1/16:0) | blue | **0.34 (0.11, 0.58)** | **0.00** | **0.02** |  | 0.15 (-0.45, 0.75) | 0.62 | 0.77 |  |
| GM3(d18:1/18:0) | grey | 0.22 (-0.05, 0.48) | 0.11 | 0.15 |  | -0.20 (-0.67, 0.26) | 0.39 | 0.61 |  |
| GM3(d18:1/20:0) | grey | 0.10 (-0.23, 0.42) | 0.56 | 0.59 |  | -0.01 (-0.34, 0.32) | 0.95 | 0.98 |  |
| GM3(d18:1/22:0) | blue | 0.21 (-0.00, 0.42) | 0.05 | 0.08 |  | 0.07 (-0.26, 0.40) | 0.69 | 0.82 |  |
| GM3(d18:1/24:0) | blue | **0.22 (0.02, 0.42)** | **0.03** | 0.06 |  | 0.09 (-0.20, 0.38) | 0.55 | 0.73 |  |
| GM3(d18:1/24:1) | blue | **0.24 (0.04, 0.44)** | **0.02** | **0.04** |  | -0.01 (-0.32, 0.29) | 0.94 | 0.97 |  |
| GM1(d18:1/16:0) | blue | **0.38 (0.09, 0.66)** | **0.01** | **0.03** |  | 0.40 (-0.16, 0.95) | 0.16 | 0.37 |  |
| Sulfatide (d18:1:/16:0(OH)) | turquoise | 0.14 (-0.24, 0.52) | 0.48 | 0.52 |  | 0.10 (-0.18, 0.38) | 0.49 | 0.68 |  |
| Sulfatide (d18:1:/16:0) | blue | **0.48 (0.11, 0.86)** | **0.01** | **0.03** |  | 0.21 (-0.07, 0.48) | 0.14 | 0.35 |  |
| Sulfatide (d18:1:/24:0(OH)) | grey | 0.10 (-0.12, 0.31) | 0.38 | 0.42 |  | -0.14 (-0.48, 0.21) | 0.43 | 0.64 |  |
| Sulfatide (d18:1:/24:0) | blue | 0.17 (-0.07, 0.40) | 0.16 | 0.20 |  | -0.09 (-0.40, 0.22) | 0.57 | 0.74 |  |
| Sulfatide (d18:1:/24:1(OH)) | blue | 0.05 (-0.26, 0.37) | 0.73 | 0.76 |  | -0.02 (-0.27, 0.23) | 0.86 | 0.92 |  |
| Sulfatide (d18:1:/24:1) | blue | **0.30 (0.03, 0.56)** | **0.03** | 0.06 |  | 0.22 (-0.07, 0.50) | 0.13 | 0.34 |  |
| SM(34:3) | blue | **0.43 (0.15, 0.72)** | **0.00** | **0.02** |  | 0.20 (-0.20, 0.61) | 0.33 | 0.56 |  |
| SM(35:2) (b) | blue | **0.42 (0.17, 0.68)** | **0.00** | **0.02** |  | 0.15 (-0.04, 0.33) | 0.13 | 0.34 |  |
| SM(37:1) | blue | **0.42 (0.15, 0.70)** | **0.00** | **0.02** |  | 0.07 (-0.23, 0.38) | 0.63 | 0.78 |  |
| SM(37:2) | blue | **0.46 (0.18, 0.74)** | **0.00** | **0.02** |  | 0.16 (-0.12, 0.45) | 0.27 | 0.49 |  |
| SM(38:3) (a) | blue | **0.23 (0.01, 0.44)** | **0.04** | 0.07 |  | 0.03 (-0.33, 0.40) | 0.86 | 0.92 |  |
| SM(38:3) (b) | blue | **0.37 (0.10, 0.64)** | **0.01** | **0.03** |  | 0.15 (-0.18, 0.49) | 0.37 | 0.59 |  |
| SM(40:3) (a) | blue | **0.40 (0.17, 0.64)** | **0.00** | **0.02** |  | 0.12 (-0.24, 0.48) | 0.51 | 0.70 |  |
| SM(40:3) (b) | blue | **0.42 (0.11, 0.73)** | **0.01** | **0.03** |  | 0.03 (-0.35, 0.41) | 0.88 | 0.93 |  |
| SM(41:0) | blue | 0.12 (-0.09, 0.34) | 0.26 | 0.30 |  | 0.14 (-0.20, 0.48) | 0.43 | 0.64 |  |
| SM(41:1) (a) | blue | **0.25 (0.00, 0.49)** | **0.05** | 0.08 |  | 0.14 (-0.13, 0.40) | 0.31 | 0.54 |  |
| SM(43:1) | blue | **0.34 (0.11, 0.57)** | **0.00** | **0.02** |  | 0.13 (-0.20, 0.46) | 0.45 | 0.65 |  |
| SM(43:2) (b) | blue | **0.58 (0.26, 0.91)** | **0.00** | **0.01** |  | 0.18 (-0.21, 0.57) | 0.36 | 0.59 |  |
| SM(43:2) (c) | blue | **0.42 (0.18, 0.67)** | **0.00** | **0.02** |  | 0.13 (-0.19, 0.46) | 0.42 | 0.64 |  |
| SM(44:1) | blue | **0.33 (0.10, 0.56)** | **0.01** | **0.03** |  | 0.24 (-0.17, 0.64) | 0.25 | 0.47 |  |
| SM(44:2) | blue | **0.38 (0.15, 0.61)** | **0.00** | **0.02** |  | 0.18 (-0.34, 0.70) | 0.50 | 0.69 |  |
| SM(44:3) | blue | **0.37 (0.11, 0.63)** | **0.01** | **0.03** |  | 0.15 (-0.32, 0.62) | 0.53 | 0.71 |  |
| SM(d16:1/19:0) | blue | **0.50 (0.17, 0.84)** | **0.00** | **0.02** |  | 0.20 (-0.14, 0.53) | 0.25 | 0.47 |  |
| SM(d16:1/23:0)/SM(d17:1/22:0) | blue | **0.66 (0.26, 1.07)** | **0.00** | **0.02** |  | 0.09 (-0.19, 0.38) | 0.51 | 0.70 |  |
| SM(d16:1/24:1) | blue | **0.45 (0.13, 0.78)** | **0.01** | **0.03** |  | 0.12 (-0.19, 0.43) | 0.44 | 0.65 |  |
| SM(d17:1/14:0) | blue | **0.39 (0.05, 0.74)** | **0.03** | 0.05 |  | 0.08 (-0.31, 0.48) | 0.68 | 0.81 |  |
| SM(d17:1/16:0) | blue | **0.41 (0.08, 0.75)** | **0.02** | **0.04** |  | 0.07 (-0.26, 0.39) | 0.69 | 0.82 |  |
| SM(d17:1/24:1) | blue | **0.47 (0.17, 0.77)** | **0.00** | **0.02** |  | 0.09 (-0.24, 0.41) | 0.60 | 0.76 |  |
| SM(d18:0/14:0) | blue | 0.17 (-0.04, 0.39) | 0.11 | 0.16 |  | 0.20 (-0.22, 0.61) | 0.35 | 0.58 |  |
| SM(d18:0/16:0) | grey | 0.05 (-0.18, 0.28) | 0.67 | 0.70 |  | -0.06 (-0.53, 0.41) | 0.80 | 0.89 |  |
| SM(d18:0/22:0) | pink | 0.07 (-0.27, 0.41) | 0.69 | 0.71 |  | -0.01 (-0.38, 0.36) | 0.95 | 0.98 |  |
| SM(d18:1/14:0/SM(d16:1/16:0) | blue | **0.43 (0.08, 0.77)** | **0.02** | **0.04** |  | 0.08 (-0.29, 0.45) | 0.67 | 0.81 |  |
| **SM(d18:1/16:0)** | **blue (hub)** | **0.39 (0.14, 0.63)** | **0.00** | **0.02** |  | 0.10 (-0.25, 0.45) | 0.57 | 0.74 |  |
| SM(d18:1/17:0)/SM(d17:1/18:0) | blue | **0.31 (0.07, 0.55)** | **0.01** | **0.03** |  | 0.08 (-0.32, 0.47) | 0.70 | 0.83 |  |
| SM(d18:1/18:0/SM(d16:1/20:0) | blue | **0.41 (0.16, 0.66)** | **0.00** | **0.02** |  | 0.09 (-0.25, 0.44) | 0.60 | 0.76 |  |
| SM(d18:1/20:0)/SM(d16:1/22:0) | blue | **0.50 (0.20, 0.80)** | **0.00** | **0.02** |  | 0.06 (-0.29, 0.41) | 0.74 | 0.85 |  |
| SM(d18:1/22:0)/SM(d16:1/24:0) | blue | **0.52 (0.24, 0.81)** | **0.00** | **0.01** |  | 0.09 (-0.27, 0.45) | 0.61 | 0.76 |  |
| SM(d18:1/23:0)/SM(d17:1/24:0) | blue | **0.67 (0.27, 1.07)** | **0.00** | **0.02** |  | 0.14 (-0.14, 0.43) | 0.32 | 0.55 |  |
| SM(d18:1/24:0) | blue | **0.41 (0.19, 0.63)** | **0.00** | **0.01** |  | 0.11 (-0.27, 0.48) | 0.58 | 0.75 |  |
| SM(d18:1/24:1) | blue | **0.53 (0.23, 0.82)** | **0.00** | **0.01** |  | 0.13 (-0.26, 0.51) | 0.52 | 0.71 |  |
| SM(d18:2/14:0) | blue | **0.40 (0.12, 0.68)** | **0.00** | **0.02** |  | 0.16 (-0.28, 0.60) | 0.48 | 0.68 |  |
| SM(d18:2/16:0) | blue | **0.48 (0.20, 0.76)** | **0.00** | **0.02** |  | 0.18 (-0.17, 0.53) | 0.31 | 0.54 |  |
| SM(d18:2/17:0) | blue | **0.46 (0.16, 0.76)** | **0.00** | **0.02** |  | 0.07 (-0.26, 0.39) | 0.69 | 0.82 |  |
| SM(d18:2/18:0) | blue | **0.37 (0.11, 0.63)** | **0.01** | **0.03** |  | 0.05 (-0.37, 0.46) | 0.83 | 0.90 |  |
| SM(d18:2/18:1) | blue | **0.46 (0.16, 0.75)** | **0.00** | **0.02** |  | 0.18 (-0.15, 0.51) | 0.27 | 0.49 |  |
| SM(d18:2/20:0) | blue | **0.34 (0.10, 0.58)** | **0.01** | **0.03** |  | -0.05 (-0.43, 0.34) | 0.80 | 0.89 |  |
| SM(d18:2/22:0) | blue | **0.37 (0.15, 0.60)** | **0.00** | **0.02** |  | 0.04 (-0.25, 0.34) | 0.79 | 0.88 |  |
| SM(d18:2/23:0) | blue | **0.62 (0.27, 0.96)** | **0.00** | **0.01** |  | 0.15 (-0.10, 0.40) | 0.23 | 0.46 |  |
| SM(d18:2/24:0) | blue | **0.33 (0.13, 0.54)** | **0.00** | **0.02** |  | 0.00 (-0.37, 0.36) | 0.98 | 0.99 |  |
| SM(d19:1/24:1) | blue | **0.33 (0.00, 0.66)** | **0.05** | 0.08 |  | 0.05 (-0.36, 0.46) | 0.82 | 0.90 |  |
| PA(34:1) | magenta | **0.32 (0.07, 0.56)** | **0.01** | **0.03** |  | **0.27 (0.10, 0.43)** | **0.00** | **0.04** |  |
| PA(36:1) | magenta | **0.32 (0.12, 0.52)** | **0.00** | **0.02** |  | **0.27 (0.08, 0.47)** | **0.01** | 0.09 |  |
| PA(36:2) | magenta | **0.35 (0.07, 0.62)** | **0.01** | **0.04** |  | **0.26 (0.07, 0.45)** | **0.01** | 0.11 |  |
| PA(36:3) | magenta | **0.27 (0.06, 0.48)** | **0.01** | **0.03** |  | **0.21 (0.06, 0.37)** | **0.01** | 0.09 |  |
| PA(36:4) | magenta | **0.21 (0.02, 0.40)** | **0.03** | 0.06 |  | **0.18 (0.02, 0.34)** | **0.03** | 0.17 |  |
| PA(40:6) | magenta | **0.25 (0.00, 0.49)** | **0.05** | 0.08 |  | 0.23 (-0.01, 0.47) | 0.06 | 0.24 |  |
| PC(14:0_16:0) | blue | **0.33 (0.03, 0.63)** | **0.03** | 0.06 |  | 0.14 (-0.20, 0.48) | 0.43 | 0.64 |  |
| PC(14:0_20:4) | turquoise | **0.63 (0.20, 1.05)** | **0.00** | **0.02** |  | 0.23 (-0.08, 0.53) | 0.15 | 0.36 |  |
| PC(14:0_22:6) | blue | **0.48 (0.04, 0.91)** | **0.03** | 0.06 |  | 0.15 (-0.15, 0.46) | 0.32 | 0.56 |  |
| PC(15-MHDA_18:1) | blue | **0.65 (0.27, 1.03)** | **0.00** | **0.02** |  | 0.21 (-0.04, 0.46) | 0.10 | 0.30 |  |
| PC(15-MHDA_18:2) | turquoise | **0.55 (0.14, 0.95)** | **0.01** | **0.03** |  | **0.24 (0.03, 0.45)** | **0.02** | 0.16 |  |
| PC(15-MHDA_20:4) | blue | **0.43 (0.10, 0.75)** | **0.01** | **0.03** |  | 0.18 (-0.15, 0.51) | 0.29 | 0.51 |  |
| PC(15-MHDA_22:6) | blue | 0.27 (-0.06, 0.60) | 0.11 | 0.16 |  | 0.06 (-0.20, 0.31) | 0.67 | 0.81 |  |
| PC(15:0_20:3) | turquoise | **0.53 (0.10, 0.95)** | **0.02** | **0.04** |  | **0.32 (0.02, 0.63)** | **0.04** | 0.20 |  |
| PC(15:0_20:4) | blue | **0.59 (0.23, 0.95)** | **0.00** | **0.02** |  | 0.29 (-0.01, 0.60) | 0.06 | 0.24 |  |
| PC(15:0_22:6) | blue | **0.39 (0.01, 0.77)** | **0.05** | 0.08 |  | 0.13 (-0.13, 0.39) | 0.33 | 0.56 |  |
| PC(16:0_16:0) | blue | **0.24 (0.03, 0.45)** | **0.02** | 0.05 |  | 0.06 (-0.30, 0.43) | 0.73 | 0.85 |  |
| PC(16:0_18:0) | blue | **0.36 (0.09, 0.63)** | **0.01** | **0.03** |  | 0.01 (-0.42, 0.43) | 0.98 | 0.99 |  |
| PC(16:0_18:1) | blue | **0.35 (0.10, 0.60)** | **0.01** | **0.03** |  | 0.23 (-0.17, 0.63) | 0.25 | 0.47 |  |
| PC(16:0_18:2) | turquoise | **0.34 (0.04, 0.63)** | **0.03** | 0.05 |  | 0.24 (-0.12, 0.59) | 0.20 | 0.42 |  |
| PC(16:0_18:3) (a) | turquoise | **0.62 (0.11, 1.12)** | **0.02** | **0.04** |  | **0.25 (0.02, 0.48)** | **0.03** | 0.18 |  |
| PC(16:0_18:3) (b) | turquoise | **0.43 (0.10, 0.76)** | **0.01** | **0.03** |  | 0.14 (-0.15, 0.43) | 0.33 | 0.56 |  |
| PC(16:0_20:3) (a) | turquoise | **0.47 (0.17, 0.78)** | **0.00** | **0.02** |  | 0.29 (-0.06, 0.63) | 0.10 | 0.30 |  |
| PC(16:0_20:3) (b) | turquoise | **0.30 (0.06, 0.55)** | **0.02** | **0.04** |  | 0.12 (-0.20, 0.43) | 0.47 | 0.67 |  |
| PC(16:0_20:4) | blue | **0.35 (0.08, 0.62)** | **0.01** | **0.03** |  | 0.26 (-0.66, 1.18) | 0.58 | 0.75 |  |
| PC(16:0_20:5) | turquoise | 0.16 (-0.15, 0.47) | 0.32 | 0.36 |  | 0.06 (-0.17, 0.28) | 0.63 | 0.78 |  |
| PC(16:0_22:6) | blue | 0.26 (-0.10, 0.62) | 0.15 | 0.19 |  | -0.08 (-0.54, 0.37) | 0.71 | 0.84 |  |
| PC(16:1_18:2) | turquoise | **0.51 (0.10, 0.92)** | **0.02** | **0.04** |  | 0.20 (-0.05, 0.45) | 0.12 | 0.33 |  |
| PC(16:1_20:4) | turquoise | **0.51 (0.15, 0.87)** | **0.01** | **0.03** |  | 0.27 (-0.09, 0.63) | 0.14 | 0.34 |  |
| PC(16:1_22:6) | blue | 0.41 (-0.01, 0.84) | 0.06 | 0.09 |  | 0.05 (-0.34, 0.45) | 0.79 | 0.88 |  |
| PC(17:0_18:1) | blue | **0.66 (0.27, 1.05)** | **0.00** | **0.02** |  | 0.20 (-0.10, 0.49) | 0.20 | 0.42 |  |
| PC(17:0_18:2) | turquoise | **0.53 (0.13, 0.92)** | **0.01** | **0.03** |  | **0.24 (0.01, 0.48)** | **0.04** | 0.20 |  |
| PC(17:0_20:4) | blue | **0.41 (0.14, 0.68)** | **0.00** | **0.02** |  | 0.11 (-0.26, 0.48) | 0.57 | 0.74 |  |
| PC(17:0_22:6) | blue | 0.24 (-0.05, 0.53) | 0.10 | 0.14 |  | -0.01 (-0.28, 0.27) | 0.95 | 0.98 |  |
| PC(17:1_18:2) | turquoise | **0.45 (0.04, 0.87)** | **0.03** | 0.06 |  | 0.19 (-0.08, 0.47) | 0.16 | 0.37 |  |
| PC(18:0_18:1) | blue | **0.47 (0.18, 0.76)** | **0.00** | **0.02** |  | 0.21 (-0.14, 0.57) | 0.24 | 0.46 |  |
| PC(18:0_18:2) | turquoise | **0.51 (0.13, 0.88)** | **0.01** | **0.03** |  | 0.20 (-0.11, 0.51) | 0.21 | 0.43 |  |
| PC(18:0_20:3) | red | **0.36 (0.08, 0.64)** | **0.01** | **0.03** |  | 0.26 (-0.19, 0.71) | 0.26 | 0.48 |  |
| PC(18:0_20:4) | blue | **0.33 (0.08, 0.58)** | **0.01** | **0.03** |  | 0.12 (-0.37, 0.61) | 0.64 | 0.78 |  |
| PC(18:0_22:4) | blue | **0.30 (0.08, 0.53)** | **0.01** | **0.03** |  | 0.24 (-0.18, 0.67) | 0.26 | 0.48 |  |
| PC(18:0_22:5) (n3) | blue | 0.21 (-0.03, 0.46) | 0.09 | 0.13 |  | 0.14 (-0.12, 0.40) | 0.30 | 0.53 |  |
| PC(18:0_22:5) (n6) | pink | **0.24 (0.04, 0.45)** | **0.02** | **0.04** |  | 0.10 (-0.18, 0.39) | 0.48 | 0.68 |  |
| PC(18:0_22:6) | yellow | 0.14 (-0.15, 0.42) | 0.34 | 0.39 |  | -0.16 (-0.49, 0.18) | 0.36 | 0.58 |  |
| PC(18:1_18:1) | turquoise | **0.63 (0.26, 1.01)** | **0.00** | **0.02** |  | 0.22 (-0.11, 0.56) | 0.18 | 0.40 |  |
| PC(18:1_18:2) | turquoise | **0.60 (0.18, 1.03)** | **0.01** | **0.03** |  | 0.22 (-0.05, 0.48) | 0.11 | 0.31 |  |
| PC(18:1_20:3) | turquoise | **0.45 (0.15, 0.75)** | **0.00** | **0.02** |  | 0.32 (-0.05, 0.68) | 0.09 | 0.29 |  |
| PC(18:1_22:6) (a) | blue | 0.28 (-0.08, 0.65) | 0.13 | 0.17 |  | 0.06 (-0.39, 0.52) | 0.78 | 0.88 |  |
| PC(18:1_22:6) (b) | blue | 0.17 (-0.14, 0.49) | 0.29 | 0.34 |  | 0.03 (-0.31, 0.37) | 0.87 | 0.93 |  |
| PC(18:2_18:2) | turquoise | **0.74 (0.20, 1.29)** | **0.01** | **0.03** |  | 0.25 (-0.02, 0.53) | 0.07 | 0.25 |  |
| PC(18:2_20:5) | turquoise | 0.39 (-0.05, 0.83) | 0.08 | 0.12 |  | 0.16 (-0.10, 0.43) | 0.23 | 0.46 |  |
| PC(20:0_20:4) | blue | **0.39 (0.15, 0.62)** | **0.00** | **0.02** |  | 0.25 (-0.26, 0.77) | 0.33 | 0.56 |  |
| PC(28:0) | turquoise | 0.39 (-0.01, 0.78) | 0.05 | 0.09 |  | 0.09 (-0.26, 0.44) | 0.61 | 0.76 |  |
| PC(31:0) (a) | blue | **0.60 (0.17, 1.04)** | **0.01** | **0.03** |  | **0.29 (0.03, 0.55)** | **0.03** | 0.17 |  |
| PC(31:0) (b) | blue | 0.23 (-0.04, 0.50) | 0.09 | 0.13 |  | 0.09 (-0.23, 0.41) | 0.58 | 0.75 |  |
| PC(32:1) | turquoise | **0.38 (0.02, 0.73)** | **0.04** | 0.07 |  | 0.18 (-0.16, 0.53) | 0.29 | 0.52 |  |
| PC(32:2) | turquoise | **0.53 (0.11, 0.95)** | **0.01** | **0.04** |  | 0.18 (-0.08, 0.44) | 0.17 | 0.39 |  |
| PC(33:0) (a) | blue | **0.54 (0.18, 0.90)** | **0.00** | **0.02** |  | 0.25 (-0.04, 0.54) | 0.09 | 0.29 |  |
| PC(33:0) (b) | blue | **0.32 (0.07, 0.58)** | **0.01** | **0.04** |  | 0.15 (-0.16, 0.46) | 0.33 | 0.56 |  |
| PC(33:1) | blue | **0.59 (0.20, 0.98)** | **0.00** | **0.02** |  | 0.21 (-0.05, 0.47) | 0.11 | 0.32 |  |
| PC(33:2) | turquoise | **0.61 (0.16, 1.05)** | **0.01** | **0.03** |  | 0.22 (-0.01, 0.46) | 0.07 | 0.25 |  |
| PC(34:5) | turquoise | 0.41 (-0.04, 0.86) | 0.07 | 0.11 |  | 0.16 (-0.07, 0.39) | 0.18 | 0.40 |  |
| PC(35:5) | turquoise | 0.30 (-0.10, 0.71) | 0.15 | 0.19 |  | 0.13 (-0.06, 0.32) | 0.17 | 0.39 |  |
| PC(36:6) (a) | turquoise | 0.22 (-0.16, 0.60) | 0.26 | 0.31 |  | 0.11 (-0.14, 0.35) | 0.39 | 0.61 |  |
| PC(38:2) | turquoise | **0.37 (0.12, 0.62)** | **0.00** | **0.02** |  | 0.21 (-0.27, 0.69) | 0.40 | 0.62 |  |
| PC(38:4) (b) | blue | **0.47 (0.15, 0.78)** | **0.00** | **0.02** |  | 0.34 (-0.12, 0.79) | 0.15 | 0.35 |  |
| PC(38:5) (a) | blue | **0.31 (0.06, 0.57)** | **0.02** | **0.04** |  | 0.31 (-0.09, 0.71) | 0.13 | 0.34 |  |
| PC(38:5) (b) | blue | **0.36 (0.06, 0.65)** | **0.02** | **0.04** |  | 0.20 (-0.21, 0.61) | 0.33 | 0.56 |  |
| PC(38:6) (a) | turquoise | **0.44 (0.07, 0.81)** | **0.02** | **0.04** |  | 0.18 (-0.12, 0.48) | 0.25 | 0.47 |  |
| PC(38:7)(c) | blue | **0.45 (0.08, 0.83)** | **0.02** | **0.04** |  | 0.07 (-0.31, 0.45) | 0.71 | 0.84 |  |
| PC(39:5)(a) | blue | 0.24 (-0.07, 0.54) | 0.13 | 0.17 |  | 0.03 (-0.22, 0.28) | 0.82 | 0.90 |  |
| PC(39:5)(b) | blue | **0.42 (0.10, 0.73)** | **0.01** | **0.03** |  | 0.18 (-0.11, 0.48) | 0.23 | 0.46 |  |
| PC(40:10) | grey | 0.22 (-0.00, 0.44) | 0.05 | 0.09 |  | 0.09 (-0.03, 0.21) | 0.14 | 0.35 |  |
| PC(40:7) (a) | blue | **0.48 (0.20, 0.77)** | **0.00** | **0.02** |  | 0.28 (-0.14, 0.70) | 0.19 | 0.41 |  |
| PC(40:8) | blue | **0.49 (0.18, 0.79)** | **0.00** | **0.02** |  | 0.23 (-0.16, 0.61) | 0.25 | 0.47 |  |
| PC(44:12) | blue | 0.31 (-0.08, 0.71) | 0.12 | 0.16 |  | -0.05 (-0.31, 0.21) | 0.70 | 0.83 |  |
| PC(O-16:0/16:0) | blue | **0.26 (0.05, 0.47)** | **0.02** | **0.04** |  | 0.07 (-0.30, 0.44) | 0.71 | 0.83 |  |
| PC(O-16:0/20:3) | blue | **0.28 (0.01, 0.55)** | **0.04** | 0.08 |  | 0.08 (-0.30, 0.46) | 0.67 | 0.81 |  |
| PC(O-16:0/20:4) | blue | **0.30 (0.08, 0.52)** | **0.01** | **0.03** |  | 0.11 (-0.21, 0.43) | 0.50 | 0.70 |  |
| PC(O-16:0/22:6) | blue | 0.19 (-0.07, 0.44) | 0.15 | 0.19 |  | -0.06 (-0.41, 0.30) | 0.75 | 0.86 |  |
| PC(O-18:0/18:1) | blue | **0.45 (0.10, 0.80)** | **0.01** | **0.03** |  | 0.16 (-0.18, 0.50) | 0.36 | 0.58 |  |
| PC(O-18:0/18:2) | turquoise | **0.55 (0.10, 0.99)** | **0.02** | **0.04** |  | 0.18 (-0.03, 0.39) | 0.09 | 0.29 |  |
| PC(O-18:0/20:4) | blue | **0.28 (0.01, 0.56)** | **0.04** | 0.08 |  | -0.03 (-0.37, 0.32) | 0.88 | 0.93 |  |
| PC(O-18:0/22:6) | blue | 0.24 (-0.07, 0.54) | 0.12 | 0.17 |  | -0.03 (-0.39, 0.32) | 0.85 | 0.92 |  |
| PC(O-18:1/18:1) | blue | **0.32 (0.00, 0.64)** | **0.05** | 0.08 |  | 0.12 (-0.07, 0.31) | 0.22 | 0.44 |  |
| PC(O-18:1/18:2) | turquoise | **0.50 (0.10, 0.90)** | **0.02** | **0.04** |  | 0.18 (-0.12, 0.48) | 0.25 | 0.47 |  |
| PC(O-32:1) | blue | 0.10 (-0.17, 0.36) | 0.47 | 0.51 |  | -0.06 (-0.40, 0.29) | 0.75 | 0.86 |  |
| PC(O-32:2) | blue | **0.33 (0.02, 0.65)** | **0.04** | 0.07 |  | 0.07 (-0.26, 0.39) | 0.70 | 0.83 |  |
| PC(O-34:1) | blue | **0.26 (0.02, 0.50)** | **0.03** | 0.06 |  | 0.17 (-0.12, 0.47) | 0.25 | 0.47 |  |
| PC(O-34:2) | turquoise | **0.43 (0.02, 0.83)** | **0.04** | 0.07 |  | 0.09 (-0.20, 0.39) | 0.54 | 0.72 |  |
| PC(O-34:4) | blue | **0.48 (0.21, 0.75)** | **0.00** | **0.01** |  | 0.23 (-0.03, 0.48) | 0.08 | 0.28 |  |
| PC(O-35:4) | blue | **0.45 (0.16, 0.75)** | **0.00** | **0.02** |  | 0.11 (-0.19, 0.41) | 0.48 | 0.68 |  |
| PC(O-36:0) | turquoise | **0.34 (0.11, 0.58)** | **0.00** | **0.02** |  | 0.09 (-0.25, 0.44) | 0.59 | 0.76 |  |
| PC(O-36:5) | turquoise | 0.14 (-0.20, 0.48) | 0.41 | 0.46 |  | -0.02 (-0.30, 0.26) | 0.90 | 0.94 |  |
| PC(O-38:5) | blue | **0.30 (0.03, 0.56)** | **0.03** | 0.05 |  | 0.15 (-0.17, 0.47) | 0.37 | 0.59 |  |
| PC(O-40:5) | blue | **0.43 (0.08, 0.79)** | **0.02** | **0.04** |  | 0.15 (-0.20, 0.50) | 0.41 | 0.62 |  |
| PC(O-40:7) (a) | blue | 0.30 (-0.02, 0.62) | 0.06 | 0.10 |  | 0.04 (-0.31, 0.38) | 0.84 | 0.91 |  |
| PC(O-40:7) (b) | blue | 0.17 (-0.03, 0.37) | 0.10 | 0.14 |  | 0.10 (-0.23, 0.44) | 0.54 | 0.73 |  |
| PC(P-15:0/20:4) (a) | turquoise | 0.52 (-0.00, 1.05) | 0.05 | 0.08 |  | 0.12 (-0.15, 0.38) | 0.39 | 0.62 |  |
| PC(P-15:0/20:4) (b) | blue | 0.31 (-0.02, 0.63) | 0.07 | 0.10 |  | -0.01 (-0.40, 0.38) | 0.96 | 0.98 |  |
| PC(P-16:0/14:0) | blue | 0.16 (-0.04, 0.35) | 0.12 | 0.16 |  | 0.05 (-0.25, 0.34) | 0.76 | 0.87 |  |
| PC(P-16:0/16:0) | blue | **0.18 (0.01, 0.36)** | **0.04** | 0.07 |  | 0.09 (-0.23, 0.41) | 0.56 | 0.74 |  |
| PC(P-16:0/16:1) | blue | 0.20 (-0.05, 0.44) | 0.11 | 0.16 |  | 0.12 (-0.20, 0.44) | 0.45 | 0.65 |  |
| PC(P-16:0/18:0) | blue | 0.20 (-0.05, 0.45) | 0.11 | 0.16 |  | 0.02 (-0.18, 0.21) | 0.86 | 0.92 |  |
| PC(P-16:0/18:1) | blue | **0.29 (0.07, 0.52)** | **0.01** | **0.03** |  | 0.06 (-0.27, 0.38) | 0.72 | 0.84 |  |
| PC(P-16:0/18:2) | turquoise | **0.44 (0.07, 0.80)** | **0.02** | **0.04** |  | 0.06 (-0.17, 0.29) | 0.60 | 0.76 |  |
| PC(P-16:0/18:3) | turquoise | **0.53 (0.04, 1.02)** | **0.03** | 0.06 |  | 0.20 (-0.09, 0.49) | 0.18 | 0.40 |  |
| PC(P-16:0/20:4) | blue | **0.33 (0.12, 0.55)** | **0.00** | **0.02** |  | 0.12 (-0.18, 0.42) | 0.43 | 0.64 |  |
| PC(P-16:0/20:5) | turquoise | 0.20 (-0.16, 0.56) | 0.28 | 0.33 |  | 0.04 (-0.23, 0.31) | 0.79 | 0.88 |  |
| PC(P-16:0/22:6) | blue | 0.20 (-0.05, 0.46) | 0.12 | 0.16 |  | -0.03 (-0.35, 0.28) | 0.83 | 0.90 |  |
| PC(P-17:0/20:4) (a) | blue | 0.18 (-0.14, 0.50) | 0.27 | 0.32 |  | 0.01 (-0.30, 0.32) | 0.97 | 0.98 |  |
| PC(P-17:0/20:4) (b) | blue | 0.26 (-0.05, 0.56) | 0.10 | 0.14 |  | -0.02 (-0.39, 0.34) | 0.89 | 0.94 |  |
| PC(P-18:0/18:2) | turquoise | **0.60 (0.13, 1.07)** | **0.01** | **0.03** |  | 0.10 (-0.14, 0.35) | 0.41 | 0.63 |  |
| PC(P-18:0/20:4) | blue | **0.34 (0.07, 0.60)** | **0.01** | **0.04** |  | 0.07 (-0.22, 0.37) | 0.64 | 0.78 |  |
| PC(P-18:0/22:5) | blue | **0.38 (0.08, 0.68)** | **0.01** | **0.03** |  | 0.09 (-0.29, 0.47) | 0.64 | 0.78 |  |
| PC(P-18:0/22:6) | blue | 0.29 (-0.01, 0.60) | 0.06 | 0.10 |  | 0.00 (-0.31, 0.31) | 1.00 | 1.00 |  |
| PC(P-18:1/18:1) | blue | **0.31 (0.08, 0.55)** | **0.01** | **0.03** |  | 0.13 (-0.21, 0.48) | 0.45 | 0.65 |  |
| PC(P-18:1/22:6) | blue | **0.36 (0.01, 0.70)** | **0.04** | 0.08 |  | 0.10 (-0.21, 0.40) | 0.54 | 0.73 |  |
| PC(P-20:0/20:4) | blue | **0.31 (0.05, 0.57)** | **0.02** | **0.04** |  | 0.10 (-0.30, 0.50) | 0.62 | 0.77 |  |
| PC(P-35:2)(a) | turquoise | **0.50 (0.03, 0.97)** | **0.04** | 0.07 |  | 0.11 (-0.11, 0.33) | 0.32 | 0.55 |  |
| PC(P-35:2)(b) | turquoise | 0.39 (-0.01, 0.80) | 0.06 | 0.09 |  | 0.08 (-0.09, 0.26) | 0.36 | 0.59 |  |
| PC(P-36:3) | blue | **0.23 (0.02, 0.45)** | **0.03** | 0.06 |  | 0.04 (-0.31, 0.39) | 0.81 | 0.89 |  |
| PC(P-38:5) (a) | blue | **0.29 (0.02, 0.56)** | **0.04** | 0.07 |  | 0.13 (-0.19, 0.45) | 0.42 | 0.63 |  |
| PC(P-38:5) (b) | blue | **0.33 (0.11, 0.54)** | **0.00** | **0.02** |  | 0.11 (-0.21, 0.42) | 0.51 | 0.70 |  |
| LPC(14:0) [sn1] | tan | 0.12 (-0.14, 0.38) | 0.37 | 0.41 |  | 0.13 (-0.15, 0.41) | 0.37 | 0.60 |  |
| LPC(14:0) [sn2] | tan | -0.05 (-0.33, 0.23) | 0.73 | 0.75 |  | 0.06 (-0.22, 0.35) | 0.67 | 0.81 |  |
| LPC(15-MHDA) [sn1] [104_sn1] | midnightblue | **0.37 (0.04, 0.70)** | **0.03** | 0.06 |  | 0.29 (-0.02, 0.59) | 0.07 | 0.25 |  |
| LPC(15-MHDA) [sn1] / LPC(17:0) [sn2] | midnightblue | **0.35 (0.03, 0.67)** | **0.03** | 0.06 |  | 0.27 (-0.09, 0.62) | 0.14 | 0.35 |  |
| LPC(15-MHDA) [sn2] | midnightblue | **0.39 (0.05, 0.73)** | **0.03** | 0.05 |  | 0.33 (-0.02, 0.67) | 0.06 | 0.24 |  |
| LPC(15:0) [sn1] | tan | 0.17 (-0.12, 0.46) | 0.24 | 0.29 |  | 0.26 (-0.07, 0.59) | 0.12 | 0.34 |  |
| LPC(15:0) [sn2] | tan | 0.15 (-0.14, 0.45) | 0.31 | 0.36 |  | 0.26 (-0.07, 0.59) | 0.12 | 0.33 |  |
| LPC(16:0) [sn1] | green | **0.37 (0.13, 0.61)** | **0.00** | **0.02** |  | 0.32 (-0.01, 0.65) | 0.06 | 0.23 |  |
| LPC(16:0) [sn2] | green | **0.41 (0.15, 0.67)** | **0.00** | **0.02** |  | **0.41 (0.09, 0.73)** | **0.01** | 0.12 |  |
| LPC(16:1) [sn1] | tan | 0.04 (-0.23, 0.32) | 0.76 | 0.78 |  | 0.06 (-0.18, 0.29) | 0.63 | 0.78 |  |
| LPC(16:1) [sn2] | tan | 0.12 (-0.18, 0.41) | 0.43 | 0.47 |  | 0.09 (-0.16, 0.35) | 0.46 | 0.67 |  |
| LPC(17:0) [sn1] | midnightblue | **0.32 (0.01, 0.63)** | **0.04** | 0.07 |  | 0.22 (-0.15, 0.59) | 0.24 | 0.46 |  |
| LPC(17:1) (a) [sn1] [104_sn1] | tan | 0.20 (-0.07, 0.48) | 0.14 | 0.19 |  | 0.20 (-0.12, 0.53) | 0.21 | 0.44 |  |
| LPC(17:1) [sn1] (a) / LPC(17:1) [sn2] (b) | tan | 0.20 (-0.08, 0.49) | 0.16 | 0.20 |  | 0.20 (-0.14, 0.55) | 0.24 | 0.47 |  |
| LPC(17:1) [sn1] (b) | tan | -0.03 (-0.41, 0.34) | 0.86 | 0.86 |  | -0.01 (-0.34, 0.32) | 0.97 | 0.98 |  |
| LPC(17:1) [sn2] (a) | tan | 0.16 (-0.11, 0.44) | 0.25 | 0.30 |  | 0.14 (-0.22, 0.50) | 0.44 | 0.65 |  |
| **LPC(18:0) [sn1]** | **green (hub)** | 0.28 (-0.01, 0.57) | 0.06 | 0.09 |  | 0.21 (-0.15, 0.57) | 0.26 | 0.48 |  |
| LPC(18:0) [sn2] | green | **0.32 (0.03, 0.60)** | **0.03** | 0.06 |  | 0.17 (-0.18, 0.52) | 0.35 | 0.58 |  |
| LPC(18:1) [sn1] | greenyellow | 0.18 (-0.09, 0.45) | 0.18 | 0.23 |  | 0.14 (-0.24, 0.52) | 0.48 | 0.68 |  |
| LPC(18:1) [sn2] | greenyellow | **0.23 (0.00, 0.46)** | **0.05** | 0.08 |  | 0.15 (-0.19, 0.49) | 0.39 | 0.62 |  |
| **LPC(18:2) [sn1]** | **greenyellow (hub)** | 0.10 (-0.25, 0.46) | 0.57 | 0.60 |  | 0.02 (-0.27, 0.31) | 0.91 | 0.95 |  |
| LPC(18:2) [sn2] | greenyellow | 0.22 (-0.07, 0.51) | 0.14 | 0.18 |  | 0.04 (-0.27, 0.35) | 0.80 | 0.89 |  |
| LPC(18:3) (a) [sn1] [104_sn1] | greenyellow | 0.09 (-0.27, 0.45) | 0.63 | 0.66 |  | 0.26 (-0.02, 0.54) | 0.07 | 0.26 |  |
| LPC(18:3) [sn1] (a)/LPC(18:3) [sn2] (b) | greenyellow | 0.14 (-0.14, 0.42) | 0.32 | 0.36 |  | 0.10 (-0.18, 0.37) | 0.50 | 0.69 |  |
| LPC(18:3) [sn1] (b) | tan | 0.07 (-0.17, 0.31) | 0.58 | 0.61 |  | 0.00 (-0.23, 0.23) | 0.99 | 1.00 |  |
| LPC(18:3) [sn2] (a) | greenyellow | 0.36 (-0.01, 0.73) | 0.05 | 0.09 |  | **0.40 (0.08, 0.71)** | **0.01** | 0.12 |  |
| LPC(19:0) (a) [sn1] [104_sn1] | midnightblue | 0.16 (-0.04, 0.37) | 0.12 | 0.16 |  | 0.18 (-0.01, 0.37) | 0.07 | 0.25 |  |
| **LPC(19:0) [sn1] (a) / LPC(19:0) [sn2] (b)** | **midnightblue (hub)** | 0.16 (-0.03, 0.35) | 0.11 | 0.15 |  | 0.21 (-0.01, 0.44) | 0.06 | 0.25 |  |
| LPC(19:0) [sn1] (b) | midnightblue | 0.17 (-0.07, 0.42) | 0.16 | 0.20 |  | 0.17 (-0.10, 0.44) | 0.21 | 0.44 |  |
| LPC(19:0) [sn2] (a) | midnightblue | 0.17 (-0.04, 0.38) | 0.12 | 0.16 |  | 0.17 (-0.03, 0.38) | 0.10 | 0.30 |  |
| LPC(19:1) (a) | midnightblue | **0.35 (0.09, 0.60)** | **0.01** | **0.03** |  | 0.25 (-0.06, 0.56) | 0.11 | 0.32 |  |
| LPC(19:1) (b) | midnightblue | **0.39 (0.12, 0.67)** | **0.01** | **0.02** |  | 0.27 (-0.10, 0.63) | 0.15 | 0.36 |  |
| LPC(19:1) (c) | grey | 0.26 (-0.05, 0.58) | 0.10 | 0.15 |  | -0.04 (-0.35, 0.28) | 0.82 | 0.90 |  |
| LPC(20:0) [sn1] | midnightblue | **0.44 (0.11, 0.77)** | **0.01** | **0.03** |  | 0.15 (-0.23, 0.54) | 0.43 | 0.64 |  |
| LPC(20:0) [sn2] | midnightblue | **0.45 (0.15, 0.75)** | **0.00** | **0.02** |  | 0.26 (-0.07, 0.60) | 0.12 | 0.34 |  |
| LPC(20:1) [sn1] | green | **0.44 (0.12, 0.77)** | **0.01** | **0.03** |  | 0.33 (-0.08, 0.75) | 0.12 | 0.33 |  |
| LPC(20:1) [sn2] | green | **0.45 (0.14, 0.76)** | **0.00** | **0.02** |  | 0.31 (-0.09, 0.71) | 0.13 | 0.34 |  |
| LPC(20:2) [sn1] | greenyellow | 0.27 (-0.00, 0.53) | 0.05 | 0.09 |  | 0.18 (-0.26, 0.62) | 0.42 | 0.64 |  |
| LPC(20:2) [sn2] | greenyellow | **0.30 (0.06, 0.55)** | **0.02** | **0.04** |  | 0.15 (-0.32, 0.62) | 0.53 | 0.71 |  |
| LPC(20:3) [sn1] | pink | 0.18 (-0.12, 0.49) | 0.23 | 0.28 |  | 0.04 (-0.29, 0.36) | 0.82 | 0.90 |  |
| LPC(20:3) [sn2] | pink | 0.22 (-0.05, 0.48) | 0.11 | 0.16 |  | 0.10 (-0.25, 0.46) | 0.57 | 0.74 |  |
| LPC(20:4) [sn1] | pink | 0.17 (-0.17, 0.52) | 0.32 | 0.37 |  | 0.06 (-0.22, 0.35) | 0.66 | 0.80 |  |
| **LPC(20:4) [sn2]** | **pink (hub)** | 0.18 (-0.13, 0.49) | 0.25 | 0.30 |  | 0.10 (-0.23, 0.42) | 0.56 | 0.74 |  |
| LPC(20:5) [sn1] | greenyellow | -0.02 (-0.27, 0.23) | 0.88 | 0.88 |  | -0.03 (-0.28, 0.22) | 0.82 | 0.90 |  |
| LPC(20:5) [sn2] | greenyellow | -0.04 (-0.27, 0.19) | 0.75 | 0.77 |  | -0.03 (-0.27, 0.22) | 0.84 | 0.91 |  |
| LPC(22:0) [sn1] | blue | 0.28 (-0.01, 0.58) | 0.06 | 0.09 |  | -0.04 (-0.48, 0.39) | 0.85 | 0.92 |  |
| LPC(22:0) [sn2] | blue | **0.32 (0.04, 0.60)** | **0.03** | 0.05 |  | 0.06 (-0.36, 0.47) | 0.79 | 0.88 |  |
| LPC(22:1) [sn1] | green | **0.40 (0.07, 0.73)** | **0.02** | **0.04** |  | 0.20 (-0.32, 0.72) | 0.45 | 0.65 |  |
| LPC(22:1) [sn2] | green | **0.44 (0.12, 0.77)** | **0.01** | **0.03** |  | 0.33 (-0.13, 0.79) | 0.16 | 0.37 |  |
| LPC(22:4) [sn1] | pink | **0.31 (0.01, 0.62)** | **0.05** | 0.08 |  | 0.07 (-0.30, 0.44) | 0.72 | 0.84 |  |
| LPC(22:4) [sn2] | pink | **0.26 (0.01, 0.50)** | **0.04** | 0.07 |  | 0.20 (-0.20, 0.61) | 0.32 | 0.55 |  |
| LPC(22:5) (n3) [sn1] [104_sn1] | pink | 0.11 (-0.18, 0.39) | 0.46 | 0.50 |  | 0.09 (-0.21, 0.39) | 0.55 | 0.73 |  |
| LPC(22:5) [sn1] (n3)/LPC(22:5) [sn2] (n6) | pink | 0.21 (-0.06, 0.49) | 0.13 | 0.17 |  | 0.15 (-0.19, 0.49) | 0.38 | 0.60 |  |
| LPC(22:5) [sn1] (n6) | pink | 0.21 (-0.00, 0.43) | 0.06 | 0.09 |  | 0.14 (-0.12, 0.40) | 0.31 | 0.53 |  |
| LPC(22:5) [sn2] (n3) | pink | 0.13 (-0.13, 0.38) | 0.34 | 0.38 |  | 0.11 (-0.20, 0.41) | 0.50 | 0.69 |  |
| LPC(22:6) [sn1] | pink | 0.09 (-0.26, 0.45) | 0.61 | 0.64 |  | -0.11 (-0.45, 0.23) | 0.52 | 0.71 |  |
| LPC(22:6) [sn2] | pink | 0.18 (-0.15, 0.51) | 0.29 | 0.34 |  | -0.10 (-0.49, 0.29) | 0.61 | 0.76 |  |
| LPC(24:0) [sn1] | blue | **0.35 (0.07, 0.62)** | **0.01** | **0.04** |  | 0.18 (-0.34, 0.70) | 0.50 | 0.69 |  |
| LPC(24:0) [sn2] | blue | **0.34 (0.09, 0.60)** | **0.01** | **0.03** |  | 0.02 (-0.47, 0.51) | 0.95 | 0.98 |  |
| LPC(26:0) [sn1] | blue | **0.36 (0.11, 0.62)** | **0.01** | **0.03** |  | 0.12 (-0.30, 0.54) | 0.58 | 0.75 |  |
| LPC(26:0) [sn2] | blue | 0.22 (-0.04, 0.48) | 0.10 | 0.14 |  | 0.14 (-0.28, 0.55) | 0.53 | 0.71 |  |
| LPC(O-16:0) | green | 0.27 (-0.03, 0.57) | 0.07 | 0.11 |  | 0.29 (-0.11, 0.68) | 0.16 | 0.37 |  |
| LPC(O-18:0) | green | **0.29 (0.03, 0.55)** | **0.03** | 0.05 |  | 0.17 (-0.24, 0.58) | 0.42 | 0.63 |  |
| LPC(O-18:1) | green | **0.43 (0.12, 0.74)** | **0.01** | **0.03** |  | 0.27 (-0.18, 0.73) | 0.24 | 0.46 |  |
| LPC(O-20:0) | green | 0.12 (-0.14, 0.37) | 0.37 | 0.42 |  | -0.02 (-0.43, 0.40) | 0.94 | 0.97 |  |
| LPC(O-20:1) | green | **0.33 (0.02, 0.63)** | **0.03** | 0.06 |  | 0.26 (-0.15, 0.67) | 0.22 | 0.44 |  |
| LPC(O-22:0) | green | 0.10 (-0.13, 0.33) | 0.37 | 0.42 |  | 0.12 (-0.34, 0.58) | 0.62 | 0.77 |  |
| LPC(O-22:1) | green | 0.17 (-0.10, 0.45) | 0.21 | 0.26 |  | 0.25 (-0.19, 0.68) | 0.27 | 0.49 |  |
| LPC(O-24:0) | green | 0.17 (-0.08, 0.41) | 0.18 | 0.23 |  | 0.08 (-0.27, 0.43) | 0.66 | 0.80 |  |
| LPC(O-24:1) | green | 0.21 (-0.07, 0.50) | 0.15 | 0.19 |  | 0.29 (-0.12, 0.70) | 0.17 | 0.38 |  |
| LPC(O-24:2) | green | 0.19 (-0.11, 0.48) | 0.21 | 0.26 |  | 0.29 (-0.14, 0.71) | 0.18 | 0.40 |  |
| LPC(P-16:0) | green | 0.24 (-0.09, 0.57) | 0.16 | 0.20 |  | 0.14 (-0.29, 0.56) | 0.53 | 0.72 |  |
| LPC(P-17:0) (a) | green | 0.09 (-0.29, 0.46) | 0.64 | 0.67 |  | 0.03 (-0.37, 0.43) | 0.88 | 0.93 |  |
| LPC(P-17:0) (b) | green | 0.08 (-0.26, 0.41) | 0.65 | 0.68 |  | 0.08 (-0.33, 0.48) | 0.70 | 0.83 |  |
| LPC(P-18:0) | green | **0.30 (0.01, 0.60)** | **0.04** | 0.08 |  | 0.12 (-0.31, 0.54) | 0.59 | 0.76 |  |
| LPC(P-18:1) | green | **0.52 (0.20, 0.84)** | **0.00** | **0.02** |  | 0.35 (-0.10, 0.81) | 0.13 | 0.34 |  |
| LPC(P-20:0) | green | 0.13 (-0.20, 0.45) | 0.44 | 0.49 |  | 0.18 (-0.29, 0.65) | 0.44 | 0.65 |  |
| PE(15-MHDA_18:1) | turquoise | **0.57 (0.17, 0.97)** | **0.01** | **0.03** |  | **0.43 (0.19, 0.66)** | **0.00** | **0.02** |  |
| PE(15-MHDA_18:2) | turquoise | **0.65 (0.22, 1.07)** | **0.00** | **0.02** |  | **0.33 (0.12, 0.53)** | **0.00** | **0.04** |  |
| PE(15-MHDA_20:4) | turquoise | **0.47 (0.09, 0.84)** | **0.02** | **0.04** |  | **0.36 (0.12, 0.60)** | **0.00** | 0.06 |  |
| PE(15-MHDA_22:6) | brown | **0.55 (0.13, 0.96)** | **0.01** | **0.03** |  | **0.31 (0.09, 0.53)** | **0.01** | 0.09 |  |
| PE(16:0_16:0) | turquoise | **0.38 (0.05, 0.70)** | **0.02** | **0.05** |  | **0.44 (0.18, 0.70)** | **0.00** | **0.04** |  |
| PE(16:0_16:1) | turquoise | **0.76 (0.33, 1.19)** | **0.00** | **0.01** |  | **0.38 (0.16, 0.59)** | **0.00** | **0.02** |  |
| PE(16:0_18:1) | turquoise | **0.62 (0.27, 0.97)** | **0.00** | **0.01** |  | **0.34 (0.16, 0.51)** | **0.00** | **0.02** |  |
| PE(16:0_18:2) | turquoise | **0.71 (0.26, 1.16)** | **0.00** | **0.02** |  | **0.38 (0.17, 0.59)** | **0.00** | **0.02** |  |
| PE(16:0_18:3) (a) | turquoise | **0.69 (0.28, 1.11)** | **0.00** | **0.02** |  | **0.30 (0.16, 0.45)** | **0.00** | **0.01** |  |
| PE(16:0_18:3) (b) | turquoise | **0.73 (0.31, 1.15)** | **0.00** | **0.02** |  | **0.44 (0.21, 0.66)** | **0.00** | **0.02** |  |
| PE(16:0_20:3) | turquoise | **0.60 (0.18, 1.02)** | **0.01** | **0.02** |  | **0.42 (0.19, 0.65)** | **0.00** | **0.02** |  |
| PE(16:0_20:4) | turquoise | **0.74 (0.29, 1.19)** | **0.00** | **0.02** |  | **0.56 (0.27, 0.86)** | **0.00** | **0.02** |  |
| PE(16:0_20:5) | turquoise | 0.37 (-0.05, 0.79) | 0.08 | 0.12 |  | **0.24 (0.04, 0.45)** | **0.02** | 0.15 |  |
| PE(16:0_22:6) | brown | **0.56 (0.04, 1.07)** | **0.03** | 0.06 |  | **0.30 (0.03, 0.57)** | **0.03** | 0.17 |  |
| PE(16:1_18:2) | turquoise | **0.62 (0.18, 1.06)** | **0.01** | **0.03** |  | **0.34 (0.12, 0.55)** | **0.00** | **0.04** |  |
| PE(16:1_20:4) | turquoise | **0.67 (0.18, 1.16)** | **0.01** | **0.03** |  | **0.44 (0.19, 0.70)** | **0.00** | **0.03** |  |
| PE(17:0_18:1) | turquoise | **0.60 (0.21, 0.99)** | **0.00** | **0.02** |  | **0.34 (0.13, 0.55)** | **0.00** | **0.04** |  |
| PE(17:0_18:2) | turquoise | **0.69 (0.25, 1.13)** | **0.00** | **0.02** |  | **0.33 (0.12, 0.54)** | **0.00** | **0.04** |  |
| PE(17:0_20:4) | turquoise | **0.55 (0.20, 0.89)** | **0.00** | **0.02** |  | **0.48 (0.21, 0.75)** | **0.00** | **0.02** |  |
| PE(17:0_22:6) | brown | **0.44 (0.04, 0.83)** | **0.03** | 0.06 |  | 0.20 (-0.02, 0.41) | 0.07 | 0.26 |  |
| PE(18:0_18:1) | turquoise | **0.61 (0.24, 0.97)** | **0.00** | **0.02** |  | **0.31 (0.13, 0.50)** | **0.00** | **0.04** |  |
| **PE(18:0_18:2)** | **turquoise (hub)** | **0.72 (0.26, 1.19)** | **0.00** | **0.02** |  | **0.29 (0.09, 0.48)** | **0.00** | 0.07 |  |
| PE(18:0_20:3) (a) | turquoise | **0.53 (0.15, 0.92)** | **0.01** | **0.03** |  | **0.42 (0.17, 0.67)** | **0.00** | **0.04** |  |
| PE(18:0_20:3) (b) | turquoise | **0.57 (0.27, 0.87)** | **0.00** | **0.01** |  | **0.46 (0.09, 0.83)** | **0.01** | 0.13 |  |
| PE(18:0_20:4) | turquoise | **0.54 (0.22, 0.87)** | **0.00** | **0.02** |  | **0.47 (0.18, 0.77)** | **0.00** | **0.04** |  |
| PE(18:0_22:4) | turquoise | **0.39 (0.14, 0.64)** | **0.00** | **0.02** |  | **0.29 (0.07, 0.51)** | **0.01** | 0.11 |  |
| PE(18:0_22:5) (n3) | turquoise | **0.46 (0.13, 0.79)** | **0.01** | **0.03** |  | **0.30 (0.10, 0.51)** | **0.00** | 0.07 |  |
| PE(18:0_22:5) (n6) | turquoise | **0.52 (0.19, 0.85)** | **0.00** | **0.02** |  | **0.28 (0.07, 0.50)** | **0.01** | 0.11 |  |
| PE(18:0_22:6) | brown | **0.44 (0.05, 0.84)** | **0.03** | 0.06 |  | 0.22 (-0.02, 0.45) | 0.07 | 0.26 |  |
| PE(18:1_18:1) | turquoise | **0.65 (0.23, 1.07)** | **0.00** | **0.02** |  | **0.30 (0.12, 0.48)** | **0.00** | **0.04** |  |
| PE(18:1_18:2) | turquoise | **0.75 (0.26, 1.24)** | **0.00** | **0.02** |  | **0.32 (0.12, 0.52)** | **0.00** | **0.04** |  |
| PE(18:1_22:6) (a) | turquoise | **0.60 (0.10, 1.09)** | **0.02** | **0.04** |  | **0.31 (0.09, 0.53)** | **0.01** | 0.09 |  |
| PE(18:1_22:6) (b) | turquoise | **0.58 (0.13, 1.04)** | **0.01** | **0.03** |  | **0.27 (0.07, 0.47)** | **0.01** | 0.11 |  |
| PE(20:0_20:4) | magenta | **0.21 (0.03, 0.40)** | **0.02** | **0.05** |  | 0.09 (-0.12, 0.29) | 0.41 | 0.63 |  |
| PE(36:0) | grey | **-0.22 (-0.43, -0.01)** | **0.04** | 0.08 |  | 0.31 (-0.84, 1.45) | 0.60 | 0.76 |  |
| PE(38:5) (a) | turquoise | **0.61 (0.19, 1.03)** | **0.00** | **0.02** |  | **0.42 (0.19, 0.65)** | **0.00** | **0.02** |  |
| PE(38:5) (b) | turquoise | **0.66 (0.26, 1.06)** | **0.00** | **0.02** |  | **0.36 (0.13, 0.59)** | **0.00** | **0.05** |  |
| PE(O-16:0/18:2) | turquoise | **0.61 (0.15, 1.07)** | **0.01** | **0.03** |  | **0.32 (0.10, 0.54)** | **0.00** | 0.07 |  |
| PE(O-16:0/20:3) | turquoise | **0.39 (0.11, 0.67)** | **0.01** | **0.03** |  | **0.22 (0.04, 0.39)** | **0.02** | 0.14 |  |
| PE(O-16:0/20:4) | turquoise | **0.38 (0.09, 0.67)** | **0.01** | **0.03** |  | 0.20 (-0.03, 0.44) | 0.09 | 0.29 |  |
| PE(O-16:0/22:4) | turquoise | **0.37 (0.18, 0.56)** | **0.00** | **0.01** |  | **0.38 (0.04, 0.72)** | **0.03** | 0.18 |  |
| PE(O-16:0/22:6) | turquoise | **0.29 (0.01, 0.56)** | **0.04** | 0.07 |  | 0.13 (-0.12, 0.38) | 0.31 | 0.54 |  |
| PE(O-18:0/20:4) | turquoise | **0.39 (0.12, 0.66)** | **0.00** | **0.02** |  | 0.19 (-0.01, 0.40) | 0.07 | 0.25 |  |
| PE(O-18:0/22:5) | turquoise | **0.53 (0.19, 0.86)** | **0.00** | **0.02** |  | **0.22 (0.04, 0.40)** | **0.02** | 0.14 |  |
| PE(O-18:0/22:6) | turquoise | **0.36 (0.07, 0.65)** | **0.01** | **0.04** |  | 0.18 (-0.07, 0.42) | 0.16 | 0.37 |  |
| PE(O-18:1/18:2) | turquoise | **0.61 (0.18, 1.04)** | **0.01** | **0.03** |  | 0.15 (-0.02, 0.32) | 0.08 | 0.28 |  |
| PE(O-18:1/22:6) | turquoise | **0.48 (0.09, 0.87)** | **0.02** | **0.04** |  | 0.21 (-0.06, 0.47) | 0.13 | 0.34 |  |
| PE(O-34:1) | turquoise | **0.64 (0.22, 1.05)** | **0.00** | **0.02** |  | **0.32 (0.07, 0.57)** | **0.01** | 0.12 |  |
| PE(O-36:5) | turquoise | **0.49 (0.13, 0.85)** | **0.01** | **0.03** |  | **0.19 (0.05, 0.32)** | **0.01** | 0.09 |  |
| PE(O-38:5) (a) | turquoise | **0.53 (0.12, 0.94)** | **0.01** | **0.03** |  | **0.25 (0.02, 0.48)** | **0.03** | 0.18 |  |
| PE(O-38:5) (b) | turquoise | **0.47 (0.14, 0.81)** | **0.01** | **0.03** |  | 0.21 (-0.05, 0.46) | 0.12 | 0.33 |  |
| PE(P-15:0/20:4) (a) | turquoise | 0.44 (-0.04, 0.91) | 0.07 | 0.11 |  | **0.19 (0.03, 0.35)** | **0.02** | 0.15 |  |
| PE(P-15:0/20:4) (b) | turquoise | 0.26 (-0.04, 0.56) | 0.09 | 0.13 |  | 0.13 (-0.01, 0.27) | 0.06 | 0.24 |  |
| PE(P-15:0/22:6) (a) | turquoise | 0.50 (-0.02, 1.02) | 0.06 | 0.09 |  | 0.18 (-0.06, 0.41) | 0.14 | 0.35 |  |
| PE(P-15:0/22:6) (b) | turquoise | **0.38 (0.16, 0.60)** | **0.00** | **0.02** |  | **0.33 (0.06, 0.59)** | **0.02** | 0.14 |  |
| PE(P-16:0/18:1) | turquoise | **0.31 (0.06, 0.57)** | **0.02** | **0.04** |  | 0.28 (-0.01, 0.58) | 0.06 | 0.24 |  |
| PE(P-16:0/18:2) | turquoise | **0.60 (0.10, 1.09)** | **0.02** | **0.04** |  | 0.17 (-0.06, 0.40) | 0.15 | 0.35 |  |
| PE(P-16:0/18:3) | turquoise | 0.31 (-0.16, 0.77) | 0.19 | 0.24 |  | 0.14 (-0.09, 0.37) | 0.24 | 0.46 |  |
| PE(P-16:0/20:3) (a) | turquoise | 0.33 (-0.00, 0.67) | 0.05 | 0.08 |  | 0.22 (-0.07, 0.51) | 0.13 | 0.34 |  |
| PE(P-16:0/20:3) (b) | turquoise | 0.36 (-0.00, 0.71) | 0.05 | 0.08 |  | 0.15 (-0.11, 0.40) | 0.27 | 0.49 |  |
| PE(P-16:0/20:4) | turquoise | **0.36 (0.06, 0.67)** | **0.02** | **0.04** |  | 0.19 (-0.07, 0.44) | 0.15 | 0.36 |  |
| PE(P-16:0/20:5) | turquoise | 0.39 (-0.04, 0.82) | 0.08 | 0.11 |  | 0.16 (-0.02, 0.34) | 0.07 | 0.26 |  |
| PE(P-16:0/22:4) | magenta | **0.32 (0.09, 0.56)** | **0.01** | **0.03** |  | 0.33 (-0.03, 0.69) | 0.08 | 0.26 |  |
| PE(P-16:0/22:5) (n3) | turquoise | **0.49 (0.09, 0.90)** | **0.02** | **0.04** |  | **0.25 (0.02, 0.48)** | **0.04** | 0.19 |  |
| PE(P-16:0/22:5) (n6) | turquoise | **0.21 (0.04, 0.39)** | **0.02** | **0.04** |  | 0.15 (-0.18, 0.49) | 0.38 | 0.60 |  |
| PE(P-16:0/22:6) | turquoise | **0.39 (0.11, 0.68)** | **0.01** | **0.03** |  | 0.18 (-0.13, 0.50) | 0.25 | 0.47 |  |
| PE(P-17:0/20:4) (a) | turquoise | 0.31 (-0.07, 0.69) | 0.11 | 0.16 |  | 0.11 (-0.13, 0.35) | 0.37 | 0.60 |  |
| PE(P-17:0/20:4) (b) | turquoise | **0.43 (0.09, 0.78)** | **0.01** | **0.04** |  | 0.18 (-0.06, 0.42) | 0.15 | 0.36 |  |
| PE(P-17:0/22:6) (a) | turquoise | 0.19 (-0.16, 0.53) | 0.29 | 0.34 |  | 0.01 (-0.26, 0.28) | 0.94 | 0.97 |  |
| PE(P-17:0/22:6) (b) | turquoise | **0.43 (0.08, 0.78)** | **0.02** | **0.04** |  | 0.17 (-0.10, 0.44) | 0.21 | 0.44 |  |
| PE(P-18:0/18:1) | turquoise | **0.46 (0.12, 0.80)** | **0.01** | **0.03** |  | **0.26 (0.04, 0.48)** | **0.02** | 0.16 |  |
| PE(P-18:0/18:2) | turquoise | **0.69 (0.16, 1.21)** | **0.01** | **0.03** |  | **0.19 (0.00, 0.38)** | **0.05** | 0.23 |  |
| PE(P-18:0/18:3) | turquoise | **0.44 (0.01, 0.86)** | **0.05** | 0.08 |  | 0.17 (-0.00, 0.35) | 0.05 | 0.23 |  |
| PE(P-18:0/20:3) (a) | turquoise | **0.38 (0.00, 0.77)** | **0.05** | 0.08 |  | **0.27 (0.03, 0.51)** | **0.03** | 0.17 |  |
| PE(P-18:0/20:3) (b) | turquoise | **0.49 (0.11, 0.86)** | **0.01** | **0.03** |  | 0.20 (-0.01, 0.42) | 0.07 | 0.25 |  |
| PE(P-18:0/20:4) | turquoise | **0.40 (0.08, 0.72)** | **0.01** | **0.04** |  | 0.21 (-0.02, 0.44) | 0.08 | 0.26 |  |
| PE(P-18:0/20:5) | turquoise | **0.47 (0.01, 0.93)** | **0.05** | 0.08 |  | 0.15 (-0.03, 0.32) | 0.10 | 0.30 |  |
| PE(P-18:0/22:4) | magenta | **0.30 (0.08, 0.52)** | **0.01** | **0.03** |  | **0.27 (0.02, 0.52)** | **0.04** | 0.19 |  |
| PE(P-18:0/22:5) (n3) | turquoise | **0.50 (0.09, 0.91)** | **0.02** | **0.04** |  | **0.23 (0.02, 0.43)** | **0.03** | 0.18 |  |
| PE(P-18:0/22:5) (n6) | turquoise | **0.34 (0.13, 0.55)** | **0.00** | **0.02** |  | **0.31 (0.00, 0.61)** | **0.05** | 0.23 |  |
| PE(P-18:0/22:6) | turquoise | **0.42 (0.07, 0.77)** | **0.02** | **0.04** |  | 0.22 (-0.06, 0.49) | 0.12 | 0.34 |  |
| PE(P-18:1/18:1) | turquoise | **0.56 (0.14, 0.97)** | **0.01** | **0.03** |  | **0.28 (0.02, 0.53)** | **0.03** | 0.18 |  |
| PE(P-18:1/18:1) (a) | turquoise | **0.55 (0.14, 0.96)** | **0.01** | **0.03** |  | **0.27 (0.01, 0.52)** | **0.04** | 0.20 |  |
| PE(P-18:1/18:1) (b) | turquoise | **0.55 (0.11, 0.99)** | **0.01** | **0.04** |  | **0.27 (0.03, 0.50)** | **0.03** | 0.16 |  |
| PE(P-18:1/18:2) (a) | turquoise | **0.79 (0.22, 1.36)** | **0.01** | **0.03** |  | **0.21 (0.01, 0.41)** | **0.04** | 0.20 |  |
| PE(P-18:1/18:2) (b) | turquoise | **0.59 (0.10, 1.07)** | **0.02** | **0.04** |  | **0.16 (0.00, 0.31)** | **0.05** | 0.23 |  |
| PE(P-18:1/18:3) | turquoise | 0.15 (-0.20, 0.50) | 0.39 | 0.43 |  | 0.06 (-0.09, 0.21) | 0.41 | 0.62 |  |
| PE(P-18:1/20:3) (a) | turquoise | 0.38 (-0.02, 0.78) | 0.06 | 0.10 |  | **0.31 (0.04, 0.58)** | **0.02** | 0.16 |  |
| PE(P-18:1/20:3) (b) | turquoise | **0.58 (0.17, 0.98)** | **0.01** | **0.03** |  | 0.19 (-0.03, 0.42) | 0.09 | 0.29 |  |
| PE(P-18:1/20:4) (a) | turquoise | **0.51 (0.10, 0.93)** | **0.02** | **0.04** |  | 0.25 (-0.02, 0.52) | 0.07 | 0.26 |  |
| PE(P-18:1/20:4) (b) | turquoise | **0.56 (0.11, 1.00)** | **0.01** | **0.04** |  | **0.23 (0.02, 0.43)** | **0.03** | 0.18 |  |
| PE(P-18:1/20:5) (a) | turquoise | 0.46 (-0.02, 0.94) | 0.06 | 0.09 |  | 0.13 (-0.03, 0.29) | 0.10 | 0.30 |  |
| PE(P-18:1/20:5) (b) | turquoise | **0.38 (0.01, 0.75)** | **0.05** | 0.08 |  | 0.09 (-0.03, 0.20) | 0.13 | 0.34 |  |
| PE(P-18:1/22:4) | magenta | **0.32 (0.09, 0.55)** | **0.01** | **0.03** |  | 0.36 (-0.00, 0.72) | 0.05 | 0.23 |  |
| PE(P-18:1/22:5) (a) | turquoise | 0.46 (-0.02, 0.94) | 0.06 | 0.09 |  | **0.30 (0.07, 0.53)** | **0.01** | 0.11 |  |
| PE(P-18:1/22:5) (b) | turquoise | **0.23 (0.01, 0.45)** | **0.04** | 0.07 |  | 0.19 (-0.10, 0.49) | 0.19 | 0.41 |  |
| PE(P-18:1/22:6) (a) | turquoise | **0.46 (0.06, 0.86)** | **0.02** | 0.05 |  | 0.14 (-0.16, 0.45) | 0.35 | 0.58 |  |
| PE(P-18:1/22:6) (b) | turquoise | **0.58 (0.14, 1.02)** | **0.01** | **0.03** |  | 0.19 (-0.07, 0.46) | 0.15 | 0.36 |  |
| PE(P-19:0/20:4) (a) | turquoise | **0.32 (0.04, 0.61)** | **0.03** | 0.05 |  | 0.15 (-0.09, 0.39) | 0.23 | 0.46 |  |
| PE(P-19:0/20:4) (b) | turquoise | **0.34 (0.07, 0.60)** | **0.01** | **0.04** |  | 0.21 (-0.04, 0.46) | 0.10 | 0.30 |  |
| PE(P-20:0/18:1) | turquoise | 0.38 (-0.10, 0.85) | 0.12 | 0.16 |  | 0.24 (-0.06, 0.53) | 0.11 | 0.33 |  |
| PE(P-20:0/18:2) | turquoise | **0.60 (0.05, 1.15)** | **0.03** | 0.06 |  | **0.23 (0.03, 0.43)** | **0.02** | 0.16 |  |
| PE(P-20:0/20:4) | turquoise | 0.27 (-0.04, 0.58) | 0.09 | 0.13 |  | 0.03 (-0.21, 0.28) | 0.78 | 0.88 |  |
| PE(P-20:0/22:6) | turquoise | **0.49 (0.11, 0.86)** | **0.01** | **0.03** |  | 0.22 (-0.04, 0.49) | 0.10 | 0.30 |  |
| PE(P-20:1/20:4) | turquoise | **0.38 (0.12, 0.64)** | **0.00** | **0.02** |  | 0.18 (-0.06, 0.41) | 0.13 | 0.34 |  |
| PE(P-20:1/22:6) | turquoise | **0.53 (0.20, 0.86)** | **0.00** | **0.02** |  | **0.30 (0.06, 0.54)** | **0.01** | 0.12 |  |
| LPE(16:0) [sn1] | green | **0.40 (0.09, 0.72)** | **0.01** | **0.03** |  | **0.58 (0.16, 0.99)** | **0.01** | 0.09 |  |
| LPE(16:0) [sn2] | green | **0.32 (0.02, 0.62)** | **0.04** | 0.07 |  | **0.63 (0.23, 1.02)** | **0.00** | **0.05** |  |
| LPE(17:0) [sn1] | grey | 0.20 (-0.03, 0.42) | 0.08 | 0.12 |  | -0.01 (-0.36, 0.34) | 0.95 | 0.98 |  |
| LPE(17:0) [sn2] | grey | 0.24 (-0.02, 0.50) | 0.07 | 0.11 |  | 0.08 (-0.41, 0.56) | 0.76 | 0.87 |  |
| LPE(18:0) [sn1] | green | **0.57 (0.28, 0.86)** | **0.00** | **0.01** |  | **0.56 (0.19, 0.94)** | **0.00** | 0.06 |  |
| LPE(18:0) [sn2] | green | **0.61 (0.32, 0.91)** | **0.00** | **0.00** |  | **0.52 (0.15, 0.89)** | **0.01** | 0.09 |  |
| LPE(18:1) [sn1] | greenyellow | **0.48 (0.11, 0.85)** | **0.01** | **0.03** |  | **0.53 (0.17, 0.89)** | **0.00** | 0.07 |  |
| LPE(18:1) [sn2] | greenyellow | **0.49 (0.14, 0.85)** | **0.01** | **0.03** |  | **0.48 (0.12, 0.84)** | **0.01** | 0.11 |  |
| LPE(18:2) [sn1] | greenyellow | 0.10 (-0.28, 0.48) | 0.61 | 0.64 |  | 0.10 (-0.17, 0.36) | 0.47 | 0.67 |  |
| LPE(18:2) [sn2] | greenyellow | 0.18 (-0.19, 0.55) | 0.35 | 0.40 |  | 0.17 (-0.10, 0.44) | 0.22 | 0.45 |  |
| LPE(20:4) [sn1] | pink | 0.02 (-0.26, 0.29) | 0.91 | 0.91 |  | 0.02 (-0.24, 0.29) | 0.87 | 0.93 |  |
| LPE(20:4) [sn2] | pink | 0.04 (-0.21, 0.29) | 0.76 | 0.78 |  | 0.08 (-0.21, 0.38) | 0.58 | 0.75 |  |
| LPE(22:6) [sn1] | pink | -0.05 (-0.44, 0.35) | 0.82 | 0.84 |  | -0.01 (-0.26, 0.24) | 0.93 | 0.97 |  |
| LPE(22:6) [sn2] | pink | 0.06 (-0.37, 0.48) | 0.79 | 0.81 |  | 0.10 (-0.14, 0.34) | 0.42 | 0.64 |  |
| LPE(P-16:0) | magenta | 0.28 (-0.01, 0.58) | 0.06 | 0.09 |  | 0.15 (-0.11, 0.41) | 0.25 | 0.47 |  |
| LPE(P-18:0) | magenta | 0.25 (-0.01, 0.51) | 0.06 | 0.09 |  | 0.23 (-0.10, 0.55) | 0.17 | 0.38 |  |
| LPE(P-18:1) | magenta | 0.21 (-0.03, 0.46) | 0.09 | 0.13 |  | 0.17 (-0.08, 0.41) | 0.18 | 0.40 |  |
| LPE(P-20:0) | grey | 0.20 (-0.03, 0.43) | 0.08 | 0.12 |  | 0.23 (-0.14, 0.60) | 0.22 | 0.44 |  |
| PI (38:5) (b) | turquoise | 0.35 (-0.03, 0.73) | 0.07 | 0.11 |  | 0.47 (-0.01, 0.95) | 0.05 | 0.23 |  |
| PI(15-MHDA_20:4)\PI(17:0_20:4) | brown | **0.44 (0.13, 0.75)** | **0.00** | **0.02** |  | **0.49 (0.06, 0.91)** | **0.02** | 0.16 |  |
| PI(16:0_16:1) | turquoise | **0.82 (0.39, 1.26)** | **0.00** | **0.01** |  | **0.56 (0.26, 0.86)** | **0.00** | **0.02** |  |
| PI(16:0_20:3) (a) | turquoise | 0.37 (-0.01, 0.75) | 0.06 | 0.09 |  | **0.49 (0.14, 0.84)** | **0.01** | 0.09 |  |
| PI(16:0_20:3) (b) | grey | **0.32 (0.05, 0.59)** | **0.02** | **0.04** |  | 0.21 (-0.10, 0.52) | 0.18 | 0.40 |  |
| PI(16:0_20:4) | brown | **0.36 (0.08, 0.64)** | **0.01** | **0.03** |  | 0.41 (-0.01, 0.84) | 0.06 | 0.23 |  |
| PI(16:0/16:0) | turquoise | **0.70 (0.23, 1.17)** | **0.00** | **0.02** |  | **0.28 (0.07, 0.49)** | **0.01** | 0.11 |  |
| PI(17:0_18:1) | turquoise | **0.54 (0.17, 0.91)** | **0.00** | **0.02** |  | **0.35 (0.12, 0.59)** | **0.00** | 0.05 |  |
| PI(17:0_18:2) | turquoise | **0.61 (0.20, 1.01)** | **0.00** | **0.02** |  | **0.31 (0.08, 0.54)** | **0.01** | 0.10 |  |
| PI(18:0_18:1) | turquoise | **0.80 (0.32, 1.28)** | **0.00** | **0.02** |  | **0.29 (0.10, 0.48)** | **0.00** | 0.06 |  |
| PI(18:0_20:2) | turquoise | 0.34 (-0.03, 0.71) | 0.07 | 0.11 |  | **0.30 (0.03, 0.56)** | **0.03** | 0.17 |  |
| PI(18:0_20:3) (a) | grey | -0.09 (-0.32, 0.13) | 0.42 | 0.46 |  | 0.10 (-0.22, 0.41) | 0.55 | 0.74 |  |
| PI(18:0_20:3) (b) | grey | **0.27 (0.02, 0.51)** | **0.04** | 0.06 |  | 0.24 (-0.08, 0.57) | 0.14 | 0.35 |  |
| PI(18:0_20:4) | brown | **0.44 (0.13, 0.75)** | **0.01** | **0.02** |  | **0.46 (0.03, 0.89)** | **0.04** | 0.19 |  |
| PI(18:0_22:4) | turquoise | **0.37 (0.04, 0.70)** | **0.03** | 0.05 |  | **0.60 (0.15, 1.05)** | **0.01** | 0.11 |  |
| PI(18:0_22:5) (n3) | brown | 0.25 (-0.10, 0.61) | 0.16 | 0.20 |  | **0.31 (0.02, 0.60)** | **0.03** | 0.19 |  |
| PI(18:0_22:5) (n6) | grey | 0.09 (-0.10, 0.28) | 0.37 | 0.42 |  | 0.04 (-0.22, 0.31) | 0.74 | 0.85 |  |
| PI(18:0_22:6) | turquoise | 0.37 (-0.04, 0.78) | 0.07 | 0.11 |  | 0.30 (-0.03, 0.62) | 0.08 | 0.26 |  |
| PI(18:1_18:2) | turquoise | **0.67 (0.22, 1.12)** | **0.00** | **0.02** |  | **0.48 (0.18, 0.77)** | **0.00** | **0.04** |  |
| PI(20:0_20:4) | brown | 0.29 (-0.09, 0.67) | 0.13 | 0.17 |  | **0.44 (0.06, 0.81)** | **0.02** | 0.16 |  |
| PI(34:0) | turquoise | **0.78 (0.30, 1.25)** | **0.00** | **0.02** |  | **0.29 (0.08, 0.50)** | **0.01** | 0.09 |  |
| PI(34:1) | turquoise | **0.75 (0.29, 1.21)** | **0.00** | **0.02** |  | **0.46 (0.20, 0.71)** | **0.00** | **0.02** |  |
| PI(36:2) | turquoise | **0.71 (0.24, 1.18)** | **0.00** | **0.02** |  | **0.35 (0.11, 0.58)** | **0.00** | 0.07 |  |
| PI(37:6) | turquoise | 0.14 (-0.20, 0.47) | 0.42 | 0.46 |  | 0.11 (-0.15, 0.37) | 0.40 | 0.62 |  |
| PI(38:5) (a) | grey | 0.21 (-0.01, 0.43) | 0.06 | 0.09 |  | **0.47 (0.06, 0.88)** | **0.03** | 0.16 |  |
| PI(38:6) | brown | 0.31 (-0.01, 0.64) | 0.06 | 0.09 |  | 0.29 (-0.07, 0.65) | 0.11 | 0.33 |  |
| PI(39:6) | brown | 0.28 (-0.04, 0.60) | 0.09 | 0.13 |  | 0.20 (-0.07, 0.48) | 0.15 | 0.36 |  |
| PIP1(38:4) | grey | -0.04 (-0.26, 0.18) | 0.73 | 0.75 |  | **0.43 (0.07, 0.79)** | **0.02** | 0.15 |  |
| LPI(18:0) [sn1] | grey | 0.14 (-0.08, 0.37) | 0.21 | 0.25 |  | 0.16 (-0.12, 0.44) | 0.27 | 0.49 |  |
| LPI(18:0) [sn2] | grey | 0.15 (-0.08, 0.37) | 0.20 | 0.25 |  | 0.14 (-0.09, 0.37) | 0.24 | 0.46 |  |
| LPI(18:1) [sn1] | blue | **0.33 (0.05, 0.60)** | **0.02** | **0.04** |  | 0.24 (-0.07, 0.56) | 0.13 | 0.34 |  |
| LPI(18:1) [sn2] | grey | 0.09 (-0.16, 0.35) | 0.47 | 0.51 |  | 0.11 (-0.11, 0.32) | 0.33 | 0.56 |  |
| LPI(18:2) [sn1] | grey | **0.51 (0.20, 0.81)** | **0.00** | **0.02** |  | **0.37 (0.03, 0.71)** | **0.03** | 0.18 |  |
| LPI(18:2) [sn2] | grey | **0.45 (0.13, 0.76)** | **0.01** | **0.03** |  | 0.23 (-0.07, 0.54) | 0.13 | 0.34 |  |
| LPI(20:4) [sn1] | pink | 0.35 (-0.01, 0.70) | 0.05 | 0.09 |  | 0.07 (-0.33, 0.46) | 0.75 | 0.86 |  |
| LPI(20:4) [sn2] | pink | **0.41 (0.14, 0.69)** | **0.00** | **0.02** |  | 0.14 (-0.32, 0.60) | 0.55 | 0.73 |  |
| PS(36:1) | magenta | **0.27 (0.01, 0.53)** | **0.04** | 0.07 |  | 0.09 (-0.06, 0.25) | 0.24 | 0.46 |  |
| PS(36:2) | magenta | 0.19 (-0.03, 0.42) | 0.09 | 0.14 |  | 0.15 (-0.00, 0.30) | 0.05 | 0.23 |  |
| **PS(38:3)** | **magenta (hub)** | 0.22 (-0.02, 0.46) | 0.08 | 0.12 |  | 0.12 (-0.01, 0.26) | 0.07 | 0.25 |  |
| PS(38:4) | magenta | 0.32 (-0.02, 0.66) | 0.06 | 0.10 |  | 0.12 (-0.03, 0.27) | 0.13 | 0.34 |  |
| PS(38:5) | magenta | 0.21 (-0.03, 0.45) | 0.08 | 0.12 |  | **0.26 (0.03, 0.48)** | **0.02** | 0.16 |  |
| PS(40:5) | magenta | 0.19 (-0.01, 0.40) | 0.06 | 0.10 |  | **0.17 (0.03, 0.31)** | **0.01** | 0.13 |  |
| PS(40:6) | magenta | **0.26 (0.04, 0.47)** | **0.02** | **0.04** |  | **0.14 (0.00, 0.28)** | **0.05** | 0.23 |  |
| PG(34:1) | brown | **0.41 (0.08, 0.74)** | **0.01** | **0.04** |  | **0.20 (0.01, 0.39)** | **0.04** | 0.20 |  |
| PG(36:1) | brown | **0.32 (0.02, 0.62)** | **0.04** | 0.06 |  | 0.19 (-0.02, 0.40) | 0.07 | 0.25 |  |
| PG(36:2) | brown | 0.18 (-0.06, 0.42) | 0.14 | 0.19 |  | 0.19 (-0.06, 0.44) | 0.14 | 0.34 |  |
| CE(14:0) | red | 0.03 (-0.29, 0.36) | 0.84 | 0.85 |  | -0.15 (-0.58, 0.28) | 0.48 | 0.68 |  |
| CE(15:0) | red | 0.08 (-0.25, 0.42) | 0.63 | 0.66 |  | -0.11 (-0.51, 0.29) | 0.60 | 0.76 |  |
| CE(16:0) | blue | **0.34 (0.13, 0.56)** | **0.00** | **0.02** |  | 0.10 (-0.56, 0.76) | 0.77 | 0.87 |  |
| CE(16:1) | red | **0.51 (0.08, 0.93)** | **0.02** | **0.04** |  | 0.14 (-0.25, 0.53) | 0.47 | 0.68 |  |
| CE(16:2) | brown | 0.00 (-0.31, 0.31) | 0.99 | 0.99 |  | -0.04 (-0.34, 0.27) | 0.81 | 0.89 |  |
| CE(17:0) | red | 0.24 (-0.03, 0.50) | 0.08 | 0.11 |  | 0.16 (-0.16, 0.48) | 0.34 | 0.56 |  |
| CE(17:1) | red | **0.41 (0.05, 0.76)** | **0.03** | 0.05 |  | 0.06 (-0.30, 0.43) | 0.74 | 0.85 |  |
| CE(18:0) | red | **0.20 (0.01, 0.39)** | **0.04** | 0.07 |  | 0.08 (-0.28, 0.44) | 0.66 | 0.80 |  |
| CE(18:1) | blue | **0.39 (0.14, 0.63)** | **0.00** | **0.02** |  | 0.19 (-0.25, 0.64) | 0.39 | 0.62 |  |
| CE(18:2) | turquoise | **0.51 (0.19, 0.82)** | **0.00** | **0.02** |  | 0.15 (-0.22, 0.51) | 0.43 | 0.64 |  |
| CE(18:3) | red | **0.33 (0.02, 0.64)** | **0.04** | 0.06 |  | 0.06 (-0.23, 0.36) | 0.67 | 0.81 |  |
| CE(20:0) | red | 0.10 (-0.08, 0.27) | 0.28 | 0.33 |  | 0.04 (-0.23, 0.31) | 0.76 | 0.86 |  |
| CE(20:1) | red | 0.11 (-0.06, 0.28) | 0.21 | 0.25 |  | 0.05 (-0.29, 0.39) | 0.78 | 0.88 |  |
| CE(20:2) | red | **0.26 (0.06, 0.45)** | **0.01** | **0.03** |  | 0.11 (-0.32, 0.55) | 0.61 | 0.76 |  |
| CE(20:3) | red | **0.38 (0.05, 0.71)** | **0.03** | 0.05 |  | -0.05 (-0.53, 0.43) | 0.83 | 0.90 |  |
| CE(20:4) | brown | -0.18 (-0.56, 0.20) | 0.36 | 0.41 |  | -0.20 (-0.49, 0.10) | 0.19 | 0.42 |  |
| CE(20:5) | brown | -0.20 (-0.45, 0.06) | 0.13 | 0.17 |  | -0.11 (-0.30, 0.09) | 0.28 | 0.51 |  |
| CE(22:0) | red | 0.12 (-0.05, 0.29) | 0.17 | 0.21 |  | 0.08 (-0.18, 0.33) | 0.54 | 0.73 |  |
| CE(22:1) | red | 0.10 (-0.07, 0.27) | 0.26 | 0.31 |  | 0.04 (-0.26, 0.34) | 0.80 | 0.89 |  |
| CE(22:4) | red | **0.28 (0.00, 0.56)** | **0.05** | 0.08 |  | -0.08 (-0.46, 0.29) | 0.65 | 0.80 |  |
| CE(22:5) (n3) | grey | 0.22 (-0.13, 0.57) | 0.22 | 0.27 |  | -0.20 (-0.56, 0.17) | 0.28 | 0.51 |  |
| CE(22:5) (n6) | pink | 0.31 (-0.01, 0.63) | 0.06 | 0.09 |  | -0.05 (-0.43, 0.33) | 0.78 | 0.88 |  |
| CE(22:6) | grey | 0.21 (-0.15, 0.57) | 0.26 | 0.31 |  | -0.20 (-0.58, 0.17) | 0.28 | 0.51 |  |
| CE(24:0) | red | 0.13 (-0.04, 0.31) | 0.14 | 0.18 |  | 0.05 (-0.27, 0.38) | 0.74 | 0.85 |  |
| **CE(24:1)** | **red (hub)** | 0.13 (-0.04, 0.31) | 0.13 | 0.18 |  | 0.05 (-0.29, 0.39) | 0.79 | 0.88 |  |
| CE(24:4) | red | 0.24 (-0.07, 0.56) | 0.13 | 0.18 |  | 0.08 (-0.14, 0.29) | 0.47 | 0.68 |  |
| CE(24:5) | red | 0.03 (-0.23, 0.29) | 0.83 | 0.84 |  | 0.03 (-0.15, 0.22) | 0.73 | 0.85 |  |
| CE(24:6) | red | 0.20 (-0.11, 0.51) | 0.21 | 0.26 |  | 0.01 (-0.26, 0.28) | 0.93 | 0.97 |  |
| COH | blue | **0.43 (0.18, 0.68)** | **0.00** | **0.02** |  | 0.41 (-0.10, 0.91) | 0.12 | 0.33 |  |
| DE(16:0) | brown | -0.07 (-0.49, 0.35) | 0.74 | 0.76 |  | -0.20 (-0.62, 0.22) | 0.35 | 0.58 |  |
| DE(18:1) | red | **0.28 (0.01, 0.56)** | **0.04** | 0.07 |  | 0.21 (-0.29, 0.71) | 0.40 | 0.62 |  |
| DE(18:2) | red | 0.29 (-0.06, 0.64) | 0.10 | 0.15 |  | 0.14 (-0.25, 0.52) | 0.49 | 0.69 |  |
| DE(20:4) | brown | -0.21 (-0.63, 0.21) | 0.33 | 0.37 |  | -0.16 (-0.43, 0.12) | 0.26 | 0.48 |  |
| DE(20:5) | grey | -0.07 (-0.34, 0.21) | 0.63 | 0.66 |  | -0.05 (-0.31, 0.21) | 0.68 | 0.82 |  |
| DE(22:6) | grey | -0.04 (-0.50, 0.42) | 0.85 | 0.86 |  | -0.27 (-0.61, 0.06) | 0.11 | 0.32 |  |
| FA(14:0) | purple | **0.50 (0.17, 0.83)** | **0.00** | **0.02** |  | 0.04 (-0.48, 0.56) | 0.88 | 0.93 |  |
| FA(15:0) | purple | -0.04 (-0.32, 0.24) | 0.78 | 0.80 |  | -0.42 (-1.50, 0.66) | 0.45 | 0.65 |  |
| FA(16:0) | purple | **0.34 (0.08, 0.61)** | **0.01** | **0.03** |  | 0.41 (-0.02, 0.83) | 0.06 | 0.24 |  |
| FA(16:1) | purple | **0.36 (0.13, 0.59)** | **0.00** | **0.02** |  | 0.19 (-0.04, 0.41) | 0.10 | 0.30 |  |
| FA(16:2) | grey | -0.11 (-0.31, 0.09) | 0.28 | 0.33 |  | -0.84 (-1.82, 0.14) | 0.09 | 0.29 |  |
| FA(17:0) | purple | -0.03 (-0.29, 0.24) | 0.85 | 0.86 |  | -0.42 (-1.40, 0.56) | 0.40 | 0.62 |  |
| FA(17:1) | purple | 0.03 (-0.26, 0.31) | 0.86 | 0.87 |  | -0.06 (-0.77, 0.65) | 0.87 | 0.93 |  |
| FA(18:0) | grey | -0.03 (-0.30, 0.23) | 0.80 | 0.81 |  | -0.77 (-2.05, 0.51) | 0.24 | 0.46 |  |
| **FA(18:1)** | **purple (hub)** | **0.39 (0.09, 0.69)** | **0.01** | **0.03** |  | 0.20 (-0.01, 0.41) | 0.07 | 0.25 |  |
| FA(18:2) | purple | **0.44 (0.14, 0.74)** | **0.00** | **0.02** |  | 0.17 (-0.03, 0.36) | 0.09 | 0.29 |  |
| FA(18:3) | purple | **0.55 (0.22, 0.88)** | **0.00** | **0.02** |  | 0.27 (-0.10, 0.64) | 0.16 | 0.37 |  |
| FA(20:2) | purple | 0.09 (-0.17, 0.35) | 0.51 | 0.55 |  | 0.24 (-0.50, 0.99) | 0.52 | 0.71 |  |
| FA(20:3) | purple | 0.17 (-0.11, 0.45) | 0.23 | 0.28 |  | 0.21 (-0.20, 0.62) | 0.32 | 0.55 |  |
| FA(20:4) | purple | 0.08 (-0.26, 0.43) | 0.64 | 0.67 |  | 0.07 (-0.21, 0.36) | 0.61 | 0.76 |  |
| FA(20:5) | purple | -0.04 (-0.24, 0.16) | 0.69 | 0.71 |  | 0.07 (-0.41, 0.56) | 0.76 | 0.87 |  |
| FA(22:4) | purple | 0.15 (-0.12, 0.43) | 0.27 | 0.32 |  | 0.21 (-0.26, 0.68) | 0.37 | 0.60 |  |
| FA(22:5) | purple | 0.11 (-0.15, 0.37) | 0.41 | 0.46 |  | -0.01 (-0.44, 0.43) | 0.97 | 0.98 |  |
| FA(22:6) | purple | 0.10 (-0.18, 0.38) | 0.49 | 0.52 |  | -0.08 (-0.38, 0.21) | 0.58 | 0.75 |  |
| AC(12:0) | cyan | **0.69 (0.35, 1.03)** | **0.00** | **0.00** |  | **0.36 (0.14, 0.58)** | **0.00** | **0.04** |  |
| AC(13:0) | grey | 0.10 (-0.21, 0.42) | 0.52 | 0.56 |  | 0.03 (-0.18, 0.25) | 0.77 | 0.87 |  |
| **AC(14:0)** | **cyan (hub)** | **0.66 (0.37, 0.96)** | **0.00** | **0.00** |  | **0.49 (0.23, 0.76)** | **0.00** | **0.02** |  |
| AC(14:1) | cyan | **0.65 (0.34, 0.96)** | **0.00** | **0.00** |  | **0.34 (0.14, 0.54)** | **0.00** | **0.04** |  |
| AC(14:2) | cyan | **0.90 (0.47, 1.33)** | **0.00** | **0.00** |  | **0.42 (0.15, 0.69)** | **0.00** | **0.05** |  |
| AC(15:0) (a) | cyan | 0.33 (-0.04, 0.70) | 0.08 | 0.12 |  | **0.36 (0.05, 0.68)** | **0.02** | 0.16 |  |
| AC(15:0) (b) | cyan | **0.68 (0.33, 1.04)** | **0.00** | **0.01** |  | **0.72 (0.35, 1.09)** | **0.00** | **0.02** |  |
| AC(16:0) | cyan | **0.76 (0.43, 1.09)** | **0.00** | **0.00** |  | **0.69 (0.31, 1.07)** | **0.00** | **0.02** |  |
| AC(16:1) | cyan | **0.65 (0.40, 0.90)** | **0.00** | **0.00** |  | **0.45 (0.23, 0.67)** | **0.00** | **0.01** |  |
| AC(17:0) (a) | cyan | **0.66 (0.28, 1.05)** | **0.00** | **0.02** |  | **0.55 (0.05, 1.06)** | **0.03** | 0.18 |  |
| AC(17:0) (b) | cyan | **0.75 (0.42, 1.08)** | **0.00** | **0.00** |  | **0.80 (0.31, 1.29)** | **0.00** | **0.04** |  |
| AC(18:0) | cyan | **0.62 (0.30, 0.94)** | **0.00** | **0.01** |  | **0.46 (0.14, 0.77)** | **0.00** | 0.07 |  |
| AC(18:1) | cyan | **0.80 (0.50, 1.09)** | **0.00** | **0.00** |  | **0.57 (0.32, 0.82)** | **0.00** | **0.01** |  |
| AC(18:2) | cyan | **0.80 (0.46, 1.13)** | **0.00** | **0.00** |  | **0.50 (0.23, 0.77)** | **0.00** | **0.02** |  |
| DG(14:0_16:0) | brown | **0.46 (0.03, 0.90)** | **0.04** | 0.06 |  | **0.41 (0.06, 0.76)** | **0.02** | 0.15 |  |
| DG(14:0_18:2) | brown | **0.40 (0.04, 0.77)** | **0.03** | 0.06 |  | **0.23 (0.02, 0.43)** | **0.03** | 0.18 |  |
| DG(16:0_16:0) | brown | **0.42 (0.05, 0.78)** | **0.02** | 0.05 |  | **0.36 (0.08, 0.65)** | **0.01** | 0.12 |  |
| DG(16:0_16:1) | brown | **0.40 (0.08, 0.72)** | **0.01** | **0.04** |  | **0.35 (0.08, 0.62)** | **0.01** | 0.11 |  |
| DG(16:0_18:1) | brown | **0.46 (0.12, 0.80)** | **0.01** | **0.03** |  | **0.27 (0.06, 0.49)** | **0.01** | 0.12 |  |
| DG(16:0_18:2) | brown | **0.38 (0.06, 0.69)** | **0.02** | **0.04** |  | **0.20 (0.01, 0.38)** | **0.04** | 0.19 |  |
| DG(16:0_20:4) | brown | 0.25 (-0.00, 0.51) | 0.05 | 0.08 |  | 0.17 (-0.02, 0.36) | 0.08 | 0.26 |  |
| DG(16:0_22:5) | brown | 0.13 (-0.08, 0.35) | 0.23 | 0.28 |  | 0.13 (-0.04, 0.30) | 0.14 | 0.35 |  |
| DG(16:0_22:6) | yellow | 0.14 (-0.11, 0.39) | 0.27 | 0.31 |  | 0.00 (-0.16, 0.17) | 0.97 | 0.98 |  |
| DG(16:1_18:1) | brown | **0.43 (0.11, 0.75)** | **0.01** | **0.03** |  | **0.31 (0.06, 0.56)** | **0.02** | 0.14 |  |
| DG(18:0_18:1) | brown | **0.35 (0.04, 0.66)** | **0.03** | 0.05 |  | **0.35 (0.10, 0.60)** | **0.01** | 0.09 |  |
| DG(18:0_18:2) | brown | 0.28 (-0.02, 0.57) | 0.06 | 0.10 |  | **0.26 (0.06, 0.46)** | **0.01** | 0.12 |  |
| DG(18:0_20:4) | magenta | **0.25 (0.05, 0.45)** | **0.02** | **0.04** |  | **0.22 (0.05, 0.39)** | **0.01** | 0.12 |  |
| DG(18:0_22:6) | grey | -0.02 (-0.22, 0.18) | 0.82 | 0.84 |  | 0.29 (-0.31, 0.88) | 0.35 | 0.58 |  |
| DG(18:1_18:1) | brown | **0.39 (0.06, 0.72)** | **0.02** | **0.04** |  | **0.23 (0.02, 0.43)** | **0.03** | 0.18 |  |
| DG(18:1_18:2) | brown | **0.40 (0.04, 0.76)** | **0.03** | 0.06 |  | 0.16 (-0.02, 0.35) | 0.09 | 0.29 |  |
| DG(18:1_18:3) | brown | 0.37 (-0.02, 0.75) | 0.06 | 0.10 |  | 0.17 (-0.06, 0.39) | 0.14 | 0.35 |  |
| DG(18:1_20:3) | brown | **0.39 (0.10, 0.68)** | **0.01** | **0.03** |  | 0.24 (-0.05, 0.53) | 0.10 | 0.31 |  |
| DG(18:1_20:4) | brown | **0.26 (0.01, 0.52)** | **0.04** | 0.07 |  | 0.17 (-0.09, 0.42) | 0.20 | 0.43 |  |
| DG(18:1_20:5) | yellow | 0.12 (-0.14, 0.38) | 0.37 | 0.41 |  | 0.09 (-0.10, 0.29) | 0.36 | 0.59 |  |
| DG(18:1_22:5) | brown | **0.30 (0.05, 0.55)** | **0.02** | **0.04** |  | 0.11 (-0.17, 0.38) | 0.45 | 0.65 |  |
| DG(18:1_22:6) | yellow | 0.11 (-0.14, 0.37) | 0.37 | 0.42 |  | -0.05 (-0.24, 0.14) | 0.63 | 0.78 |  |
| DG(18:2_18:2) | brown | 0.23 (-0.08, 0.55) | 0.14 | 0.18 |  | 0.08 (-0.08, 0.24) | 0.30 | 0.53 |  |
| DG(18:2_20:4) | yellow | 0.19 (-0.05, 0.43) | 0.12 | 0.16 |  | 0.09 (-0.12, 0.29) | 0.42 | 0.64 |  |
| DG(18:2_22:6) | yellow | 0.10 (-0.15, 0.35) | 0.42 | 0.46 |  | -0.04 (-0.20, 0.12) | 0.61 | 0.76 |  |
| TG(48:0) [SIM] | brown | **0.41 (0.10, 0.72)** | **0.01** | **0.03** |  | 0.16 (-0.11, 0.43) | 0.24 | 0.46 |  |
| TG(48:1) [SIM] | brown | **0.37 (0.07, 0.66)** | **0.01** | **0.04** |  | 0.26 (-0.04, 0.56) | 0.09 | 0.29 |  |
| TG(48:2) [SIM] | brown | **0.51 (0.20, 0.82)** | **0.00** | **0.02** |  | **0.30 (0.04, 0.56)** | **0.02** | 0.16 |  |
| TG(48:3) [SIM] | brown | **0.72 (0.26, 1.19)** | **0.00** | **0.02** |  | **0.36 (0.06, 0.67)** | **0.02** | 0.15 |  |
| TG(49:1) [SIM] | brown | **0.42 (0.05, 0.78)** | **0.02** | 0.05 |  | 0.31 (-0.01, 0.62) | 0.05 | 0.23 |  |
| TG(50:0) [SIM] | brown | **0.39 (0.09, 0.69)** | **0.01** | **0.03** |  | 0.20 (-0.04, 0.44) | 0.11 | 0.31 |  |
| TG(50:1) [SIM] | brown | **0.30 (0.06, 0.54)** | **0.01** | **0.04** |  | 0.29 (-0.03, 0.60) | 0.07 | 0.26 |  |
| TG(50:2) [SIM] | brown | **0.25 (0.04, 0.45)** | **0.02** | **0.04** |  | 0.31 (-0.02, 0.64) | 0.06 | 0.25 |  |
| **TG(50:3) [SIM]** | **brown (hub)** | **0.32 (0.09, 0.56)** | **0.01** | **0.03** |  | 0.23 (-0.02, 0.48) | 0.07 | 0.26 |  |
| TG(50:4) [SIM] | brown | **0.39 (0.07, 0.70)** | **0.02** | **0.04** |  | 0.20 (-0.03, 0.43) | 0.09 | 0.29 |  |
| TG(51:0) [SIM] | grey | -0.12 (-0.36, 0.11) | 0.31 | 0.36 |  | 0.11 (-1.09, 1.31) | 0.86 | 0.92 |  |
| TG(51:1) [SIM] | brown | **0.43 (0.10, 0.77)** | **0.01** | **0.03** |  | 0.26 (-0.01, 0.53) | 0.06 | 0.25 |  |
| TG(51:2) [SIM] | brown | **0.44 (0.12, 0.76)** | **0.01** | **0.03** |  | **0.25 (0.00, 0.49)** | **0.05** | 0.23 |  |
| TG(52:1) [SIM] | brown | **0.41 (0.13, 0.68)** | **0.00** | **0.02** |  | **0.24 (0.01, 0.47)** | **0.04** | 0.21 |  |
| TG(52:2) [SIM] | brown | **0.24 (0.01, 0.47)** | **0.04** | 0.07 |  | **0.38 (0.00, 0.76)** | **0.05** | 0.23 |  |
| TG(52:3) [SIM] | brown | **0.26 (0.03, 0.48)** | **0.02** | **0.05** |  | 0.28 (-0.03, 0.60) | 0.07 | 0.26 |  |
| TG(52:4) [SIM] | brown | **0.30 (0.05, 0.55)** | **0.02** | **0.04** |  | 0.23 (-0.01, 0.47) | 0.06 | 0.25 |  |
| TG(52:5) [SIM] | brown | **0.32 (0.05, 0.59)** | **0.02** | **0.04** |  | 0.18 (-0.08, 0.43) | 0.17 | 0.39 |  |
| TG(53:2) [SIM] | brown | **0.38 (0.05, 0.72)** | **0.02** | **0.05** |  | **0.28 (0.01, 0.56)** | **0.04** | 0.21 |  |
| TG(54:0) [SIM] | brown | **0.54 (0.08, 0.99)** | **0.02** | **0.04** |  | 0.40 (-0.02, 0.82) | 0.06 | 0.25 |  |
| TG(54:1) [SIM] | brown | **0.49 (0.12, 0.85)** | **0.01** | **0.03** |  | **0.26 (0.01, 0.51)** | **0.04** | 0.21 |  |
| TG(54:2) [SIM] | brown | **0.42 (0.11, 0.73)** | **0.01** | **0.03** |  | **0.29 (0.07, 0.51)** | **0.01** | 0.11 |  |
| TG(54:3) [SIM] | brown | **0.32 (0.00, 0.64)** | **0.05** | 0.08 |  | **0.38 (0.03, 0.72)** | **0.03** | 0.18 |  |
| TG(54:4) [SIM] | brown | **0.33 (0.05, 0.60)** | **0.02** | **0.04** |  | 0.27 (-0.03, 0.58) | 0.08 | 0.27 |  |
| TG(54:5) [SIM] | yellow | **0.26 (0.01, 0.51)** | **0.04** | 0.08 |  | 0.18 (-0.12, 0.48) | 0.24 | 0.46 |  |
| TG(54:6) [SIM] | yellow | 0.18 (-0.05, 0.41) | 0.12 | 0.17 |  | 0.04 (-0.22, 0.30) | 0.77 | 0.87 |  |
| TG(54:7) [SIM] | yellow | 0.17 (-0.05, 0.39) | 0.12 | 0.16 |  | 0.02 (-0.20, 0.24) | 0.85 | 0.92 |  |
| TG(56:6) [SIM] | yellow | 0.13 (-0.03, 0.29) | 0.12 | 0.16 |  | 0.02 (-0.27, 0.30) | 0.91 | 0.95 |  |
| TG(56:7) [SIM] | yellow | 0.11 (-0.07, 0.29) | 0.24 | 0.29 |  | -0.06 (-0.30, 0.17) | 0.59 | 0.76 |  |
| **TG(56:8) [SIM]** | **yellow (hub)** | 0.11 (-0.10, 0.33) | 0.28 | 0.33 |  | -0.02 (-0.23, 0.19) | 0.84 | 0.91 |  |
| TG(56:9) [SIM] | yellow | 0.10 (-0.12, 0.32) | 0.37 | 0.42 |  | -0.02 (-0.30, 0.27) | 0.92 | 0.96 |  |
| TG(58:10) [SIM] | yellow | 0.10 (-0.14, 0.33) | 0.43 | 0.47 |  | -0.07 (-0.31, 0.18) | 0.59 | 0.76 |  |
| TG(58:8) [SIM] | yellow | 0.10 (-0.13, 0.34) | 0.38 | 0.42 |  | -0.08 (-0.37, 0.20) | 0.56 | 0.74 |  |
| TG(58:9) [SIM] | yellow | 0.12 (-0.11, 0.35) | 0.32 | 0.36 |  | -0.06 (-0.30, 0.18) | 0.61 | 0.76 |  |
| TG(48:0) [NL-16:0] | brown | 0.27 (-0.03, 0.57) | 0.08 | 0.11 |  | 0.15 (-0.09, 0.39) | 0.21 | 0.44 |  |
| TG(48:0) [NL-18:0] | brown | 0.30 (-0.09, 0.70) | 0.13 | 0.17 |  | 0.18 (-0.10, 0.45) | 0.20 | 0.43 |  |
| TG(48:1) [NL-16:1] | brown | **0.40 (0.11, 0.68)** | **0.01** | **0.03** |  | 0.17 (-0.07, 0.41) | 0.17 | 0.38 |  |
| TG(48:1) [NL-18:1] | brown | **0.49 (0.19, 0.80)** | **0.00** | **0.02** |  | **0.26 (0.04, 0.47)** | **0.02** | 0.15 |  |
| TG(48:2) [NL-14:0] | brown | **0.39 (0.11, 0.68)** | **0.01** | **0.03** |  | **0.21 (0.01, 0.40)** | **0.04** | 0.20 |  |
| TG(48:2) [NL-14:1] | brown | **0.44 (0.13, 0.75)** | **0.01** | **0.03** |  | **0.20 (0.03, 0.37)** | **0.02** | 0.16 |  |
| TG(48:2) [NL-16:1] | brown | **0.49 (0.20, 0.78)** | **0.00** | **0.02** |  | **0.31 (0.06, 0.57)** | **0.02** | 0.14 |  |
| TG(48:2) [NL-18:2] | brown | **0.42 (0.09, 0.75)** | **0.01** | **0.03** |  | 0.18 (-0.02, 0.38) | 0.08 | 0.28 |  |
| TG(48:3) [NL-14:0] | brown | **0.42 (0.09, 0.75)** | **0.01** | **0.03** |  | 0.16 (-0.01, 0.33) | 0.06 | 0.24 |  |
| TG(48:3) [NL-16:1] | brown | **0.49 (0.19, 0.79)** | **0.00** | **0.02** |  | **0.29 (0.04, 0.53)** | **0.02** | 0.16 |  |
| TG(48:3) [NL-18:3] | brown | **0.49 (0.12, 0.87)** | **0.01** | **0.03** |  | 0.17 (-0.03, 0.37) | 0.09 | 0.29 |  |
| TG(49:1) [NL-16:1] | brown | 0.20 (-0.05, 0.46) | 0.12 | 0.16 |  | 0.33 (-0.01, 0.68) | 0.06 | 0.24 |  |
| TG(49:1) [NL-17:1] | brown | **0.43 (0.11, 0.75)** | **0.01** | **0.03** |  | **0.28 (0.01, 0.56)** | **0.04** | 0.21 |  |
| TG(50:0) [NL-18:0] | brown | **0.49 (0.20, 0.79)** | **0.00** | **0.02** |  | 0.17 (-0.01, 0.35) | 0.06 | 0.24 |  |
| TG(50:1) [NL-14:0] | brown | 0.22 (-0.11, 0.54) | 0.19 | 0.23 |  | 0.18 (-0.04, 0.40) | 0.11 | 0.33 |  |
| TG(50:1) [NL-16:0] | brown | **0.32 (0.08, 0.56)** | **0.01** | **0.03** |  | 0.22 (-0.04, 0.48) | 0.10 | 0.30 |  |
| TG(50:1) [NL-18:1] | brown | **0.31 (0.06, 0.56)** | **0.01** | **0.04** |  | 0.25 (-0.05, 0.54) | 0.10 | 0.30 |  |
| TG(50:2) [NL-14:0] | brown | **0.31 (0.02, 0.60)** | **0.04** | 0.07 |  | 0.19 (-0.01, 0.39) | 0.06 | 0.24 |  |
| TG(50:2) [NL-16:1] | brown | **0.33 (0.10, 0.57)** | **0.00** | **0.02** |  | **0.25 (0.00, 0.49)** | **0.05** | 0.23 |  |
| TG(50:2) [NL-18:1] | brown | **0.27 (0.06, 0.47)** | **0.01** | **0.03** |  | **0.33 (0.04, 0.61)** | **0.03** | 0.16 |  |
| TG(50:2) [NL-18:2] | brown | 0.20 (-0.02, 0.42) | 0.07 | 0.10 |  | 0.17 (-0.09, 0.43) | 0.20 | 0.42 |  |
| TG(50:3) [NL-14:0] | brown | **0.39 (0.09, 0.68)** | **0.01** | **0.03** |  | **0.20 (0.03, 0.36)** | **0.02** | 0.16 |  |
| TG(50:3) [NL-14:1] | brown | **0.41 (0.09, 0.73)** | **0.01** | **0.03** |  | **0.23 (0.06, 0.41)** | **0.01** | 0.11 |  |
| TG(50:3) [NL-16:1] | brown | **0.33 (0.09, 0.57)** | **0.01** | **0.03** |  | 0.23 (-0.01, 0.46) | 0.06 | 0.24 |  |
| TG(50:3) [NL-18:2] | brown | **0.35 (0.11, 0.60)** | **0.00** | **0.02** |  | 0.22 (-0.01, 0.44) | 0.06 | 0.24 |  |
| TG(50:3) [NL-18:3] | brown | **0.32 (0.05, 0.59)** | **0.02** | **0.04** |  | 0.16 (-0.07, 0.38) | 0.18 | 0.40 |  |
| TG(50:4) [NL-14:0] | brown | **0.39 (0.04, 0.73)** | **0.03** | 0.05 |  | 0.12 (-0.04, 0.28) | 0.13 | 0.34 |  |
| TG(50:4) [NL-18:3] | brown | **0.37 (0.08, 0.67)** | **0.01** | **0.04** |  | 0.17 (-0.04, 0.38) | 0.11 | 0.32 |  |
| TG(50:4) [NL-20:4] | yellow | 0.18 (-0.05, 0.42) | 0.12 | 0.16 |  | 0.08 (-0.11, 0.28) | 0.40 | 0.62 |  |
| TG(51:0) [NL-16:0] | brown | **0.67 (0.27, 1.06)** | **0.00** | **0.02** |  | **0.25 (0.00, 0.50)** | **0.05** | 0.23 |  |
| TG(51:1) [NL-17:0] | brown | **0.54 (0.22, 0.86)** | **0.00** | **0.02** |  | **0.30 (0.08, 0.52)** | **0.01** | 0.09 |  |
| TG(51:2) [NL-15:0] | brown | **0.45 (0.12, 0.79)** | **0.01** | **0.03** |  | **0.24 (0.06, 0.43)** | **0.01** | 0.11 |  |
| TG(51:2) [NL-17:0] | brown | **0.40 (0.10, 0.69)** | **0.01** | **0.03** |  | **0.23 (0.01, 0.46)** | **0.04** | 0.21 |  |
| TG(51:2) [NL-17:1] | brown | **0.40 (0.10, 0.69)** | **0.01** | **0.03** |  | **0.23 (0.01, 0.45)** | **0.04** | 0.21 |  |
| TG(52:1) [NL-18:0] | brown | **0.39 (0.11, 0.67)** | **0.01** | **0.03** |  | 0.19 (-0.00, 0.39) | 0.05 | 0.23 |  |
| TG(52:1) [NL-18:1] | brown | **0.43 (0.14, 0.71)** | **0.00** | **0.02** |  | **0.20 (0.02, 0.38)** | **0.03** | 0.17 |  |
| TG(52:2) [NL-16:0] | brown | **0.26 (0.03, 0.49)** | **0.03** | 0.06 |  | 0.15 (-0.11, 0.40) | 0.27 | 0.49 |  |
| TG(52:2) [NL-18:2] | brown | 0.18 (-0.04, 0.41) | 0.11 | 0.16 |  | 0.11 (-0.12, 0.33) | 0.35 | 0.58 |  |
| TG(52:3) [NL-16:1] | brown | 0.14 (-0.05, 0.33) | 0.15 | 0.19 |  | 0.17 (-0.07, 0.41) | 0.16 | 0.37 |  |
| TG(52:3) [NL-18:2] | brown | 0.19 (-0.05, 0.43) | 0.11 | 0.16 |  | 0.13 (-0.09, 0.36) | 0.25 | 0.47 |  |
| TG(52:4) [NL-16:1] | brown | **0.31 (0.08, 0.53)** | **0.01** | **0.03** |  | 0.16 (-0.02, 0.35) | 0.07 | 0.26 |  |
| TG(52:4) [NL-18:2] | brown | 0.22 (-0.06, 0.50) | 0.12 | 0.16 |  | 0.11 (-0.09, 0.31) | 0.29 | 0.51 |  |
| TG(52:4) [NL-18:3] | brown | **0.37 (0.09, 0.65)** | **0.01** | **0.03** |  | 0.15 (-0.05, 0.35) | 0.14 | 0.35 |  |
| TG(52:5) [NL-18:3] | brown | **0.39 (0.06, 0.72)** | **0.02** | **0.04** |  | 0.12 (-0.06, 0.30) | 0.18 | 0.40 |  |
| TG(52:5) [NL-20:4] | yellow | **0.23 (0.00, 0.47)** | **0.05** | 0.08 |  | 0.06 (-0.16, 0.29) | 0.58 | 0.75 |  |
| TG(52:5) [NL-20:5] | yellow | 0.09 (-0.11, 0.29) | 0.39 | 0.43 |  | 0.00 (-0.16, 0.17) | 0.98 | 0.99 |  |
| TG(53:2) [NL-17:1] | brown | **0.36 (0.06, 0.66)** | **0.02** | **0.04** |  | **0.26 (0.00, 0.51)** | **0.05** | 0.23 |  |
| TG(53:2) [NL-18:1] | brown | **0.48 (0.15, 0.81)** | **0.00** | **0.02** |  | **0.27 (0.04, 0.49)** | **0.02** | 0.15 |  |
| TG(54:0) [NL-18:0] | brown | **0.50 (0.14, 0.86)** | **0.01** | **0.03** |  | 0.23 (-0.02, 0.49) | 0.07 | 0.25 |  |
| TG(54:1) [NL-18:1] | brown | **0.43 (0.09, 0.77)** | **0.01** | **0.03** |  | 0.17 (-0.03, 0.37) | 0.09 | 0.29 |  |
| TG(54:2) [NL-18:0] | brown | **0.40 (0.10, 0.71)** | **0.01** | **0.03** |  | **0.22 (0.03, 0.40)** | **0.02** | 0.16 |  |
| TG(54:2) [NL-20:1] | brown | **0.50 (0.17, 0.82)** | **0.00** | **0.02** |  | **0.25 (0.07, 0.42)** | **0.01** | 0.09 |  |
| TG(54:3) [NL-18:1] | brown | 0.23 (-0.10, 0.57) | 0.17 | 0.21 |  | **0.32 (0.02, 0.61)** | **0.04** | 0.19 |  |
| TG(54:3) [NL-18:2] | brown | 0.28 (-0.02, 0.58) | 0.06 | 0.10 |  | 0.18 (-0.04, 0.40) | 0.11 | 0.32 |  |
| TG(54:4) [NL-18:2] | brown | **0.36 (0.03, 0.70)** | **0.04** | 0.06 |  | 0.17 (-0.05, 0.39) | 0.12 | 0.34 |  |
| TG(54:4) [NL-20:3] | brown | **0.23 (0.05, 0.40)** | **0.01** | **0.03** |  | 0.14 (-0.08, 0.37) | 0.21 | 0.43 |  |
| TG(54:5) [NL-18:3] | brown | **0.43 (0.05, 0.81)** | **0.03** | 0.05 |  | 0.13 (-0.07, 0.33) | 0.20 | 0.42 |  |
| TG(54:5) [NL-20:4] | yellow | 0.12 (-0.04, 0.28) | 0.15 | 0.19 |  | 0.04 (-0.19, 0.27) | 0.76 | 0.87 |  |
| TG(54:6) [NL-18:3] | brown | **0.43 (0.02, 0.84)** | **0.04** | 0.07 |  | 0.11 (-0.07, 0.30) | 0.24 | 0.46 |  |
| TG(54:6) [NL-20:4] | yellow | 0.13 (-0.06, 0.32) | 0.18 | 0.23 |  | 0.01 (-0.22, 0.25) | 0.91 | 0.95 |  |
| TG(54:6) [NL-20:5] | yellow | 0.06 (-0.12, 0.24) | 0.52 | 0.55 |  | 0.02 (-0.15, 0.20) | 0.79 | 0.88 |  |
| TG(54:6) [NL-22:6] | yellow | 0.07 (-0.21, 0.34) | 0.64 | 0.67 |  | -0.11 (-0.29, 0.07) | 0.24 | 0.46 |  |
| TG(54:7) [NL-20:5] | yellow | 0.10 (-0.11, 0.30) | 0.36 | 0.41 |  | 0.04 (-0.12, 0.21) | 0.60 | 0.76 |  |
| TG(54:7) [NL-22:6] | yellow | 0.12 (-0.15, 0.40) | 0.38 | 0.42 |  | -0.05 (-0.21, 0.11) | 0.52 | 0.71 |  |
| TG(56:6) [NL-20:4] | yellow | 0.10 (-0.07, 0.27) | 0.25 | 0.30 |  | -0.01 (-0.25, 0.23) | 0.93 | 0.97 |  |
| TG(56:6) [NL-22:5](a) | yellow | 0.15 (-0.03, 0.34) | 0.10 | 0.14 |  | -0.01 (-0.25, 0.24) | 0.97 | 0.98 |  |
| TG(56:6) [NL-22:5](b) | yellow | 0.16 (-0.03, 0.34) | 0.09 | 0.14 |  | -0.01 (-0.25, 0.24) | 0.96 | 0.98 |  |
| TG(56:7) [NL-20:4] | yellow | 0.12 (-0.04, 0.29) | 0.15 | 0.19 |  | 0.06 (-0.15, 0.26) | 0.58 | 0.75 |  |
| TG(56:7) [NL-20:5] | yellow | 0.08 (-0.12, 0.27) | 0.43 | 0.47 |  | 0.05 (-0.14, 0.23) | 0.64 | 0.78 |  |
| TG(56:7) [NL-22:5](a) | yellow | 0.16 (-0.05, 0.36) | 0.13 | 0.17 |  | 0.00 (-0.24, 0.24) | 1.00 | 1.00 |  |
| TG(56:7) [NL-22:5](b) | yellow | 0.16 (-0.05, 0.36) | 0.13 | 0.17 |  | 0.00 (-0.24, 0.24) | 1.00 | 1.00 |  |
| TG(56:7) [NL-22:6] | yellow | 0.10 (-0.13, 0.33) | 0.38 | 0.42 |  | -0.06 (-0.25, 0.12) | 0.51 | 0.70 |  |
| TG(56:8) [NL-20:4] | yellow | 0.15 (-0.05, 0.36) | 0.13 | 0.17 |  | 0.01 (-0.18, 0.21) | 0.88 | 0.93 |  |
| TG(56:8) [NL-20:5] | yellow | 0.11 (-0.10, 0.33) | 0.30 | 0.35 |  | 0.04 (-0.12, 0.20) | 0.63 | 0.78 |  |
| TG(56:8) [NL-22:6] | yellow | 0.07 (-0.17, 0.31) | 0.58 | 0.61 |  | -0.06 (-0.22, 0.11) | 0.49 | 0.69 |  |
| TG(56:9) [NL-22:6] | yellow | 0.12 (-0.13, 0.38) | 0.34 | 0.39 |  | -0.03 (-0.18, 0.12) | 0.70 | 0.83 |  |
| TG(58:10) [NL-22:6] | yellow | 0.11 (-0.14, 0.36) | 0.38 | 0.42 |  | -0.05 (-0.21, 0.11) | 0.56 | 0.74 |  |
| TG(58:8) [NL-22:6] | yellow | 0.11 (-0.12, 0.34) | 0.33 | 0.38 |  | -0.08 (-0.26, 0.11) | 0.41 | 0.63 |  |
| TG(58:9) [NL-22:6] | yellow | 0.08 (-0.14, 0.31) | 0.47 | 0.50 |  | -0.02 (-0.17, 0.14) | 0.83 | 0.90 |  |
| TG(O-50:1) [SIM] | black | 0.34 (-0.04, 0.73) | 0.08 | 0.11 |  | 0.17 (-0.30, 0.65) | 0.47 | 0.68 |  |
| TG(O-50:2) [SIM] | black | -0.01 (-0.22, 0.19) | 0.89 | 0.90 |  | -0.07 (-0.50, 0.35) | 0.74 | 0.85 |  |
| TG(O-50:3) [SIM] | black | -0.07 (-0.28, 0.13) | 0.48 | 0.51 |  | 0.01 (-0.63, 0.65) | 0.98 | 0.99 |  |
| TG(O-52:0) [SIM] | grey | 0.10 (-0.16, 0.35) | 0.46 | 0.50 |  | 0.06 (-0.15, 0.27) | 0.56 | 0.74 |  |
| TG(O-52:1) [SIM] | black | 0.47 (-0.03, 0.96) | 0.06 | 0.10 |  | 0.22 (-0.24, 0.67) | 0.35 | 0.58 |  |
| TG(O-52:2) [SIM] | black | 0.41 (-0.17, 1.00) | 0.17 | 0.21 |  | 0.29 (-0.27, 0.86) | 0.31 | 0.53 |  |
| TG(O-54:2) [SIM] | black | 0.39 (-0.18, 0.97) | 0.18 | 0.22 |  | 0.18 (-0.36, 0.71) | 0.51 | 0.70 |  |
| TG(O-54:3) [SIM] | grey | -0.23 (-0.54, 0.09) | 0.16 | 0.20 |  | -0.23 (-0.90, 0.43) | 0.49 | 0.69 |  |
| TG(O-54:4) [SIM] | brown | 0.22 (-0.06, 0.50) | 0.12 | 0.16 |  | 0.25 (-0.15, 0.65) | 0.22 | 0.45 |  |
| TG(O-50:1) [NL-15:0] | black | **0.41 (0.05, 0.76)** | **0.02** | **0.05** |  | 0.13 (-0.08, 0.34) | 0.24 | 0.46 |  |
| TG(O-50:1) [NL-16:0] | black | **0.63 (0.20, 1.06)** | **0.00** | **0.02** |  | 0.18 (-0.10, 0.46) | 0.21 | 0.44 |  |
| TG(O-50:1) [NL-17:1] | black | **0.40 (0.07, 0.73)** | **0.02** | **0.04** |  | 0.20 (-0.18, 0.59) | 0.31 | 0.53 |  |
| TG(O-50:1) [NL-18:1] | black | **0.64 (0.14, 1.14)** | **0.01** | **0.03** |  | 0.17 (-0.11, 0.44) | 0.23 | 0.46 |  |
| TG(O-50:2) [NL-16:1] | black | 0.11 (-0.08, 0.29) | 0.26 | 0.31 |  | 0.00 (-0.26, 0.25) | 0.97 | 0.98 |  |
| TG(O-50:2) [NL-18:1] | black | 0.37 (-0.05, 0.79) | 0.08 | 0.12 |  | 0.04 (-0.29, 0.37) | 0.82 | 0.90 |  |
| TG(O-50:2) [NL-18:2] | black | **0.52 (0.15, 0.89)** | **0.01** | **0.03** |  | 0.17 (-0.05, 0.39) | 0.13 | 0.34 |  |
| TG(O-50:3) [NL-18:2] | grey | 0.00 (-0.21, 0.22) | 0.97 | 0.97 |  | 0.02 (-0.10, 0.13) | 0.79 | 0.88 |  |
| TG(O-52:0) [NL-16:0] | black | **0.50 (0.20, 0.80)** | **0.00** | **0.02** |  | 0.25 (-0.07, 0.57) | 0.13 | 0.34 |  |
| TG(O-52:1) [NL-16:0] | black | **0.63 (0.22, 1.05)** | **0.00** | **0.02** |  | 0.16 (-0.08, 0.40) | 0.19 | 0.40 |  |
| TG(O-52:1) [NL-18:1] | black | **0.69 (0.20, 1.18)** | **0.01** | **0.03** |  | 0.14 (-0.09, 0.38) | 0.23 | 0.46 |  |
| TG(O-52:2) [NL-16:0] | black | **0.84 (0.30, 1.38)** | **0.00** | **0.02** |  | **0.24 (0.01, 0.46)** | **0.04** | 0.21 |  |
| TG(O-52:2) [NL-17:1] | black | **0.85 (0.27, 1.42)** | **0.00** | **0.02** |  | 0.21 (-0.12, 0.55) | 0.21 | 0.44 |  |
| TG(O-52:2) [NL-18:1] | black | **0.71 (0.09, 1.34)** | **0.03** | 0.05 |  | 0.21 (-0.09, 0.51) | 0.17 | 0.38 |  |
| TG(O-54:2) [NL-17:1] | black | **0.56 (0.13, 1.00)** | **0.01** | **0.03** |  | 0.07 (-0.13, 0.27) | 0.49 | 0.68 |  |
| TG(O-54:2) [NL-18:1] | black | **0.74 (0.13, 1.35)** | **0.02** | **0.04** |  | 0.16 (-0.10, 0.42) | 0.24 | 0.46 |  |
| TG(O-54:3) [NL-17:1] | grey | 0.09 (-0.16, 0.35) | 0.48 | 0.52 |  | 0.00 (-0.07, 0.08) | 0.91 | 0.95 |  |
| TG(O-54:3) [NL-18:1] | black | 0.64 (-0.02, 1.31) | 0.06 | 0.09 |  | 0.18 (-0.10, 0.46) | 0.21 | 0.43 |  |
| TG(O-54:4) [NL-17:1] | turquoise | **0.43 (0.17, 0.69)** | **0.00** | **0.02** |  | **0.16 (0.06, 0.26)** | **0.00** | **0.04** |  |
| TG(O-54:4) [NL-18:2] | black | 0.31 (-0.05, 0.67) | 0.09 | 0.14 |  | 0.06 (-0.10, 0.21) | 0.46 | 0.67 |  |
| Ubiquinone | turquoise | **0.63 (0.25, 1.02)** | **0.00** | **0.02** |  | **0.34 (0.03, 0.65)** | **0.03** | 0.18 |  |
| CE(18:2) [+OH] | grey | 0.11 (-0.11, 0.33) | 0.32 | 0.36 |  | -0.09 (-0.22, 0.03) | 0.15 | 0.36 |  |
| CE(20:4) [+OH] | red | **0.35 (0.10, 0.61)** | **0.01** | **0.03** |  | 0.01 (-0.32, 0.33) | 0.98 | 0.99 |  |
| CE(22:6) [+OH] | blue | 0.32 (-0.01, 0.65) | 0.06 | 0.09 |  | -0.05 (-0.45, 0.35) | 0.81 | 0.89 |  |
| LPC(18:2) [+OH] | grey | **0.42 (0.10, 0.74)** | **0.01** | **0.03** |  | 0.06 (-0.18, 0.30) | 0.63 | 0.78 |  |
| LPC(20:4) [+OH] | grey | **0.46 (0.06, 0.86)** | **0.03** | 0.05 |  | 0.04 (-0.08, 0.16) | 0.48 | 0.68 |  |
| LPC(22:6) [+OH] | grey | **0.36 (0.05, 0.67)** | **0.02** | 0.05 |  | 0.06 (-0.08, 0.20) | 0.38 | 0.61 |  |
| PC(34:2) [+OH] | grey | **0.23 (0.02, 0.45)** | **0.03** | 0.06 |  | -0.17 (-0.40, 0.06) | 0.14 | 0.35 |  |
| PC(36:4) [+OH] | blue | **0.41 (0.16, 0.67)** | **0.00** | **0.02** |  | 0.08 (-0.30, 0.47) | 0.67 | 0.81 |  |
| PC(38:6) [+OH] | blue | **0.38 (0.06, 0.69)** | **0.02** | **0.04** |  | 0.13 (-0.15, 0.42) | 0.36 | 0.59 |  |
| **Note:** AMD, adjusted mean difference in lipid concentration (log2 transformed with 95% CI) per unit increase in CBCL raw score; CBCL-ADHP, Child Behavior Checklist attention-deficit/hyperactivity problems subscale; CBCL-ASP, Child Behavior Checklist autism spectrum problems subscale.  Model adjusted for child sex, gestational age (weeks) at birth, minutes from cord blood sample collection to storage, days cord blood sample stored, maternal blood contamination and child's age at CBCL. | | | | | | | | | |

***Supplementary Table S6.2:*** **The association between total cord blood lipid class concentrations and subsequent CBCL-ADHP and CBCL-ASP outcomes at 2 years.**

|  | **CBCL-ADHD** | | | |  | **CBCL-ASD** | | | |
| --- | --- | --- | --- | --- | --- | --- | --- | --- | --- |
| **Cord blood lipid species** | **AMD  (95% CI)** | ***P-*value** | ***Q-*value** | ***R^2^*** |  | ***AMD  (95% CI)*** | ***P-*value** | ***Q-*value** | ***R^2^*** |
| **Class** |  |  |  |  |  |  |  |  |  |
| **Sph** | 0.21 (-0.02, 0.43) | 0.07 | 0.09 | 0.04 |  | 0.17 (-0.05, 0.38) | 0.13 | 0.30 | 0.01 |
| **S1P** | -0.08 (-0.32, 0.17) | 0.54 | 0.57 | 0.04 |  | -0.17 (-0.49, 0.14) | 0.28 | 0.40 | 0 |
| **dhCer** | 0.11 (-0.10, 0.31) | 0.30 | 0.33 | 0.04 |  | 0.29 (-0.07, 0.65) | 0.12 | 0.29 | 0.01 |
| **Cer** | **0.76 (0.34, 1.17)** | **0.00** | **0.01** | 0.06 |  | **0.39 (0.06, 0.73)** | **0.02** | 0.11 | 0.01 |
| **C1P** | **0.50 (0.11, 0.89)** | **0.01** | **0.03** | 0.04 |  | **0.32 (0.05, 0.60)** | **0.02** | 0.11 | 0.01 |
| **MHC** | 0.16 (-0.06, 0.38) | 0.15 | 0.17 | 0.04 |  | -0.04 (-0.36, 0.29) | 0.83 | 0.85 | 0 |
| **DHC** | 0.23 (-0.07, 0.52) | 0.13 | 0.15 | 0.04 |  | 0.10 (-0.26, 0.47) | 0.58 | 0.68 | 0 |
| **THC** | **0.33 (0.11, 0.54)** | **0.00** | **0.02** | 0.04 |  | 0.16 (-0.33, 0.66) | 0.52 | 0.63 | 0 |
| **GM3** | **0.25 (0.03, 0.48)** | **0.03** | **0.05** | 0.04 |  | 0.03 (-0.47, 0.52) | 0.92 | 0.92 | 0 |
| **GM1** | **0.38 (0.09, 0.66)** | **0.01** | **0.03** | 0.04 |  | 0.40 (-0.16, 0.95) | 0.16 | 0.32 | 0.01 |
| **Sulfatide** | 0.38 (0.01, 0.76) | **0.05** | 0.07 | 0.04 |  | 0.14 (-0.24, 0.52) | 0.48 | 0.63 | 0 |
| **SM** | **0.46 (0.20, 0.73)** | **0.00** | **0.01** | 0.05 |  | 0.11 (-0.30, 0.53) | 0.59 | 0.68 | 0 |
| **PA** | **0.28 (0.06, 0.50)** | **0.01** | **0.03** | 0.04 |  | **0.25 (0.07, 0.42)** | **0.01** | 0.05 | 0.01 |
| **PC** | **0.50 (0.19, 0.81)** | **0.00** | **0.02** | 0.05 |  | 0.35 (-0.24, 0.94) | 0.24 | 0.39 | 0 |
| **PC(O)** | **0.32 (0.06, 0.59)** | **0.02** | **0.03** | 0.04 |  | 0.12 (-0.24, 0.48) | 0.51 | 0.63 | 0 |
| **PC(P)** | **0.33 (0.07, 0.59)** | **0.01** | **0.03** | 0.04 |  | 0.11 (-0.23, 0.45) | 0.51 | 0.63 | 0 |
| **LPC** | **0.31 (0.06, 0.56)** | **0.02** | **0.03** | 0.04 |  | 0.30 (-0.10, 0.70) | 0.14 | 0.30 | 0.01 |
| **LPC(O)** | 0.29 (0.02, 0.57) | **0.03** | 0.06 | 0.04 |  | 0.31 (-0.14, 0.77) | 0.17 | 0.32 | 0 |
| **LPC(P)** | 0.26 (-0.06, 0.58) | 0.12 | 0.14 | 0.04 |  | 0.16 (-0.30, 0.61) | 0.50 | 0.63 | 0 |
| **PE** | **0.66 (0.24, 1.08)** | **0.00** | **0.02** | 0.05 |  | **0.45 (0.17, 0.73)** | **0.00** | **0.03** | 0.02 |
| **PE(O)** | **0.50 (0.17, 0.83)** | **0.00** | **0.02** | 0.04 |  | **0.27 (0.02, 0.53)** | **0.04** | 0.16 | 0.01 |
| **PE(P)** | **0.53 (0.16, 0.90)** | **0.01** | **0.02** | 0.04 |  | 0.28 (-0.03, 0.59) | 0.07 | 0.24 | 0.01 |
| **LPE** | 0.23 (-0.04, 0.49) | 0.09 | 0.11 | 0.04 |  | 0.40 (-0.03, 0.82) | 0.07 | 0.24 | 0.01 |
| **LPE(P)** | 0.26 (-0.00, 0.51) | 0.05 | 0.07 | 0.04 |  | 0.20 (-0.09, 0.50) | 0.18 | 0.32 | 0 |
| **PI** | **0.66 (0.22, 1.09)** | **0.00** | **0.02** | 0.05 |  | **0.70 (0.23, 1.17)** | **0.00** | **0.05** | 0.02 |
| **PIP1** | -0.04 (-0.26, 0.18) | 0.73 | 0.73 | 0.04 |  | **0.43 (0.07, 0.79)** | **0.02** | 0.11 | 0.01 |
| **LPI** | **0.39 (0.13, 0.66)** | **0.00** | **0.02** | 0.04 |  | 0.32 (-0.15, 0.78) | 0.18 | 0.32 | 0 |
| **PS** | 0.27 (-0.00, 0.55) | 0.05 | 0.07 | 0.04 |  | 0.14 (-0.02, 0.29) | 0.08 | 0.25 | 0.01 |
| **PG** | 0.31 (0.01, 0.61) | **0.04** | 0.07 | 0.04 |  | 0.21 (-0.02, 0.44) | 0.07 | 0.24 | 0.01 |
| **CE** | **0.33 (0.11, 0.55)** | **0.00** | **0.02** | 0.04 |  | 0.12 (-0.44, 0.67) | 0.68 | 0.75 | 0 |
| **DE** | 0.08 (-0.28, 0.43) | 0.67 | 0.69 | 0.04 |  | -0.08 (-0.57, 0.40) | 0.73 | 0.79 | 0 |
| **FFA** | **0.38 (0.06, 0.70)** | **0.02** | **0.04** | 0.04 |  | 0.25 (-0.18, 0.69) | 0.25 | 0.39 | 0 |
| **Acylcarnitine** | **0.81 (0.50, 1.12)** | **0.00** | **0.00** | 0.07 |  | **0.65 (0.33, 0.97)** | **0.00** | **0.00** | 0.04 |
| **DG** | **0.43 (0.09, 0.78)** | **0.01** | **0.03** | 0.04 |  | **0.29 (0.05, 0.53)** | **0.02** | 0.11 | 0.01 |
| **TG(SIM)** | **0.31 (0.06, 0.55)** | **0.01** | **0.03** | 0.05 |  | 0.29 (-0.05, 0.63) | 0.09 | 0.26 | 0.01 |
| **TG [NL]** | **0.30 (0.06, 0.53)** | **0.01** | **0.03** | 0.05 |  | 0.23 (-0.05, 0.51) | 0.10 | 0.27 | 0.01 |
| **TG(O)** | 0.17 (-0.26, 0.60) | 0.43 | 0.47 | 0.04 |  | 0.09 (-0.49, 0.67) | 0.77 | 0.81 | 0 |
| **TG(O) [NL]** | **0.70 (0.19, 1.20)** | **0.01** | **0.02** | 0.04 |  | 0.18 (-0.12, 0.48) | 0.24 | 0.39 | 0 |
| **OxSpecies** | 0.23 (-0.00, 0.46) | 0.05 | 0.07 | 0.04 |  | -0.12 (-0.33, 0.09) | 0.27 | 0.40 | 0 |

**Note:** AMD, adjusted mean difference in lipid concentration (log2 transformed with 95% CI) per unit increase in CBCL raw score; CBCL-ADHP, Child Behavior Checklist attention-deficit/hyperactivity problems subscale; CBCL-ASP, Child Behavior Checklist autism spectrum problems subscale.

Model adjusted for child sex, gestational age (weeks) at birth, minutes from cord blood sample collection to storage, days cord blood sample stored, maternal blood contamination and child's age at CBCL.

***Supplementary Table 7.1:* The association between the cord blood lipid module eigenlipids and subsequent SDQ outcomes at 4 years.**

|  | | **SDQ-Hyperactivity** | | | |  | **SDQ-Peer Problems** | | | | |  | **SDQ-Prosocial** | | | | |
| --- | --- | --- | --- | --- | --- | --- | --- | --- | --- | --- | --- | --- | --- | --- | --- | --- | --- |
| **Cord blood lipid profile** | ***P*-value** | | ***Q*-value** | **R^2^** | **AIC** |  | **AMD  (95% CI)** | ***P*-value** | ***Q*-value** | **R^2^** | **AIC** |  | **AMD  (95% CI)** | ***P*-value** | ***Q*-Value** | **R^2^** | **AIC** |
| **Single eigenlipid class**  **(hub lipid's class)** | **0.001** | | **0.022** | 0.06 | 2899.20 |  | **0.13 (0.01, 0.26)** | **0.04** | 0.49 | 0.03 | 2334.33 |  | **-0.17 (-0.32, -0.02)** | **0.03** | 0.13 | 0.04 | 2620.23 |
| Cyan-AC | 0.22 | | 0.42 | 0.03 | 2909.27 |  | 0.1 (-0.03, 0.23) | 0.15 | 0.49 | 0.02 | 2336.54 |  | **-0.17 (-0.31, -0.03)** | **0.02** | 0.13 | 0.04 | 2620.19 |
| Turquoise-PE(hub) | **0.005** | | **0.04** | 0.04 | 2903.19 |  | 0.07 (-0.06, 0.20) | 0.29 | 0.67 | 0.02 | 2337.61 |  | -0.14 (-0.28, 0.00) | 0.06 | 0.13 | 0.04 | 2621.87 |
| Brown-TG(hub) | 0.17 | | 0.42 | 0.03 | 2908.69 |  | 0.09 (-0.03, 0.21) | 0.16 | 0.49 | 0.02 | 2336.82 |  | **-0.17 (-0.31, -0.02)** | **0.02** | 0.20 | 0.04 | 2620.11 |
| Magenta-PS(hub) | 0.23 | | 0.42 | 0.03 | 2909.42 |  | 0.11 (-0.01, 0.23) | 0.08 | 0.49 | 0.02 | 2335.68 |  | -0.13 (-0.27, 0.01) | 0.07 | 0.20 | 0.03 | 2621.86 |
| Blue-SMD(hub) | 0.47 | | 0.71 | 0.03 | 2910.24 |  | 0.02 (-0.10, 0.13) | 0.79 | 0.82 | 0.02 | 2338.76 |  | 0.01 (-0.15, 0.17) | 0.91 | 0.36 | 0.02 | 2625.01 |
| Red-CE(hub) | 0.92 | | 0.92 | 0.03 | 2910.73 |  | 0.05 (-0.07, 0.18) | 0.40 | 0.67 | 0.02 | 2338.03 |  | -0.05 (-0.20, 0.10) | 0.49 | 0.46 | 0.03 | 2624.53 |
| Green-LPC(hub) | 0.82 | | 0.91 | 0.03 | 2910.69 |  | 0.03 (-0.09, 0.15) | 0.62 | 0.82 | 0.02 | 2338.59 |  | -0.09 (-0.24, 0.07) | 0.28 | 0.46 | 0.03 | 2623.80 |
| Midnightblue-LPC | 0.81 | | 0.91 | 0.03 | 2910.69 |  | 0.02 (-0.10, 0.13) | 0.80 | 0.82 | 0.02 | 2338.76 |  | -0.11 (-0.26, 0.04) | 0.14 | 0.46 | 0.03 | 2622.92 |
| Black-TG(o) | 0.40 | | 0.67 | 0.03 | 2910.07 |  | 0.1 (-0.04, 0.23) | 0.16 | 0.49 | 0.03 | 2336.55 |  | -0.09 (-0.26, 0.07) | 0.27 | 0.65 | 0.03 | 2623.63 |
| Purple-FFA | **0.01** | | **0.05** | 0.04 | 2904.16 |  | -0.02 (-0.15, 0.11) | 0.81 | 0.82 | 0.02 | 2338.76 |  | -0.04 (-0.20, 0.11) | 0.59 | 0.66 | 0.03 | 2624.72 |
| Yellow-TG(hub) | 0.64 | | 0.88 | 0.03 | 2910.55 |  | 0.06 (-0.06, 0.18) | 0.34 | 0.67 | 0.02 | 2337.90 |  | -0.02 (-0.17, 0.13) | 0.80 | 0.73 | 0.02 | 2624.96 |
| Greenyellow-LPC(hub) | 0.14 | | 0.41 | 0.03 | 2908.61 |  | 0.06 (-0.07, 0.19) | 0.38 | 0.67 | 0.02 | 2337.93 |  | 0.01 (-0.15, 0.16) | 0.94 | 0.93 | 0.03 | 2625.01 |
| Pink-LPC(hub) | 0.85 | | 0.91 | 0.03 | 2910.71 |  | 0.02 (-0.12, 0.16) | 0.74 | 0.82 | 0.02 | 2338.69 |  | 0.06 (-0.09, 0.21) | 0.43 | 0.94 | 0.03 | 2624.47 |
| Tan-LPC(hub) | **0.03** | | **0.11** | 0.03 | 2906.68 |  | 0.01 (-0.10, 0.12) | 0.82 | 0.82 | 0.02 | 2338.78 |  | -0.09 (-0.22, 0.05) | 0.22 | 0.94 | 0.03 | 2623.60 |
| Salmon-HexCer |  | |  |  |  |  |  |  |  |  |  |  |  |  |  |  |  |

**Note**: AMD, adjusted mean difference in module eigenlipid (SD units with 95% CI) per unit increase in CBCL raw score; R^2^, variation in the outcome explained by the predictors; AIC, Akaike information criterion; AC, Acylcarnitine; PE, Phosphatidylethanolamine; TG, Triglyceride; PS, Phosphatidylserine; SM, Sphingomyelin; CE, Cholesteryl esters; LPC, Lysophosphatidylcholine; TG(o), Alkyldiacylglycerol; FFA, Free Fatty Acid; HexCer, Hexosylceramide; SDQ, Strength and Difficulties Questionnaire.

^1^Models adjusted for child sex, gestational age (weeks) at birth, minutes from cord blood sample collection to storage, days cord blood sample stored, maternal blood contamination, child's age at 4-year interview.

***Supplementary Table 7.2:*** **The association between the cord blood lipid module eigenlipids and subsequent SDQ outcomes at 4 years (Set A confounders).**

| **Cord blood lipid profile** | **SDQ-Hyperactivity^1^** | | | |  | **SDQ-Peer Problems^1^** | | | |  | **SDQ-Prosocial^1^** | | | |
| --- | --- | --- | --- | --- | --- | --- | --- | --- | --- | --- | --- | --- | --- | --- |
| **Single eigenlipid class**  **(hub lipid's class)** | **AMD  (95% CI)** | ***P*-value** | **R^2^** | **AIC** |  | **AMD  (95% CI)** | ***P-*value** | **R^2^** | **AIC** |  | **AMD  (95% CI)** | ***P*-value** | **R^2^** | **AIC** |
| Cyan-AC | 0.27 (-0.03, 0.57) | 0.08 | 0.11 | 1517.46 |  | 0.12 (-0.08, 0.31) | 0.24 | 0.09 | 1239.77 |  | -0.28 (-0.49, -0.07) | 0.008 | 0.07 | 1359.93 |
| Turquoise-PE(hub) | 0.20 (-0.05, 0.45) | 0.12 | 0.1 | 1518.95 |  | 0.18 (-0.00, 0.36) | 0.06 | 0.1 | 1237.54 |  | -0.37 (-0.57, -0.16) | 0 | 0.09 | 1354.88 |
| Brown-TG(hub) | 0.14 (-0.10, 0.37) | 0.27 | 0.1 | 1520.02 |  | 0.05 (-0.13, 0.22) | 0.61 | 0.09 | 1241.08 |  | -0.24 (-0.43, -0.04) | 0.02 | 0.07 | 1361.32 |
| Magenta-PS(hub) | 0.26 (-0.01, 0.52) | 0.06 | 0.11 | 1517.61 |  | 0.02 (-0.16, 0.20) | 0.84 | 0.09 | 1241.32 |  | -0.28 (-0.47, -0.09) | 0.004 | 0.07 | 1359.76 |
| Blue-SMD(hub) | 0.15 (-0.11, 0.41) | 0.26 | 0.1 | 1519.79 |  | **0.22 (0.06, 0.39)** | **0.009** | **0.1** | **1234.83** |  | -0.28 (-0.49, -0.06) | 0.01 | 0.07 | 1359.68 |
| Red-CE(hub) | 0.04 (-0.25, 0.33) | 0.78 | 0.1 | 1520.99 |  | 0.10 (-0.07, 0.28) | 0.24 | 0.09 | 1240.00 |  | -0.14 (-0.39, 0.11) | 0.27 | 0.06 | 1364.74 |
| Green-LPC(hub) | -0.01 (-0.29, 0.27) | 0.94 | 0.1 | 1521.08 |  | 0.16 (-0.03, 0.35) | 0.1 | 0.09 | 1238.57 |  | -0.14 (-0.38, 0.09) | 0.24 | 0.06 | 1364.98 |
| Midnightblue-LPC | -0.14 (-0.44, 0.15) | 0.34 | 0.1 | 1520.10 |  | 0.09 (-0.09, 0.28) | 0.33 | 0.09 | 1240.43 |  | -0.12 (-0.36, 0.12) | 0.32 | 0.06 | 1365.45 |
| Black-TG(o) | -0.09 (-0.36, 0.19) | 0.53 | 0.1 | 1520.69 |  | 0.05 (-0.13, 0.23) | 0.6 | 0.09 | 1241.09 |  | -0.26 (-0.47, -0.05) | 0.01 | 0.07 | 1360.81 |
| Purple-FFA | 0.04 (-0.23, 0.31) | 0.76 | 0.1 | 1520.99 |  | 0.12 (-0.08, 0.32) | 0.26 | 0.09 | 1239.80 |  | -0.16 (-0.42, 0.09) | 0.2 | 0.06 | 1364.39 |
| Yellow-TG(hub) | 0.18 (-0.10, 0.45) | 0.2 | 0.1 | 1519.30 |  | -0.01 (-0.19, 0.17) | 0.9 | 0.09 | 1241.34 |  | -0.14 (-0.35, 0.08) | 0.21 | 0.06 | 1364.88 |
| Greenyellow-LPC(hub) | 0.00 (-0.25, 0.25) | 0.99 | 0.1 | 1521.08 |  | 0.18 (0.02, 0.33) | 0.03 | 0.1 | 1237.44 |  | -0.07 (-0.27, 0.14) | 0.52 | 0.06 | 1366.14 |
| Pink-LPC(hub) | 0.06 (-0.20, 0.32) | 0.66 | 0.1 | 1520.90 |  | 0.10 (-0.07, 0.27) | 0.26 | 0.09 | 1240.18 |  | -0.04 (-0.26, 0.18) | 0.71 | 0.06 | 1366.40 |
| Tan-LPC(hub) | -0.03 (-0.27, 0.20) | 0.78 | 0.1 | 1521.02 |  | 0.08 (-0.11, 0.27) | 0.4 | 0.09 | 1240.52 |  | 0.08 (-0.14, 0.29) | 0.48 | 0.06 | 1366.05 |
| Salmon-HexCer | **0.32 (0.10, 0.54)** | **0.005** | **0.12** | **1513.71** |  | 0.10 (-0.04, 0.25) | 0.16 | 0.09 | 1239.58 |  | -0.10 (-0.28, 0.09) | 0.31 | 0.06 | 1365.50 |

**Note:** AMD, adjusted mean difference in module eigenlipid (SD units with 95% CI) per unit increase in CBCL raw score; R^2^, variation in the outcome explained by the predictors; AIC, Akaike information criterion; AC, Acylcarnitine; PE Phosphatidylethanolamine; TG, Triglyceride; PS, Phosphatidylserine; SM, Sphingomyelin; CE, Cholesteryl esters; LPC, Lysophosphatidylcholine; TG(o), Alkyldiacylglycerol; FFA, Free Fatty Acid; HexCer, Hexosylceramide; SDQ, Strength and Difficulties Questionnaire.

^1^ Model adjusted for child sex, gestational age (weeks) at birth, minutes from cord blood sample collection to storage, days cord blood sample stored, maternal blood contamination, child's age at 4 year interview, **household income (decreasing), high weight gain during pregnancy (>20kg vs <20kg), Apgar score at 5 minutes, maternal age, lone parent during pregnancy(yes vs no), perceived stress score during trimester 1/2 and smoking during pregnancy (any vs none).**

***Supplementary Table 7.3:*** **The association between the cord blood lipid module eigenlipids and subsequent SDQ outcomes at 4 years (Set B confounders).**

| **Cord blood lipid profile** | **SDQ-Hyperactivity^1^** | | | |  | **SDQ-Peer Problems^1^** | | | |  | **SDQ-Prosocial^1^** | | | |
| --- | --- | --- | --- | --- | --- | --- | --- | --- | --- | --- | --- | --- | --- | --- |
| **Single eigenlipid model**  **(hub lipid's class)** | **AMD  (95% CI)** | ***P-*value** | **R^2^** | **AIC** |  | **AMD  (95% CI)** | ***P*-value** | **R^2^** | **AIC** |  | **AMD  (95% CI)** | ***P*-value** | **R^2^** | **AIC** |
| Cyan-AC | 0.21 (-0.06, 0.48) | 0.12 | 0.06 | 1922.50 |  | 0.14 (-0.04, 0.31) | 0.12 | 0.06 | 1572.53 |  | **-0.22 (-0.41, -0.02)** | **0.03** | **0.06** | **1733.00** |
| Turquoise-PE(hub) | 0.08 (-0.13, 0.30) | 0.45 | 0.06 | 1924.82 |  | 0.15 (-0.02, 0.32) | 0.08 | 0.06 | 1571.67 |  | **-0.31 (-0.50, -0.11)** | **0.002** | **0.07** | **1727.88** |
| Brown-TG(hub) | 0.10 (-0.13, 0.34) | 0.40 | 0.06 | 1924.63 |  | 0.07 (-0.11, 0.24) | 0.45 | 0.05 | 1574.48 |  | **-0.23 (-0.42, -0.04)** | **0.02** | **0.06** | **1732.28** |
| Magenta-PS(hub) | 0.20 (-0.04, 0.44) | 0.10 | 0.06 | 1922.62 |  | 0.07 (-0.09, 0.22) | 0.41 | 0.05 | 1574.44 |  | **-0.22 (-0.40, -0.03)** | **0.02** | **0.06** | **1732.54** |
| Blue-SMD(hub) | 0.04 (-0.18, 0.26) | 0.71 | 0.06 | 1925.15 |  | **0.19 (0.04, 0.34)** | **0.02** | **0.06** | 1569.37 |  | **-0.25 (-0.43, -0.07)** | **0.006** | **0.07** | **1730.43** |
| Red-CE(hub) | -0.06 (-0.31, 0.19) | 0.66 | 0.06 | 1925.05 |  | 0.08 (-0.07, 0.24) | 0.29 | 0.05 | 1574.07 |  | -0.15 (-0.36, 0.06) | 0.17 | 0.06 | 1735.13 |
| Green-LPC(hub) | -0.07 (-0.31, 0.17) | 0.57 | 0.06 | 1924.95 |  | 0.16 (-0.02, 0.33) | 0.08 | 0.06 | 1571.46 |  | -0.13 (-0.33, 0.06) | 0.19 | 0.05 | 1735.61 |
| Midnightblue-LPC | -0.13 (-0.38, 0.11) | 0.28 | 0.06 | 1924.05 |  | 0.09 (-0.08, 0.26) | 0.3 | 0.05 | 1573.86 |  | -0.15 (-0.34, 0.05) | 0.14 | 0.06 | 1735.19 |
| Black-TG(o) | -0.13 (-0.38, 0.11) | 0.27 | 0.06 | 1924.13 |  | 0.04 (-0.13, 0.20) | 0.68 | 0.05 | 1574.94 |  | **-0.23 (-0.42, -0.04)** | **0.02** | **0.06** | **1732.25** |
| Purple-FFA | 0.04 (-0.21, 0.28) | 0.77 | 0.06 | 1925.18 |  | 0.13 (-0.05, 0.31) | 0.17 | 0.06 | 1572.62 |  | -0.07 (-0.31, 0.16) | 0.54 | 0.05 | 1736.87 |
| Yellow-TG(hub) | 0.17 (-0.08, 0.42) | 0.17 | 0.06 | 1923.31 |  | -0.01 (-0.18, 0.16) | 0.93 | 0.05 | 1575.10 |  | -0.12 (-0.32, 0.07) | 0.22 | 0.05 | 1735.85 |
| Greenyellow-LPC(hub) | -0.08 (-0.30, 0.14) | 0.49 | 0.06 | 1924.86 |  | 0.11 (-0.04, 0.27) | 0.16 | 0.05 | 1573.15 |  | -0.03 (-0.20, 0.15) | 0.78 | 0.05 | 1737.36 |
| Pink-LPC(hub) | 0.10 (-0.13, 0.33) | 0.41 | 0.06 | 1924.64 |  | 0.12 (-0.04, 0.29) | 0.13 | 0.06 | 1572.71 |  | -0.02 (-0.21, 0.17) | 0.84 | 0.05 | 1737.38 |
| Tan-LPC(hub) | -0.02 (-0.23, 0.19) | 0.85 | 0.06 | 1925.25 |  | 0.06 (-0.12, 0.23) | 0.52 | 0.05 | 1574.59 |  | 0.03 (-0.16, 0.23) | 0.72 | 0.05 | 1737.29 |
| Salmon-HexCer | **0.24 (0.03, 0.45)** | **0.02** | **0.07** | **1920.70** |  | 0.08 (-0.07, 0.22) | 0.3 | 0.05 | 1574.04 |  | -0.09 (-0.27, 0.08) | 0.3 | 0.05 | 1736.32 |

**Note:** AMD, adjusted mean difference in module eigenlipid (SD units with 95% CI) per unit increase in CBCL raw score; R^2^, variation in the outcome explained by the predictors; AIC, Akaike information criterion; AC, Acylcarnitine; PE Phosphatidylethanolamine; TG, Triglyceride; PS, Phosphatidylserine; SM, Sphingomyelin; CE, Cholesteryl esters; LPC, Lysophosphatidylcholine; TG(o), Alkyldiacylglycerol; FFA, Free Fatty Acid; HexCer, Hexosylceramide; SDQ, Strength and Difficulties Questionnaire.

^1^ Model adjusted for child sex, gestational age (weeks) at birth, minutes from cord blood sample collection to storage, days cord blood sample stored, maternal blood contamination, age at CBCL testing, **House hold income (decreasing), low birth weight (>2.5kg vs <2.5kg), high weight gain during pregnancy (>20kg vs <20kg), multiparity and Apgar score at 5 minutes.**

| ***Supplementary Table S8.1:*** **Mediation by either NOPMS at birth or GlycA at birth of the relationships between the lipid module eigenlipids and CBCL-ADHP.**   \| **Early life factor** \| **CBCL-ADHP (c)^a^** \| \|  \| **Mediation analysis** \| \| \| \| \| \| \| \| --- \| --- \| --- \| --- \| --- \| --- \| --- \| --- \| --- \| --- \| --- \| \| **(exposure vs reference)** \| **Total effect** \| \|  \| **Direct effect (c’)** \| \|  \| **Indirect effect (ab)** \| \|  \| **Percentage mediated** \| \|  \| **𝜷 (95% CI)** \| ***P-*value** \|  \| **𝜷 (95% CI)** \| ***P*-value** \|  \| **𝜷 (95% CI)** \| ***P*-value** \|  \| \| **NOPMS at birth (PC1)** \|  \|  \|  \|  \|  \|  \|  \|  \|  \|  \| \| **Cyan-AC** \| **16.6 (9.82, 23.38)** \| **<0.0001** \|  \| **17.86 (9.5, 26.21)** \| **<0.0001** \|  \| -1.26 (-6.31, 3.79) \| 0.63 \|  \|  \| \| **Turquoise-PE** \| **13.83 (6.04, 21.62)** \| **0.0005** \|  \| **11.99 (4.02, 19.96)** \| **0.003** \|  \| 1.84 (-0.43, 4.11) \| 0.11 \|  \| 0.13 \| \| Brown-TG(hub) \| **9.69 (3.14, 16.24)** \| **0.004** \|  \| **7.23 (0.04, 14.43)** \| **0.049** \|  \| 2.46 (-0.24, 5.16) \| 0.07 \|  \| 0.25 \| \| Magenta-PS(hub) \| **10.67 (4.02, 17.32)** \| **0.002** \|  \| **8.05 (0.97, 15.12)** \| **0.03** \|  \| 2.62 (-0.75, 5.99) \| 0.13 \|  \| 0.25 \| \| Blue-SMD(hub) \| **10.99 (4.62, 17.37)** \| **0.0007** \|  \| **9.67 (3.17, 16.18)** \| **0.004** \|  \| 1.32 (-0.04, 2.68) \| 0.06 \|  \| 0.12 \| \| Green-LPC(hub) \| **7.99 (1.58, 14.4)** \| **0.02** \|  \| **6.49 (-0.04, 13.02)** \| **0.05** \|  \| 1.5 (-0.04, 3.04) \| 0.06 \|  \| 0.19 \| \| Midnightblue-LPC \| **7.28 (0.72, 13.84)** \| **0.03** \|  \| 5.66 (-1.04, 12.36) \| 0.10 \|  \| **1.62 (0.08, 3.16)** \| **0.04** \|  \| 0.22 \| \| Black-TG(o) \| **6.94 (0.78, 13.11)** \| **0.03** \|  \| 5.63 (-0.32, 11.58) \| 0.06 \|  \| 1.31 (-0.01, 2.64) \| 0.05 \|  \| 0.19 \| \| Purple-FFA \| **9.06 (2.17, 15.95)** \| **0.01** \|  \| 6 (-1.91, 13.92) \| 0.14 \|  \| 3.05 (-0.42, 6.53) \| 0.09 \|  \| 0.34 \| \|  \|  \|  \|  \|  \|  \|  \|  \|  \|  \|  \| \| **GlycA at birth** \|  \|  \|  \|  \|  \|  \|  \|  \|  \|  \| \| **Cyan-AC** \| **16.6 (10.24, 22.96)** \| **<0.0001** \|  \| **13.9 (6.32, 21.49)** \| **0.0003** \|  \| 2.7 (-1.75, 7.15) \| 0.23 \|  \| 0.16 \| \| **Turquoise-PE** \| **12.23 (4.34, 20.12)** \| **0.002** \|  \| 6.67 (-2.28, 15.62) \| 0.14 \|  \| 5.56 (-0.26, 11.39) \| 0.06 \|  \| 0.45 \| \| Brown-TG(hub) \| **9.24 (2.47, 16.01)** \| **0.008** \|  \| 1.92 (-6.28, 10.11) \| 0.65 \|  \| **7.32 (1.28, 13.36)** \| **0.02** \|  \| 0.79 \| \| Magenta-PS(hub) \| **9.09 (2.79, 15.4)** \| **0.005** \|  \| 4.1 (-2.82, 11.01) \| 0.25 \|  \| **5 (0.85, 9.14)** \| **0.02** \|  \| 0.55 \| \| Blue-SMD(hub) \| **10.16 (3.5, 16.83)** \| **0.003** \|  \| **6.3 (-0.73, 13.33)** \| **0.08** \|  \| **3.86 (0.64, 7.08)** \| **0.02** \|  \| 0.38 \| \| Green-LPC(hub) \| **8.12 (1.81, 14.43)** \| **0.01** \|  \| 5.83 (-0.84, 12.49) \| 0.09 \|  \| **2.29 (0.16, 4.42)** \| **0.04** \|  \| 0.28 \| \| Midnightblue-LPC \| **7.8 (1.61, 13.99)** \| **0.01** \|  \| 5.46 (-1.09, 12.01) \| 0.10 \|  \| **2.34 (0.35, 4.33)** \| **0.02** \|  \| 0.3 \| \| Black-TG(o) \| **7.69 (1.49, 13.89)** \| **0.02** \|  \| 3.06 (-2.95, 9.08) \| 0.32 \|  \| **4.62 (0.98, 8.26)** \| **0.01** \|  \| 0.6 \| \| Purple-FFA \| **7.11 (-0.35, 14.56)** \| **0.06** \|  \| 0.7 (-7.37, 8.76) \| 0.87 \|  \| **6.41 (1.88, 10.95)** \| **0.006** \|  \| 0.9 \| |
| --- | --- | --- | --- | --- | --- | --- | --- | --- | --- | --- | --- | --- | --- | --- | --- | --- | --- | --- | --- | --- | --- | --- | --- | --- | --- | --- | --- | --- | --- | --- | --- | --- | --- | --- | --- | --- | --- | --- | --- | --- | --- | --- | --- | --- | --- | --- | --- | --- | --- | --- | --- | --- | --- | --- | --- | --- | --- | --- | --- | --- | --- | --- | --- | --- | --- | --- | --- | --- | --- | --- | --- | --- | --- | --- | --- | --- | --- | --- | --- | --- | --- | --- | --- | --- | --- | --- | --- | --- | --- | --- | --- | --- | --- | --- | --- | --- | --- | --- | --- | --- | --- | --- | --- | --- | --- | --- | --- | --- | --- | --- | --- | --- | --- | --- | --- | --- | --- | --- | --- | --- | --- | --- | --- | --- | --- | --- | --- | --- | --- | --- | --- | --- | --- | --- | --- | --- | --- | --- | --- | --- | --- | --- | --- | --- | --- | --- | --- | --- | --- | --- | --- | --- | --- | --- | --- | --- | --- | --- | --- | --- | --- | --- | --- | --- | --- | --- | --- | --- | --- | --- | --- | --- | --- | --- | --- | --- | --- | --- | --- | --- | --- | --- | --- | --- | --- | --- | --- | --- | --- | --- | --- | --- | --- | --- | --- | --- | --- | --- | --- | --- | --- | --- | --- | --- | --- | --- | --- | --- | --- | --- | --- | --- | --- | --- | --- | --- | --- | --- | --- | --- | --- | --- | --- | --- | --- | --- | --- | --- | --- | --- | --- | --- | --- | --- | --- | --- | --- | --- | --- | --- | --- | --- | --- | --- | --- | --- | --- | --- | --- | --- | --- | --- | --- | --- | --- | --- | --- | --- | --- | --- | --- | --- | --- |

The indirect effect (ab) is the amount provided by NOMPS at birth or GlycA at birth.

Proportion mediated was calculated when the direct effect and indirect effect where in the same direction.

**Note:** NOPMS, non-oxidative pyruvate metabolism score, PC1 of pyruvate, lactate, acetate and alanine. ^6^ (all loadings were in the positive direction and therefore higher PC1 values were interpreted as increased pathway activity); CBCL-ADHP, Child Behavior Checklist attention-deficit/hyperactivity problems subscale.

^a^ Models adjusted for child sex, gestational age (weeks) at birth, minutes from cord blood sample collection to storage, days cord blood sample stored and maternal blood contamination.

***Supplementary Table S8.2:*** **Mediation by either NOPMS at birth or GlycA at birth of the relationships between the lipid module eigenlipids and CBCL-ASP.**

| **Early life factor** | **CBCL-ASP (c)^a^** | |  | **Mediation analysis** | | | | | | |
| --- | --- | --- | --- | --- | --- | --- | --- | --- | --- | --- |
| **(exposure vs reference)** | **Total effect** | |  | **Direct effect (c’)** | |  | **Indirect effect (ab)** | |  | **Percentage mediated** |
|  | **𝜷 (95% CI)** | ***P*-value** |  | **𝜷 (95% CI)** | ***P*-value** |  | **𝜷 (95% CI)** | ***P*-value** |  |  |
| **NOPMS at birth (PC1)** |  |  |  |  |  |  |  |  |  |  |
| **Cyan-AC** | **10.57 (4.68, 16.47)** | **0.0004** |  | **11.84 (4.3, 19.38)** | **0.002** |  | -1.27 (-5.75, 3.22) | 0.58 |  | - |
| **Turquoise-PE** | **7.33 (1.3, 13.36)** | **0.02** |  | **6.17 (0, 12.34)** | **0.05** |  | 1.16 (-0.79, 3.11) | 0.24 |  | 0.16 |
| Brown-TG(hub) | **6.43 (0.68, 12.18)** | **0.03** |  | 5.09 (-1.29, 11.47) | 0.12 |  | 1.34 (-0.93, 3.62) | 0.25 |  | 0.21 |
| Magenta-PS(hub) | **6.04 (0.53, 11.56)** | **0.03** |  | 4.44 (-1.2, 10.09) | 0.12 |  | 1.6 (-1.19, 4.39) | 0.26 |  | 0.26 |
|  |  |  |  |  |  |  |  |  |  |  |
| **GlycA at birth** |  |  |  |  |  |  |  |  |  |  |
| **Cyan-AC** | **11.77 (6.09, 17.45)** | **0.00005** |  | **11.57 (4.85, 18.28)** | **0.0007** |  | 0.2 (-2.46, 2.86) | 0.88 |  | 0.02 |
| **Turquoise-PE** | **7.33 (1.3, 13.36)** | **0.02** |  | **6.17 (0, 12.34)** | **0.05** |  | 1.16 (-0.79, 3.11) | 0.24 |  | 0.16 |
| Brown-TG(hub) | **6.43 (0.68, 12.18)** | **0.03** |  | 5.09 (-1.29, 11.47) | 0.12 |  | 1.34 (-0.93, 3.62) | 0.25 |  | 0.21 |
| Magenta-PS(hub) | **6.04 (0.53, 11.56)** | **0.03** |  | 4.44 (-1.2, 10.09) | 0.12 |  | 1.6 (-1.19, 4.39) | 0.26 |  | 0.26 |

The indirect effect (ab) is the amount provided by NOPMS at birth or GlycA at birth.

Proportion mediated was calculated when the direct effect and indirect effect where in the same direction.

**Note:** NOPMS, non-oxidative pyruvate metabolism score, PC1 of pyruvate, lactate, acetate and alanine. ^6^ (all loadings were in the positive direction and therefore higher PC1 values were interpreted as increased pathway activity); CBCL-ASP, Child Behavior Checklist autism spectrum problems subscale.

^a^ Models adjusted for child sex, gestational age (weeks) at birth, minutes from cord blood sample collection to storage, days cord blood sample stored and maternal blood contamination.

***Supplementary Table S9:* Mutually adjusted model of all eigenlipids and subsequent CBCL-ADHP and CBCL-ASP outcomes at 2 years.**

| **Cord blood lipid profile** | **CBCL-ADHP** | |  | **CBCL-ASP** | |
| --- | --- | --- | --- | --- | --- |
| **Mutually adjusted eigenlipid model**  **(hub lipid's class)** | **AMD (95% CI)** | ***P-*value** |  | **ADM (95% CI)** | ***P*-value** |
| Cyan-AC | **0.53 (0.25, 0.82)** | **<0·0001** |  | **0.4 (0.14, 0.65)** | **<0·0001** |
| Turquoise-PE(hub) | 0.36 (-0.36, 1.09) | 0.33 |  | **0.64 (0.08, 1.20)** | **0.03** |
| Brown-TG(hub) | -0.04 (-0.53, 0.46) | 0.88 |  | 0.13 (-0.24, 0.50) | 0.48 |
| Magenta-PS(hub) | 0.01 (-0.31, 0.34) | 0.93 |  | -0.06 (-0.32, 0.20) | 0.64 |

**Note:** AMD, adjusted mean difference in module eigenlipid (SD units with 95% CI) per unit increase in CBCL raw score; Acylcarnitine; PE, Phosphidytalethanolamine; TG, Triglyceride; PS, Phosphatidylserine; SM, Sphingomyelin; CE, Cholesteryl esters; LPC, Lysophosphotidylcholine; TG(o), Alkyldiacylglycerol; FFA, Free Fatty Acid; HexCer, Hexosylceramide; CBCL-ADHP, Child Behavior Checklist attention-deficit/hyperactivity problems subscale; CBCL-ASP, Child Behavior Checklist autism spectrum problems subscale.

Top four lipid modules shown. This model has been mutually adjusted for all other lipid modules (Cyan-AC, Turquoise-PE(hub), Brown-TG(hub), Magenta-PS(hub), Blue-SMD(hub), Red-CE(hub), Green-LPC(hub), Midnightblue-LPC, Black-TG(o), Purple-FFA, Yellow-TG(hub), Greenyellow-LPC(hub), Pink-LPC(hub), Tan-LPC(hub), Salmon-HexCer) and covariates child sex, gestational age (weeks) at birth, minutes from cord blood sample collection to storage, days cord blood sample stored, maternal blood contamination and age at CBCL testing.





**Figure S2. Correlation matrix of the lipid modules , neurodevelopmental outcomes and covariates used in regression models.**

*, p<0.05; **, p<0.01; ***, p<0.001; ****, p<0.0001.

**Note:** AC, Acylcarnitine; PE, Phosphatidylethanolamine; TG, Triglyceride; PS, Phosphatidylserine; SM, Sphingomyelin; CE, Cholesteryl esters; LPC, Lysophosphotidylcholine; TG(o), Alkyldiacylglycerol; FFA, Free Fatty Acid; HexCer, Hexosylceramide; CBCL-ADHP, Child Behavior Checklist attention-deficit/hyperactivity problems subscale; CBCL-ASP, Child Behavior Checklist autism spectrum problems subscale; SDQ, Strength and Difficulties Questionnaire, Min, minutes, col, collection. See Supplementary Table S4.3 for covariate details.

**References**

1. Evans D, Chaix B, Lobbedez T, Verger C, Flahault A. Combining directed acyclic graphs and the change-in-estimate procedure as a novel approach to adjustment-variable selection in epidemiology. *BMC medical research methodology* 2012; **12**: 156.

2. Ponsonby A-L. Reflection on modern methods: building causal evidence within high-dimensional molecular epidemiological studies of moderate size. *International journal of epidemiology* 2021; **50**(3): 1016-29.

3. Langfelder P, Horvath S. WGCNA: an R package for weighted correlation network analysis. *BMC Bioinformatics* 2008; **9**(1): 559.

4. Giles G IP. Dietary questionnaire for epidemiological studies (version 2). The Cancer Council Victoria; 1996.

5. Dawson SL, O'Hely M, Jacka FN, et al. Maternal prenatal gut microbiota composition predicts child behaviour. *eBioMedicine* 2021; **68**.

6. Thomson S, Drummond K, O'Hely M, et al. Increased maternal non-oxidative energy metabolism mediates association between prenatal di-(2-ethylhexyl) phthalate (DEHP) exposure and offspring autism spectrum disorder symptoms in early life: A birth cohort study. *Environment International* 2023; **171**: 107678.
